# Revision of the subfamily Opiinae (Hymenoptera, Braconidae) from Hunan (China), including thirty-six new species and two new genera

**DOI:** 10.3897/zookeys.268.4071

**Published:** 2013-02-13

**Authors:** Xi-Ying Li, Cornelis van Achterberg, Ji-Cai Tan

**Affiliations:** 1 College of Bio-Safety Science and Technology, Hunan Agriculture University, Changsha 410128, China; 2 Department of Terrestrial Zoology, Naturalis Biodiversity Center, Postbus 9517, 2300 RA Leiden, The Netherlands

**Keywords:** Braconidae, Opiinae, *Apodesmia*, *Areotetes*, *Coleopioides*, *Coleopius*, *Fopius*, *Opiognathus*, *Opius*, *Phaedrotoma*, *Rhogadopsis*, *Utetes*, *Xynobius*, new species, new genus, new combinations, new names, key, distribution, Oriental, China, Hunan

## Abstract

The species of the subfamily Opiinae (Hymenoptera: Braconidae) from Hunan (Oriental China) are revised and illustrated. Thirty-six new species are described: *Apodesmia bruniclypealis* Li & van Achterberg, **sp. n.**, *Apodesmia melliclypealis* Li & van Achterberg, **sp. n.**, *Areotetes albiferus* Li & van Achterberg, **sp. n.**, *Areotetes carinuliferus* Li & van Achterberg, **sp. n.**, *Areotetes striatiferus* Li & van Achterberg, **sp. n.**, *Coleopioides diversinotum* Li & van Achterberg, **sp. n.**, *Coleopioides postpectalis* Li & van Achterberg, **sp. n.**, *Fopius dorsopiferus* Li, van Achterberg & Tan, **sp. n.**, *Indiopius chenae* Li & van Achterberg, **sp. n.**, *Opiognathus aulaciferus* Li & van Achterberg, **sp. n.**, *Opiognathus brevibasalis* Li & van Achterberg, **sp. n.**, *Opius crenuliferus* Li & van Achterberg, **sp. n.**, *Opius malarator* Li, van Achterberg & Tan, **sp. n.**, *Opius monilipalpis* Li & van Achterberg, **sp. n.**, *Opius pachymerus* Li & van Achterberg, **sp. n.**, *Opius songi* Li & van Achterberg, **sp. n.**, *Opius youi* Li & van Achterberg, **sp. n.**, *Opius zengi* Li & van Achterberg, **sp. n.**, *Phaedrotoma acuticlypeata* Li & van Achterberg, **sp. n.**, *Phaedrotoma angiclypeata* Li & van Achterberg, **sp. n.**, *Phaedrotoma antenervalis* Li & van Achterberg, **sp. n.**, *Phaedrotoma depressiclypealis* Li & van Achterberg, **sp. n.**, *Phaedrotoma flavisoma* Li & van Achterberg, **sp. n.**, *Phaedrotoma nigrisoma* Li & van Achterberg, **sp. n.**, *Phaedrotoma protuberator* Li & van Achterberg, **sp. n.**, *Phaedrotoma rugulifera* Li & van Achterberg, **sp. n.**, Li & van Achterberg,*Phaedrotoma striatinota* Li & van Achterberg, **sp. n.**, *Phaedrotoma vermiculifera* Li & van Achterberg, **sp. n.**, *Rhogadopsis latipennis* Li & van Achterberg, **sp. n.,** *Rhogadopsis longicaudifera* Li & van Achterberg, **sp. n.**, *Rhogadopsis maculosa* Li, van Achterberg & Tan, **sp. n.**, *Rhogadopsis obliqua* Li & van Achterberg, **sp. n.**, *Rhogadopsis sculpturator* Li & van Achterberg, **sp. n.**, *Utetes longicarinatus* Li & van Achterberg, **sp. n.** and *Xynobius notauliferus* Li & van Achterberg, **sp. n.** *Areotetes* van Achterberg & Li, **gen. n.** (type species: *Areotetes carinuliferus* **sp. n.**) and *Coleopioides* van Achterberg & Li, **gen. n.** (type species: *Coleopioides postpectalis* **sp. n.** are described. All species are illustrated and keyed. In total 30 species of Opiinae are sequenced and the cladograms are presented. *Neopius* Gahan, 1917, *Opiognathus* Fischer, 1972, *Opiostomus* Fischer, 1972, and *Rhogadopsis* Brèthes, 1913, are treated as a valid genera based on molecular and morphological differences. *Opius vittata* Chen & Weng, 2005 (not *Opius vittatus* Ruschka, 1915), *Opius ambiguus* Weng & Chen, 2005 (not Wesmael, 1835) and *Opius mitis* Chen & Weng, 2005 (not Fischer, 1963) are primary homonymsandarerenamed into *Phaedrotoma depressa* Li & van Achterberg, **nom. n.**, *Opius cheni* Li & van Achterberg, **nom. n.** and*Opius wengi* Li & van Achterberg, **nom. n.**, respectively. *Phaedrotoma terga* (Chen & Weng, 2005) **comb. n.**,*Diachasmimorpha longicaudata* (Ashmead, 1905) and *Biosteres pavitita* Chen & Weng, 2005, are reported new for Hunan, *Opiostomus aureliae* (Fischer, 1957) **comb. n.** is new for China and Hunan; *Xynobius maculipennis*(Enderlein, 1912) **comb. n.** is new for Hunan and continental China and *Rhogadopsis longuria* (Chen & Weng, 2005) **comb. n.** is new for Hunan. The following new combinations are given: *Apodesmia puncta* (Weng & Chen, 2005) **comb. n.**, *Apodesmia tracta* (Weng & Chen, 2005) **comb. n.**, *Areotetes laevigatus* (Weng & Chen, 2005) **comb. n.**, *Phaedrotoma dimidia* (Chen & Weng, 2005) **comb. n.**, *Phaedrotoma improcera* (Weng & Chen, 2005) **comb. n.**, *Phaedrotoma amputata* (Weng & Chen, 2005) **comb. n.**, *Phaedrotoma larga* (Weng & Chen, 2005) **comb. n.**, *Phaedrotoma osculas* (Weng & Chen, 2005) **comb. n.**, *Phaedrotoma postuma* (Chen & Weng, 2005) **comb. n.**, *Phaedrotoma rugulosa* (Chen & Weng, 2005) **comb. n.**, *Phaedrotoma tabularis* (Weng & Chen, 2005) **comb. n.**, *Rhogadopsis apii* (Chen & Weng, 2005) **comb. n.**, *Rhogadopsis dimidia* (Chen & Weng, 2005) **comb. n.,** *Rhogadopsis diutia* (Chen & Weng, 2005) **comb. n.**, *Rhogadopsis longuria* (Chen & Weng, 2005) **comb. n.**, *Rhogadopsis pratellae***(**Weng & Chen, 2005) **comb. n.**, *Rhogadopsis pratensis* (Weng & Chen, 2005) **comb. n.**, *Rhogadopsis sculpta* (Chen & Weng, 2005) **comb. n.**, *Rhogadopsis sulcifer* (Fischer, 1975) **comb. n.,** *Rhogadopsis tabidula*(Weng & Chen, 2005) **comb. n.,** *Xynobius complexus* (Weng & Chen, 2005) **comb. n.**, *Xynobius indagatrix* (Weng & Chen, 2005) **comb. n.,** *Xynobius multiarculatus* (Chen & Weng, 2005) **comb. n.**

The following (sub)genera are synonymised: Snoflakopius Fischer, 1972, Jucundopius Fischer, 1984, Opiotenes Fischer, 1998, and Oetztalotenes Fischer, 1998, with Opiostomus Fischer, 1971; Xynobiotenes Fischer, 1998, with Xynobius Foerster, 1862; Allotypus Foerster, 1862, Lemnaphilopius Fischer, 1972, Agnopius Fischer, 1982, and Cryptognathopius Fischer, 1984, with Apodesmia Foerster, 1862; Nosopoea Foerster, 1862, Tolbia Cameron, 1907, Brachycentrus Szépligeti, 1907, Baeocentrum Schulz, 1911, Hexaulax Cameron, 1910, Coeloreuteus Roman, 1910, Neodiospilus Szépligeti, 1911, Euopius Fischer, 1967, Gerius Fischer, 1972, Grimnirus Fischer, 1972, Hoenirus Fischer, 1972, Mimirus Fischer, 1972, Gastrosema Fischer, 1972, Merotrachys Fischer, 1972, Phlebosema Fischer, 1972, Neoephedrus Samanta, Tamili, Saha & Raychaudhuri, 1983, Adontopius Fischer, 1984, Kainopaeopius Fischer, 1986, Millenniopius Fischer, 1996, and Neotropopius Fischer, 1999, with Phaedrotoma Foerster, 1862.

## Introduction

The large subfamily Opiinae (Braconidae), with 1,981 valid species according to Yu et al. (2012), is a common group containing generally small (2-5 mm) parasitoid wasps of mainly mining or fruit-infesting dipterous larvae. It has a worldwide distribution and the world fauna has been reviewed by Fischer (1972b, 1977, 1986, 1987). Currently about 35 genera are used; Wharton (1988, 1997), van Achterberg (1997b, 2004a, 2004b), van Achterberg and Salvo (1997) and van Achterberg and Chen (2004) published updates or some additions for the existing keys to the genera of the Opiinae, but the number of genera and the limits of several genera is still a matter of discussion.

Chen and Weng (2005) published an overview of the Opiinae from China, but no Opiinae were listed for the province of Hunan of the total of 121 species known from China. Also in the review of the Hymenoptera of Hunan (You and Wei 2006) no Opiinae were listed for Hunan. Therefore, in 2009-2011 the first two authors made several short surveys to collect Opiinae; mainly in Badagong Mountains in northern Hunan. The collected species proved to be nearly all new to science, indicating the primordial status of our knowledge of Chinese Opiinae and the incompleteness of the review by Chen and Weng (2005) for China.

Opiinae are solitary koinobiont endoparasitoids of larvae of cyclorraphous Diptera, but oviposition may take place in the egg of the host (ovo-larval parasitoids). They may play an important role in the control of dipterous pests such as fruit-infesting Tephritidae and mining Agromyzidae. The parasitoid larva has it final development when the host larva has made its puparium and the adult parasitoid emerges from this puparium.

## Material and methods

Specimens of Opiinae from Hunan were collected and directly killed in 70% alcohol. A selection was made of presumably different species for molecular analyses. After DNA extraction (see below) the specimens were prepared after chemical treatment (AXA method; van Achterberg 2009 and van Achterberg et al. 2010). The sequences have been uploaded to GenBank; the accession numbers of the analysed specimens are listed in Table 1.

For identification of the subfamily Opiinae, see van Achterberg (1990, 1993, and 1997a), for identification of the genera, see Chen and Weng (2005), Wharton (1988, 1997), van Achterberg (1997b, 2004a) and the key below, for references to the Opiinae, see Yu et al. (2012) and for the terminology used in this paper, see van Achterberg (1988, 1993). Measurements are taken as indicated by van Achterberg (1988). Additional non-exclusive characters in the key are bracketed. For additional taxonomic information the reader is referred to the Taxapad interactive catalogue (Yu et al. 2012). The synonomies proposed in this paper are by the second author.

**Table 1.** List of DNA vouchers and GenBank Accession numbers.

RMNH INS. No.	Species	Locality	GenBank Accession Numbers		
			16S	28S	COI
CVA04235	*Apodesmia melliclypealis* sp. n.	China: Hunan: Badagong Mts: Sangzhi	JQ736245	JQ736272	JQ736301
CVA04236	*Phaedrotoma* sp. (specimen lost)	China: Hunan: Badagong Mts: Sangzhi	JQ736246	JQ736273	JQ736302
CVA04237	*Phaedrotoma rugulifera* sp. n.	China: Hunan: Badagong Mts: Sangzhi	JQ736247	JQ736274	JQ736303
CVA04238	*Xynobius* sp. (specimen lost)	China: Hunan: Badagong Mts: Sangzhi	JQ736248	JQ736275	JQ736304
CVA04239	*Phaedrotoma depressa* nom. n.	China: Hunan: Badagong Mts: Sangzhi	JQ736249	JQ736276	JQ736305
CVA04240	*Xynobius notauliferus* sp. n.	China: Hunan: Badagong Mts: Sangzhi	JQ736250	JQ736277	JQ736306
CVA04241	*Rhogadopsis obliqua* sp. n.	China: Hunan: Badagong Mts: Sangzhi	JQ736251	JQ736278	JQ736307
CVA04242	*Opius monilipalpis* sp. n.	China: Hunan: Badagong Mts: Sangzhi	JQ736252	JQ736279	JQ736308
CVA04243	*Opius youi* sp. n.	China: Hunan: Badagong Mts: Sangzhi	JQ736253	JQ736280	JQ736309
CVA04244	*Coleopioides diversinotum* sp. n.	China: Hunan: Badagong Mts: Sangzhi	-	JQ736281	JQ736310
CVA04245	*Psyttoma latilabris* (Chen & Weng)	China: Shandong: Anqiu: Wenquan	JQ736254	JQ736282	-
CVA04246	*Fopius dorsopiferus* sp. n.	China: Hunan: Nan Mts: Chengbu	JQ736255	JQ736283	JQ736311
CVA04247	*Opius monilipalpis* sp. n.	China: Hunan: Nan Mts: Chengbu	JQ736256	JQ736284	JQ736312
CVA04248	*Phaedrotoma rugulifera* sp. n.	China: Hunan: Badagong Mts: Sangzhi	JQ736257	JQ736285	JQ736313
CVA04249	*Areotetes carinuliferus* sp. n.	China: Hunan: Badagong Mts: Sangzhi	JQ736258	JQ736286	JQ736314
CVA04250	*Phaedrotoma vermiculifera* sp. n.	China: Hunan: Badagong Mts: Sangzhi	JQ736259	JQ736287	JQ736315
CVA04251	*Opius pachymerus* sp. n.	China: Hunan: Nan Mts: Chengbu	JQ736260	JQ736288	JQ736316
CVA04252	*Opius crenuliferus* sp. n.	China: Hunan: Badagong Mts: Sangzhi	JQ736261	JQ736289	JQ736317
CVA04254	*Phaedrotoma protuberator* sp. n.	China: Chengbu/Hunan: Nan Mts	JQ736263	JQ736291	JQ736319
CVA04255	*Coleopioides postpectalis* sp. n.	China: Anqiu/Shandong: Wenquan	-	JQ736292	JQ736320
CVA04256	*Phaedrotoma semiplanata* sp. n.	China: Sangzhi/Hunan: Badagong Mts	JQ736264	JQ736293	JQ736321
CVA04257	*Apodesmia incisula* (Fischer)	Netherlands: Waarder	JQ736265	JQ736294	JQ736322
CVA04258	*Utetes testaceus*	Netherlands: Castricum	-	-	JQ736323
CVA04259	*Neopius rudis* (Wesmael)	Netherlands: Waarder	JQ736266	JQ736295	JQ736324
CVA04261	*Opiognathus ocreatus* (Papp) comb. n.	Slovakia: Predna Hora	JQ736268	JQ736297	JQ736326
CVA04262	*Opius pallipes* Wesmael	Netherlands: Waarder	JQ736269	JQ736298	JQ736327
CVA04263	*Biosteres carbonarius* (Nees)	Slovakia: Predna Hora	JQ736270	JQ736299	JQ736328
CVA04264	*Opiognathus pactus* (Haliday)	Netherlands: Waarder	JQ736271	JQ736300	JQ736329
Outgroup	*Colastes braconius* Haliday	United Kingdom: England: nr Bristol	sequence from S. Yaakop	HQ416430	sequence from S. Yaakop

### Abbreviations for depositories

**FAFU** Institute of Beneficial Insects, Plant Protection College, Fujian Agriculture and Forestry University, Fuzhou, China

**RMNH** Naturalis Biodiversity Center, Leiden, the Netherlands

**ZUH** Institute of Insect Sciences, Zhejiang University, Hangzhou, China.

### Molecular analyses

*DNA extraction*. A total of 30 species were used for the molecular study. The analysed specimens and the Gen Bank accession numbers are listed in Table 1. The whole insect was used for DNA extraction, using a modified method from DNeasy Blood & Tissue Kit (Qiagen, Valencia, California, U.S.A.); the modification is referred to as the ‘freezing method’ by Yaakop et al. (2009). The first to third steps from the original protocol were modified. According to the original protocol, samples should be cut into small pieces and added to 180 µl of buffer ATL + 20 µl of proteinase K, then the sample has to be incubated at 55°C. However, with the freezing method, the sample is soaked with 180 µl of buffer ATL + 20 µl of proteinase K without destroying the sample, followed by 10 minutes incubation at 55°C and then kept in a freezer at -22°C overnight. After that the general protocol was used for the remaining steps. The advantage from this modified method is that samples (vouchers) used in this molecular study still exist. If necessary, the specimen can be re-examined.

*Selection of molecular markers*. Three molecular markers were selected based on earlier successful use in entomology: n28S rDNA, D2 region, mt16S rDNA and COI, a mitochondrial protein coding marker. Gimeno et al. (1997) and Yaakop et al. (2009) showed that 28S and 16S were informative for resolving the relationships of the subfamily Opiinae. Belshaw et al. (1998) used the 28S for resolving the phylogeny of the Ichneumonidae. Shi et al. (2005) used 28S, 18S and 16S and morphological data to recover relationships in the family Braconidae, but Pitz et al. (2007) could not reproduce the results. Kambhampati et al. (2000) used 16S to resolve the relationships among genera of the subfamily Aphidiinae and Dowton et al. (1998) for the family Braconidae in general. COI was selected because it successfully resolved the phylogeny of the cyclostome subfamilies in Braconidae in combination with 28S and morphological datasets (Zaldívar-Riverón et al. 2006).

*Polymerase Chain Reaction (PCR)*. PCR reaction volume was 25µl containing 18.75µl H2O, 2.5µl 10× buffer, 1µl of 10 pmol of each primer, 0.5µl of 0.2mM DNTPs,0.25µl of 5 U of Taq DNA polymerase and 1µl specimen DNA. The primers used for all amplifications are presented in Table 2. The PCR amplification was conducted with Thermocycler Perkin Elmer 2400. Amplification conditions were: (i) COI: 45cycles of 15s at 94º, 30s at 45º, and 5min at 72º following the initial denaturation at 94º for 3min; (ii) 16SrDNA: 35cycles of 15s at 94º, 30s at 48º, and 7min at 72º following the initial denaturation at 94º for 3min; (iii) 28SrDNAD2: 45cycles of 15s at 94º, 30s at 55º, and 8min at 72º following the initial denaturation at 94º for 3min.

**Table 2.** List of primers sequences used in this study.

**Gene**	**Primer**	**Sequences(5’- 3’)**	**Reference**
**COI**	Forward-L1490	GGTCAA CAA ATC ATA AAG ATA TTG G	Folmer et al. 1994
	Reverse-H2198	TAA ACT TCA GGG TGA CCA AAA AAT CA	Folmer et al. 1994
**16S**	Forward-16Swb	CAC CTG TTT ATC AAA AAC AT	Dowton and Austin 1998
	Reverse-16Souter	CTT ATT CAA ATC GAG GTC	Whitfield 1997
**28S**	Forward-28S3665	AGA GAG AGT TCA AGA GTA CGT G	Belshaw and Quicke 1997
	Reverse-28S4047	TTG GTC CGT GTT TCA AGA CGG G	Campbell et al. 1993

*Sequence and data alignment*.After electrophoresis, an amplified fragment was purified by Wizard Genomic DNA Purification Kit (PROMEGA). Sequences obtained were edited using Sequencher 4.2 and were aligned using Clustal X 1.8 (Thompson et al. 1997). Alignments were manually adjusted to minimize informative sites and ambiguously aligned regions were defined as character sets for possible exclusion using MacClade 4.08. DNA sequences were compared with sequences of the GenBank database using the BLAST algorithm to verify the amplification of the correct gene. The resulting sequences were deposited in GenBank (accession numbers in Table 1)

### Phylogenetic analysis

After alignment, phylogenetic analyses were performed by neighbor-joining (NJ) and maximum parsimony (MP) methods using PAUP*4.0b10 (Swofford 2002) and Bayesian Inference using MrBayes v3.2. The NJ method for obtaining a minimum-evolution tree with a bootstrap test was performed using the Clustal W progrom. The bootstrap values were based on 1,000 replicates. MP analyses were performed with gaps treated as missing data, heuristic parsimony search (Hillis et al. 1996) was performed using 100 replicates of random addition sequences and including the TBR (tree bisection reconnection) option for branch swapping. Statistical support was obtained by bootstrap analysis with 1000 replications (Felsenstein 1985). For the Bayesian analysis the same general models as identified by Modeltest were used. Analyses were initiated with random starting trees and run for 1.0 × 10^6^ generations sampling the four Markov chains every 2500 generations resulting in 1000 sampled trees. *Colastes braconius* Haliday is used as an outgroup because it belongs to the subfamily Exothecinae which is probably the sistergroup of the Opiinae + Alysiinae (Gimeno et al. 1997) or even the sister of Opiinae in the Opiinae + Alysiinae clade with ≥ 0.95 posterior probability (Sharanowski et al. 2011); it has also a high morphological resemblance to the Opiinae. According to Gimeno et al. (1997) the bootstrap values for (Exothecinae (Opiinae + Alysiinae)) are 79-100 % and the genus *Gnamptodon* (Gnamptodontinae) is found to be more distantly related.

## Results and discussion

Only nearly half of the new species could be sequenced because part of the material was too old and because of lack of time. Some European species were added of which *Opius pactus* Haliday is the type species of the subgenus *Opiognathus* Fischer, *Opius testaceus* Wesmael of the genus *Utetes* Foerster, *Opius pallipes* Wesmael of the genus *Opius* Wesmael, *Opius carbonarius* Nees of the genus *Biosteres* Foerster and *Opius rudis* Wesmael is the oldest name for the type species of the subgenus *Neopius* Gahan. The latest general keys to the genera of Opiinae are given by Fischer (1987) and Wharton (1997) and need updating; molecular data have not been used so far but should play a role here. Nevertheless, DNA sequences can never replace a sound morphological approach; it is an additional tool to check the outcome of the morphological analysis. Gimeno et al. (1997), Dowton et al. (1998, 2002) and Shi et al. (2005) included a few genera in their molecular studies but no larger surveys have been made up to now. GenBank contains only few taxa of Opiinae (considering the size of the subfamily) and possibly some of them are misidentified. Pitz et al. (2007) showed by using GenBank sequences of 16S, 18S and 28S of four genera (*Biosteres*, *Xynobius*, *Opius* and *Fopius*) that Opiinae are the sistergroup of Alysiinae and that *Biosteres* is the most basal genus of the four genera. The bootstrap percentages were below 60 within the Opiinae, indicating that the results are at best provisional. The results of the combined DNA analysis of 11-13 (sub)generic groups (Figs 10–12) show several interesting results which largely agree with still unpublished results of Dr S. Yaakop (Bangi, Malaysia) obtained from Malaysian Opiinae.

The parsimony results of the combined analyses of the three molecular markers (Figs 10–11) did not result in a well supported backbone of the cladogram; the confidence levels for the higher levels are acceptable. Bayesian analyses led to better supported results (Fig. 12) for most groups. Two species are always misplaced: *Opius youi* and *Phaedrotoma protuberator*, which may be the result of contamination and both species need to be re-sampled.

This is the first study of Opiinae that includes molecular data for 13 (sub)genera for phylogenetic analysis. Compared to the study by Gimeno et al. (1997), this study includes the following seven additional (sub)genera: *Coleopioides*, *Neopius*, *Apodesmia*, *Opiognathus*, *Rhogadopsis*, *Areotetes* and *Psyttoma*. Due to the limited number of analysed taxa, the results of Gimeno et al. (1997) are difficult to compare with our study. Our results partly corroborate Wharton’s (1988) view on the basal lineages of the Opiinae. Considering its morphology *Biosteres* is supposed to be one of the basal lineages of the Opiinae which we found in the combined Bayesian analysis (Fig. 12), but was the sistergroup of *Opius* s.s. in parsimony analysis (Figs 10–11). The group with the inner side of the hind tibia with a fine carinula baso-laterally (formerly the genus *Utetes* Foerster) is split in this paper because one group (here named *Areotetes* gen. n.) always came out distantly related to the other groups (*Utetes* s.s. and *Opiognathus*). The latter is not closely related to *Utetes* s.s. either (Fig. 3) and, therefore, this subgenus of the genus *Opius* is treated as a valid genus in this paper. The three groups are also morphologically different (see key below).

**Figure 1.**
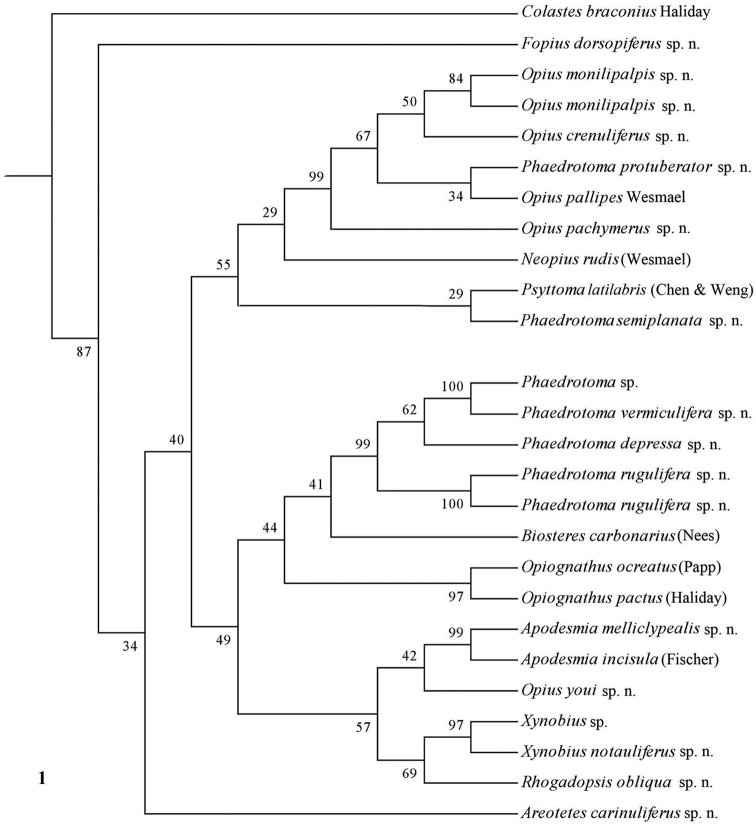
Phylogeny of the Opiinae based on 16S using NJ method (50% consensus tree). Numbers at nodes are bootstrap values (%).

**Figure 2.**
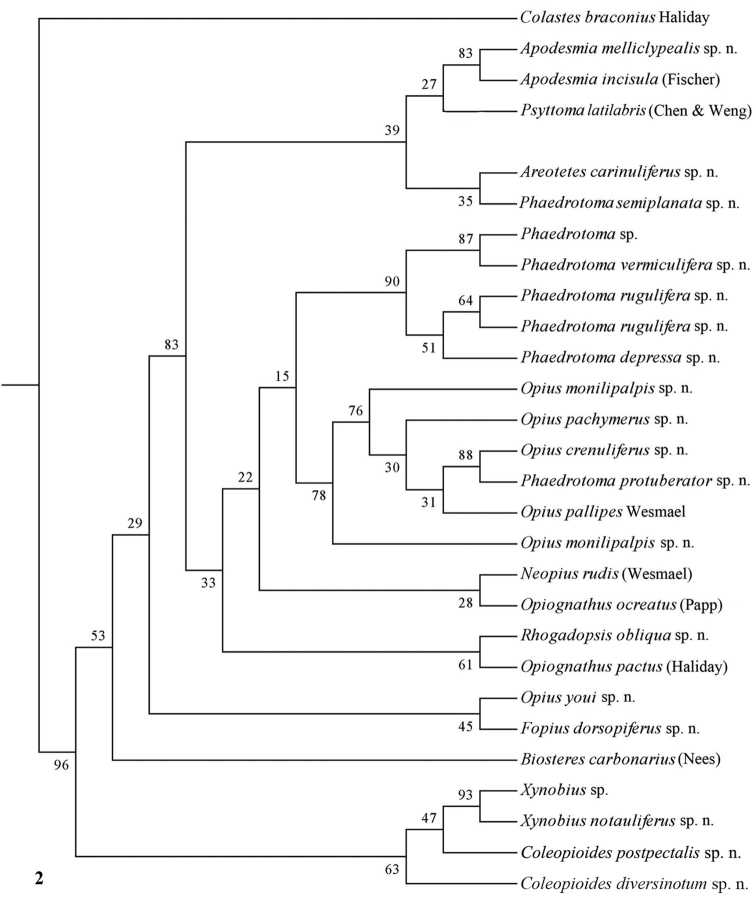
Phylogeny of the Opiinae based on 28S using NJ method (50% consensus tree). Numbers at nodes are bootstrap values (%).

**Figure 3.**
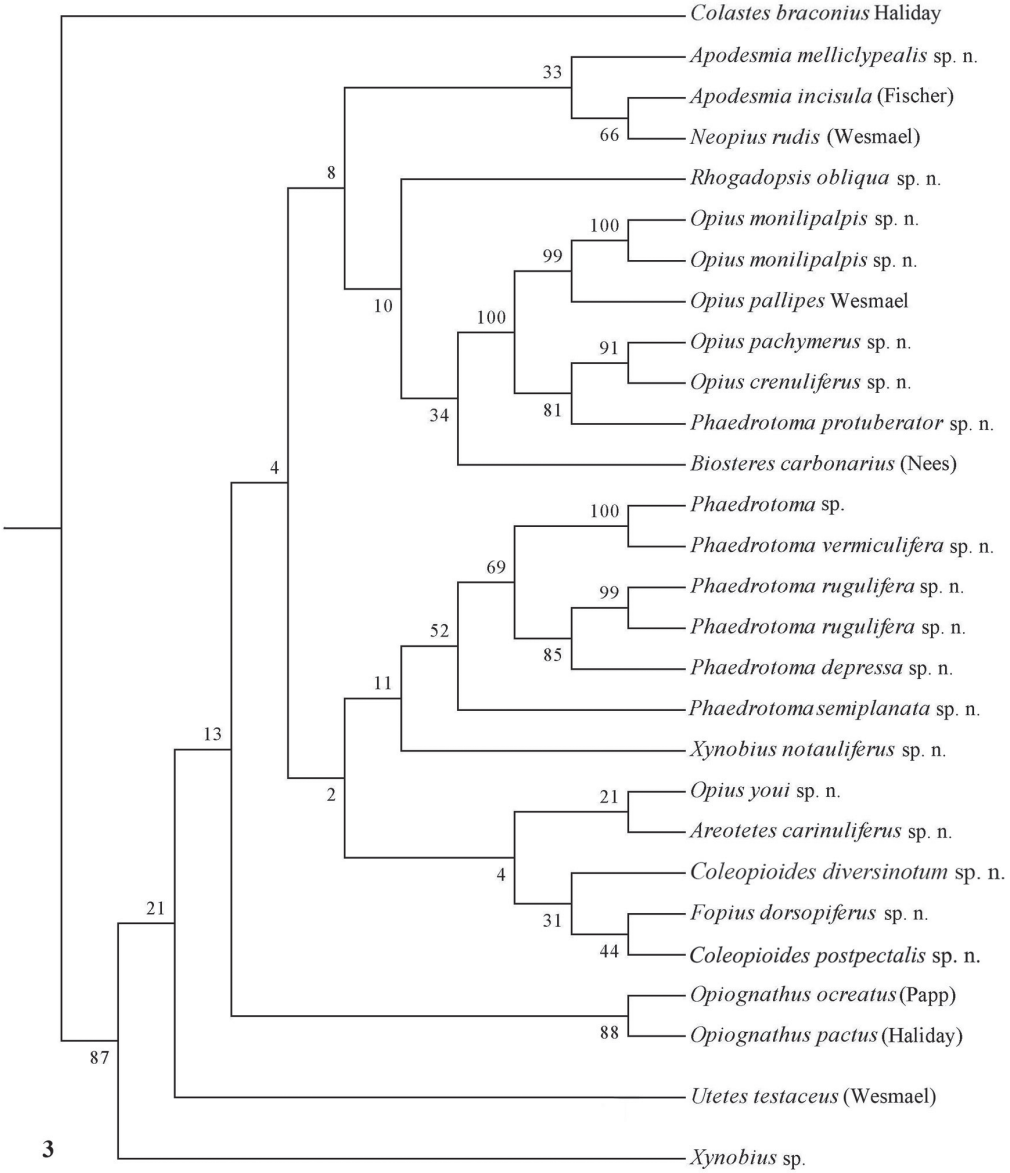
Phylogeny of the Opiinae based on COI using NJ method (50% consensus tree). Numbers at nodes are bootstrap values (%).

**Figure 4.**
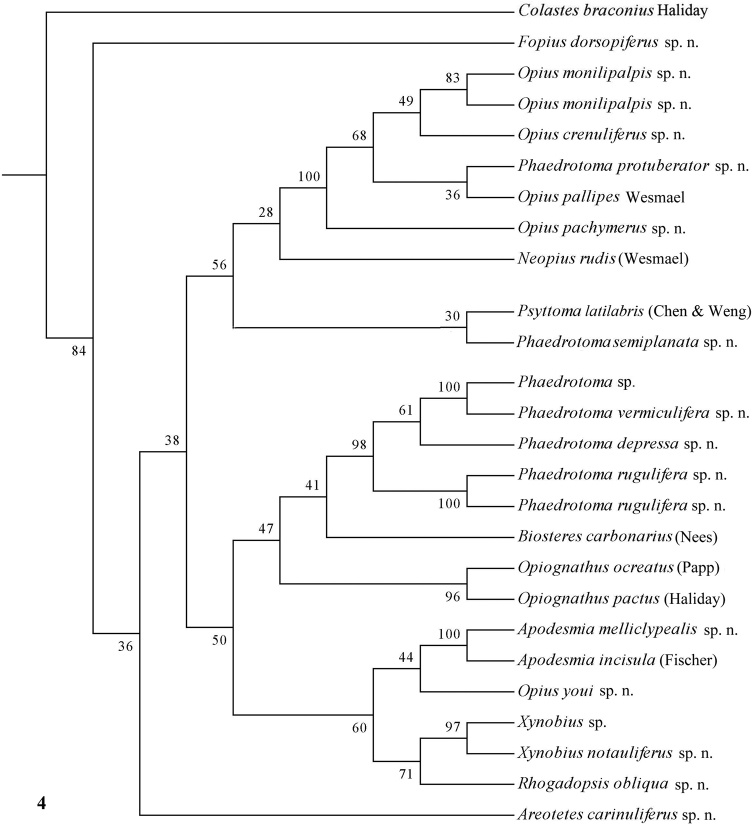
Phylogeny of the Opiinae based on 16S using MP method (50% consensus tree). Numbers at nodes are bootstrap values (%).

**Figure 5.**
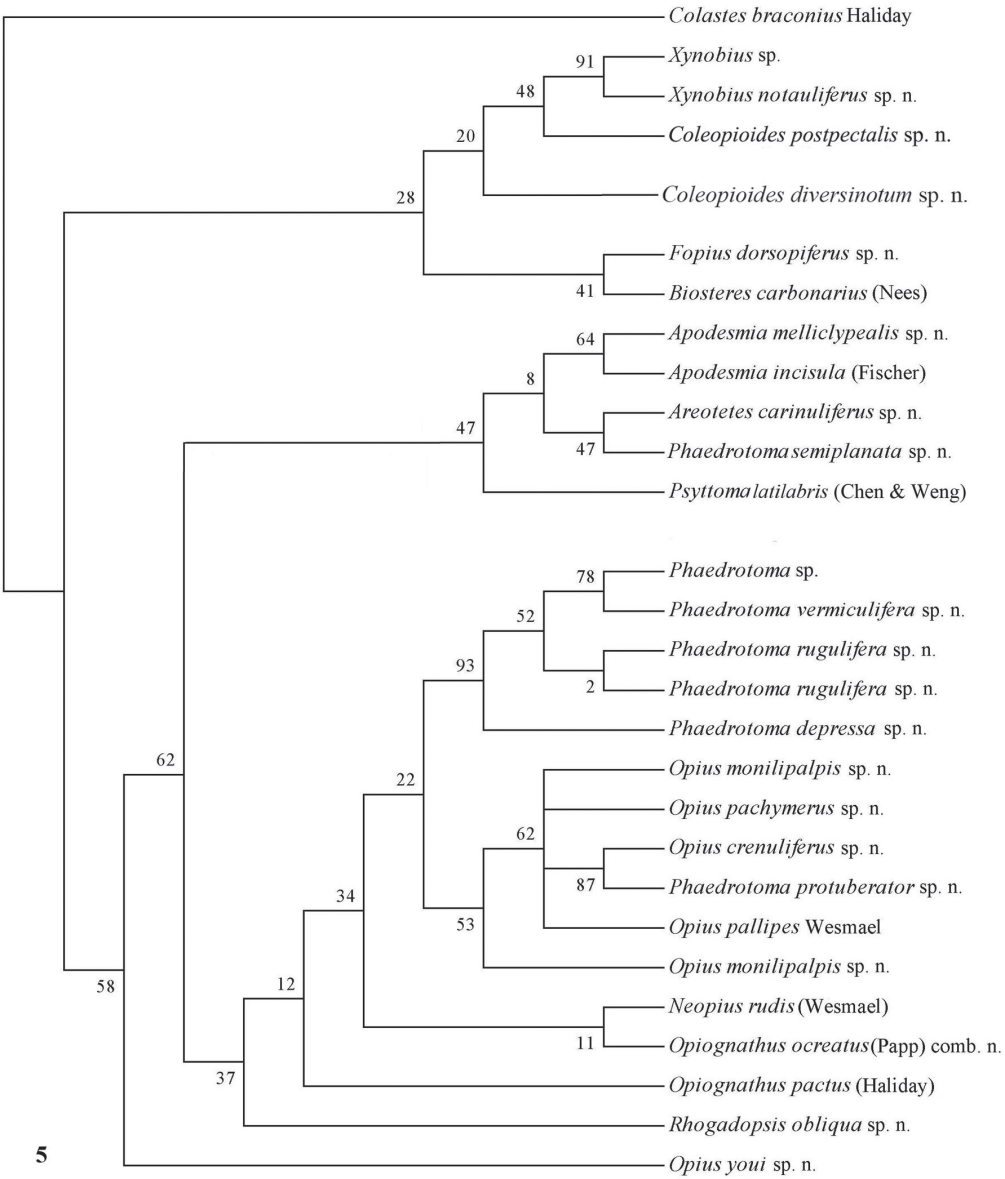
Phylogeny of the Opiinae based on 28S using MP method (50% consensus tree). Numbers at nodes are bootstrap values (%).

**Figure 6.**
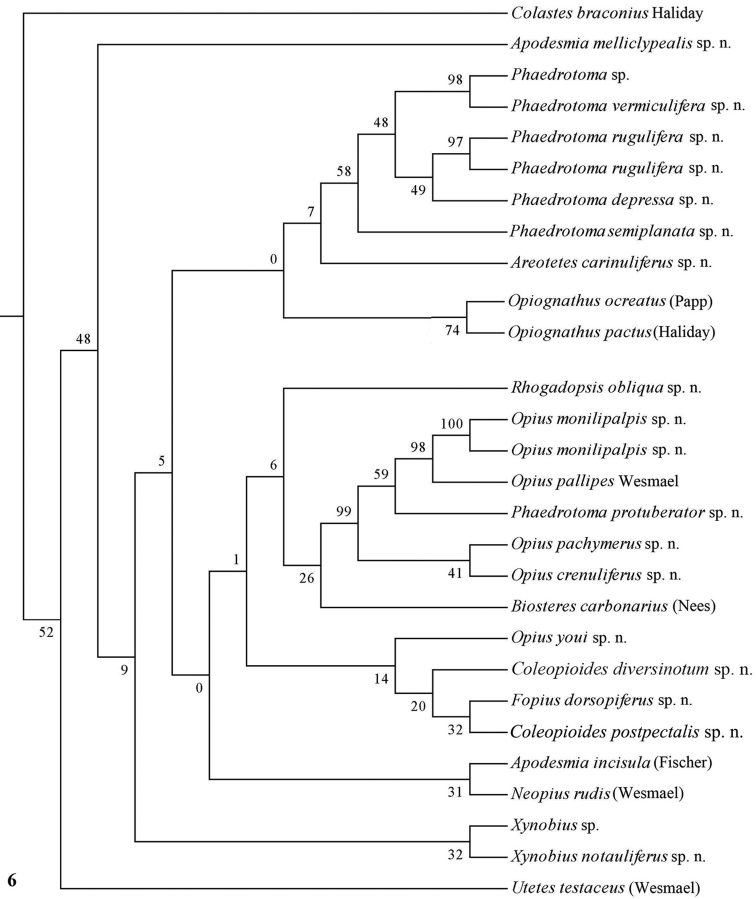
Phylogeny of the Opiinae based on COI using MP method (50% consensus tree). Numbers at nodes are bootstrap values (%).

**Figure 7.**
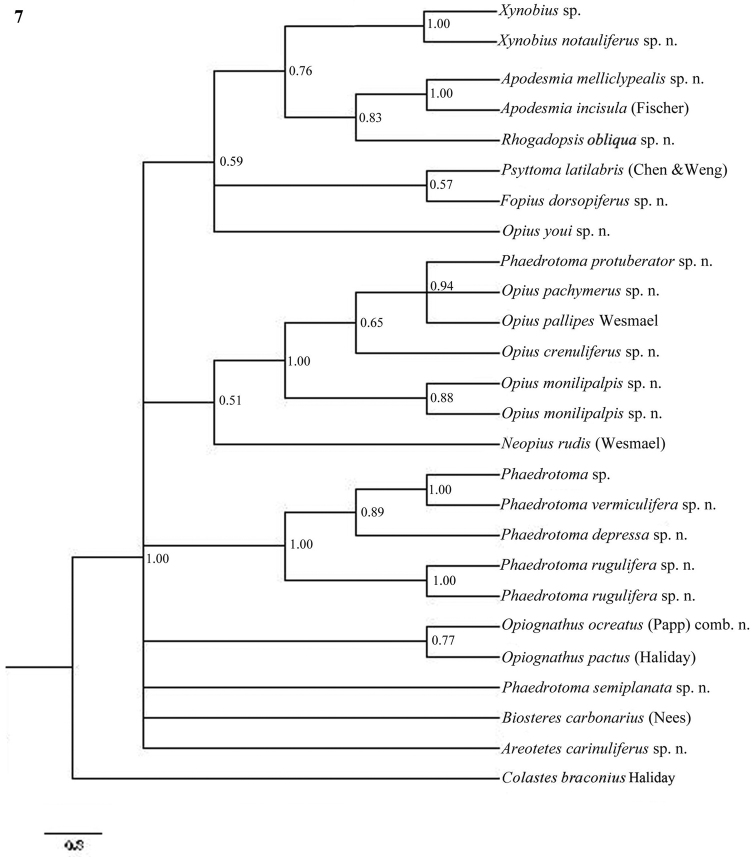
Phylogeny of the Opiinae based on 16S using Bayesian method. Numbers at nodes are Bayesian posterior probabilities.

**Figure 8.**
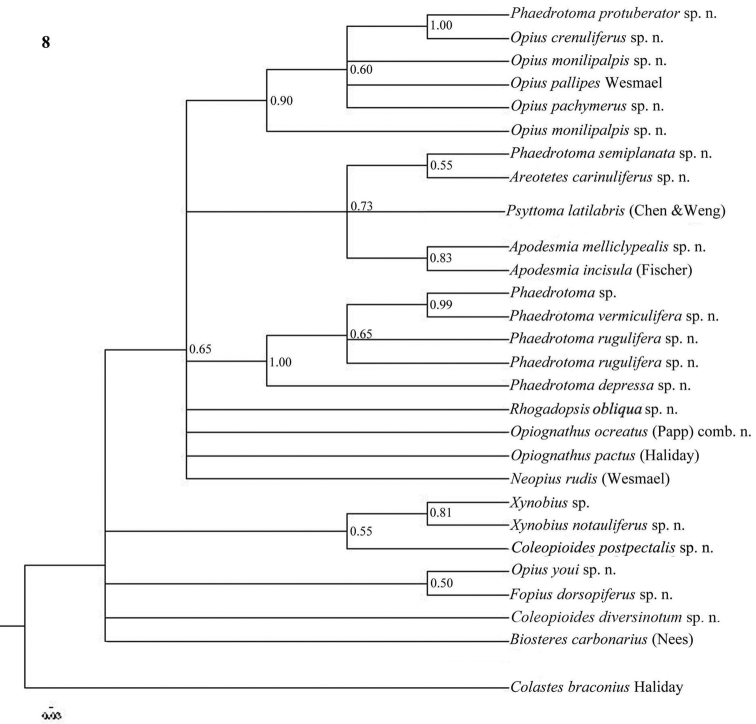
Phylogeny of the Opiinae based on 28S using Bayesian method. Numbers at nodes are Bayesian posterior probabilities.

**Figure 9.**
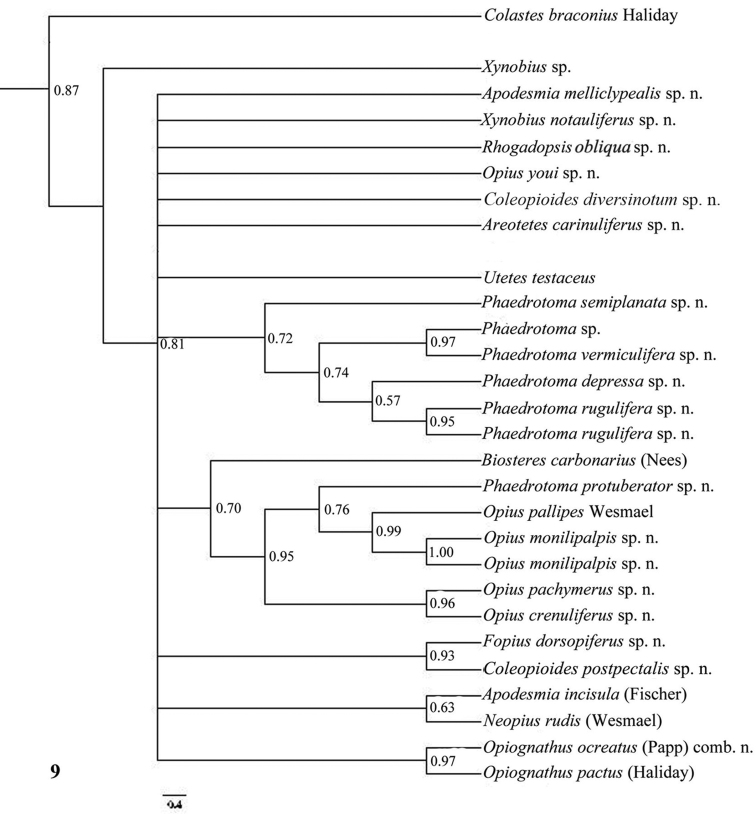
Phylogeny of the Opiinae based on COI using Bayesian method. Numbers at nodes are Bayesian posterior probabilities.

**Figure 10.**
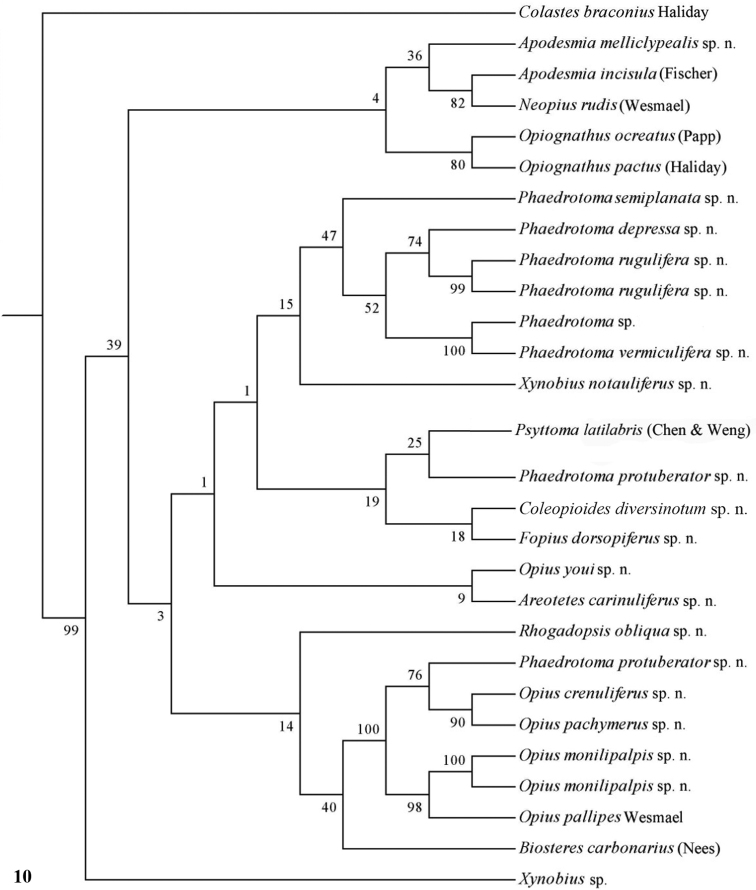
Phylogeny of the Opiinae based on 16S, 28S and COI using NJ method (50% consensus tree). Numbers at nodes are bootstrap values (%).

**Figure 11.**
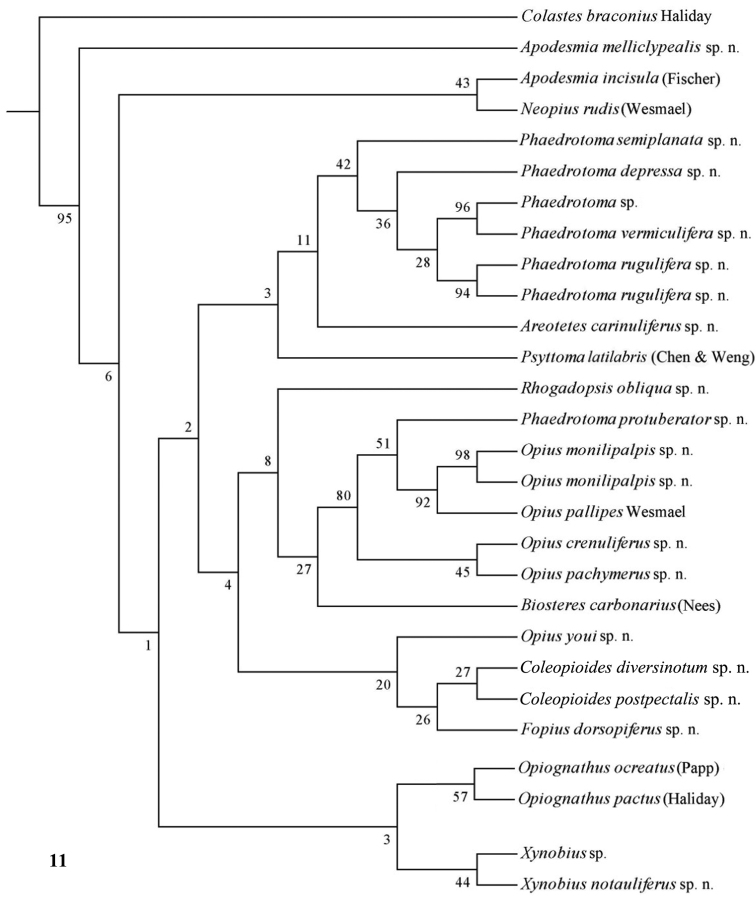
Phylogeny of the Opiinae based on 16S, 28S and COI using MP method (50% consensus tree). Numbers at nodes are bootstrap values (%).

**Figure 12.**
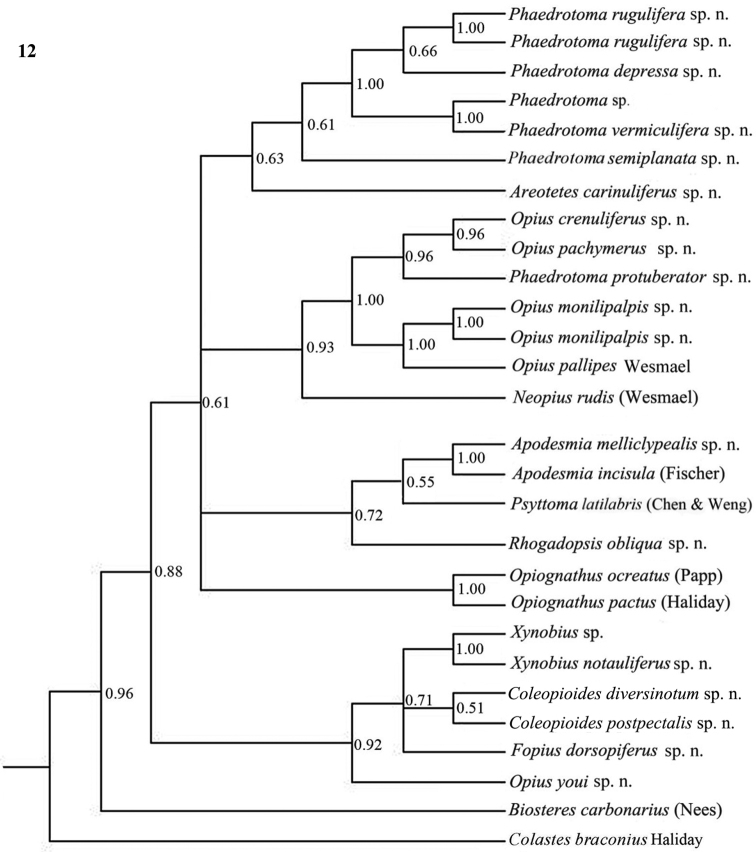
Phylogeny of the Opiinae based on 16S, 28S and COI using Bayesian method. Numbers at nodes are Bayesian posterior probabilities.

### Individual molecular markers

Each individual dataset of a single gene resulted in a less resolved phylogeny of the Opiinae (Figs 1–9). Generally, the genus *Phaedrotoma* (formerly included in the genus *Opius*) is well separated from the genus *Opius* s.s. With 16S *Fopius* and *Areotetes* come out as basal groups if NJ and MP are used, but with Bayesian analysis *Fopius* groups with *Psyttoma* and is much less basal. With 28S *Xynobius* + *Coleopioides* and *Fopius* + *Biosteres* are the basal groups in NJ and parsimony analyses, but with Bayesian results *Xynobius* and *Coleopioides* group together and there are no distinct basal lineages. With COI and parsimony *Utetes*, *Lissosema*, *Apodesmia* and *Xynobius* are the basal groups (and with NJ also *Opiognathus*), but there are no distinct basal lineages in the Bayesian results. Only 28S gives basal groups having morphological character states corroborating a basal position (e.g., the included species of *Xynobius*, *Fopius* and *Biosteres* all have a dorsope on the first metasomal tergite). *Coleopioides* is, according to 28S, a derived group related to *Fopius* (grouping also recovered with COI, Fig. 9); having an elongate second submarginal cell of the fore wing. The type species of *Coleopioides* shares with the type species of *Fopius* the presence of a postpectal carina.

### Combining data partitions

In this study, by combining datasets it was possible to obtain a better resolved phylogeny of the Opiinae (Figs 10–12). The results do not support the hypothesis that the Opiinae comprises two tribes: Opiini and Biosterini as suggested by Shi et al. (2005). In our study, the genus *Biosteres* has a variable position, compared to the *Xynobius*-group or the Opiini. Traditionally, the genus *Biosteres* is characterized by having a short second submarginal cell of the fore wing (Fischer 1972b). This character state is not very helpful because it is also present in the genus *Fopius* and several other genera of the Opiini (as defined by exclusion of the Biosterini). *Biosteres* needs, therefore, as pointed out by Wharton (1988), to be defined by a combination of characters. The *Xynobius*-group (consisting of *Fopius*, *Coleopioides* and *Xynobius*) is separated from *Biosteres* and Opiini in the Bayesian analysis (Fig. 12). However, no apomorphic character states were found to group these genera.

The short second submarginal cell of the fore wing is an apomorphy for the genera *Fopius* and *Utetes*, while the presence of the dorsope in the genus *Xynobius* is considered to be a plesiomorphy. The dorsope of the first tergite is absent in the Opiini. *Xynobius* contains parasitoids of leaf-mining larvae of Anthomyiidae and Scathophagidae as in *Biosteres* (Wharton 1997) and both have a dorsope. Wharton (1988, 1997) abandoned the use of tribes and subtribes in the Opiinae, using informal genus groups, and considering our results this seems still the best solution.

#### Subfamily Opiinae

Blanchard, 1845

Opiites Blanchard, 1845: 157.
Opioidae Foerster, 1862: 258.
Baeocentrini Fahringer, 1928: 8.
Ademonini Fischer, 1964: 207.
Biosterina Fischer, 1970: 85.
Coleopiina Fischer, 1970: 85.
Pokomandyina Fischer, 1970: 87.
Desmiostomatini Fischer, 1972b: 59.
Bitomina Fischer, 1982: 29 (unavailable name).

##### Diagnosis.

Prepectal carina absent (but partly developed in *Ademon*); posterior flange of propleuron present; maxillary palp with 6 segments and labial palp with 4 segments; hypoclypeal depression present or absent, if present then shallow, medio-ventral rim of clypeus near level of upper condyli of mandible; ventral part of clypeus not part of the hypoclypeal depression; labrum flat or weakly concave and glabrous, rarely distinctly concave; occipital carina usually present laterally, but nearly always widely interrupted medio-dorsally; notauli often largely absent on mesoscutal disc, often with a medio-posterior depression (or “mid-pit”, but sometimes groove-like); pronope absent to very large, round or slit-like; vein M+CU1 of fore wing largely unsclerotized (only pigmented and not tubular); if completely tubular and fully sclerotized then laterope of first metasomal tergite distinct; fore tibia without row of pegs or spines, but sometimes bristly setose; dorsope of first metasomal tergite absent or present; first tergite with or without convex lateral parts and movably connected to second tergite; second tergite without transverse elevated area and usually with pair of oblique depressions basally and similarly or more strongly sclerotized than its epipleuron; ovipositor usually short (hardly or not protruding beyond apex of metasoma) in parasitoids of mining larvae and well protruding in parasitoids of larvae in fruits.

##### Biology.

Koinobiont endoparasitoids of Diptera-Cyclorrhapha: Agromyzidae (including *Phytobia* species in woody branches), Anthomyiidae (*Pegomya* species), Drosophilidae, Psilidae (*Chyliza* species in galls), Lonchaeidae, Ephydridae (*Hydrellia* species in waterplants), Scathophagidae (*Chylizosoma* and *Cleigastra* species) and Tephritidae. Some species are used for biocontrol, e.g. *Psyttalia* and *Utetes* species against *Ceratitis capitata* Wiedemann and *Opius pallipes* Wesmael against leaf miners in greenhouses, but several others have a high potential for biocontrol.

##### Distribution.

Cosmopolitan; frequently collected.

##### Key to genera and species of the subfamily Opiinae from Hunan

**Table:** 

1	Marginal cell of fore wing open apically (Fig. 96); veins m-cu and r-m of fore wing absent (Fig. 96); occipital carina largely absent (Fig. 97); genus *Indiopius* Fischer, 1966	*Indiopius chenae* sp. n.
–	Marginal cell of fore wing closed apically (Figs 14, 146, 364); veins m-cu and r-m of fore wing present (Figs 14, 146); occipital carina present laterally, at least behind lower half of eyes (Figs 20, 147, 149)	2
2	Second metasomal tergite distinctly (1.3–2.1 times) longer than third tergite and bordered posteriorly by a crenulate second metasomal suture (Fig. 421); third tergite with sharp lateral crease (Fig. 417); genus *Orientopius*Fischer, 1966	*Orientopius punctatus* van Achterberg & Li, 2012
–	Second tergite about as long as third tergite (Figs 16, 32, 76) and if longer then without a crenulate second metasomal suture posteriorly (cf. Fig. 76); third tergite without sharp lateral crease (Figs 73, 105)	3
3	Inner side of hind tibia with a more or less oblique fine carinula baso-laterally (Figs 39, 47, 59, 123, 378); propodeum often with medio-longitudinal carina anteriorly (Figs 35, 44, 55, 56, 383); [vein 3-SR of fore wing slightly curved; malar suture absent; pronotum short and vertical; medio-posterior depression of mesoscutum absent or small; precoxal sulcus narrowly crenulate; clypeus obtuse ventrally]	4
–	Inner side of hind tibia without a carinula baso-laterally (Figs 186, 190); propodeum usually without medio-longitudinal carina (Figs 16, 199, 249)	9
4	Clypeus obtuse and slightly concave ventrally (Figs 38, 48, 58); posterior face of propodeum with areolation (Figs 44, 56); vein m-cu of fore wing slightly postfurcal (Figs 33, 43, 53); [pronope round or obsolescent, if slit-shaped then indistinctly developed; precoxal sulcus narrowly crenulate; epicnemial area smooth; medio-longitudinal carina of propodeum medium-sized]; *Areotetes* gen. n.	5
–	Clypeus thin and protruding or truncate ventrally (Figs 111, 122, 379); posterior face of propodeum without distinct areolation (Figs 108, 118, 377); vein m-cu of fore wing usually distinctly postfurcal (Figs 106, 116), sometimes nearly interstitial (Fig. 375)	7
5	Mesoscutum with a small medio-posterior depression (Figs 34, 55); second metasomal tergite distinctly costate striate medially (Figs 35, 56); apical third of antenna of female partly pale yellowish (Figs 36, 61); face yellowish-brown or pale yellowish (Figs 38, 58)	6
–	Medio-posterior depression of mesoscutum absent (Fig. 44); second tergite smooth (Fig. 45); antenna of female subapically (Fig. 46) and face dark brown or blackish (Fig. 48); [first metasomal tergite at least partly smooth and shiny (Fig. 45)]	*Areotetes carinuliferus* sp. n.
6	Vein 3-SR of fore wing about 2.5 times as long as vein 2-SR and weakly curved (Fig. 53); head dorsally (except orbita) dark brown; apical third of antenna of male dark brown; pterostigma comparatively narrow and longer (Fig. 53); [apical antennal segments of female brown (Fig. 61)]	*Areotetes striatiferus* sp. n.
–	Vein 3-SR of fore wing about 1.7 times as long as vein 2-SR and nearly straight (Fig. 33); head dorsally (except stemmaticum and its surroundings) yellow; apical third of antenna of male pale yellowish (Fig. 36); pterostigma comparatively wide and short (Fig. 33)	*Areotetes albiferus* sp. n.
7	Mandible abruptly narrowed submedially and more or less widened basally (Figs 112, 124); pronope round (Fig. 121) or slit-shaped (Fig. 107); precoxal sulcus narrowly crenulate (Fig. 105); epicnemial area smooth (Fig. 105); [carinula of hind tibia sinuate (Figs 113, 123); genus *Opiognathus* Fischer, 1972	8
–	Mandible triangular, gradually narrowed apically (Figs 379, 380); pronope slit-shaped and deep (Fig. 381); precoxal sulcus widely crenulate; epicnemial area more or less crenulate; genus *Utetes* Foerster, 1862 *sensu stricto*; [vein m-cu of fore wing subinterstitial (Fig. 375); medio-longitudinal carina of propodeum complete (Fig. 377); ventral margin of clypeus truncate (Fig. 379); wing membrane subhyaline] ; genus *Utetes* Foerster, 1862	*Utetes longicarinatus* sp. n.
8	Mandible gradually widened basally (Fig. 112); hypoclypeal depression present (Fig. 111); medio-posterior depression of mesoscutum deep (Fig. 107); first metasomal tergite sculptured and less shiny (Fig. 108); propodeum coarsely reticulate and without smooth areas (Fig. 108)	*Opiognathus aulaciferus* sp. n.
–	Mandible abruptly widened basally (Figs 122, 124); hypoclypeal depression absent (Fig. 122); medio-posterior depression of mesoscutum absent (Fig. 117); first tergite largely smooth and shiny (Fig. 118); propodeum large smooth (Fig. 118)	*Opiognathus brevibasalis* sp. n.
9	Vein 3-SR of fore wing shorter than vein 2-SR (Figs 85, 86); if up to 1.3 times longer than vein 2-SR then dorsope present (cf. Fig. 88); vein m-cu of hind wing present at least as a distinctly pigmented trace (Fig. 86); length of fore wing usually more than 3 mm	10
–	Vein 3-SR of fore wing distinctly longer than vein 2-SR (Figs 14, 64, 146); if 1.3-1.4 times longer (Figs 296, 326) then dorsope absent (Figs 298, 328); vein m-cu of hind wing usually largely or completely absent (Figs 64, 74, 248, 326); length of fore wing usually less than 3 mm	12
10	Propleuron with short subapical oblique carina (Fig. 94); medio-ventrally postpectal carina coarsely developed (Fig. 94); frons coarsely sculptured (Fig. 92); [mandible normal basally (Fig. 93)]; genus *Fopius* Wharton, 1987	*Fopius dorsopiferus* sp. n.
–	Propleuron without a short subapical oblique carina; medio-ventrally postpectal carina absent; frons smooth	11
11	Dorsope present; second metasomal tergite smooth; mandible somewhat widened basally in addition to a short ventral carina; [vein 3-SR of fore wing 2.0-2.7 times as long as vein r; apical half of scutellum finely densely rugulose (but more smooth in male from Hunan); hind coxa yellowish basally]; genus *Biosteres* Foerster, 1862	*Biosteres pavititus* Chen & Weng, 2005
–	Dorsope absent; second tergite striate; mandible normal basally; genus *Diachasmimorpha* Viereck, 1913	*Diachasmimorpha longicaudata* (Ashmead, 1905)
12	Dorsope present (Fig. 389)	13
–	Dorsope absent (Figs 16, 66, 129, 250, 267, 289)	15
13	Hypoclypeal depression present, large (Fig. 388) and medially ventral margin of clypeus (just) above upper level of condyli of mandibles (“subcyclostome condition”; Fig. 390); mandible normal ventrally (Fig. 390); notauli complete or nearly so (Fig. 386); genus *Xynobius* Foerster, 1862	14
–	Hypoclypeal depression absent or narrow, and medially ventral margin of cly-peus near upper level of condyli of mandibles (“mouth closed”); mandible widened ventro-basally; notauli largely absent or only posteriorly absent; [apical third of antenna of female dark brown or blackish; second tergite smooth]; genus *Opiostomus* Fischer, 1971	*Opiostomus aureliae* (Fischer, 1957) comb. n.
14	Notauli completely crenulate and comparatively wide (Fig. 386); wing membrane without dark patch (Fig. 385); middle lobe of mesoscutum moderately setose (Fig. 386); apical half of antenna of female with 3-8 pale yellowish segments (Fig. 387); second metasomal tergite longitudinally costate-striate (Fig. 389)	*Xynobius notauliferus* sp. n.
–	Notauli smooth and narrow; wing membrane with conspicuous dark patch; middle lobe of mesoscutum densely setose; apical third of antenna of female dark brown; second tergite smooth; [scutellum strongly convex]	*Xynobius maculipennis* (Enderlein, 1912) comb. n.
15	Propodeum with a transverse carina subbasally (Figs 65, 66, 76) and precoxal sulcus wide and crenulate (Fig. 73); second and third metasomal tergites enlarged, longer than following segments (Figs 63, 73); lateral margin of meso-scutum crenulate (Figs 63, 65, 73, 75, 83); scutellar sulcus wide and coarsely crenulate (Figs 65, 75, 83)	16
–	Propodeum without a transverse carina subbasally (Figs 16, 129, 217), ifpresent (Figs 328, 336, 354) then precoxal sulcus narrow and smooth or nearly so (Figs 334, 351); second and third tergites normal, about as long as following segments or shorter (Figs 13, 157, 167, 318, 403); lateral margin of mesoscutum smooth or nearly so (Figs 15, 128, 217, 227, 366); scutellar sulcus variable, usually medium-sized or narrow (Figs 24, 128)	18
16	Clypeus triangularly protruding ventrally; labrum concealed; frons, vertex and mesoscutum distinctly punctate; [genus not yet found in Hunan, but *Bitomus cheleutos* (Weng & Chen, 2005) comb. n. occurs in Fujian]	genus *Bitomus* Szépligeti, 1910
–	Clypeus truncate ventrally (Figs 68, 78); labrum more or less exposed (Fig. 78); frons, vertex and mesoscutum largely smooth (Figs 63, 65); genus *Coleopioides* gen. n.	17
17	Notauli largely absent on mesoscutal disc and notaulic area smooth (Figs 66, 83); postpectal carina present (Fig. 82); second metasomal tergite granulate (Fig. 76); propleuron rugulose subposteriorly; first discal cell of fore wing comparatively transverse (Fig. 74); tegulae dark brown; antenna of female with 24–26 segments; occipital carina coarsely crenulate (Fig. 84)	*Coleopioides postpectalis* sp. n.
–	Notauli nearly complete, narrow and finely crenulate (Fig. 65); postpectal carina absent; second tergite smooth (Fig. 66); propleuron smooth subposteriorly (Fig. 63); first discal cell of fore wing less transverse (Fig. 64); tegulae pale yellowish; antenna of female with 19 segments; occipital carina finely crenulate (Fig. 69)	*Coleopioides diversinotum* sp. n.
18	Occipital carina above mandibular base curved towards and meeting (just) hypostomal carina (Figs 20, 29); vein m-cu of fore wing subinterstitial (Figs 14, 23); genus *Apodesmia* Foerster, 1862	19
–	Occipital carina not or slightly curved ventrally and remain removed from hypostomal carina (Figs 132, 173, 203, 243, 292, 410); position of vein m-cu of fore wing variable, but rather frequently distinctly postfurcal (Figs 137, 146, 183, 248, 276, 287, 315, 325, 352, 364)	20
19	Medio-posterior depression of mesoscutum present (Fig. 24); vein m-cu of fore wing slightly antefurcal (Fig. 23); clypeus yellowish-brown ventrally (Fig. 27); mesosoma robust (Fig. 22); vein SR1 of fore wing evenly curved (Fig. 23)	*Apodesmia melliclypealis* sp. n.
–	Medio-posterior depression of mesoscutum absent (Fig. 15); vein m-cu of fore wing slightly postfurcal (Fig. 14); clypeus dark brown ventrally (Fig. 18); mesosoma slender (Fig. 13); vein SR1 of fore wing sinuate (Fig. 14)	*Apodesmia bruniclypealis* sp. n.
20	Apical half of mandible comparatively narrow and resulting in small teeth, mandible abruptly widened baso-ventrally (Figs 153, 155, 163, 172, 183, 193) and more or less tooth-like protruding basally and not only widened by a protruding carina; malar suture deep (Figs 182, 193); [medio-posterior depression of mesoscutum absent; mesoscutum strongly shiny (Fig. 188)]; genus *Opius* Wesmael, 1835 *sensu stricto*	21
–	Apical half of mandible comparatively wide and resulting in comparatively robust teeth, basally mandible not or gradually widened, at most with a carina ventrally (Figs 203, 221, 234, 253, 340, 370), without tooth-like protuberance and basally symmetrical or nearly so; if gradually widened ventro-basally (Figs 203, 212, 282) then malar suture reduced (Figs 203, 212, 282)	27
21	Hind femur robust (Fig. 161); third antennal segment of female about 3.4 times as long as wide (Fig. 165); area between malar suture and clypeus with some distinct punctures (Fig. 163)	*Opius pachymerus* sp. n.
–	Hind femur comparatively slender (Figs 130, 136, 140, 150, 171, 181, 190); third antennal segment of female 3.7–4.5 times as long as wide (Figs 134, 143, 145, 175, 191); area between malar suture and clypeus without distinct punctures or with irregular depressions (Figs 132, 142, 153, 193)	22
22	Basal cell of hind wing and second submarginal cell of fore wing comparatively wide (Fig. 137); malar suture short (Fig. 142); clypeus wide and ventral margin acute (Figs 141, 142); hypoclypeal depression deep (Fig. 141)	*Opius malarator* sp. n.
–	Basal cell of hind wing and second submarginal cell of fore wing comparatively narrow (Figs 127, 146, 178); malar suture longer (Figs 132, 173, 193) and sometimes partly absent or obsolescent; clypeus medium-sized (Figs 131, 192) and its ventral margin variable; hypoclypeal depression comparatively shallow (Figs 151, 182, 192)	23
23	Setose part of ovipositor sheath about 0.16 times as long as fore wing, half as long as hind tibia and 1.5 times as long as first tergite (Figs 186, 195); first tergite comparatively slender (Fig. 189)	*Opius zengi* sp. n.
–	Setose part of ovipositor sheath 0.05-0.06 times as long as fore wing, 0.2–0.3 times as long as hind tibia and about half as long as hind tibia and about half as long as first tergite (Figs 126, 133, 154, 167); first tergite comparatively robust (Figs 129, 148, 170, 180)	24
24.	Pronope deep and medium-sized (Fig. 176); pronotum yellowish-brown (Fig. 167); propodeum steep posteriorly (Fig. 167)	*Opius songi* sp. n.
–	Pronope absent or obsolescent (Figs 135, 152, 184); pronotum black, dark or chestnut brown (Figs 126, 145, 177); propodeum gradually lowered posteriorly (Figs 129, 145, 149, 177)	25
25	Clypeus flattened, comparatively large (Fig. 182) and its ventral margin slightly curved; first metasomal tergite yellowish as second tergite (Fig. 180)	*Opius youi* sp. n.
–	Clypeus convex and somewhat smaller (Figs 131, 151) and its ventral margin truncate; first tergite darker than second tergite (Figs 129, 148)	26
26	Oblique groove of pronotal side distinctly crenulate (Fig. 126); hind tibia comparatively wide (Fig. 130); vein m-cu of fore wing parallel to vein 1-M and slightly postfurcal (Fig. 127)	*Opius crenuliferus* sp. n.
–	Oblique groove of pronotal side largely smooth (Fig. 145); hind tibia narrow (Fig. 150); vein m-cu of fore wing converging to vein 1-M posteriorly and distinctly postfurcal (Fig. 146)	*Opius monilipalpis* sp. n.
27	Propodeum with medio-longitudinal carina anteriorly (Fig. 289) and vein 1r-m of hind wing about 0.4 times as long as vein 1-M (Fig. 287); anterior groove of metapleuron smooth (Fig. 286)	*Phaedrotoma semiplanata* sp. n.
–	Medio-longitudinal carina of propodeum variable, if present anteriorly (Figs 317, 325, 328, 353, 366) then vein 1r-m of hind wing 0.6–1.5 times as long as vein 1-M (Figs 316, 326, 335, 343, 352) and anterior groove of metapleuron crenulate (Figs 334, 342, 351)	28
28	Propodeum with medio-longitudinal carina anteriorly (Figs 317, 325, 328, 353, 366); vein m-cu of fore wing gradually merging into 2-CU1 and linear with vein 2-M or nearly so (Figs 287, 315, 335, 352, 364); vein 1r-m of hind wing less oblique and 0.6-1.0 times as long as vein 1-M (combined with a comparatively wide hind wing; Figs 316, 326, 334, 343, 352, 364); anterior groove of metapleuron crenulate (Figs 315, 334, 342, 351); vein CU1b of fore wing medium-sized (Figs 315, 335, 342, 343, 365); genus *Rhogadopsis* Brèthes, 1913	29
–	Medio-longitudinal carina of propodeum absent anteriorly (Figs 199, 240, 249, 266, 396); vein m-cu of fore wing angled with vein 2-M (Figs 197, 206, 228, 238, 276, 395), but rarely linear then angled with vein 2-CU1 (Fig. 248); vein 1r-m of hind wing usually distinctly oblique and 0.3-0.6 times as long as vein 1-M (Figs 197, 276, 296); at least dorsal half of anterior groove of metapleuron smooth (Figs 226, 295, 304); vein CU1b of fore wing usually short or absent (Figs 258, 305, 395), but sometimes moderately long (Figs 228, 258); genus *Phaedrotoma* Foerster, 1862	34
29	Medio-posterior depression of mesoscutum absent (Figs 325, 327, 336); posterior groove of pronotal side sometimes smooth (Fig. 334)	30
–	Medio-posterior depression of mesoscutum present (Figs 317, 344, 353, 355); ventral half of posterior groove of pronotal side crenulate (Figs 342, 351, 363)	31
30	First tergite elongate and with median carina (Fig. 337); propodeum largely smooth (Fig. 336); setose part of ovipositor sheath about 0.7 times length of first tergite; clypeus wide (Fig. 339); posterior groove of pronotal side smooth (Fig. 334)	*Rhogadopsis longuria* (Chen & Weng, 1995) comb. n.
–	First tergite normal and without median carina (Figs 325, 328); propodeum largely rugulose or rugose (Fig. 328); setose part of ovipositor sheath about 4 times length of first tergite (Fig. 325); clypeus comparatively narrow (Fig. 330); posterior groove of pronotal side crenulate; [setose part of ovipositor sheath 0.5 times as long as fore wing and twice as long as hind tibia]	*Rhogadopsis longicaudifera* sp. n.
31	Vein m-cu of fore wing slightly longer than vein 2-SR+M (Fig. 352); basal cell of hind wing wide and vein 1r-m of hind wing 0.8-1.0 times as long as vein 1-M (Fig. 352); second and third tergites smooth (Fig. 354) or superficially aciculate	*Rhogadopsis obliqua* sp. n.
–	Vein m-cu of fore wing distinctly longer than vein 2-SR+M (Figs 315, 343, 364); basal cell of hind wing narrower and vein 1r-m of hind wing 0.6–0.7 times or about 1.5 times as long as vein 1-M (Figs 316, 343, 364); sculpture of second and third tergites variable	32
32	First tergite about 1.4 times as long as wide (Fig. 367); second and third tergites finely longitudinally rugulose-striate (Fig. 367); hind femur comparatively slender (Fig. 368); length of eye about 4 times temple in dorsal view	*Rhogadopsis sculpturator* sp. n.
–	First tergite about as long as wide (Figs 307, 334); second and third tergites smooth (Figs 307, 324); hind femur robust (Figs 323, 346); length of eye 1.5–2.8 times temple in dorsal view; [only males known]	33
33	Vein 1r-m of hind wing 0.6-0.7 times as long as vein 1-M (Fig. 343); meso-scutum slightly wider than long and with long medio-posterior depression (Fig. 344); area below pterostigma slightly infuscate (Fig. 343); length of eye about 1.5 times temple in dorsal view	*Rhogadopsis maculosa* sp. n.
–	Vein 1r-m of hind wing about 1.5 times as long as vein 1-M (Fig. 316); meso-scutum distinctly wider than long and with short medio-posterior depression (Fig. 317); area below pterostigma hyaline (Fig. 315); length of eye about 2.8 times temple in dorsal view (Fig. 322)	*Rhogadopsis latipennis* sp. n.
34	Pronotal side striate (Figs 295, 303); head strongly transverse and yellow (Fig. 302); clypeus very wide (Fig. 300); [second metasomal tergite smooth; female unknown]	*Phaedrotoma striatinota* sp. n.
–	Pronotal side largely smooth or coriaceous (Figs 196, 205, 226); head at most moderately transverse and yellow or dark brown (Fig. 254); clypeus narrower (Figs 242, 252, 270, 281), but wide in *Phaedrotoma depressa* (Fig. 233)	35
35	Vertex and mesosoma densely coriaceous; vein SR1 of fore wing about 3 times as long as vein 3-SR	*Phaedrotoma terga* (Chen & Weng, 2005) comb. n.
–	Vertex and mesosoma mainly smooth (Fig. 226); vein SR1 of fore wing shorter (Figs 197, 216, 238), except in *Phaedrotoma depressa* (Figs 228, 229)	36
36	Vein SR1 of fore wing 3.4-4.0 times as long as vein 3-SR (Figs 228, 229); clypeus depressed ventrally and narrow sickle-shaped (Fig. 233); mesosoma (except black mesoscutum) orange-brown or largely dark brown (Figs 225–227); second and third metasomal tergite micro-sculptured and propodeum smooth (Fig. 231)	*Phaedrotoma depressa* nom. n.
–	Vein SR1 of fore wing 1.4-3.4 times as long as vein 3-SR (Figs 197, 216, 238, 248, 276); clypeus normal ventrally and semicircular or narrow and transverse (Figs 242, 252, 270, 281); mesosoma completely or largely black (Figs 198, 207, 239, 259) or entirely pale yellow (Figs 215, 249); second and third metasomal tergite variable, if micro-sculptured then propodeum rugulose (Figs 199, 218)	37
37	Clypeus 3.3–4.0 times wider than high (Figs 252, 261)	38
–	Clypeus 2.0–3.1 times wider than high (Figs 202, 211, 220, 242, 270, 281, 309)	39
38	Antenna of ♂ with about 27 segments and 1.4 times as long as fore wing (Fig. 247); body brownish-yellow and head ivory (Figs 247, 249, 252); legs pale yellow (Fig. 247); vein SR1 1.9 times vein 3-SR (Fig. 248); vein 3-SR nearly twice as long as vein 2-SR (Fig. 248)	*Phaedrotoma flavisoma* sp. n.
–	Antenna of ♂ with about 21 segments and about as long as fore wing (Fig. 257); body black (Figs 257, 259, 263); legs dark brown (Fig. 257); vein SR1 of fore wing 3 times as long as vein 3-SR (Fig. 258); vein 3-SR 1.7 times as long as vein 2-SR (Fig. 258)	*Phaedrotoma nigrisoma* sp. n.
39	First metasomal tergite longitudinally costate-striate (Fig. 307); anterior groove of metapleuron smooth or finely crenulate (Fig. 304); propodeum largely vermiculate-rugose (Fig. 307); clypeus slightly protruding medially (Fig. 309); apical half of first tergite widened apically and comparatively short (Fig. 307); occipital carina remain far removed from hypostomal carina (Fig. 310)	*Phaedrotoma vermiculifera* sp. n.
–	First tergite reticulate-rugose or granulate (Figs 199, 208, 218, 240, 267, 278); anterior groove of metapleuron often crenulate (Figs 196, 403); propodeum largely rugulose, rugose or smooth (Figs 199, 207, 217, 240, 267, 278); clypeus truncate medially (Figs 202, 211, 220, 242, 270, 281, 399); apical half of first metasomal tergite subparallel-sided (Figs 199, 208, 218, 240, 267, 397, 406) or diverging and more elongate (Figs 267, 278); occipital carina comparatively close to hypostomal carina (Figs 400, 410); [malar suture partly shallowly impressed or absent]	40
40	Anterior groove of metanotum smooth (Fig. 205); propodeum largely smooth and shiny posteriorly (Fig. 207); hind tarsus pale yellowish as basal half of hind tibia (Figs 205, 209); [length of malar space 0.6 times basal width of mandible (Fig. 212); face mainly dark brown or brownish-yellow (Fig. 211); third antennal segment about 4.3 (female) times as long as wide; antenna 1.3 times longer than fore wing. If length of malar space 0.3 times basal width of mandible, face ivory and antenna 1.6 times longer than fore wing, cf. *Phaedrotoma flavisoma*sp. n.]	*Phaedrotoma angiclypeata* sp. n.
–	Anterior groove of metanotum crenulate (Figs 196, 403); propodeum more or less rugulose or rugose and matt posteriorly (Figs 199, 217, 240, 267, 278); colour of hind tarsus variable, if slightly infuscate then darker than basal half of hind tibia (Fig. 237)	41
41	Setose part of ovipositor sheath 0.6-0.8 times as long as hind tibia (Figs 215, 218, 223, 275, 283, 284); pronotum with large round pronope (Figs 222, 285); propodeum usually largely densely rugose (Figs 217, 278); second and third metasomal tergites superficially granulate (Figs 218, 278)	42
–	Setose part of ovipositor sheath 0.1–0.3 times as long as hind tibia (Figs 196, 204, 237, 245, 401, 411); pronope, sculpture of propodeum and second and third tergites variable	43
42	Vein m-cu of fore wing postfurcal (Fig. 276); precoxal sulcus narrow, linear (Fig. 275); mesosoma dark brown or blackish laterally and ventrally (Fig. 275)	*Phaedrotoma rugulifera* sp. n.
–	Vein m-cu of fore wing slightly antefurcal (Fig. 216); precoxal sulcus somewhat wider, in elliptical depression (Fig. 215); mesosoma yellowish-brown laterally and ventrally (Fig. 215)	*Phaedrotoma antenervalis* sp. n.
43	Length of mesosoma about 1.5 times its height (Fig. 237); antenna of female about 1.3 times as long as fore wing (Fig. 237); length of eye in dorsal view about 3.5 times temple; clypeus depressed medially (Figs 242, 243); propodeum coarsely rugose (Fig. 240)	*Phaedrotoma depressiclypealis* sp. n.
–	Length of mesosoma 1.2–1.3 times its height (Figs 196, 264, 394, 403); antenna of female 1.5–1.7 times as long as fore wing (Figs 196, 403; male: 1.5–1.6 times); length of eye in dorsal view 1.5–2.8 times temple; clypeus convex medially (Figs 203, 271, 400, 410)); sculpture of propodeum variable, from superficially granulate-rugulose (Fig. 266) to rather coarsely rugose (Fig. 199)	44
44	Pronotal side and mesopleuron superficially granulate; precoxal sulcus wide and comparatively shallow, densely finely sculptured (Fig. 196); hind tarsus slender and pale yellowish as femur (Fig. 200); length of eye in dorsal view about 2.8 times temple	*Phaedrotoma acuticlypeata* sp. n.
–	Pronotal side and mesopleuron largely smooth or nearly so; precoxal sulcus narrow and deep, sparsely finely sculptured or smooth (Figs 264, 394, 403); hind tarsus less slender and often slightly darker than femur (Figs 264, 268, 403), but sometimes similar to hind tibia (Figs 394, 398); length of eye in dorsal view about 1.5 times temple	*Phaedrotoma protuberator* sp. n.

## Systematics

### Genus Apodesmia

Foerster, 1862

http://species-id.net/wiki/Apodesmia

**Figure 13.**
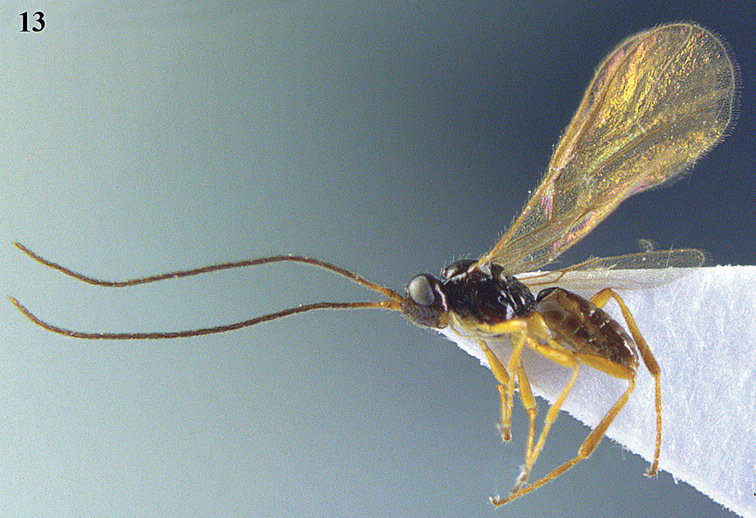
*Apodesmia bruniclypealis* sp. n., male, holotype. Habitus lateral.

Figs 13 [Fig F14] [Fig F15] –31

Apodesmia Foerster, 1862: 259. Type species (by original designation): *Apodesmia taeniata* Foerster, 1862 [examined].
Allotypus Foerster, 1862: 259. Type species (by original designation): *Opius irregularis* Wesmael, 1835 [examined]. **Syn. n.**
Lemnaphilopius Fischer, 1972b: 70. Type species (by original designation): *Opius lemnaphilae* Muesebeck, 1939 [examined; according to Fischer (1999) a valid genus with veins cu-a and 2-1A of fore wing reduced]. **Syn. n.**
Agnopius Fischer, 1982: 21. Type species (by original designation): *Opius similis* Szépligeti, 1898 [examined]. **Syn. n.**
Cryptognathopius Fischer, 1984a: 34. Type species (by original designation): *Opius uttoi* Fischer, 1971 [examined]. **Syn. n.**

#### Diagnosis.

**Figures 14–21. F14:**
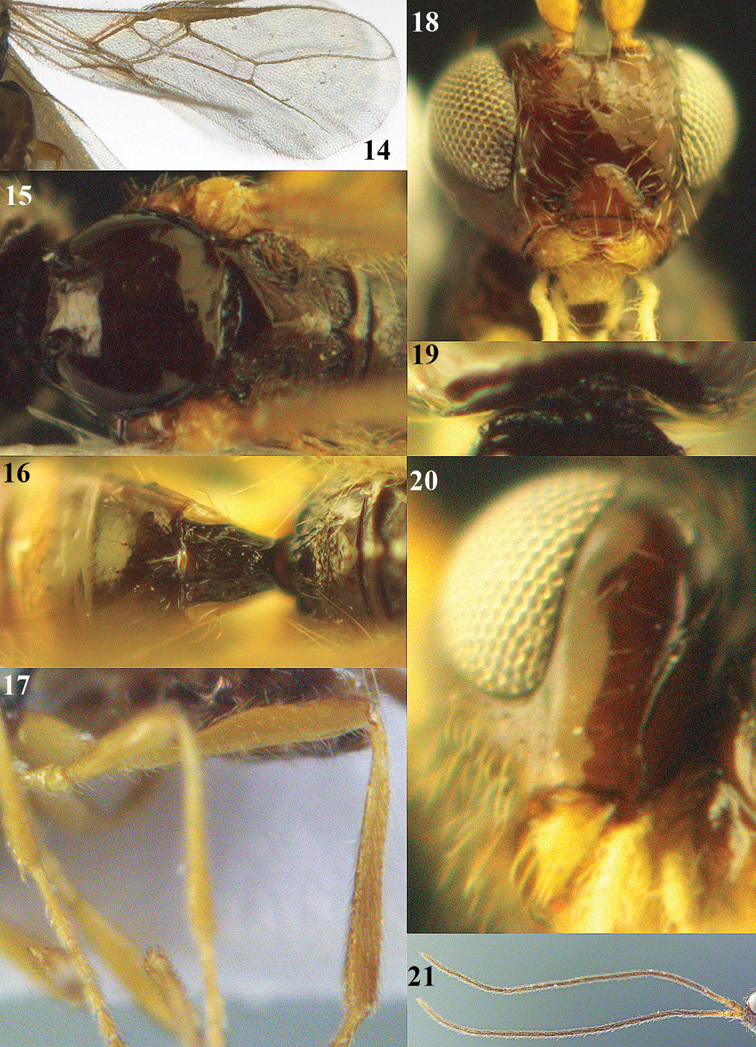
*Apodesmia bruniclypealis* sp. n., male, holotype. **14** Wings **15** mesosoma dorsal **16** propodeum and 1^st^-3^rd^ metasomal tergites dorsal **17** hind leg **18** head anterior **19** pronope dorsal **20** head lateral **21** antennae.

Occipital carina present laterally, above mandibular base curved towards and (just) meeting hypostomal carina (Figs 20, 29), near level of middle of eye straight or nearly so, without transverse carina or crest; face without tubercles (Figs 18, 27); scapus, fore coxa and trochanter at most weakly compressed; epistomal suture without large depressions; labrum normal, without emargination ventrally; clypeus not impressed; mandible not abruptly widened basally (but frequently rather gradually widened), without lamella, at most with a fine carina baso-ventrally; medio-posterior depression of mesoscutum present (Fig. 24), but rarely absent (Fig. 15); scutellar sulcus wide to rather narrow; postpectal carina absent; sfirst subdiscal cell of fore wing at least partly closed by vein 3-CU1 postero-apically; vein cu-a of hind wing nearly always present (Figs 14, 23); (but Nearctic species may have both veins cu-a of hind wing and CU1b of fore wing absent); vein 3-SR of fore wing distinctly longer than vein 2-SR (Figs 14, 23); vein m-cu of hind wing usually absent or obsolescent, only distinct in type species; first tergite without dorsope (Figs 16, 25); and basal half of third tergite without sharp lateral crease, if sometimes weakly developed then second tergite smooth; length of second and third tergites combined less than 0.7 times length of metasoma behind first tergite; vein 2-SR of fore wing present (Figs 14, 23), rarely reduced; fifth and following tergites partly exposed; ovipositor sheath more or less setose basally (Fig. 31); length of fore wing usually less than 3 mm.

#### Biology.

Parasitoids of mining Agromyzidae, Anthomyiidae, Lonchaeidae, Scathophagidae and Tephritidae.

#### Notes.

This Holarctic and Oriental genus has been used mostly as a subgenus (e.g., Fischer 1972b, Chen and Weng 2005 and 21998) but Papp (2002) used it as a valid genus. The definition in these papers is different; its main character, the curvature of the occipital carina, is used here for the first time. The mainly plesiomorphic character states used by other authors result in a mixed group; the curved occipital carina seems to be the best apomorphic character state to separate this group from *Opius* Wesmael s.l.

### Apodesmia bruniclypealis

Li & van Achterberg sp. n.

urn:lsid:zoobank.org:act:6B16C216-5B4A-4C8F-B6FC-5FD872FA1B55

http://species-id.net/wiki/Apodesmia_bruniclypealis

Figs 13 –21

#### Type material.

Holotype, ♂ (ZUH), “S. China: Hunan, nr Chengbu, Nan Mt., Shaoyang, 1500 m, 10–11.VI.2009, Xi-Ying Li, RMNH’09”.

#### Diagnosis.

Mandible with a short ventral carina (Fig. 20); clypeus dark brown; hind tibia completely pale yellowish or brownish-yellow basally; medio-posterior depression of mesoscutum absent (Fig. 15); mesosoma slender (Fig. 13); propodeum without a transverse carina subbasally (Fig. 16); vein m-cu of fore wing slightly postfurcal (Fig. 14).

#### Description.

Holotype, ♂, length of body 1.6 mm, of fore wing 2.2 mm.

*Head*. Antenna with 26 segments and 1.3 times as long as fore wing (Fig. 13); length of third segment 1.5 times fourth segment and without thyloids and shiny, length of third, fourth and penultimate segments 5.3, 3.5, and 2.5 times their width, respectively (Fig. 21); length of maxillary palp 1.2 times height of head; labial palp segments rather short; occipital carina dorsally absent; median groove behind stemmaticum absent; hypostomal carina narrow (Fig. 20); length of eye in dorsal view twice temple; frons flat and glabrous medially, smooth and laterally distinctly convex and glabrous; face smooth, medially slightly elevated (Fig. 18); width of clypeus 1.8 times its maximum height and 0.55 times width of face, clypeus convex, smooth and its ventral margin differentiated and slightly concave (Fig. 18); hypoclypeal depression medium-sized (Fig. 18); malar suture indicated as slightly impressed narrow groove; mandible triangular, moderately convex and with fine ventral carina (Fig. 20).

*Mesosoma*. Length of mesosoma 1.4 times its height; dorsal pronope absent (Fig. 19); pronotal side smooth, but posteriorly slightly superficially crenulate; epicnemial area smooth dorsally; precoxal sulcus only medially distinctly impressed, narrow and smooth (Fig. 13); rest of mesopleuron smooth; pleural sulcus smooth, but ventrally slightly crenulate; mesosternal sulcus narrow and finely crenulate; notauli absent on disc, except for a short crenulate part anteriorly (Fig. 15); mesoscutum glabrous; medio-posterior depression of mesoscutum absent (Fig. 15); scutellar sulcus narrow and finely crenulate; scutellum smooth and slightly convex; dorsal surface of propodeum largely smooth without medio-longitudinal carina and posteriorly mainly finely rugulose (Fig. 16).

*Wings*. Fore wing (Fig. 14): pterostigma sublinear; 1-R1 reaching wing apex and 1.3 times as long as pterostigma; r:3-SR:SR1 = 3:21:43; 2-SR:3-SR:r-m = 10:21:6; r slightly widened; 1-M straight; SR1 weakly sinuate; m-cu slightly postfurcal; cu-a slightly postfurcal and 1-CU1 slender; first subdiscal cell narrowly open, CU1b absent; apical quarter of M+CU1 sclerotized. Hind wing (Fig. 14): M+CU:1-M:1r-m = 10:9:5; cu-a straight, short; m-cu absent; 1-1A curved.

*Legs*. Length of femur, tibia and basitarsus of hind leg 4.6, 9.5 and 7.2 times as long as wide, respectively; setae of hind femur and tibia moderately long (Fig. 17).

*Metasoma*. Length of first tergite 1.3 times its apical width, its surface evenly convex medially and with longitudinal (partly superficial) fine rugae and dorsal carinae remain separated and up to middle of tergite (Fig. 16); basal quarter of second tergite with oblique striae medially (Fig. 16); second suture obsolescent; third and following tergites smooth.

*Colour*. Dark brown; palpi and tegulum pale yellowish; scapus, pedicellus and base of third segment, legs (but hind femur and tibia slightly darkened apically), metasoma baso-ventrally and apex of third-seventh tergites brownish-yellow; mesopleuron below precoxal sulcus chestnut-brown; pterostigma and veins mainly pale brown; wing membrane subhyaline.

*Molecular data*. None.

#### Distribution.

*China (Hunan).

#### Biology.

Unknown.

#### Etymology.

Name derived from “brunneus” (Latin for brown) and “clypeus”, because of the brown clypeus.

#### Notes.

Runs in the key by Chen and Weng (2005) to *Phaedrotoma larga* (Weng & Chen, 2005) comb. n. It differs by having the occipital carina curved ventrally, the clypeus 1.8 times wider than high (2.6 times in *Phaedrotoma larga*) and the first tergite 1.3 times as long as wide apically (0.9 times).

### Apodesmia melliclypealis

Li & van Achterberg sp. n.

urn:lsid:zoobank.org:act:ACFE3F75-7C8F-4428-9C46-14A47E5B8E2E

http://species-id.net/wiki/Apodesmia_melliclypealis

Figs 22 –31

#### Type material.

Holotype, ♀ (ZUH), “S. China: Hunan, nr Zhangjiajie, Badagong Mts, Tian Ping Mt., 9–13.VII.2009, 550 m, Xi-Ying Li, RMNH’10”. Paratypes (RMNH): 1 ♀ with same label data; 1 ♀, “S. China: Hunan, nr Zhangjiajie, Badagong Mts, Longtanping, 4–5.VI.2009, 550 m, Xi-Ying Li, RMNH’09”; 1 ♀, “S. China: Hunan, nr Chengbu, Nan Mt., Shaoyang, 1500 m, 10–11.VI.2009, Xi-Ying Li, RMNH’09”; 1 ♀, “S. China: Hunan, nr Suining, Huangsang N.R., Shaoyang, 12–13.VI.2009, 1000 m, Xi-Ying Li, RMNH’09”.

#### Diagnosis.

**Figure 22. F15:**
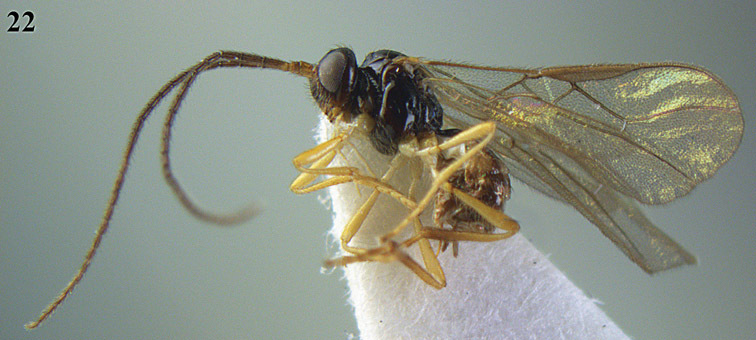
*Apodesmia melliclypealis* sp. n., female, holotype. Habitus lateral.

**Figures 23–31.**
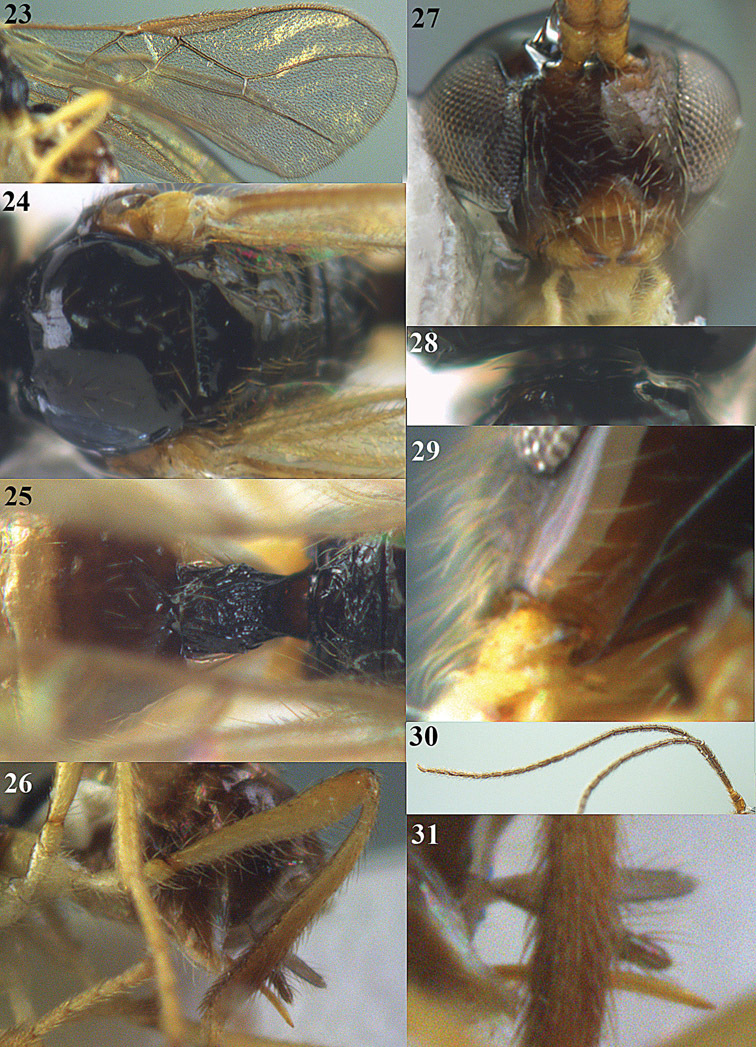
*Apodesmia melliclypealis* sp. n., female, holotype. **23** Wings **24** mesosoma dorsal **25** propodeum and 1^st^-3^rd^ metasomal tergites dorsal **26** hind leg **27** head anterior **28** pronope dorsal **29** head lateral **30** antennae **31** ovipositor sheath.

Mandible with a fine ventral carina (Fig. 29); clypeus yellowish-brown ventrally; small medio-posterior depression of mesoscutum present (Fig. 24); mesosoma robust (Fig. 22); propodeum without a transverse carina subbasally (Fig. 25); vein m-cu of fore wing slightly antefurcal (Fig. 23).

#### Description.

Holotype, ♀, length of body 1.7 mm, of fore wing 2.2 mm.

*Head*. Antenna with 28 segments and 1.4 times as long as fore wing (Fig. 22); length of third segment 1.2 times fourth segment and with thyloids and matt, length of third, fourth and penultimate segments 4.0, 3.3, and 2.5 times their width, respectively (Fig. 30); length of maxillary palp 1.1 times height of head; labial palp segments rather short; occipital carina dorsally absent; median groove behind stemmaticum absent; hypostomal carina narrow (Fig. 29); length of eye in dorsal view 3.0 times temple; frons flat and glabrous medially, smooth and laterally distinctly convex and glabrous; face smooth, medially slightly elevated (Fig. 27); width of clypeus twice its maximum height and 0.5 times width of face, clypeus moderately convex, largely smooth and its ventral margin differentiated and straight medially (Fig. 27); hypoclypeal depression medium-sized (Fig. 27); malar suture absent; mandible triangular, moderately convex and with fine ventral carina (Fig. 29).

*Mesosoma*. Length of mesosoma 1.2 times its height (Fig. 22); dorsal pronope obsolescent, small (Fig. 28); pronotal side smooth, but oblique groove finely crenulate, posteriorly largely smooth (Fig. 22); epicnemial area largely smooth dorsally; precoxal sulcus only medially distinctly impressed, narrow and smooth (Fig. 22); rest of mesopleuron smooth; pleural sulcus entirely smooth; mesosternal sulcus narrow and finely crenulate; notauli absent on disc, except for an indistinct smooth depression anteriorly (Fig. 24); mesoscutum glabrous except some setae on imaginary notaulic courses (Fig. 24); medio-posterior depression of mesoscutum small, pit-shaped (Fig. 24); scutellar sulcus narrow and finely crenulate; scutellum smooth and flattened; surface of propodeum partly rugulose and partly smooth, without medio-longitudinal carina (Fig. 25).

*Wings*. Fore wing (Fig. 23): pterostigma elliptical; 1-R1 reaching wing apex and 1.1 times as long as pterostigma; r:3-SR:SR1 = 2:31:60; 2-SR:3-SR:r-m = 15:31:8; r slightly widened; 1-M straight; SR1 weakly sinuate; m-cu slightly antefurcal; cu-a slightly postfurcal and 1-CU1 slender; first subdiscal cell narrowly open, CU1b reduced, only pigmented; apical quarter of M+CU1 sclerotized. Hind wing (Fig. 23): M+CU:1-M:1r-m = 15:2:5; cu-a straight, short; m-cu absent; 1-1A curved.

*Legs*. Length of femur, tibia and basitarsus of hind leg 5.0, 10.6 and 5.3 times as long as wide, respectively; setae of hind femur long and tibia moderately long (Fig. 26).

*Metasoma*. Length of first tergite 1.5 times its apical width, its surface evenly convex medially and with longitudinal (mainly superficial) fine rugae and dorsal carinae remain separated and up to middle of tergite (Fig. 25); basal quarter of second tergite smooth medially; second suture absent; third and following tergites smooth; length of setose part of ovipositor sheath 0.08 times fore wing and 0.25 times hind tibia (Figs 22, 31).

*Colour*. Dark brown; mesosoma and head dorsally blackish; palpi, tegulae and coxae pale yellowish; scapus, clypeus ventrally, remainder of legs (but hind tibia apically and hind tarsus slightly darkened), metasoma baso-ventrally and apex of third-seventh tergites brownish-yellow; mesopleuron below precoxal sulcus chestnut-brown; pterostigma and veins mainly brown; wing membrane subhyaline.

*Molecular data*. COI, 16S, 28S (CVA4235).

*Variation*. Length of body 1.5-1.9 mm, of fore wing 2.0-2.2 mm; antenna of ♀ with 25 (1), 26 (2), 27 (1) or 28 (1) segments.

#### Distribution.

*China (Hunan).

#### Biology.

Unknown.

#### Etymology.

Name derived from “melleus” (Latin for honey-coloured) and “clypeus”, because of the yellowish clypeus.

#### Notes.

According to the molecular markers belongs to a clade with the Palaearctic *Apodesmia incisula* (Fischer, 1964) (DNA: CVA4257, sharing the indistinct pronope and a medio-posterior depression of mesoscutum) together with *Neopius rudis* (Wesmael). The new species has vein m-cu of fore wing antefurcal as in the very common Palaearctic *Apodesmia irregularis* (Wesmael, 1835), but it has the second tergite smooth basally and rather dark brown, medio-posterior depression of the mesoscutum minute and distinctly removed from the scutellar sulcus (more than diameter of depression from scutellar sulcus; less than length of depression in *Apodesmia irregularis*), the first tergite with distinct (but small) smooth knob medio-apically (absent or nearly so in *Apodesmia irregularis*).

It runs in the key by Chen and Weng (2005) to *Apodesmia tracta* (Weng & Chen, 2005) comb. n. It differs by having the eye 3 times longer than temple (twice in *Apodesmia tracta*), the first tergite with finer sculpture between its coarse rugae (absent), the second tergite without distinct sculpture (longitudinally striate) and the base of the second tergite comparatively wide between the oblique depressions (base narrow).

### Genus Areotetes

van Achterberg & Li gen. n.

urn:lsid:zoobank.org:act:79C141F0-F174-4B6B-90D0-28FEFA95CB67

http://species-id.net/wiki/Areotetes

**Figure 32.**
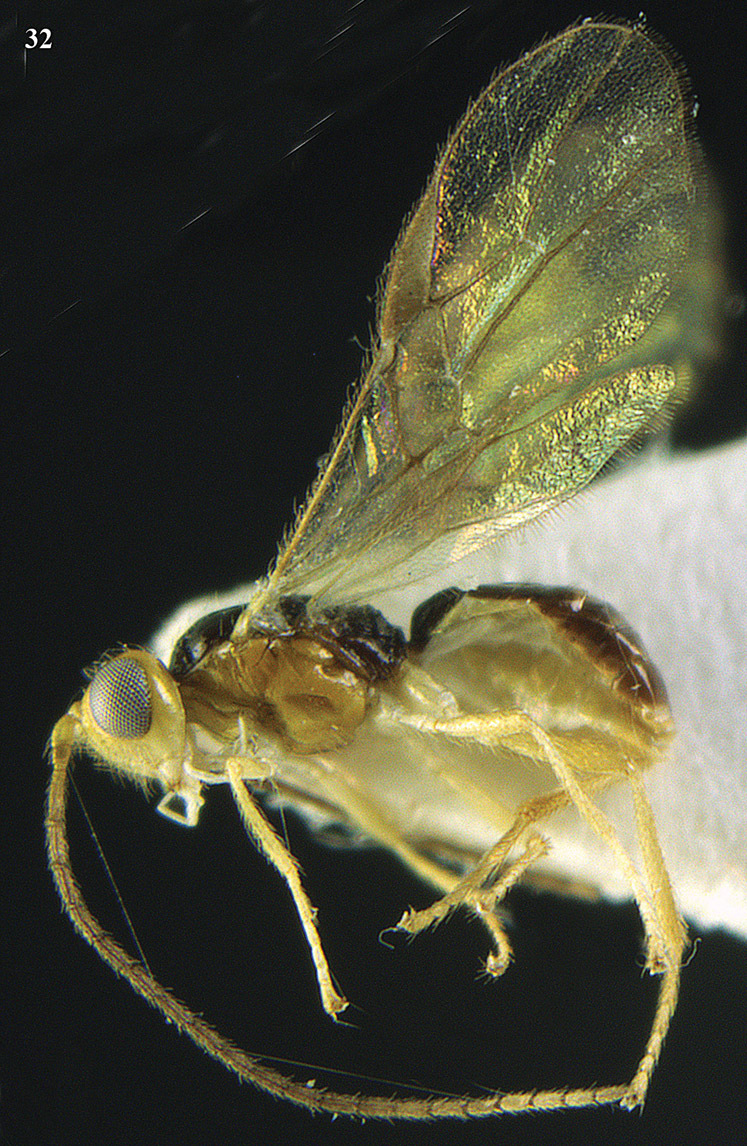
*Areotetes albiferus* sp. n., male, holotype. Habitus lateral.

Figs 32 [Fig F18] [Fig F19] [Fig F20] [Fig F21] –62

#### Type species.

*Areotetes carinuliferus* sp. n.

#### Etymology.

From “areola” (Latin for “room”) and the generic name *Utetes* Foerster, 1862, because it is similar but has an areolate propodeum. Gender: masculine.

#### Diagnosis.

**Figures 33–41. F18:**
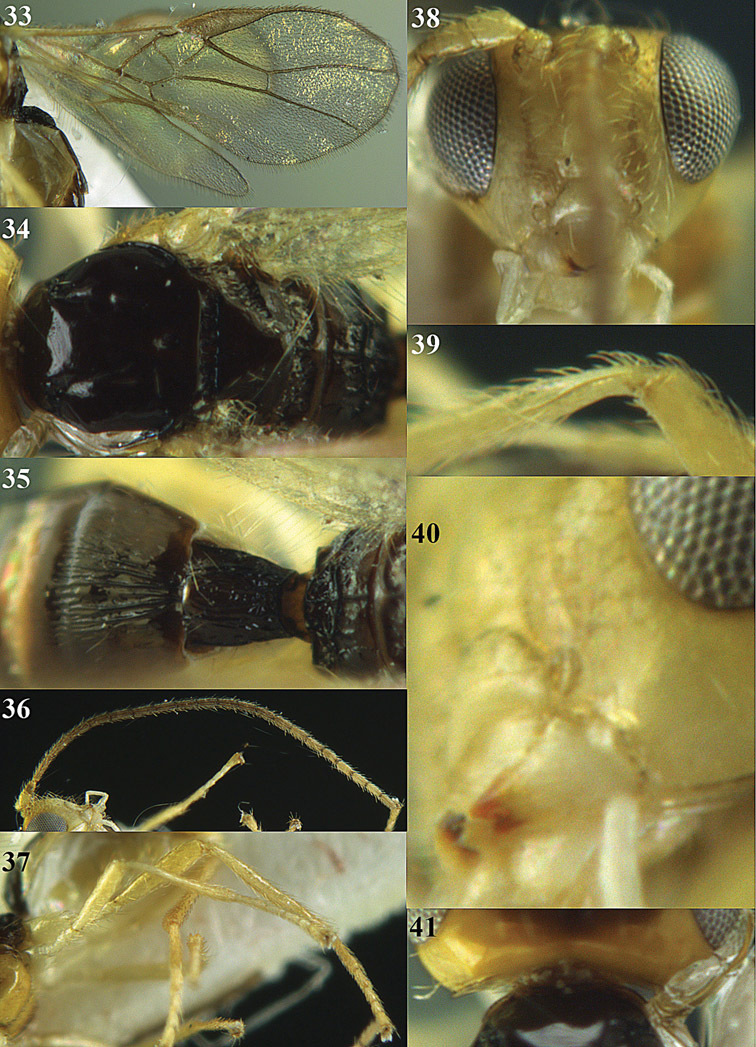
*Areotetes albiferus* sp. n., male, holotype. **33** Wings **34** mesosoma dorsal **35** propodeum and 1^st^-3^rd^ metasomal tergites dorsal **36** antenna **37** hind leg **38** head anterior **39** base of hind tibia inner side **40** mandible **41** pronope dorsal.

Hind tibia with a long nearly straight carinula basally (Figs 39, 47, 59); face without tubercles; in front of anterior ocellus without a distinct semi-circular or triangular depression; frons without a pair of distinct depressions above antennal sockets; occipital carina present laterally, not or slightly curved ventrally and remaining removed from hypostomal carina, near level of middle of eye straight or nearly so, without transverse carina or crest; clypeus more or less convex and high (Fig. 48); labrum normal, without emargination ventrally; hypoclypeal depression distinct (Fig. 48); malar suture absent; scapus, fore coxa and trochanter at most weakly compressed; epistomal suture without large depressions; mandible normal, triangular (Fig. 51); pronotum short and subvertical; pronope absent or obsolescent; side of pronotum anteriorly below groove with distinctly elevated area; medio-posterior depression of mesoscutum rather small (Fig. 34) or absent (e.g. in *Areotetes carinuliferus*); scutellar sulcus usually rather wide (Fig. 34); propodeum areolate and smooth between carinae, with medium-sized medio-longitudinal carina (Figs 35, 44, 56); precoxal sulcus smooth or finely crenulate; postpectal carina completely absent; vein 2-SR of fore wing present; first subdiscal cell of fore wing at least partly closed by vein 3-CU1 and short vein CU1b postero-apically (Figs 33, 43, 53); vein 1-M of fore wing straight or slightly curved and vein 1-SR short; vein cu-a of hind wing present and vein m-cu absent; vein 3-SR of fore wing distinctly longer than vein 2-SR (Figs 33, 43, 53); length of fore wing less than 3.5 mm; second tergite without sharp lateral crease, smooth or striate; length of second and third tergites combined less than 0.7 times length of metasoma behind first tergite; fourth and following tergites (at least partly) exposed; ovipositor sheath about 0.1 times as long as fore wing. According to Fig. 6 not closely related to the genus *Utetes* Foerster, despite having carinulae of the hind tibia in common.

#### Biology.

Unknown, butthe related genus *Utetes* Foerster, 1862, contains parasitoids of fruit infesting Tephritidae and to a lesser degree of Agromyzidae and Anthomyiidae; at least some of the host records may be the result of misidentification of the host or parasitoid.

#### Notes.

Can be separated from *Utetes* Foerster as follows:

**Table:** 

1	Epicnemial area crenulate; pronope slit-shaped and deep; clypeus thin and protruding ventrally; vein m-cu of fore wing far postfurcal; posterior face of propodeum without areolation; medio-longitudinal carina of propodeum short or absent; precoxal sulcus usually widely crenulate; length of fore wing often more than 3.5 mm	*Utetes* Foerster *sensu stricto*
–	Epicnemial area smooth; pronope round, if slit-shaped then indistinctly developed; clypeus obtuse and truncate ventrally; vein m-cu of fore wing slightly postfurcal; posterior face of propodeum with areolation; medio-longitudinal carina of propodeum medium-sized; precoxal sulcus smooth or finely crenulate; length of fore wing less than 3.5 mm	*Areotetes* van Achterberg & Li, gen. n.

### Areotetes albiferus

Li & van Achterberg sp. n.

urn:lsid:zoobank.org:act:7F60EAB3-39FC-4449-A8BD-D8E16A219BEE

http://species-id.net/wiki/Areotetes_albiferus

Figs 32 –41

#### Type material.

Holotype, ♂ (ZUH), “S. China: Hunan, nr Zhangjiajie, Badagong Mts, Bamaoxi, 2–3.VI.2009, 540 m, Xi-Ying Li, RMNH’09”.

#### Diagnosis.

Second metasomal tergite distinctly striate medially (Fig. 35); second metasomal suture widely crenulate; antenna of ♀ unknown, probably apical third pale yellowish as in male (Fig. 36); head dorsally (except stemmaticum and surroundings) yellow; face yellowish-brown; frons with pair of small depressions above antennal sockets; pronotum short and vertical; medio-posterior depression of mesoscutum small and deep (Fig. 34); precoxal sulcus smooth except for some crenulae (Fig. 32); vein 3-SR of fore wing about 1.7 times as long as vein 2-SR and nearly straight (Fig. 33); vein m-cu of fore wing angulate with vein 2-CU1.

#### Description.

Holotype, ♂, length of body 1.8 mm, of fore wing 2.1 mm.

*Head*. Antenna with 21 segments and 1.2 times as long as fore wing (Fig. 32); length of third segment 1.3 times fourth segment, length of third, fourth and penultimate segments 4.7, 3.7, and 2.7 times their width, respectively (Fig. 36); length of maxillary palp 1.3 times height of head; labial palp segments elongate (Fig. 32); occipital carina moderately close to hypostomal carina and dorsally absent (Fig. 40); median groove behind stemmaticum present; hypostomal carina narrow; length of eye in dorsal view 3.4 times temple; frons flat and glabrous medially, smooth and laterally convex and largely glabrous; face smooth, medially broadly elevated (Fig. 38); width of clypeus 1.6 times its maximum height and 0.4 times width of face, clypeus convex, smooth and its ventral margin not differentiated and concave (Fig. 38); hypoclypeal depression medium-sized (Fig. 38); mandible convex (Fig. 40).

*Mesosoma*. Length of mesosoma 1.5 times its height; dorsal pronope absent (Fig. 41); pronotal side smooth, but medial groove with ventral oblique carina posteriorly and a short carina perpendicularly connected to it and posterior groove obsolescent (Fig. 32); epicnemial area smooth dorsally; precoxal sulcus only medially distinctly impressed, with few short crenulae (Fig. 32); rest of mesopleuron smooth; pleural sulcus smooth; mesosternal sulcus narrow and finely crenulate; notauli absent on disc, except for a short crenulate part anteriorly (Fig. 34); mesoscutum glabrous; medio-posterior depression of mesoscutum rather small, deep (Fig. 34); scutellar sulcus moderately crenulate; scutellum smooth and flat; dorsal surface of propodeum largely smooth except for medio-longitudinal carina and a strong transverse carina behind it, posteriorly areolate (Fig. 35).

*Wings*. Fore wing (Fig. 33): pterostigma triangular; 1-R1 reaching wing apex and 1.4 times as long as pterostigma; r:3-SR:SR1 = 1:18:37; 2-SR:3-SR:r-m = 11:18:4; r widened; 1-M nearly straight; SR1 weakly curved; m-cu slightly postfurcal; cu-a slightly postfurcal and 1-CU1 widened; first subdiscal cell closed, CU1b short; apical quarter of M+CU1 sclerotized. Hind wing (Fig. 33): M+CU:1-M:1r-m = 15:13:7; cu-a straight; m-cu absent.

*Legs*. Length of femur, tibia and basitarsus of hind leg 4.2, 8.0 and 4.3 times as long as wide, respectively; setae of hind femur and tibia moderately long (Fig. 37).

*Metasoma*. Length of first tergite 1.2 times its apical width, its surface evenly convex medially and with longitudinal (medially irregular) costae and dorsal carinae united in its anterior 0.3 and up to apex (Fig. 35); second tergite medially longitudinally costate (Fig. 35); second suture widely crenulate (Fig. 35); third and following tergites smooth.

*Colour*. Dark brown; apical third of antenna pale (3 penultimate segments pale yellowish, apical and other segments pale brownish; Fig. 36); palpi, face, mandible, malar space, tegulae, legs (but middle and hind tarsi somewhat brownish), apex of third-seventh tergites and metasoma ventrally whitish or pale yellowish; scapus, remainder of head (but stemmaticum and surroundings dark brown), prothorax, mesopleuron and mesosternum yellow; pterostigma and veins mainly brown; wing membrane subhyaline.

*Molecular data*. None.

#### Distribution.

*China (Hunan).

#### Biology.

Unknown.

#### Etymology.

Name derived from “albus” (Latin for “white”) and “fero” (Latin for “carrying”), because of the whitish mandibles and the pale apex of the antenna.

#### Notes.

Does not run in the key by Chen and Weng (2005) to any species of the group with a carinula on the hind tibia.

### Areotetes carinuliferus

Li & van Achterberg sp. n.

urn:lsid:zoobank.org:act:0E2F0F10-3941-4AD6-B0AB-1F0F511144F8

http://species-id.net/wiki/Areotetes_carinuliferus

Figs 42 –51

#### Type material.

Holotype, ♀ (ZUH), “S. China: Hunan, nr Zhangjiajie, Badagong Mts, Longtanping, 4–5.VI.2009, 550 m, Xi-Ying Li, RMNH’09”, “4249, sp. 15” [= DNA sample taken from this specimen]. Paratypes (RMNH): 1 ♂, same label data; 3 ♀, id., but Bamaoxi, 2–3.VI.2009, 540 m; 1 ♂, “S. China: Hunan, nr Chengbu, Nan Mt., Shaoyang, 1500 m, 10–11.VI.2009, Xi-Ying Li, RMNH’09”.

#### Diagnosis.

Second metasomal tergite smooth or nearly so (Fig. 45); apical third of antenna of ♀ dark brown subapically; face dark brown; frons without depressions above or between antennal sockets; pronotum short and vertical; medio-posterior depression of mesoscutum absent (Fig. 44); scutellar sulcus finely crenulate; precoxal sulcus narrowly crenulate; propodeum areolate and smooth between carinae (Fig. 44); vein 3-SR of fore wing slightly curved and twice as long as vein 2-SR (Fig. 43); vein m-cu of fore wing gradually merging into vein 2-CU1; first metasomal tergite partly smooth and shiny (Fig. 45).

#### Description.

Holotype, ♀, length of body 1.7 mm, of fore wing 2.0 mm.

*Head*. Antenna with 21 segments and 1.2 times as long as fore wing; length of third segment 1.2 times fourth segment, length of third, fourth and penultimate segments 5.5, 4.5 and 3.5 times their width, respectively (Fig. 46); length of maxillary palp 1.3 times height of head; labial palp segments elongate; occipital carina moderately close to hypostomal carina and dorsally absent; median groove behind stemmaticum obsolescent; hypostomal carina narrow; length of eye in dorsal view 3.6 times temple; frons flat and glabrous medially, smooth and laterally convex and largely glabrous; face smooth, medially elevated (Fig. 48); width of clypeus 1.8 times its maximum height and 0.5 times width of face, clypeus convex, smooth and its ventral margin not differentiated and straight medially (Fig. 48); hypoclypeal depression medium-sized (Fig. 48); mandible slightly convex and with fine ventral carina (Fig. 51).

**Figure 42. F19:**
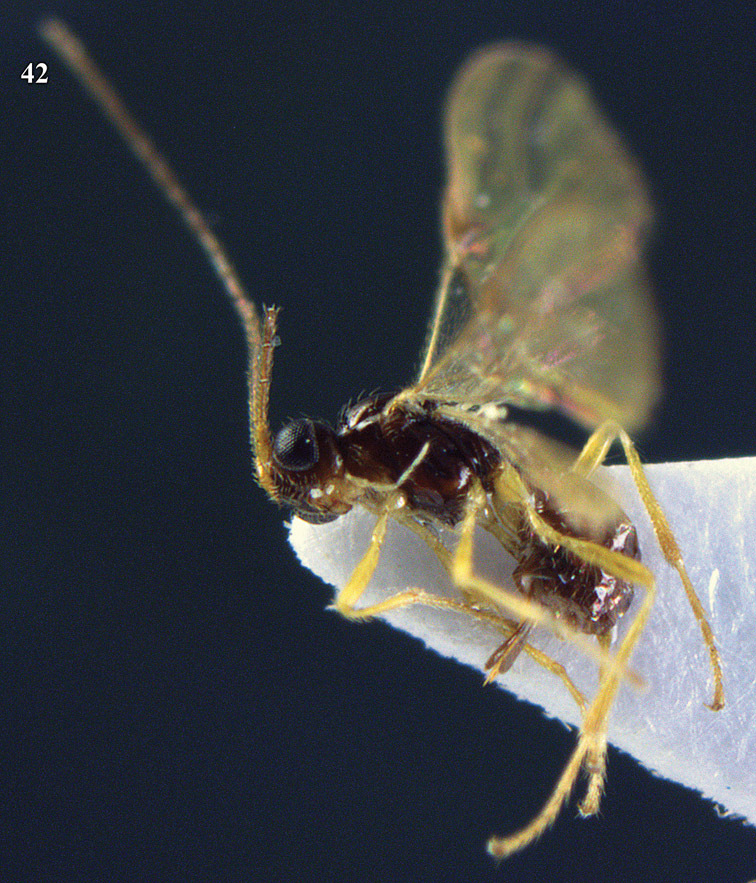
*Areotetes cariniferus* sp. n., female, holotype. Habitus lateral.

*Mesosoma*. Length of mesosoma 1.5 times its height; dorsal pronope absent; pronotal side smooth, but medial groove indistinctly crenulate posteriorly, with ventral oblique carina posteriorly and posterior groove obsolescent (Fig. 42); epicnemial area smooth dorsally; precoxal sulcus only medially distinctly impressed, posteriorly with few indistinct crenulae (Fig. 42); rest of mesopleuron smooth; pleural sulcus smooth; mesosternal sulcus narrow and finely crenulate; notauli absent on disc, except for a short smooth part anteriorly (Fig. 44); mesoscutum glabrous; medio-posterior depression of mesoscutum absent (Fig. 44); scutellar sulcus finely crenulate; scutellum smooth and flat; dorsal surface of propodeum smooth except for a medio-longitudinal carina connected to a pentagonal areola and complete costulae (Fig. 44).

**Figures 43–51. F20:**
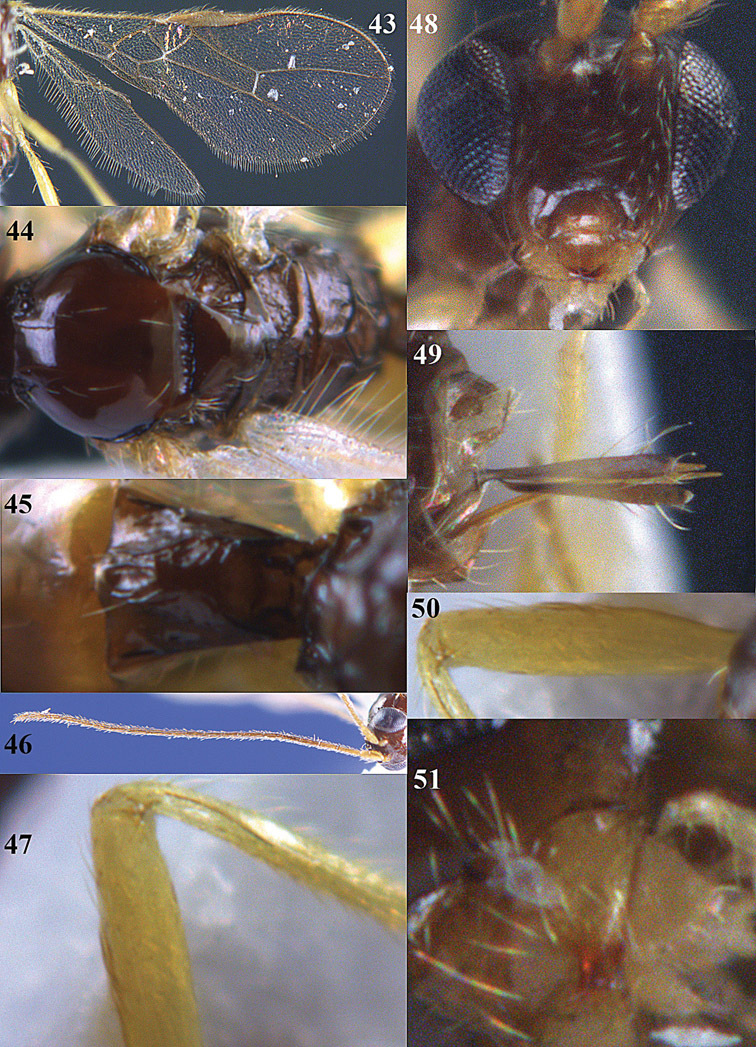
*Areotetes cariniferus* sp. n., female, holotype. **43** Wings **44** mesosoma dorsal **45** propodeum and 1^st^-2^nd^ metasomal tergites dorsal **46** antenna **47** hind leg, inner side **48** head anterior **49** ovipositor sheath **50** hind femur **51** mandible.

*Wings*. Fore wing (Fig. 43): pterostigma nearly elliptical; 1-R1 reaching wing apex and 1.5 times as long as pterostigma; r:3-SR:SR1 = 2:33:56; 2-SR:3-SR:r-m = 14:33:10; r widened; 1-M, 3-SR and SR1 nearly straight; m-cu slightly postfurcal and gradually merging into vein 2-CU1; cu-a slightly postfurcal and 1-CU1 widened; first subdiscal cell closed, CU1b short; apical quarter of M+CU1 sclerotized. Hind wing (Fig. 43): M+CU:1-M:1r-m = 10:11:5; cu-a straight; m-cu absent.

*Legs*. Length of femur, tibia and basitarsus of hind leg 5.2, 10.7 and 8.0 times as long as wide, respectively; setae of hind femur long and of tibia moderately long (Fig. 50); hind tibia with a long nearly straight carinula basally (Fig. 47).

*Metasoma*. Length of first tergite 1.1 times its apical width, its surface evenly convex medially, largely smooth, but with a few oblique weak carinae and dorsal carinae united in its anterior 0.3 and absent behind it (Fig. 45); second tergite smooth, but antero-medially with indistinct patch of sculpture; second suture smooth and shallowly impressed; third and following tergites smooth and partly desclerotized apically; length of setose part of ovipositor sheath 0.1 times fore wing and 0.3 times hind tibia (Fig. 49).

*Colour*. Dark brown; antenna dark brown but 4 basal segments brownish-yellow; palpi, mandible, tegulae, legs, base of second tergite and apex of third tergite pale yellowish; clypeus and malar space mainly, side of pronotum ventrally, propleuron and area below precoxal sulcus yellowish-brown; pterostigma and veins mainly brown; wing membrane subhyaline.

*Molecular data*. COI, 16S, 28S (CVA4249).

*Variation*. Length of body 1.4–1.7 mm, of fore wing 2.0-2.1 mm; antenna of ♀ with 20 (2) or 21 (2) segments, of ♂ with 21 (1) or 22 (1) segments; third and fourth antennal segments and face dark brown or yellowish-brown.

#### Etymology.

Name derived from “carinula” (Latin for small carina or crest) and “fero” (suffix in Latin meaning carrying or having), because of the carinula on the inner side of the hind tibia.

#### Notes.

Does not run well in the key by Chen and Weng (2005) to any species of the group with a carinula of the hind tibia. It is similar to *Areotetes laevigatus* (Weng & Chen, 2005) comb. n., but it has the length of the mesosoma 1.5 times its height (1.7 times in *Areotetes laevigatus*), the propodeum without a median carina posteriorly (present), vein 3-SR of fore wing about 2.4 times vein 2-SR (about twice) and length of the hind femur about 5 times as long as wide (4 times).

### Areotetes striatiferus

Li & van Achterberg sp. n.

urn:lsid:zoobank.org:act:8D1A6F6E-DBB8-4EC1-85F1-88FD0F59D5B7

http://species-id.net/wiki/Areotetes_striatiferus

Figs 52 –62

#### Type material.

Holotype, ♀ (ZUH), “S. China: Hunan, nr Zhangjiajie, Badagong Mts, Tian Ping Mt., 9–13.VII.2009, 550 m, Xi-Ying Li, RMNH’10”. Paratype, ♂ (RMNH), same label data.

#### Diagnosis.

**Figure 52. F21:**
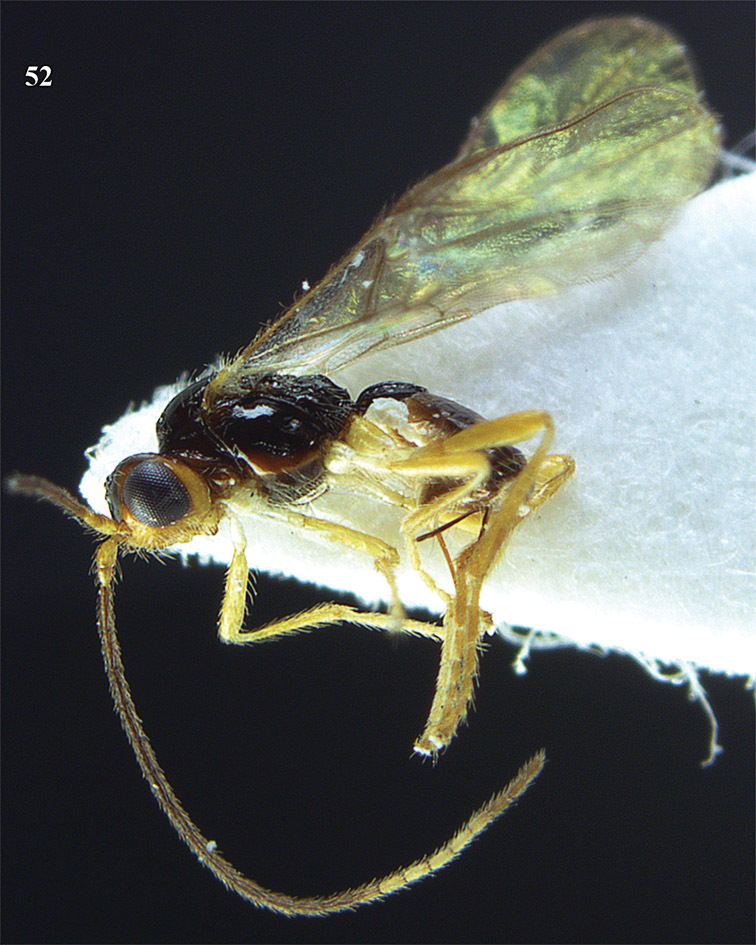
*Areotetes striatiferus* sp. n., female, holotype. Habitus lateral.

Second metasomal tergite striate medially (Fig. 56); apical third of antenna of ♀ partly pale yellowish (Fig. 61), of ♂ dark brown; face brownish-yellow and head dorsally largely dark brown; frons with small pit between sockets and no depressions above antennal sockets; medio-posterior depression of mesoscutum small, round (Fig. 55); scutellar sulcus finely crenulate; precoxal sulcus narrowly crenulate (Fig. 52); propodeum areolate and with smooth areas (Fig. 56); vein 3-SR of fore wing slightly curved and about 2.5 times as long as vein 2-SR; vein m-cu of fore wing gradually merging into vein 2-CU1; first metasomal tergite sparsely sculptured (Fig. 56).

#### Description.

Holotype, ♀, length of body 1.8 mm, of fore wing 2.2 mm.

*Head*. Antenna with 24 segments and 1.4 times as long as fore wing; length of third segment 1.3 times fourth segment, length of third, fourth and penultimate segments 5.0, 4.0 and 2.6 times their width, respectively (Fig. 61); length of maxillary palp 1.3 times height of head; labial palp segments elongate; occipital carina moderately close to hypostomal carina and dorsally absent (Fig. 62); median groove behind stemmaticum obsolescent; hypostomal carina narrow; length of eye in dorsal view 5.5 times temple; frons flat and glabrous medially, smooth and laterally convex and largely glabrous; face smooth, medially elevated; width of clypeus twice its maximum height and 0.45 times width of face, clypeus convex, smooth and its ventral margin not differentiated and concave (Fig. 58); hypoclypeal depression medium-sized (Fig. 58); mandible slightly convex and with fine ventral carina (Fig. 62).

*Mesosoma*. Length of mesosoma 1.5 times its height; dorsal pronope absent; pronotal side smooth, but medial groove indistinctly crenulate, with ventral oblique carina posteriorly and posterior groove obsolescent (Fig. 52); epicnemial area smooth dorsally; precoxal sulcus only medially distinctly impressed, with short crenulae (Fig. 52); rest of mesopleuron smooth; pleural sulcus smooth; mesosternal sulcus narrow and finely crenulate; notauli absent on disc, except for a short largely smooth part anteriorly (Fig. 55); mesoscutum glabrous; medio-posterior depression of mesoscutum small, deep, round in a shallow linear depression (Fig. 55); scutellar sulcus finely crenulate; scutellum smooth and flat; dorsal surface of propodeum largely smooth except for a medio-longitudinal carina and a moderately developed transverse carina behind it, posteriorly areolate (Fig. 56).

**Figures 53–62.**
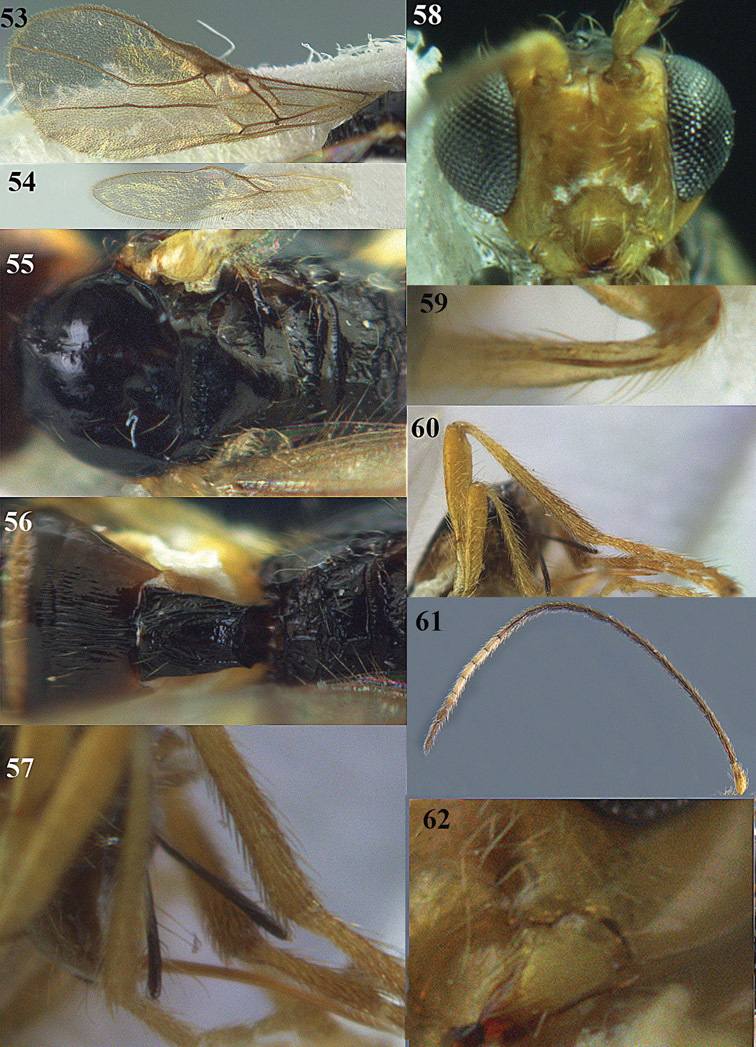
*Areotetes striatiferus* sp. n., female, holotype. **53** Fore wing **54** hind wing **55** mesosoma dorsal **56** propodeum and 1^st^-2^nd^ metasomal tergites dorsal **57** ovipositor sheath **58** head anterior **59** base of hind tibia, inner side **60** hind leg **61** antenna **62** mandible.

*Wings*. Fore wing (Fig. 53): pterostigma nearly elliptical; 1-R1 reaching wing apex and 1.5 times as long as pterostigma; r:3-SR:SR1 = 1:25:43; 2-SR:3-SR:r-m = 10:25:6; r widened; 1-M nearly straight; SR1 weakly curved; m-cu slightly postfurcal; cu-a slightly postfurcal and 1-CU1 widened; first subdiscal cell closed, CU1b short; apical third of M+CU1 sclerotized. Hind wing (Fig. 54): M+CU:1-M:1r-m = 21:20:9; cu-a straight; m-cu absent.

*Legs*. Length of femur, tibia and basitarsus of hind leg 3.9, 11.4 and 6.3 times as long as wide, respectively (Fig. 60); setae of hind femur long and of tibia moderately long; hind tibia with a short nearly straight carinula basally (Fig. 59).

*Metasoma*. Length of first tergite 1.4 times its apical width, its surface evenly convex medially and with few oblique costae and dorsal carinae united in its anterior 0.3 and absent behind it (Fig. 56); second tergite medially longitudinally costate striate (Fig. 56); second suture finely crenulate and not impressed (Fig. 56); third and following tergites smooth and partly desclerotized; length of setose part of ovipositor sheath 0.09 times fore wing and 0.25 times hind tibia (Figs 52, 57).

*Colour*. Blackish-brown; apical third of antenna pale (5 penultimate segments pale yellowish, 3 apical segments brownish; Fig. 61); palpi, mandible, malar space, tegulae, legs (especially coxae, rest mostly brownish-yellow), apex of third-seventh tergites and metasoma baso-ventrally pale yellowish; pedicellus brown; face, temple, orbita, scapus, side of pronotum ventrally, propleuron and area below precoxal sulcus yellowish-brown; pterostigma and veins mainly brown; wing membrane subhyaline.

*Variation*. Male paratype has length of fore wing 2.0 mm, and of body 1.7 mm; antenna with 21 segments, 1.3 times as long as fore wing and dark brown except for yellowish scapus; medio-posterior depression of mesoscutum comparatively large and elliptical.

*Molecular data*. None.

#### Distribution.

*China (Hunan).

#### Biology.

Unknown.

#### Etymology.

Name derived from “striatus” (Latin for “striated”) and “fero” (Latin for “carrying”), because of the coarsely striate second metasomal tergite.

#### Notes.

Does not run well in the key by Chen and Weng (2005) to any species of the group with a carinula of the hind tibia. It is similar to *Utetes acustratus* (Fischer, 1988) from New Guinea, but it has the length of the mesosoma 1.5 times its height (1.8 times in *Utetes acustratus*), the apex of the antenna dark brown (white or ivory); length of the eye about 5 times temple in dorsal view (twice) and vein m-cu of fore wing angled with vein 2-M (linear) and length of the hind femur about 4 times as long as wide (3 times).

### Biosteres

Foerster, 1862

http://species-id.net/wiki/Biosteres

Biosteres Foerster, 1862: 259; Fischer, 1972b: 485. Type species (by original designation): *Bracon carbonarius* Nees, 1834.
Rhinoplus Foerster, 1862: 258; Fischer 1972b: 540; Wharton 1988: 350 (synonymy). Type species (by original designation): *Rhinoplus laevigatus* Foerster, 1862 [examined].
Rhabdospilus Foerster, 1862: 259; Fischer 1972b: 485. Type species (by original designation): *Opius placidus* Haliday, 1837 [examined].
Zetetes Foerster, 1862: 258; Fischer 1972b: 486. Type species (by original designation): *Zetetes ultor* Foerster, 1862 [examined].
Stenospilus Foerster, 1862: 259. Type species (by original designation): *Stenospilus vagator* Foerster, 1862 [= *Opius bicolor* Wesmael, 1835; examined].
Opiellus Ashmead, 1900c: 368; Fischer 1972b: 486 (new name for *Zetetus*). Type species (by implication): *Zetetes ultor* Foerster, 1862.
Celiestiella Cameron, 1903: 343; Fischer 1972b: 486. Type species (by monotypy): *Celiestiella testaceipes* Cameron, 1903 [examined].

#### Diagnosis.

Hypoclypeal depression absent or narrow, and medially ventral margin of clypeus near upper level of condyli of mandibles (“mouth closed”); clypeus comparatively sparsely and short setose, if rather long and dense then clypeus flattened; mandible with ventro-basal carina, rarely obsolescent or on a small protuberance (resembling a small tooth); epistomal suture present; if suture is shallow then basal half of vein M+CU1 of fore wing largely unsclerotized; vein 3-SR of fore wing 1.3 times vein 2-SR or less, if rarely 1.4-1.5 times then pronope very large or pterostigma triangular; mesosternum normal, much longer than fore coxa; hind coxae normal, rounded ventrally; second-fourth tarsal segments comparatively slender; telotarsus and arolium not enlarged; dorsope usually large and close to lateral margin of first tergite; hypopygium of female at most slightly incised.

#### Biology.

Parasitoids of Anthomyiidae and Scathophagidae.

#### Distribution.

Holarctic, Neotropical, Oriental, Australian.

### Biosteres pavititus

Chen & Weng, 2005

http://species-id.net/wiki/Biosteres_pavititus

Biosteres pavitita Chen & Weng, 2005: 31, 185.

#### Distribution.

Oriental China: Hubei, Hunan.

#### Notes.

One female from Hunan is very similar to *Biosteres carbonarius*(Nees, 1834) with vein r of fore wing about as long as width of pterostigma and much longer than wide; vein 2-M of fore wing 1.9-2.2 times vein 3-SR; second metasomal tergite black, at most anteriorly brownish and first tergite finely and densely sculptured medially.

### Genus Coleopioides

van Achterberg & Li gen. n.

http://species-id.net/wiki/Coleopioides

**Figure 63.**
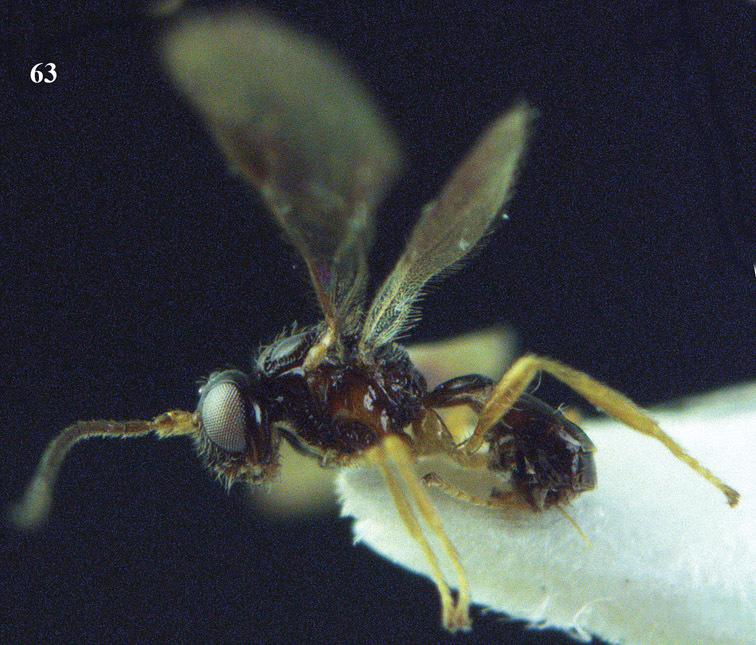
*Coleopioides diversinotum* sp. n., female, holotype. Habitus lateral.

Figs 63 [Fig F24] [Fig F25] [Fig F26] –84

#### Type species.

*Coleopioides postpectalis* sp. n.

#### Etymology.

From the generic name “*Coleopius*” and the addition “oides”, because it is superficially similar to the genus *Coleopius* Fischer.

#### Diagnosis.

**Figures 64–72. F24:**
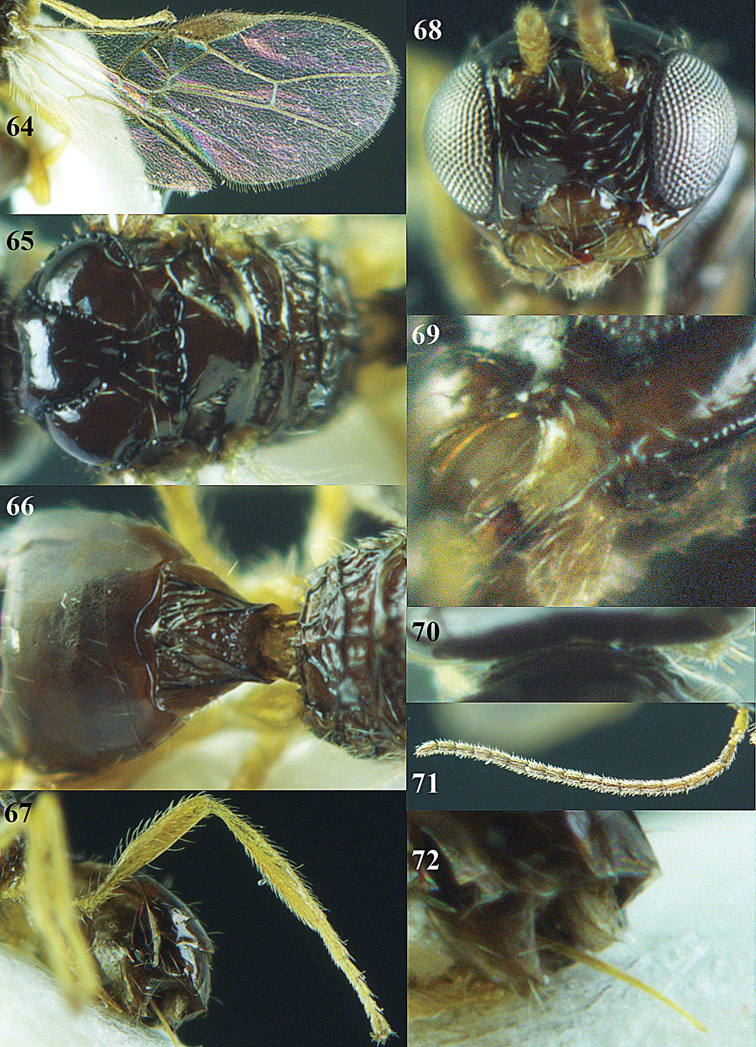
*Coleopioides diversinotum* sp. n., female, holotype. **64** Wings **65** mesosoma dorsal **66** propodeum and 1^st^-2^nd^ metasomal tergites dorsal **67** hind leg **68** head anterior **69** mandible **70** pronope dorsal **71** antenna **72** ovipositor.

**Figure 73. F25:**
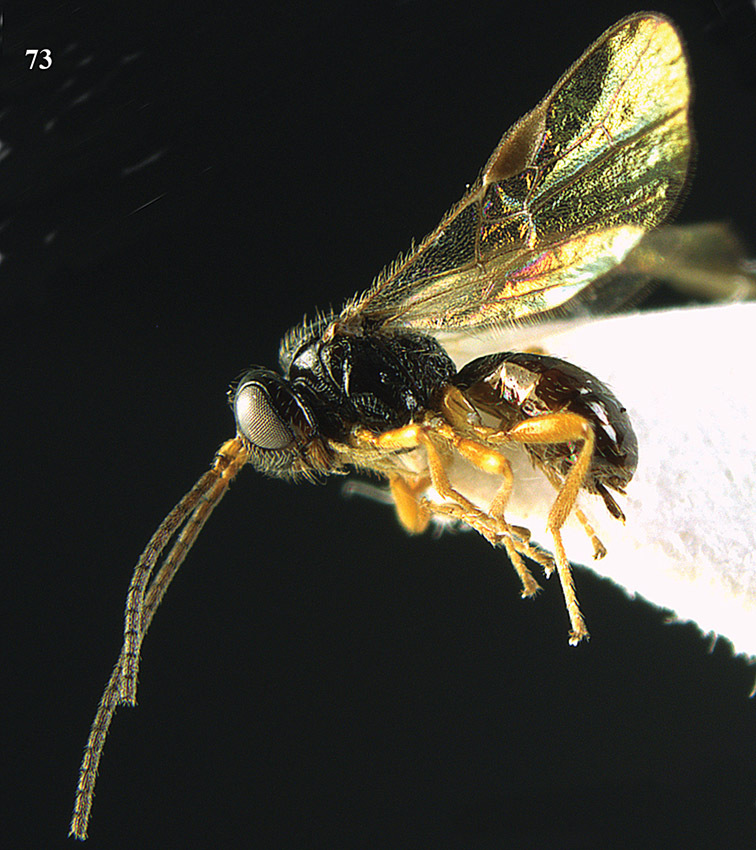
*Coleopioides postpectalis* sp. n., female, holotype. Habitus lateral.

**Figures 74–81. F26:**
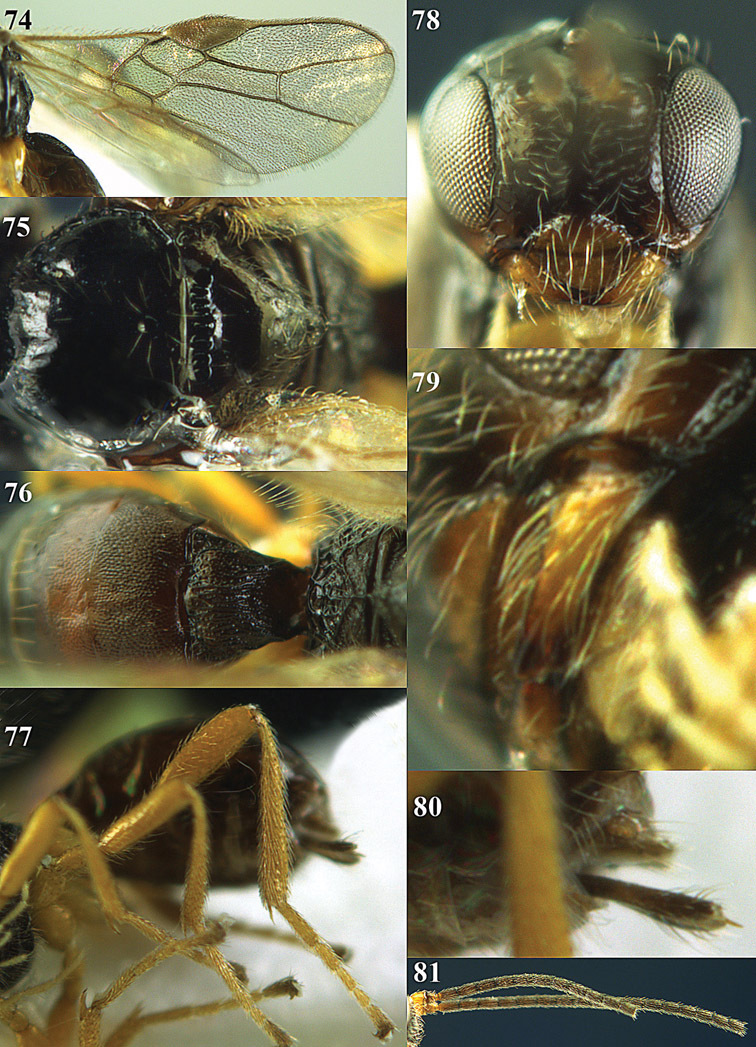
*Coleopioides postpectalis* sp. n., female, holotype. **74** Wings **75** mesosoma dorsal **76** propodeum and 1^st^-2^nd^ metasomal tergites dorsal **77** hind leg **78** head anterior **79** mandible **80** ovipositor sheath **81** antennae.

Face without tubercles; scapus, fore coxa and trochanter at most weakly compressed; epistomal suture without large depressions; inner sides of antennal sockets normal, not protruding; labrum exposed; clypeus truncate ventrally and hypoclypeal depression present (Figs 68, 78); mandibles long and slender (Fig. 79); scutellar sulcus usually rather wide (Figs 65, 75); at least part of postpectal carina present medio-ventrally, but absent in *Coleopioides diversinotum*; notauli complete (Fig. 65) or largely absent (Fig. 75); mesoscutum with medio-posterior depression (Fig. 75); propodeum with a transverse carina subbasally and with long medio-longitudinal carina (Figs 65, 76); precoxal sulcus wide and crenulate (Figs 63, 73); second submarginal cell elongate, vein 3-SR 1.5–1.8 times as long as vein 2-SR (Figs 64, 74); second and third metasomal tergites more or less enlarged, longer than following segments; dorsope absent; laterope distinct; second and third tergites enlarged, longer than following segments (Figs 66, 76); second metasomal suture absent or superficially impressed and smooth; third tergite more or less sculptured (but may be largely smooth); epipleuron of third metasomal tergite slightly differentiated from notum and without lateral crease; fourth metasomal tergite well exposed.

#### Biology.

Unknown.

**Notes.** Both new species are similar to *Coleopius* Fischer because of the presence of the hypoclypeal depression, the short metasoma and the shape of the clypeus. The long second submarginal cell (vein 3-SR 1.5-1.8 times vein 2-SR; shorter in *Coleopius*), the similar third epipleuron and notum (epipleuron distinctly less sclerotized in *Coleopius*), and the third tergite about as long as second tergite and without sharp lateral crease (lateral crease present in *Coleopius*) indicate that they do not belong in *Coleopius*. The new species differ from the similar genus *Bitomus* Szépligeti by the same character states, except that the second submarginal cell is intermediate. The new genus *Coleopioides* belongs to a separate basal group in the tribe Opiini together with *Areotetes* gen. n., according to the molecular data of the nuclear 28S marker of both species. *Areotetes* gen. n. shares the medio-longitudinal carina and the areolate posterior part of the propodeum with *Coleopioides*, but differs by having the carinula of the hind tibia, the crenulate posterior groove of the pronotum and the absence of the malar suture and of the depressions behind the antennal sockets.

### Coleopioides diversinotum

Li & van Achterberg sp. n.

urn:lsid:zoobank.org:act:4071C1E7-C2EE-4F05-9E27-CC0BF4D1A1CB

http://species-id.net/wiki/Coleopioides_diversinotum

Figs 63 –72

#### Type material.

Holotype, ♀ (ZUH), “S. China: Hunan, nr Zhangjiajie, Badagong Mts, Tian Ping Mt., 9–13.VII.2009, 550 m, Xi-Ying Li, RMNH’10”, “CVA4244, sp. 10”.

#### Diagnosis.

Mandible without ventral carina; ventral rim of clypeus narrow and upcurved (Fig. 68); clypeus medium-sized; face rather tuberculate medio-ventrally (Fig. 68); pronotum without distinct pronope (Fig. 70); propleuron smooth subposteriorly; pronotum short; notauli nearly complete and finely crenulate (Fig. 65); precoxal sulcus present anteriorly and medially, wide and crenulate (Fig. 63); postpectal carina completely absent; propodeum with a transverse carina subbasally (Fig. 65); first discal cell of fore wing less transverse than in *Coleopioides postpectalis* (Fig. 64); vein m-cu of fore wing twice longer than vein 2-SR+M; second tergite smooth (Fig. 66).

#### Description.

Holotype, ♀, length of body 1.6 mm, of fore wing 2.0 mm.

*Head*. Antenna with 19 segments and 0.8 times as long as fore wing; length of third segment 1.2 times fourth segment, length of third, fourth and penultimate segments 3.0, 2.5, and 2.2 times their width, respectively (Fig. 71); length of maxillary palp 0.9 times height of head; labial palp segments short; occipital carina rather close to hypostomal carina and dorsally and behind upper half of eye absent (Fig. 69); no depression behind stemmaticum; hypostomal carina medium-sized; length of eye in dorsal view 2.6 times temple; frons medially convex, depressed behind antennal sockets and laterally convex, entirely glabrous; face smooth, evenly convex; width of cly-peus twice its maximum height and 0.55 times width of face, clypeus convex, smooth and its ventral margin differentiated, narrow, thin, straight and upcurved (Fig. 68); hypoclypeal depression rather large (Fig. 69); malar suture present; mandible normal, slightly convex and without ventral carina (Fig. 69).

*Mesosoma*. Length of mesosoma 1.3 times its height; dorsal pronope absent (Fig. 70); pronotal side smooth, but distinctly crenulate posteriorly and a few crenulae anteriorly, no ventral oblique carina (Fig. 63); epicnemial area largely smooth dorsally, with few weak crenulae; precoxal sulcus anteriorly and medially present, wide, coarsely rugose-crenulate (Fig. 63); rest of mesopleuron smooth; pleural sulcus smooth, except for some indistinct crenulae ventrally; mesosternal sulcus rather wide and with few crenulae, without postpectal carina; notauli nearly complete, narrow and finely crenulate (Fig. 65); mesoscutum smooth and with some setae medially; medio-posterior depression of mesoscutum round and small (Fig. 65); lateral margin of mesoscutum crenulate; scutellar sulcus widely and coarsely crenulate (Fig. 65); scutellum smooth and slightly convex; dorsal surface of propodeum short and with short medio-longitudinal carina, transverse carina strong, posterior surface largely smooth, with median and sublateral carinae (Fig. 65).

*Wings*. Fore wing (Fig. 64): pterostigma wide elliptical; 1-R1 reaching wing apex and 1.5 times as long as pterostigma; r:3-SR:SR1 = 2:23:37; 2-SR:3-SR:r-m = 15:23:7; r short and slender; 1-M nearly straight; SR1 slightly curved; m-cu postfurcal by half its length; cu-a postfurcal and 1-CU1 widened; first subdiscal cell closed, CU1b short; apical quarter of M+CU1 sclerotized; first discal cell of fore wing comparatively short. Hind wing (Fig. 64): M+CU:1-M:1r-m = 18:12:7; cu-a slightly reclivous; m-cu absent; 1-1A curved.

*Legs*. Length of femur, tibia and basitarsus of hind leg 4.8, 7.8 and 3.7 times as long as wide, respectively; hind femur and tibia with moderately long setae (Fig. 67).

*Metasoma*. Length of first tergite equal to its apical width, its surface strongly convex gradually medially, coarsely rugose and with dorsal carinae united near apical fifth of tergite (Fig. 66); second suture absent; second and following tergites smooth; second and third tergites 0.6 times as long as metasoma after first tergite; length of setose part of ovipositor sheath 0.05 times fore wing and 0.2 times hind tibia (Figs 63, 72).

*Colour*. Dark brown; mandible, palpi, scapus, tegulae and legs (but telotarsi darkened) pale yellowish; clypeus, malar space and mesopleuron largely yellowish-brown; pterostigma and veins brown; wing membrane slightly infuscate below base of pterostigma, remainder subhyaline.

*Molecular data*. COI, 16S, 28S (CVA 4244).

#### Distribution.

*China (Hunan).

#### Biology.

Unknown.

#### Etymology.

Name derived from “diversus” (Latin for “different”) and “notum”, because of the differently sculptured mesoscutum.

#### Notes.

The new species runs in the key by Chen and Weng (2005) to *Xynobius indagatrix* (Weng & Chen, 2005) comb. n.; *Coleopioides diversinotum* differs by lacking a dorsope (present in *Xynobius indagatrix*), the length of the eye in dorsal view 2.6 times temple (4.2 times in *Xynobius indagatrix*), length of the first tergite about equal to its apical width (1.3 times) and the propodeum with strong transverse carina and carinate (densely rugose and without distinct transverse carina).

### Coleopioides postpectalis

Li & van Achterberg sp. n.

urn:lsid:zoobank.org:act:2FBC0E1E-FCC4-4FE7-86A7-A59D04B47C5C

http://species-id.net/wiki/Coleopioides_postpectalis

Figs 73 [Fig F26] –84

#### Type material.

Holotype, ♀ (ZUH), “N. China: Shandong, Anqiu, Suotou Mt., 31.VII.2009, c. 120 m, Li Xi-Ying, RMNH’09”. Paratypes (RMNH): 3 ♀ + 1 ♂ with same label-data; 1 ♂, “S. China: Hunan, nr Suining, Huangsang N.R., Shaoyang, 12–13.VI.2009, 1000 m, Xi-Ying Li, RMNH’09”.

#### Diagnosis.

Mandible with long fine ventral carina (Fig. 84); rim of clypeus pointed downwards (Fig. 78); pronotum and mesopleuron dorsally superficially granulate;occipital carina finely crenulate; clypeus comparatively wide (Fig. 78); tegulae dark brown; propleuron rugulose subposteriorly; notauli only anteriorly distinctly impressed; precoxal sulcus wide and crenulate (Fig. 73); postpectal carina medio-ventrally coarsely developed; first discal cell of fore wing comparatively transverse (Fig. 74); vein m-cu of fore wing 5 times longer than vein 2-SR+M (Fig. 74); second metasomal tergite of ♀ granulate (Fig. 76).

#### Description.

Holotype, ♀, length of body 2.0 mm, of fore wing 2.1 mm.

*Head*. Antenna with 19 segments (incomplete; in paratypes 1.3 times as long as fore wing); length of third segment 1.2 times fourth segment, length of third and fourth penultimate segments 2.3 and 2.0 times their width, respectively (Fig. 81); length of maxillary palp equal to height of head; labial palp segments slender; occipital carina finely crenulate (Fig. 84), rather close to hypostomal carina, absent dorsally and behind upper third of eye; with distinct groove behind stemmaticum; hypostomal carina medium-sized; length of eye in dorsal view 3.0 times temple; frons medially narrowly convex between depressions behind antennal sockets, with depression between antennal sockets and laterally slightly convex, glabrous; face sparsely finely punctate, evenly convex (Fig. 78); width of clypeus 2.8 times its maximum height and 0.7 times width of face, clypeus rather flat, smooth except for some punctures and its ventral margin slightly differentiated, thin, straight and pointed downwards; hypoclypeal depression rather large (Fig. 78); malar suture present; mandible normal, slightly convex and sparsely punctate and with fine ventral carina (Fig. 79).

*Mesosoma*. Length of mesosoma 1.2 times its height; pronope absent (Fig. 83); pronotal side smooth, but distinctly crenulate posteriorly, superficially granulate dorsally and oblique groove finely crenulate, no ventral oblique carina (Fig. 73); propleuron rugulose subposteriorly (Fig. 73); epicnemial area largely smooth dorsally, with few crenulae anteriorly; precoxal sulcus anteriorly and medially present, wide, coarsely crenulate (Fig. 73); rest of mesopleuron smooth; pleural sulcus indistinctly crenulate dorsally and distinctly crenulate ventrally; mesosternal sulcus as a row of punctures, with strong postpectal carina medio-ventrally; notauli only anteriorly widely impressed (Fig. 75); mesoscutum smooth and only posteriorly with few setae; medio-posterior depression of mesoscutum round and small (Fig. 83); lateral margin of mesoscutum crenulate; scutellar sulcus wide and coarsely crenulate (Fig. 75); scutellum smooth and slightly convex; dorsal surface of propodeum not differentiated and with long medio-longitudinal carina, transverse carina distinct, posterior surface largely reticulate-rugose, with sublateral carinae (Fig. 76).

*Wings*. Fore wing (Fig. 74): pterostigma triangular; 1-R1 reaching wing apex and 1.7 times as long as pterostigma; r:3-SR:SR1 = 3:27:42; 2-SR:3-SR:r-m = 15:27:12; r short and slender; 1-M and SR1 nearly straight; m-cu postfurcal by fifth of its length; cu-a subinterstitial and 1-CU1 hardly widened; first subdiscal cell closed, CU1b short; apical quarter of M+CU1 sclerotized; first subdiscal cell of fore wing moderately long. Hind wing (Fig. 74): M+CU:1-M:1r-m = 5:4:2; cu-a slightly reclivous; m-cu absent; 1-1A curved.

*Legs*. Length of femur, tibia and basitarsus of hind leg 3.2, 8.0 and 4.0 times as long as wide, respectively; hind femur and tibia with long setae (Fig. 77).

*Metasoma*. Length of first tergite 0.9 times its apical width, its surface evenly gradually convex medially, longitudinally rugose and punctate posteriorly and with dorsal carinae separated and up to middle of tergite (Fig. 76); second suture obsolescent; second tergite as long as third tergite (Fig. 73) and densely and finely granulate-punctate with some fine striae (Fig. 76), its epipleuron slightly less sclerotized than notum; third tergite (except apically) superficially granulate and following tergites smooth; second and third tergites 0.7 times as long as metasoma after first tergite; length of setose part of ovipositor sheath 0.06 times fore wing and 0.2 times hind tibia (Figs 73, 80).

*Colour*. Dark brown (including tegulae and apex of antenna in paratypes); head dorsally and mesosoma black; palpi, scapus and legs (but telotarsi darkened) pale yellowish; mandible, clypeus and malar space yellowish-brown; pterostigma (but apically pale brown) and veins brown; wing membrane evenly slightly infuscate.

*Molecular data*. COI, 16S, 28S (CVA4255).

*Variation*. Length of body 1.7–2.0 mm, of fore wing 1.9–2.1 mm; antennal segments of ♀ 24 (1), 25 (1) or 26 (1) and of ♂ 24 (2); ventral rim somewhat upcurved or pointed downwards; pronotal side and second tergite dark brown or yellowish-brown; third tergite of male only superficially granulate, second tergite largely sculptured as in female or superficially granulate.

**Figures 82–84.**
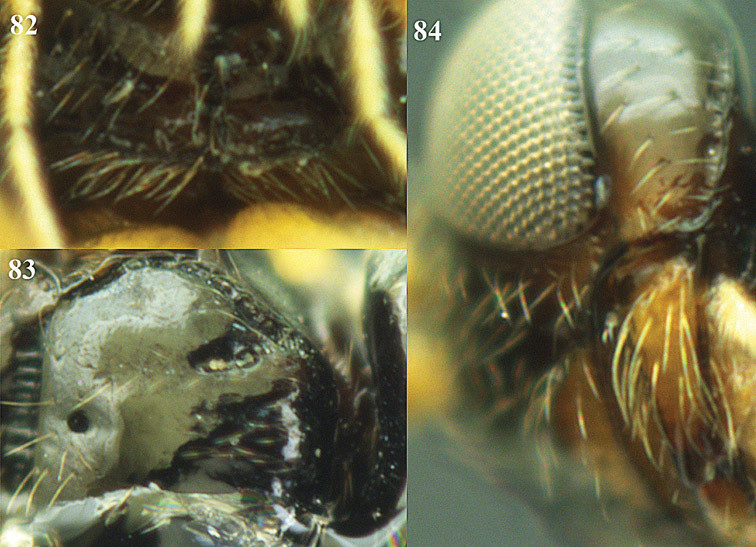
*Coleopioides postpectalis* sp. n., female, holotype. **82** Postpectal carina **83** mesoscutum dorsal **84** head lateral

#### Distribution.

*China (Shandong, Hunan).

#### Biology.

Unknown.

#### Etymology.

Name derived from “postpectus” (Latin for “posterior part of breast”), because of the presence of the postpectal carina.

#### Notes.

The new species does not run well in the key by Chen and Weng (2005) because it has the second tergite granulate (the choices in the key are longitudinally striate or smooth), but if it is assumed that the sculpture is superficial and the tergite is nearly smooth then it runs to *Xynobius indagatrix* (Weng & Chen, 2005) comb. n.; *Coleopioides postpectalis* differs by having the length of the eye in dorsal view 3 times temple (4.2 times in *Xynobius indagatrix*), no dorsope (present), length of the hind femur about 3 times its width (4.7 times), length of first tergite 0.9 times its apical width (1.3 times), the second tergite granulate-punctate with some fine striae (smooth) and the propodeum with a median carina and areolate (densely rugose and without carina).

### Diachasmimorpha

Viereck, 1913

http://species-id.net/wiki/Diachasmimorpha\according_to_Li_et_al_2013

? Trigonospilus Ashmead, 1900a: 134. Type species (by original designation): *Trigonospilus hopkinsi* Ashmead, 1900 [type lost?].
Diachasmimorpha Viereck, 1913b: 641. Type species (by original designation): *Diachasmimorpha comperei* Viereck, 1913 [? = *Diachasmimorpha albobalteata* (Cameron, 1912)].
Parasteres Fischer, 1967: 3. Type species (by original designation): *Biosteres acidusae* Fischer, 1967 (= *Opius tryoni* Cameron, 1911).

#### Diagnosis.

Face without tubercles; antenna with 35–67 segments; clypeus evenly curved ventrally, at most widely triangularly protruding, usually narrower, or longer, not impressed; no distinct hypoclypeal depression, at most with a narrow slit; mandible usually comparatively short and wide; occipital carina near level of middle of eye straight or nearly so, without transverse carina or crest; clypeus more or less convex and comparatively high; scapus, fore coxa and trochanter at most weakly compressed; epistomal suture without large depressions; scutellar sulcus usually rather wide; postpectal carina completely absent; vein 2-SR of fore wing present, rarely absent; first subdiscal cell of fore wing at least partly closed by vein 3-CU1 postero-apically; vein cu-a of hind wing nearly always present; vein 3-SR of fore wing shorter than vein 2-SR; if subequal then vein m-cu of hind wing present as a weakly pigmented trace and precoxal sulcus absent; vein 1-M of fore wing more or less curved posteriorly, but sometimes nearly straight; vein m-cu of fore wing slightly postfurcal; length of fore wing usually more than 3 mm; fourth tergite (at least partly) exposed; third tergite (largely) smooth; ovipositor long, usually as long as fore wing or longer.

#### Biology.

Parasitoids of Tephritidae.

#### Distribution.

Cosmopolitan, but no species known from Northwest Europe.

### Diachasmimorpha longicaudata

(Ashmead, 1905)

http://species-id.net/wiki/Diachasmimorpha_longicaudata

Biosteres longicaudatus Ashmead, 1905: 970.

#### Biology.

Polyphagous parasitoid of Tephritidae, including *Bactrocera*, *Carponmya*, *Dacus*, *Myopites*, *Philophylla*, *Procecidochares*, *Rhagoletis* and *Toxotrypana* spp.

#### Distribution.

Oriental and introduced in southern U.S.A., Neotropics, Pacific, southern Europe and Australia for biocontrol.

### Genus Fopius

Wharton, 1987

http://species-id.net/wiki/Fopius

Figs 85 –94

Fopius Wharton, 1987: 68-69(as subgenus of *Rhynchosteres* Fischer, 1965), 1988: 353, 1997: 16–19 (genus rank), 1999: 50 (review with matrix of character states); van Achterberg and Maetô 1990: 60 (genus rank). Type species (by original designation): *Rhynchosteres (Fopius) silvestrii* Wharton, 1987 [examined].

#### Diagnosis.

**Figure 85.**
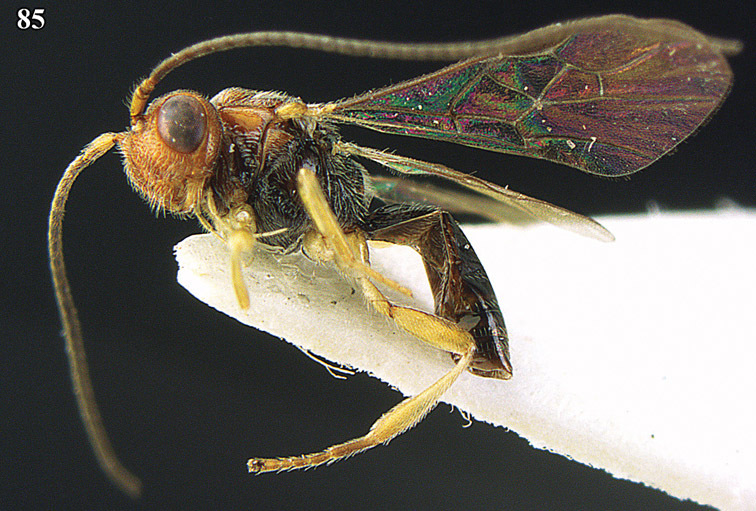
*Fopius dorsopiferus* sp. n., male, holotype. Habitus lateral.

**Figures 86–94.**
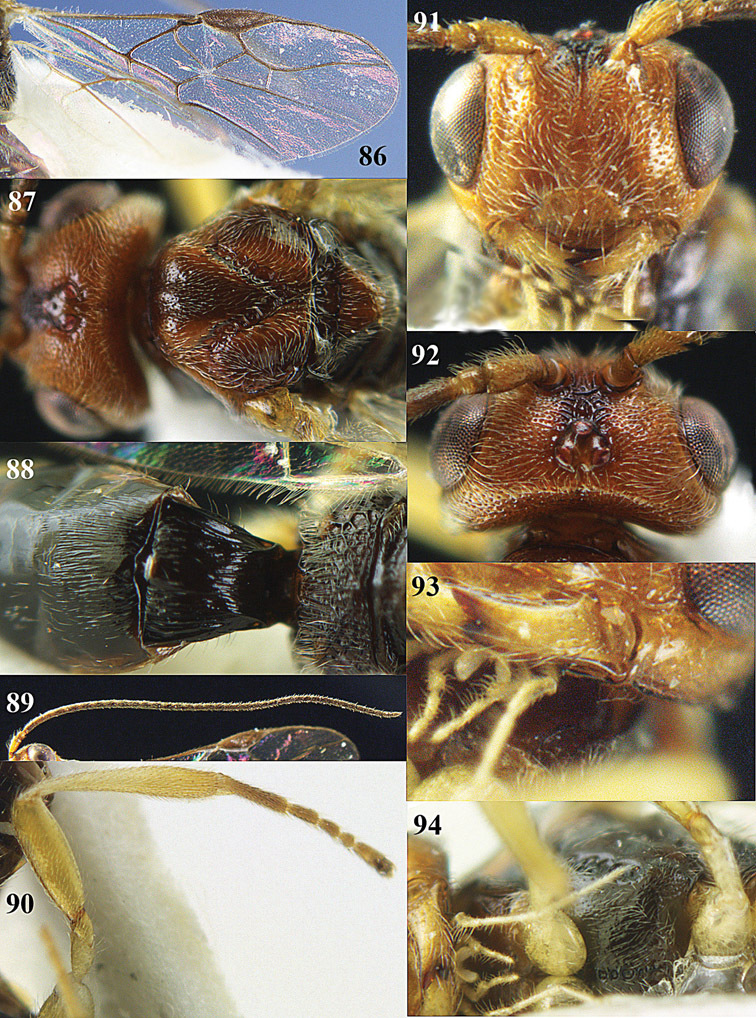
*Fopius dorsopiferus* sp. n., male, holotype. **86** Wings **87** head and mesosoma dorsal **88** propodeum and 1^st^-2^nd^ metasomal tergites dorsal **89** antenna **90** hind leg **91** head anterior **92** head dorsal **93** mandible **94** mesosternum ventral.

Face without tubercles; propleuron with short subapical carina; clypeus more or less convex ventrally and comparatively high, without distinct hypoclypeal depression, at most with a narrow slit (Fig. 91); mandible robust (Fig. 93); occipital carina near level of middle of eye straight or nearly so, without transverse carina or crest; scapus, fore coxa and trochanter at most weakly compressed; epistomal suture without large depressions; scutellar sulcus wide (Fig. 85); postpectal carina more or less developed medio-ventrally; vein 2-SR of fore wing present; first subdiscal cell of fore wing closed by vein 3-CU1 and short vein CU1b postero-apically (Fig. 86); vein cu-a of hind wing present (Fig. 86); vein 3-SR of fore wing shorter than vein 2-SR; vein m-cu of hind wing present as a weakly pigmented trace (Fig. 86); precoxal sulcus present; vein m-cu of fore wing antefurcal or interstitial; length of fore wing usually more than 3 mm; dorsope of first tergite more or less present (Fig. 86); fourth tergite exposed; third tergite (largely) smooth; ovipositor long, usually as long as fore wing or longer.

#### Biology.

Parasitoids of Tephritidae, at least partly ovo-larval (Wharton 1997).

### Fopius dorsopiferus

Li, van Achterberg & Tan sp. n.

urn:lsid:zoobank.org:act:BB415FA4-5FE0-4F47-98C8-F14290E7D0AE

http://species-id.net/wiki/Fopius_dorsopiferus

Figs 85 –94

#### Type material.

Holotype, ♂ (ZUH), “S. China: Hunan, nr Chengbu, Nan Mt., Shaoyang, 1500 m, 10–11.VI.2009, Xi-Ying Li, RMNH’09”, “CVA 4246, sp. 12”.

#### Diagnosis.

Oblique carina of propleuron present (Fig. 85); postpectal carina coarsely developed medio-ventrally (Fig. 85); length of fore wing more than 3 mm; vein 3-SR of fore wing shorter than vein 2-SR (Fig. 86); dorsope of first tergite present (Fig. 88).

#### Description.

Holotype, ♂, length of body 3.6 mm, of fore wing 3.2 mm.

*Head*. Antenna with 39 segments and 1.8 times as long as fore wing; length of third segment 1.1 times fourth segment, length of third, fourth and penultimate segments 3.2, 2.8, and 2.3 times their width, respectively (Fig. 89); length of maxillary palp equal to height of head; labial palp segments slender (Fig. 94); occipital carina far from hypostomal carina and dorsally absent; median pit behind stemmaticum present (Fig. 91); hypostomal carina narrow; length of eye in dorsal view 2.5 times temple; frons medially flat, rugose and depressed near antennal sockets, rest setose, densely and coarsely punctate and slightly convex (Fig. 92); face largely coarsely punctate, medially indistinctly elevated (Fig. 91); width of clypeus 2.1 times its maximum height and 0.6 times width of face, clypeus flattened, largely smooth and its ventral margin differentiated, wide, thin and slightly curved (Fig. 91); hypoclypeal depression nearly absent (Fig. 91); malar suture present; mandible large, punctate, without ventral carina (Fig. 93).

*Mesosoma*. Length of mesosoma 1.3 times its height; dorsal pronope absent; pronotal side smooth dorsally and ventrally, remainder largely costate crenulate, no ventral oblique carina (Fig. 85); epicnemial area largely smooth dorsally, with few weak crenulae; precoxal sulcus only medially distinctly impressed, wide, coarsely rugose-crenulate (Fig. 85); rest of mesopleuron smooth; pleural sulcus smooth, except for some indistinct crenulae ventrally; mesosternal sulcus hardly impressed but row of coarse punctures and posteriorly with strongly developed postpectal carina; notauli complete, deep and widely crenulate (Fig. 87); middle lobe with pair of longitudinal depressions, lobes largely densely setose and finely punctate (Fig. 87); medio-posterior depression of mesoscutum absent; scutellar sulcus widely crenulate; scutellum sparsely punctate and slightly convex; dorsal surface of propodeum narrow and with short medio-longitudinal carina, surface rather coarsely reticulate-rugose (Fig. 88).

*Wings*. Fore wing (Fig. 86): pterostigma wide elliptical; 1-R1 not reaching wing apex and 1.5 times as long as pterostigma; r:3-SR:SR1 = 10:18:73; 2-SR:3-SR:r-m = 25:18:13; r long and slender; 1-M nearly straight; SR1 sinuate; m-cu interstitial; cu-a slightly postfurcal and 1-CU1 widened; first subdiscal cell closed, CU1b rather short; M+CU1 entirely sclerotized. Hind wing (Fig. 86): M+CU:1-M:1r-m = 30:23:14; cu-a straight; m-cu long; subbasal cell largely glabrous.

*Legs*. Length of femur, tibia and basitarsus of hind leg 3.2, 7.0 and 4.2 times as long as wide, respectively; hind femur and tibia with moderately long setae (Fig. 90).

*Metasoma*. Length of first tergite 1.0 times its apical width, dorsope small, its surface rather flat, longitudinally striate (but basally smooth) and with dorsal carinae separated and up to basal 0.6 of tergite (Fig. 88); second tergite largely longitudinally aciculate (Fig. 88); second suture absent; third and following tergites smooth.

*Colour*. Yellowish-brown; mandible, palpi, tegulae and legs (but hind tarsus more or less dark brown) pale yellowish; antenna (except scapus), mesosoma (except mesoscutum, scutellum, pronotum dorsally and mesopleuron antero-dorsally), metasoma, pterostigma and veins dark brown; wing membrane slightly infuscate.

*Molecular data*. COI, 16S, 28S (CVA 4246).

#### Distribution.

*China (Hunan).

#### Biology.

Unknown.

#### Etymology.

Name derived from “dorsope” (morphological term for the dorso-basal depression of first tergite) and “fero” (Latin for “carrying”), because of the distinctly impressed dorsope.

#### Notes.

The new species runs in the key by Chen and Weng (2005) to *Fopius vandenboschi* (Fullaway, 1952). *Fopius dorsopiferus* differs by having the length of the hind femur about 3 times its width (5 times in *Fopius vandenboschi*), length of first tergite equal to its apical width (0.8 times), the postpectal carina strongly developed (obsolescent or absent) and the first tergite with small dorsope (absent).

### Indiopius

Fischer, 1966

http://species-id.net/wiki/Indiopius

Figs 95 –104

Indiopius Fischer, 1966: 154–155. Type species (by original designation): *Indiopius humillimus* Fischer, 1966 [examined].

#### Diagnosis.

Marginal cell of fore wing open apically and long (Fig. 96); veins m-cu and r-m of fore wing absent (Fig. 96); vein 2-SR of fore wing absent (Fig. 96); first subdiscal cell of fore wing open postero-apically; vein cu-a of hind wing absent (Fig. 96); clypeus wide, short and impressed (Fig. 101); mandible long and slender (Fig. 102); occipital carina entirely absent; first-third metasomal tergites more or less coriaceous or rugulose (Fig. 98); fourth-six tergites largely retracted.

#### Biology.

Unknown.

### Indiopius chenae

van Achterberg & Li sp. n.

urn:lsid:zoobank.org:act:7576A822-B223-432D-A033-AAC11863C820

http://species-id.net/wiki/Indiopius_chenae

Figs 95 –104

#### Material.

Holotype, ♀ (ZUH), “China: Hunan, Changde, Taoyuan, Mao-zong-ling Xiang, Xian-feng, Ri-tou-wang, at light, CN 1032, 11.viii.2010, P.-P. Chen, RMNH’11”.

#### Diagnosis.

Frons without elongate depression or punctures; antenna of ♀ with about 19 segments; occipital carina absent, at most with short ventral part present; head dorsally dark brown and mesoscutum chestnut-brown; scutellar sulcus wide (Fig. 97); vein 2-1A of fore wing pigmented; vein cu-a of fore wing postfurcal by its width or interstitial; vein 1-R1 of fore wing 1.0-1.3 times as long as pterostigma and vein 1-R1 of fore wing 1-4 times as long as distance between its apex and apex of fore wing; posterior margin of pterostigma straight; vein 1-SR present; vein 3-SR+SR1 pointing to apex of fore wing (Fig. 96); hind femur wider than middle femur (Fig. 99); fore femur about as wide as middle femur (Figs 99, 100); first tergite granulate or rugulose and about as long as wide apically.

#### Description.

Holotype, ♀, length of body 1.3 mm, of fore wing 1.5 mm.

*Head*. Antenna with 19 segments and 0.9 times as long as fore wing; length of third segment 1.4 times fourth segment, length of third, fourth and penultimate segments 3.4, 2.2 and 2.7 times their width, respectively (Fig. 103); length of maxillary palp 0.6 times height of head; labial palp segments slender (Fig. 101); short part of occipital carina far from hypostomal carina (Fig. 102), remainder largely absent; median depression behind stemmaticum absent; hypostomal carina narrow; length of eye in dorsal view 1.7 times temple (Fig. 97); frons medially with small pit, smooth and depressed near antennal sockets, glabrous (Fig. 97); face smooth except for some setiferous punctures (Fig. 101); width of clypeus 3.3 times its maximum height and 0.7 times width of face, clypeus flattened, largely smooth and its ventral margin not differentiated, thin and flat (Fig. 102); hypoclypeal depression medium-sized (Fig. 101); malar suture present as wide depression (Fig. 102); mandible large, hardly twisted, smooth and with narrow ventral carina (Fig. 102).

**Figure 95.**
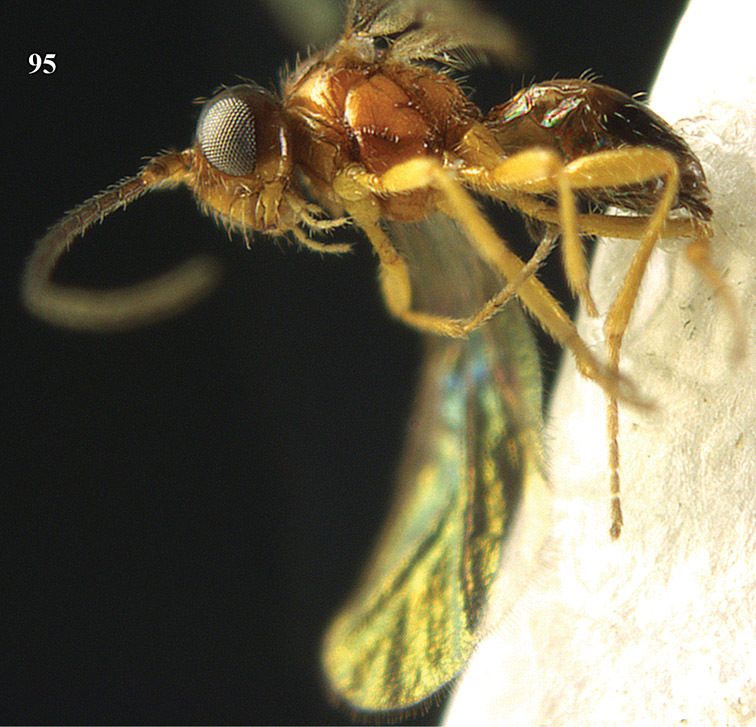
*Indiopius chenae* sp. n., female, holotype. Habitus lateral.

*Mesosoma*. Length of mesosoma 1.2 times its height; dorsal pronope absent; pronotal side smooth, no ventral oblique carina (Fig. 95); epicnemial area smooth; precoxal sulcus only medially and anteriorly impressed, narrow and distinctly crenulate (Fig. 95); rest of mesopleuron smooth; pleural sulcus smooth, except for some indistinct crenulae ventrally; mesosternal sulcus impressed, crenulate and posteriorly without postpectal carina, but with short carina above base of middle coxa; notauli absent on disc, with a pair of short crenulate impressions anteriorly (Fig. 97); middle lobe with a shallow longitudinal depression anteriorly, lobes smooth and glabrous; medio-posterior depression of mesoscutum absent; scutellar sulcus moderately wide and distinctly crenulate, but narrowed laterally (Fig. 97); scutellum smooth and slightly convex, wide posteriorly (Fig. 97); surface of propodeum oblique and without medio-longitudinal carina, surface smooth, except some crenulae posteriorly (Fig. 97).

**Figures 96–104.**
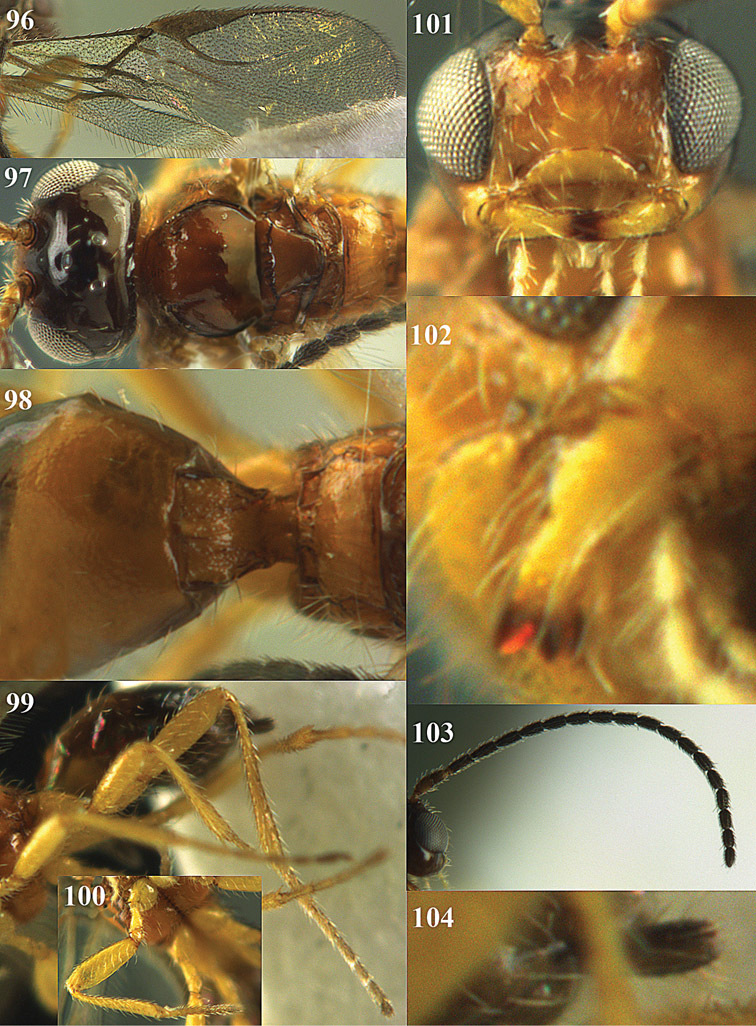
*Indiopius chenae* sp. n., female, holotype. **96** Wings **97** head and mesosoma dorsal **98** propodeum and 1^st^-2^nd^ metasomal tergites dorsal **99** hind leg **100** fore leg **101** head anterior **102** mandible **103** antenna **104** ovipositor sheath.

*Wings*. Fore wing (Fig. 96): pterostigma nearly triangular; 1-R1 not reaching wing apex and as long as pterostigma; veins r, 3-SR and SR1 not differentiated; 1-M straight; cu-a slightly postfurcal and 1-CU1 widened; first subdiscal cell open, CU1b absent; M+CU1 only apically sclerotized. Hind wing (Fig. 96): narrow, cu-a absent.

*Legs*. Length of femur, tibia and basitarsus of hind leg 3.3, 9.0 and 6.0 times as long as wide, respectively; hind femur with long setae ventrally (Fig. 99).

*Metasoma*. Length of first tergite 1.1 times its apical width, dorsope absent, its surface rather flat, longitudinally finely rugulose (but basally smooth) and with dorsal carinae separated and up to apex of tergite (Fig. 98); second tergite largely superficially granulate and shiny (Fig. 98); second suture absent; third and following tergites smooth; setose part of ovipositor sheath short (Fig. 104), 0.04 times as long as fore wing.

*Colour*. Yellowish-brown; mandible, scapus ventrally, clypeus, palpi and legs (but telotarsi more or less infuscate) yellow; antenna (except scapus and pedicellus), head dorsally, laterally and posteriorly, and apical half of metasoma dark brown; tegulae, pterostigma and veins brown; mesoscutum and scutellum chestnut-brown; wing membrane slightly infuscate.

*Molecular data*. None.

#### Distribution.

*China (Hunan).

#### Notes.

Only one other species is known from China (Fujian): *Indiopius alutacius* Weng & Chen, 2001. It has the head yellowish-brown dorsally (with only the frons medially infuscate; dark brown in *Indiopius chenae*), the scutellar sulcus is narrow (moderately wide) and the fore femur is moderately slender (robust).

#### Etymology.

Named after its collector and well-known specialist of aquatic Hemiptera, Dr Ping-Ping Chen (Tiel).

### Neopius

Gahan, 1917 stat. n.

Neopius Gahan, 1917: 203. Type species (by original designation): *Neopius carinaticeps* Gahan, 1917 (= *Opius rudis* Wesmael, 1835)[senior synonym examined].

#### Notes.

Small Holarctic genus that has not yet been found in Hunan, but likely occurs in northern China. The type species occurs in Far East Russia and Korea (Yu et al. 2012). Traditionally *Neopius* Gahan has been included as synonym in the subgenus *Xynobius* (e.g. Fischer 1972b) and recently (Papp 2005) as a subgenus of the genus *Xynobius*. Provisionally, Quicke et al. (1997) included *Neopius* as a subgenus in *Phaedrotoma* because it has no apomorphies in common with *Xynobius* or *Opius* s.s. and *Phaedrotoma* was the “taxonomic dustbin genus” where it was included on the basis of plesiomorphies. The molecular data presented here indicate that it is not closely related to *Phaedrotoma* and, consequently, it is treated as independent genus with its own apomorphies (occipital carina secondarily complete dorsally (sometimes irregular and weak medio-dorsally) and frons distinctly granulate).

### Opiognathus

Fischer, 1972 stat. n.

**Figure 105.**
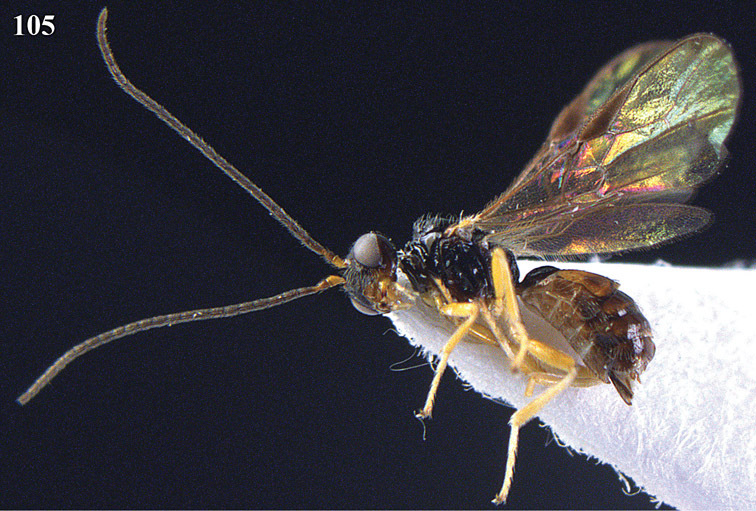
*Opiognathus aulaciferus* sp. n., female, holotype. Habitus lateral.

Figs 105 [Fig F33] [Fig F34] –125

Opiognathus Fischer, 1972b: 140 (as subgenus of *Opius* Wesmael). Type species (by original designation): *Opius pactus* Haliday, 1837 [examined].

#### Diagnosis.

Hind tibia with a long to medium-sized and more or less curved carinula basally (Figs 113, 123); face without tubercles; in front of anterior ocellus without a distinct depression; frons with pair of shallow depressions above antennal sockets; occipital carina present laterally, not or slightly curved ventrally and remain removed from hypostomal carina (Figs 112, 124), near level of middle of eye straight or nearly so, without transverse carina or crest; clypeus more or less convex and high (Figs 111, 122); labrum normal, without emargination ventrally; hypoclypeal depression distinct; malar suture absent; scapus, fore coxa and trochanter at most weakly compressed; epistomal suture without large depressions; mandible gradually (Fig. 112) or abruptly widened baso-ventrally and apical half abruptly narrowed (Fig. 124) or not; pronotum short and subvertical; pronope absent (and with a deep slit-like depression in front of middle lobe of mesoscutum) or large; side of pronotum anteriorly below groove without a distinctly elevated area; medio-posterior depression of mesoscutum large (Fig. 107) or absent (Fig. 117); scutellar sulcus rather wide to medium-sized; propodeum reticulate (Figs 108, 118) or largely smooth posteriorly, with a short or no medio-longitudinal carina and no posterior areola; precoxal sulcus coarsely or very finely crenulate; postpectal carina completely absent; vein 2-SR of fore wing present; first subdiscal cell of fore wing at least partly closed by vein 3-CU1 and comparatively long vein CU1b postero-apically (Figs 116, 127); vein 1-M of fore wing slightly to moderately curved and vein 1-SR medium-sized; vein cu-a of hind wing present and vein m-cu absent; vein 3-SR of fore wing distinctly longer than vein 2-SR; length of fore wing less than 3.5 mm; second tergite without sharp lateral crease, smooth; length of second and third tergites combined less than 0.7 times length of metasoma behind first tergite; fourth and following tergites (at least partly) exposed; ovipositor sheath about 0.1 times as long as fore wing. According to the molecular data, belongs at the base of a clade with *Opius*, *Phaedrotoma* and related genera.

#### Biology.

The type species has been reported as a parasitoid of leaf-mining Tephritidae.

#### Notes.

Unfortunately, Fischer designated thrice a type species of the subgenus *Opiognathus* Fischer, 1972b (not 1972a: 393, it is a nomen nudum in this paper because no type species was designated and more than one species was included (Int. Code Zool. Nom. Art. 13b)). Fischer (1972b) designated *Opius pactus* Haliday, 1837, which has the mandible only gradually widened basally, less than indicated in the general diagnosis. In 1978 Fischer designated a second type species for *Opiognathus*: *Opius mokotoensis* Fischer, 1968, which has indeed the mandible more distinctly widened basally, but this designation is invalid because in 1972 a valid type species was designated. In 1986 Fischer (p. 613) designated a third type species for the subgenus: *Opius dewulfi* Fischer, 1968, which is equally invalid. In this paper the original designation is accepted and the quotation about the widened mandible is considered to be a gradually widened mandible and *Opiognathus* is treated as a valid genus separate from the genus *Utetes* Foerster despite the presence of the carinula of the hind tibia because of morphological and molecular differences. *Opiognathus* can be separated as follows:

**Table:** 

1	Mandible with large rectangular baso-ventral lobe (about as wide as mandible subbasally) or gradually widened baso-ventrally; pronope rather large and elliptical; in front of anterior ocellus flat, without a distinct impression	*Opiognathus* Fischer
–	Mandible normal, without distinct baso-ventral lobe or tooth, at most slightly widened ventrally; pronope large groove- or slit-like or round, rarely absent; area in front of anterior ocellus variable	*Utetes* Foerster

### Opiognathus aulaciferus

Li & van Achterberg sp. n.

urn:lsid:zoobank.org:act:A840EDA2-C9C0-4390-BA55-1BC8F19A0D60

http://species-id.net/wiki/Opiognathus_aulaciferus

Figs 105 –114

#### Type material.

Holotype, ♀ (ZUH), “S. China: Hunan, nr Zhangjiajie, Badagong Mts, Tian Ping Mt., 9–13.VII.2009, 550 m, Xi-Ying Li, RMNH’10”.

#### Diagnosis.

Mandible gradually widened basally (Fig. 112); hypoclypeal depression medium-sized; medio-posterior depression of mesoscutum deep and large (Fig. 107); first tergite sculptured and rather matt (Fig. 108); propodeum coarsely reticulate and without smooth areas (Fig. 108); posterior groove of pronotum widely crenulate; propleuron slightly concave.

#### Description.

Holotype, ♀, length of body 2.3 mm, of fore wing 2.3 mm.

*Head*. Antenna with 26 segments and 1.1 times as long as fore wing; length of third segment 1.2 times fourth segment, length of third, fourth and penultimate segments 3.3, 2.4 and 2.2 times their width, respectively (Fig. 109); length of maxillary palp equal to height of head; labial palp segments moderately elongate; occipital carina moderately close to hypostomal carina and dorsally absent (Fig. 112); median groove behind stemmaticum obsolescent; hypostomal carina narrow; length of eye in dorsal view 2.4 times temple; frons flat and glabrous medially, smooth and laterally convex and setose; face smooth, medially slightly elevated (Fig. 111); width of clypeus twice its maximum height and 0.5 times width of face, clypeus convex, largely smooth and its ventral margin differentiated and slightly curved medially (Fig. 111); hypoclypeal depression medium-sized (Fig. 111); mandible slightly convex gradually widened ventro-basally and with fine ventral carina (Fig. 112).

*Mesosoma*. Length of mesosoma 1.3 times its height; dorsal pronope absent, but with deep slit-like depression in front of middle lobe of mesoscutum (as in type species); pronotal side smooth, no ventral oblique carina posteriorly and posterior groove widely crenulate (Fig. 105); propleuron slightly concave; epicnemial area smooth dorsally; precoxal sulcus only medially distinctly impressed and moderately crenulate (Fig. 105); rest of mesopleuron smooth; pleural sulcus smooth; mesosternal sulcus medium-sized and coarsely crenulate; notauli absent on disc, except for a pair of short crenulate impressions anteriorly; mesoscutum glabrous; medio-posterior depression of mesoscutum large, deep and elliptical (Fig. 107); scutellar sulcus wide and coarsely crenulate (Fig. 107); scutellum smooth and slightly convex; dorsal surface of propodeum short and with a short medio-longitudinal carina connected to an irregular transverse carina and with a nearly triangular areola, without costulae and remaining far from medio-longitudinal carina (Fig. 108).

**Figures 106–114. F33:**
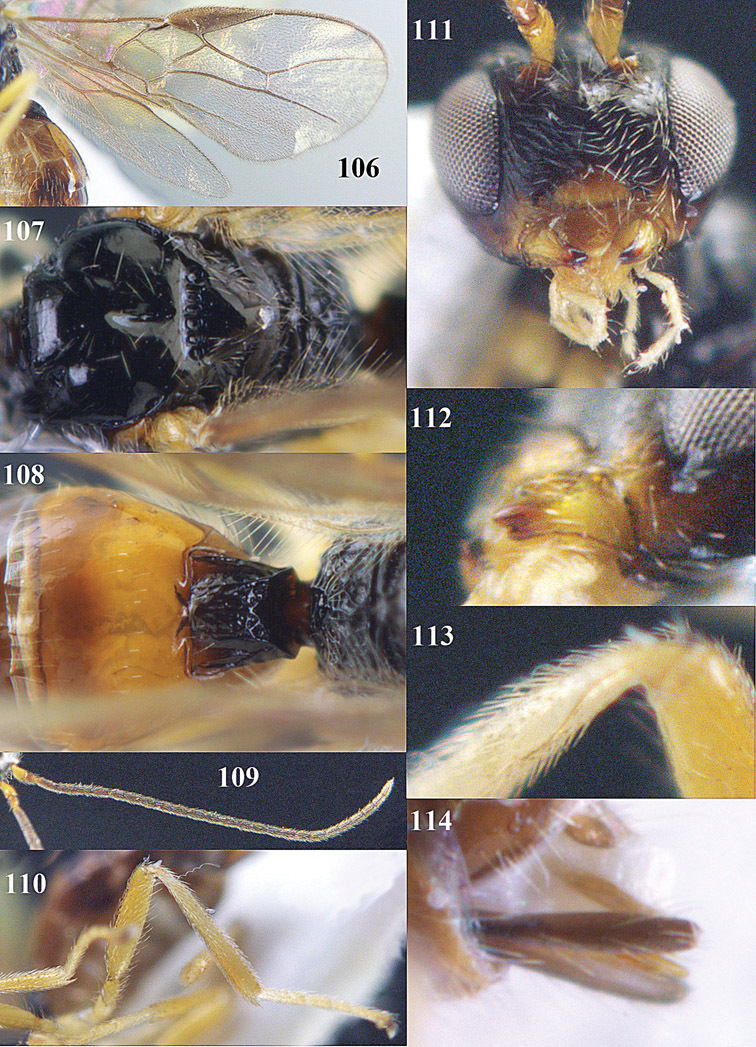
*Opiognathus aulaciferus* sp. n., female, holotype. **106** Wings **107** mesosoma dorsal **108** propodeum and 1^st^-3^rd^ metasomal tergites dorsal **109** antenna **110** hind leg **111** head anterior **112** mandible **113** base of hind tibia inner side **114** ovipositor sheath.

*Wings*. Fore wing (Fig. 106): pterostigma elongate triangular; 1-R1 reaching wing apex and 1.2 times as long as pterostigma; r:3-SR:SR1 = 3:23:42; 2-SR:3-SR:r-m = 14:23:6; r slender; 1-M curved; 2-M and SR1 weakly curved; m-cu moderately far postfurcal and gradually merging into vein 2-CU1; cu-a slightly postfurcal and 1-CU1 widened; first subdiscal cell closed, CU1b comparatively long; apical third of M+CU1 sclerotized. Hind wing (Fig. 106): M+CU:1-M:1r-m = 15:13:8; cu-a straight; m-cu absent.

*Legs*. Length of femur, tibia and basitarsus of hind leg 4.1, 7.1 and 5.2 times as long as wide, respectively (Fig. 110); setae of hind femur and tibia moderately long; carinula of hind tibia long and distinctly curved (Fig. 113).

*Metasoma*. Length of first tergite 1.1 times its apical width, its surface evenly convex medially and reticulate rugose, dorsal carinae remain separated from each other and reaching apex of tergite (Fig. 108); second and following tergites smooth; second suture absent; third and following tergites partly desclerotized apically; length of setose part of ovipositor sheath 0.10 times fore wing and 0.35 times hind tibia (Figs 105, 114).

*Colour*. Black or blackish-brown; antenna dark brown but scapus brownish-yellow; palpi, mandible, tegulae, legs and humeral plate pale yellowish; clypeus and malar space mainly, tegulum, base of second tergite and metasoma baso-ventrally yellowish-brown; propleuron and area below precoxal sulcus dark brown; pterostigma and veins mainly brown; wing membrane subhyaline.

*Molecular data*. None.

#### Distribution.

*China (Hunan).

#### Biology.

Unknown.

#### Etymology.

Name derived from “aulon” (Greek for “channel”) and “fero” (Latin for “carrying”), because of the anteriorly shallowly impressed notauli.

#### Notes.

The new species runs in the key by Chen and Weng (2005) to *Utetes antennbrevis* Weng & Chen, 2005. *Opiognathus aulaciferus* differs by having the length of the pleural sulcus smooth (crenulate in *Utetes antennbrevis*), the second submarginal cell of the fore wing comparatively narrow (wide), length of the antenna 1.1 times fore wing (about 0.8 times) and head and mesosoma mainly blackish-brown (reddish-brown).

### Opiognathus brevibasalis

Li & van Achterberg sp. n.

urn:lsid:zoobank.org:act:8768DAB9-003E-4C7C-AE41-BE35F6CB257D

http://species-id.net/wiki/Opiognathus_brevibasalis

Figs 115 –125

#### Type material.

Holotype, ♀ (ZUH), “S. China: Hunan, nr Zhangjiajie, Badagong Mts, Bamaoxi, 2–3.VI.2009, 540 m, Xi-Ying Li, RMNH’09”. Paratypes (RMNH): 3 ♀, same label data.

#### Diagnosis.

Mandible abruptly widened basally (Fig. 122); hypoclypeal depression absent (Fig. 122); medio-posterior depression of mesoscutum absent; first tergite largely smooth and shiny (Fig. 118); propodeum largely smooth, with square areola posteriorly, remaining far removed from short medio-longitudinal carina and from transverse subbasal carina (Fig. 118); posterior groove of pronotum smooth; propleuron flattened.

#### Description.

Holotype, ♀, length of body 2.1 mm, of fore wing 2.0 mm.

*Head*. Antenna with 24 segments and 1.2 times as long as fore wing; length of third segment 1.3 times fourth segment, length of third, fourth and penultimate segments 4.2, 3.2 and 2.2 times their width, respectively (Fig. 119); length of maxillary palp 0.9 times height of head; labial palp segments short, about twice as long as wide; occipital carina moderately close to hypostomal carina (Fig. 124) and dorsally absent; median groove behind stemmaticum obsolescent; hypostomal carina rather narrow; length of eye in dorsal view 1.8 times temple; frons glabrous medially and laterally, smooth; face smooth, medially slightly elevated; width of clypeus twice its maximum height and 0.6 times width of face, clypeus convex, largely smooth and its ventral margin differentiated and nearly straight medially (Fig. 122); hypoclypeal depression absent (Fig. 122); mandible slightly convex, abruptly widened ventro-basally and with a short ventral carina (Fig. 124).

**Figure 115. F34:**
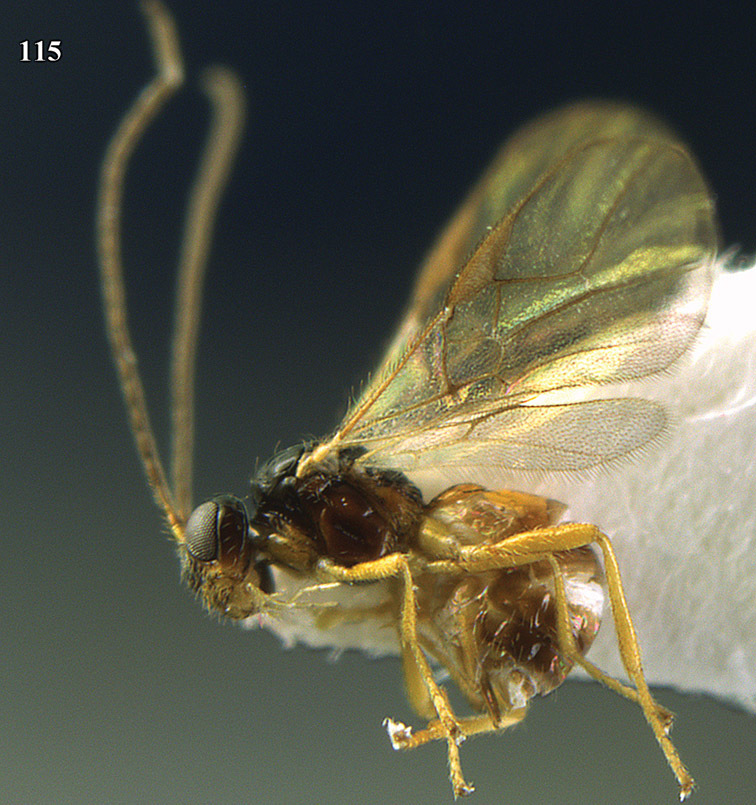
*Opiognathus brevibasalis* sp. n., female, holotype. Habitus lateral.

*Mesosoma*. Length of mesosoma 1.2 times its height; dorsal pronope large and round (Fig. 121); pronotal side largely smooth, oblique groove largely finely crenulate, with a ventral oblique carina posteriorly and posterior groove absent or nearly so, slightly finely crenulate (Fig. 115); propleuron flattened; epicnemial area smooth dorsally; precoxal sulcus only medially distinctly impressed and finely crenulate (Fig. 115); rest of mesopleuron smooth; pleural sulcus smooth; mesosternal sulcus narrow and finely crenulate; notauli absent on disc, except for a pair of short partly crenulate impressions anteriorly; mesoscutum glabrous (Fig. 117); medio-posterior depression of mesoscutum absent; scutellar sulcus medium-sized and finely crenulate; scutellum smooth and slightly convex; dorsal surface of propodeum short and with a short medio-longitudinal carina connected to a rather regular transverse carina, largely smooth, posteriorly with small square areola, remaining far removed from medio-longitudinal carina and transverse carina (Fig. 118).

**Figures 116–125.**
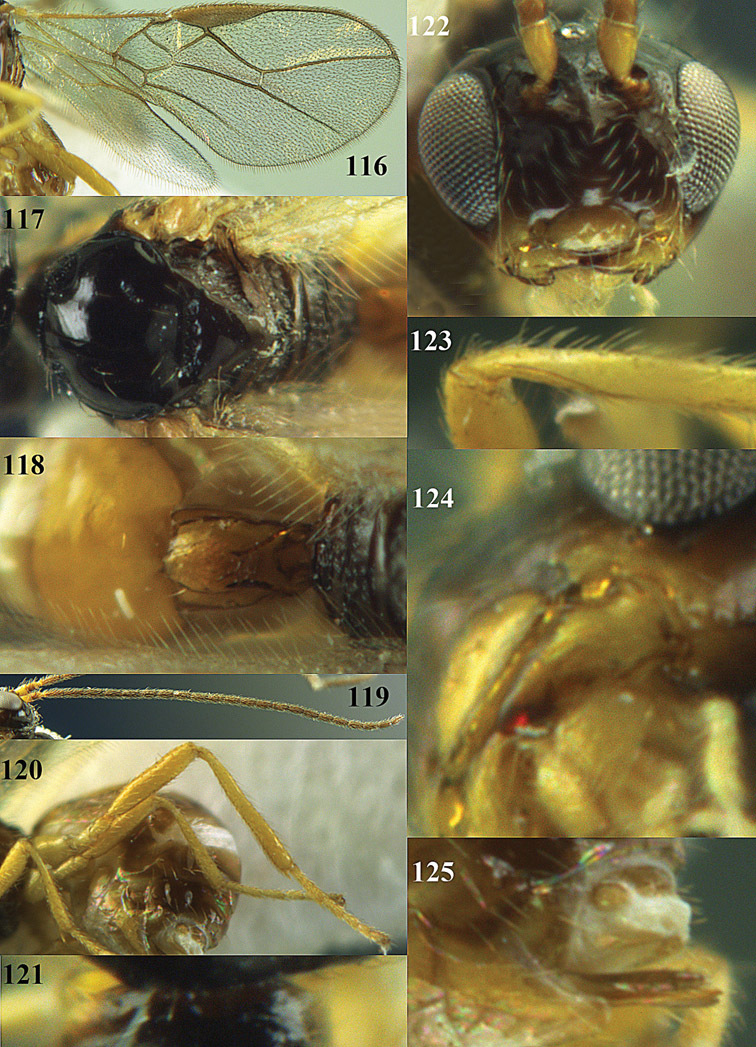
*Opiognathus brevibasalis* sp. n., female, holotype. **116** Wings **117** mesosoma dorsal **118** propodeum and 1^st^-2^nd^ metasomal tergites dorsal **119** antenna **120** hind leg **121** pronope dorsal **122** head anterior **123** base of hind tibia inner side **124** mandible **125** ovipositor sheath.

*Wings*. Fore wing (Fig. 116): pterostigma elongate triangular; 1-R1 reaching wing apex and 1.2 times as long as pterostigma; r:3-SR:SR1 = 1:22:44; 2-SR:3-SR:r-m = 14:22:6; r slender; 1-M curved; 2-M and SR1 nearly straight; m-cu moderately far postfurcal and angled to vein 2-CU1; cu-a interstitial; first subdiscal cell closed, CU1b comparatively long; apical third of M+CU1 sclerotized. Hind wing (Fig. 116): M+CU:1-M:1r-m = 15:12:7; cu-a straight; m-cu absent.

*Legs*. Length of femur, tibia and basitarsus of hind leg 4.9, 8.8 and 4.7 times as long as wide, respectively (Fig. 120); setae of hind femur and tibia moderately long; carinula of hind tibia long and distinctly sinuate (Fig. 120).

*Metasoma*. Length of first tergite 1.1 times its apical width, its surface evenly convex medially but posteriorly protuberant and largely smooth, dorsal carinae remain separated from each other (except for a short ruga) and end near middle of tergite (Fig. 118); second and following tergites smooth; second suture absent; third and following tergites partly desclerotized apically; length of setose part of ovipositor sheath 0.06 times fore wing and 0.2 times hind tibia (Figs 115, 125).

*Colour*. Brownish-black; antenna dark brown but scapus brownish-yellow; palpi, mandible, clypeus and malar space mainly, tegulae, legs, propleuron, pronotal side postero-ventrally and metasoma ventrally pale yellowish; mesopleuron largely, metapleuron, metasoma largely dorsally yellowish-brown; pterostigma and veins mainly brown; wing membrane subhyaline.

*Molecular data*. None.

*Variation*. Length of body 1.7-2.1 mm and of fore wing 2.0-2.2 mm; antennal segments of ♀ 24 (1), 25 (1) or 26 (2); first metasomal tergite yellowish-brown or mainly dark brown.

#### Distribution.

*China (Hunan).

#### Biology.

Unknown.

#### Etymology.

Name derived from “brevis” (Latin for “short”) and “basis” (Latin for “base”), because of the basally shortly widened mandibles.

#### Notes.

The new species runs (with some difficulty) in the key by Chen and Weng (2005) to *Rhogadopsis pratensis* (Weng & Chen, 2005) comb. n. *Opiognathus brevi-basalis* differs by having a sinuate carinula on the hind tibia (absent in *Rhogadopsis pratensis*), the medio-posterior depression of the mesoscutum absent (present), the first tergite yellowish-brown apically and similar to second tergite (reddish-brown and darker) and length of first tergite 1.1 times its apical width (1.3 times).

### Opiostomus

Fischer, 1972 stat. n.

Opiostomus Fischer, 1972a: 389 (as subgenus of *Opius* Wesmael, 1835). Type species (by monotypy): *Opius kovacsi* Fischer, 1963.
Snoflakopius Fischer, 1972b: 70, 177 (as subgenus of *Opius* Wesmael, 1835) Type species (by original designation): *Opius snoflaki* Fischer, 1959 [examined]. **Syn. n.**
Jucundopius Fischer, 1984a: 34 (as subgenus of *Opius* Wesmael, 1835), 1998: 22, 24-25 (as subgenus in the genus *Eurytenes* Wesmael, 1836). Type species (by original designation): *Opius (Jucundopius) jucundicola* Fischer, 1984 [examined]. **Syn. n.**
Opiotenes Fischer, 1998: 23 (as subgenus of *Eurytenes* Wesmael, 1836). Type species (by original designation): *Opius leptostigma* Wesmael, 1835 [examined].**Syn. n.**
Oetztalotenes Fischer, 1998: 22-23 (as subgenus of *Eurytenes* Wesmael, 1836). Type species (by original designation): *Eurytenes (Oetztalotenes) oetztalicola* Fischer, 1998 [examined].**Syn. n.**

#### Diagnosis.

Hypoclypeal depression absent or narrow, and medially ventral margin of clypeus near upper level of condyli of mandibles (“mouth closed”); mandible strongly twisted, its second tooth hardly visible in lateral view, and with a medium-sized to large ventro-basal tooth, but minute or obsolescent in the subgenus *Snoflakopius* Fischer (stat. n.); pronope usually absent and at most medium-sized; pterostigma sublinear; vein 3-SR of fore wing 1.4 times vein 2-SR or longer; hind coxae normal, rounded ventrally; second-fourth tarsal segments comparatively slender; telotarsus and arolium not enlarged; dorsope usually large and close to lateral margin of first tergite; hypopygium of female at most slightly incised.

#### Biology.

Parasitoids of Agromyzidae.

#### Distribution.

Holarctic, Afrotropical, northern Oriental.

#### Notes.

Ranked as a genus, because it does not fit in *Opius* and, according to its morphology, it is different from other genera with dorsope present.

### Opiostomus aureliae

(Fischer, 1957) comb. n.

http://species-id.net/wiki/Opiostomus_aureliae

Opius aureliae Fischer, 1957: 343 [examined].

#### Additional material.

1 ♀ (HANC), “S. China: Hunan, Zhangjiajie, Forest Park, 1.VIII.1989, Benzhy Dai, No. 213”.

#### Biology.

Unknown.

#### Distribution.

Holarctic, North Oriental (China: Hunan). New for China.

### Genus Opius

Wesmael, 1835 s. str.

Figs 126 [Fig F37] [Fig F38] [Fig F39] [Fig F40] [Fig F41] [Fig F42] [Fig F43] [Fig F44] [Fig F45] [Fig F46] [Fig F47] [Fig F48] –195

Opius Wesmael, 1835: 113; Fischer, 1972b: 67-69. Type species (designation by Muesebeck and Walkley (1951), validated by ICZN Opinion 1497 (1988): *Opius pallipes* Wesmael, 1835 [examined].
Hypolabis Foerster, 1862: 115. Type species (by original designation): *Opius pallipes* Wesmael, 1835 [examined].
Cryptonastes Foerster, 1862: 260. Type species (by original designation): *Cryptonastes tersus* Foerster, 1862 [examined].
Misophthora Foerster, 1862: 266. Type species (by original designation): *Misophthora laevigata* Foerster, 1862 [examined].
Hypocynodus Foerster, 1862: 260. Type species (by original designation): *Opius crassipes* Wesmael, 1835 [examined].
Desmatophorus Thomson, 1895: 2194. Type species (by Viereck 1914b): *Bracon pygmaeator* Nees, 1812 [type lost].
Stomosema Fischer, 1972b: 70, 330. Type species (by original designation): *Opius mutus* Fischer, 1964.
Nosopaeopius Fischer, 1972b: 70, 206. Type species (by original designation): *Opius ochrogaster* Wesmael, 1835 [examined].
Pendopius Fischer, 1972b: 71, 409. Type species (by original designation): *Opius pendulus* Haliday, 1837 [examined].
Allophlebus Fischer, 1972b: 71, 416. Type species (by original designation): *Opius singularis* Wesmael, 1835 [examined].
Opiothorax Fischer, 1972b: 71, 441, 1995: 220-221 (key to Palaearctic species). Type species (by original designation): *Opius levis* Wesmael, 1835 [examined].
Odontopoea Fischer, 1987: 610. Type species (by original designation): *Opius (Nosopoea) epulatus* Papp, 1981[examined].

#### Diagnosis.

Face without tubercles; in front of anterior ocellus without distinct depression; frons without pair of distinct depressions above antennal sockets, but whole frons may be depressed; occipital carina present laterally, not or slightly curved ventrally and remain removed from hypostomal carina (Figs 132, 142, 153, 163, 173, 183, 193), near level of middle of eye straight or nearly so, without transverse carina or crest; clypeus more or less convex and higher, usually narrower, or longer, not impressed; labrum normal, without emargination ventrally; scapus, fore coxa and trochanter at most weakly compressed; epistomal suture without large depressions; mandible asymmetrical and more or less abruptly widened basally (Figs 132, 142, 153, 163, 173, 183, 193), with rectangular or acute angle, rarely in males less developed (e.g. *Opius ochrogaster*); medio-posterior depression of mesoscutum variable; pronope round or wide elliptical, or pronotum only with a shallow transverse groove; scutellar sulcus usually rather wide; propodeum usually smooth or superficially sculptured; postpectal carina completely absent; vein 2-SR of fore wing present, rarely absent; first subdiscal cell of fore wing at least partly closed by vein 3-CU1 postero-apically (Figs 127, 137, 146, 158, 168, 178, 187); vein 1-M of fore wing usually straight; vein cu-a of hind wing nearly always present; vein 3-SR of fore wing distinctly longer than vein 2-SR; if subequal then vein m-cu of hind wing or precoxal sulcus (almost) absent; length of fore wing usually less than 3.5 mm; second and basal half of third tergite without sharp lateral crease, if sometimes weakly developed then second tergite smooth; length of second and third tergites combined less than 0.7 times length of metasoma behind first tergite; fourth and following tergites (at least partly) exposed; ovipositor sheath more or less setose basally.

#### Biology.

Parasitoids of mining larvae of Agromyzidae, Anthomyiidae, Drosophilidae, Tephritidae, Ephydridae and Cecidomyiidae.

#### Notes.

The asymmetrical (excluding ventro-basal carina) and more or less abruptly widened mandible is essential for the recognition of this group. Some opiine species may cause confusion because of a rather wide ventro-basal carina of the mandible but are excluded from *Opius* s.s. because the mandible itself (in ventro-lateral view) is gradually widened basally.

### Opius crenuliferus

Li & van Achterberg sp. n.

urn:lsid:zoobank.org:act:0B2F7D87-9042-4B7C-8DF2-00B28DAE794D

http://species-id.net/wiki/Opius_crenuliferus

Figs 126 –135

#### Type material.

Holotype, ♀ (ZUH), “S. China: Hunan, nr Zhangjiajie, Badagong Mts, Bamaoxi, 2–3.VI.2009, 540 m, Xi-Ying Li, RMNH’09”, “CVA4252, sp. 18”.

#### Diagnosis.

**Figure 126.**
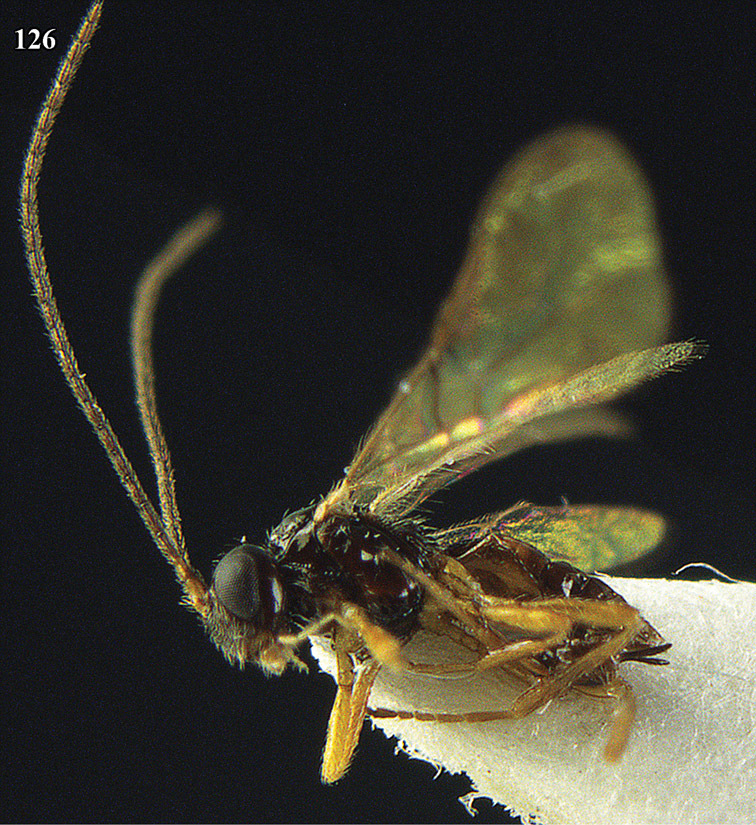
*Opius crenuliferus* sp. n., female, holotype. Habitus lateral.

**Figures 127–135. F37:**
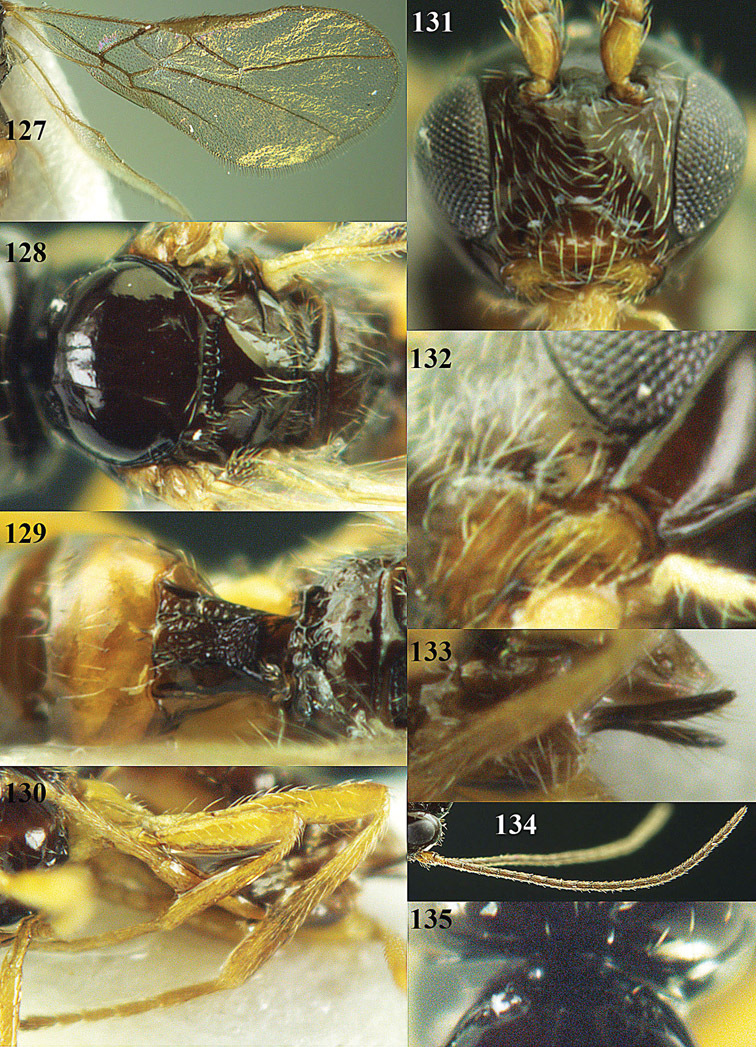
*Opius crenuliferus* sp. n., female, holotype. **127** Wings **128** mesosoma dorsal **129** propodeum and 1^st^-2^nd^ metasomal tergites dorsal **130** hind leg **131** head anterior **132** mandible **133** ovipositor sheath **134** antenna **135** pronope dorsal.

Oblique groove of pronotum largely crenulate; setose part of ovipositor sheath 0.06 times as long as fore wing and about 0.7 times as long as first tergite (Fig. 126); clypeus truncate ventrally; hypoclypeal depression narrow slit-shaped (Fig. 131); medio-posterior depression of mesoscutum absent (Fig. 128); vein CU1b of fore wing short (Fig. 127); vein r of fore wing short (Fig. 127); vein m-cu of fore wing parallel to vein 1-M (Fig. 127); hind tibia apically and tarsus brownish.

#### Description.

Holotype, ♀, length of body 1.8 mm, of fore wing 2.1 mm.

*Head*. Antenna with 25 segments and 1.2 times as long as fore wing; third segment 1.1 times as long as fourth segment, length of third, fourth and penultimate segments 4.0, 3.5 and 2.3 times their width, respectively (Fig. 134); length of maxillary palp 0.9 times height of head; labial palp segments petiolate, rather moniliform; occipital carina rather close to hypostomal carina and dorsally absent (Fig. 132); hypostomal carina medium-sized; length of eye in dorsal view 2.1 times temple; frons flattened and glabrous, smooth; face smooth, medially weakly elevated; width of clypeus 1.6 times its maximum height and 0.55 times width of face; clypeus flattened, smooth except for some punctures and its ventral margin thin and straight (Fig. 131); hypoclypeal depression slit-like, narrow (Fig. 131); malar suture present; without punctures between malar suture and clypeus; mandible abruptly widened baso-ventrally, with medium-sized and weakly protruding ventral carina (Fig. 132).

*Mesosoma*. Length of mesosoma 1.2 times its height; dorsal pronope obsolescent and pronotum oblique anteriorly (Fig. 135); oblique groove of pronotum largely crenulate, remainder of sides smooth and posterior groove largely absent (Fig. 126); epicnemial area smooth dorsally; precoxal sulcus medially shallowly impressed, smooth as rest of mesopleuron; pleural sulcus smooth; mesosternal sulcus very narrow and very finely crenulate; notauli absent on disc, only anteriorly with short smooth impressions (Fig. 128); mesoscutum glabrous and strongly shiny; medio-posterior depression of mesoscutum absent; scutellar sulcus narrow and finely crenulate; scutellum smooth and slightly convex; surface of propodeum smooth, except for some short curved carinae medio-posteriorly (Fig. 129).

*Wings*. Fore wing (Fig. 127): pterostigma long elliptical; 1-R1 reaching wing apex and 1.3 times as long as pterostigma; r:3-SR:SR1 = 1:20:51; 2-SR:3-SR:r-m = 13:20:5; r -widened; 1-M straight and SR1 nearly so; m-cu slightly postfurcal; cu-a postfurcal and 1-CU1 hardly widened; first subdiscal cell narrowly open, CU1b short; M+CU1 nearly completely unsclerotized; vein m-cu of fore wing parallel to vein 1-M. Hind wing (Fig. 127): M+CU:1-M:1r-m = 15:16:8; cu-a straight; m-cu absent.

*Legs*. Length of femur, tibia and basitarsus of hind leg 4.6, 9.6 and 5.0 times as long as wide, respectively; hind femur with long and tibia with medium-sized setae. (Fig. 130)

*Metasoma*. Length of first tergite 1.1 times its apical width, its surface distinctly convex medially and coarsely rugose (including basally between dorsal carinae) and dorsal carinae developed in basal 0.6 of tergite, straight (Fig. 129); second suture absent; second and following tergites smooth; length of setose part of ovipositor sheath 0.06 times fore wing and 0.2 times hind tibia (Figs 126, 133).

*Colour*. Dark or blackish-brown; scapus ventrally and pedicellus apically yellowish; palpi pale yellowish; clypeus ventrally, mandible, tegulae and legs (but hind tibia apically and hind tarsus slightly darkened) brownish-yellow; mesopleuron partly chestnut-brown; second tergite and metasoma baso-ventrally yellowish-brown; pterostigma and veins mainly brown; wing membrane subhyaline.

*Molecular data*. COI, 16S, 28S (CVA 4252).

#### Distribution.

*China (Hunan).

#### Biology.

Unknown.

#### Etymology.

Name derived from “crenulatus” (Latin for “minutely notched”) and “ferum” (suffix in Latin meaning carrying or having), because of the crenulate oblique groove of the pronotal side.

#### Notes.

A female from Tian Ping Mt. and sampled as CVA4253 is very similar but is according to the molecular data a different species. It has the crenulate oblique groove of the pronotum, but the propodeum is steep and the segments of the labial palp are less moniliform. Molecular data from COI place it near *Opiognathus* and from 28S near *Opiognathus* + *Coleopioides*.

The new species runs (with some difficulty) in the key by Chen and Weng (2005) to *Opius clusilis* Weng & Chen, 2005. *Opius crenuliferus* differs by having the head roundly narrowed behind eyes in dorsal view (directly narrowed in *Opius clusilis*), the dorsal pronope obsolescent (distinct), length of the first tergite 1.1 times its apical width (1.4 times) and length of the third antennal segment 4 times its width (2.6 times).

### Opius malarator

Li, van Achterberg & Tan sp. n.

urn:lsid:zoobank.org:act:286BDBC6-734C-41B2-828C-2E801D24ECDC

http://species-id.net/wiki/Opius_malarator

Figs 136 –144

#### Type material.

Holotype, ♂ (ZUH), “S. China: Hunan, nr Zhangjiajie, Badagong Mts, Bamaoxi, 2–3.VI.2009, 540 m, Xi-Ying Li, RMNH’09”.

#### Diagnosis.

Malar suture deep (Fig. 142); clypeus wide (Fig. 141); second submarginal cell of fore wing large (Fig. 137); hypoclypeal depression large and deep (Fig. 141).

#### Description.

Holotype, ♂, length of body 2.7 mm, of fore wing 3.2 mm.

*Head*. Antenna with 29 segments and 1.1 times as long as fore wing; third segment 1.2 times as long as fourth segment, length of third, fourth and penultimate segments 3.8, 3.2 and 2.7 times their width, respectively (Fig. 143); length of maxillary palp 1.3 times height of head; labial palp segments normal; occipital carina far removed from hypostomal carina and dorsally absent; hypostomal carina wide; length of eye in dorsal view 1.2 times temple; frons flattened anteriorly and glabrous, smooth; face smooth, medially elevated; width of clypeus 2.4 times its maximum height and 0.55 times width of face and semi-elliptical (Fig. 142); clypeus slightly convex, somewhat protruding forwards, smooth except for some punctures and its ventral margin thin and straight (Fig. 141); hypoclypeal depression wide and deep (Fig. 141); labrum slanted backwards; malar suture deep (Fig. 142); mandible with tooth-like protrusion, with a narrow ventral carina and second tooth small (Fig. 142).

**Figure 136. F38:**
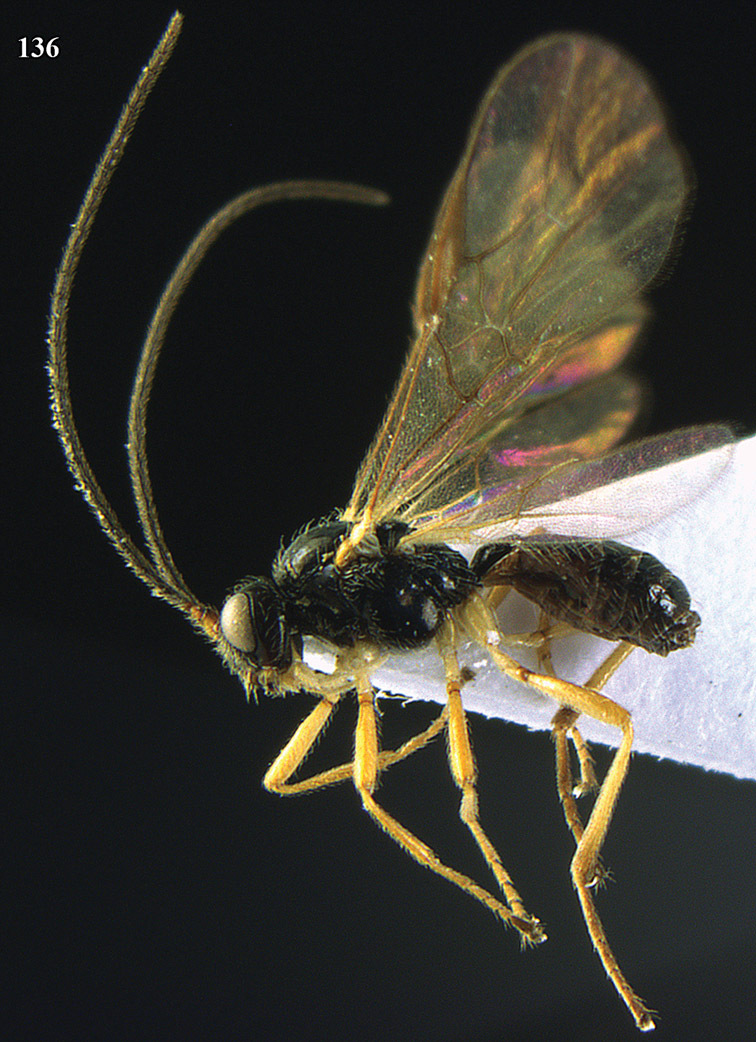
*Opius malarator* sp. n., male, holotype. Habitus lateral.

*Mesosoma*. Length of mesosoma 1.3 times its height; dorsal pronope deep, round, medium-sized (Fig. 144); pronotal side smooth and posterior groove largely absent (Fig. 136); epicnemial area smooth dorsally; precoxal sulcus hardly impressed, smooth as rest of mesopleuron (Fig. 136); pleural sulcus smooth; mesosternal sulcus rather deep and narrow and finely crenulate; notauli absent on disc, only anteriorly with pair of short smooth impressions (Fig. 138); mesoscutum glabrous and shiny; medio-posterior depression of mesoscutum absent; scutellar sulcus narrow and moderately crenulate; scutellum slightly convex medially; surface of propodeum smooth, except for a square rugose area medio-posteriorly (Fig. 139).

**Figures 137–144. F39:**
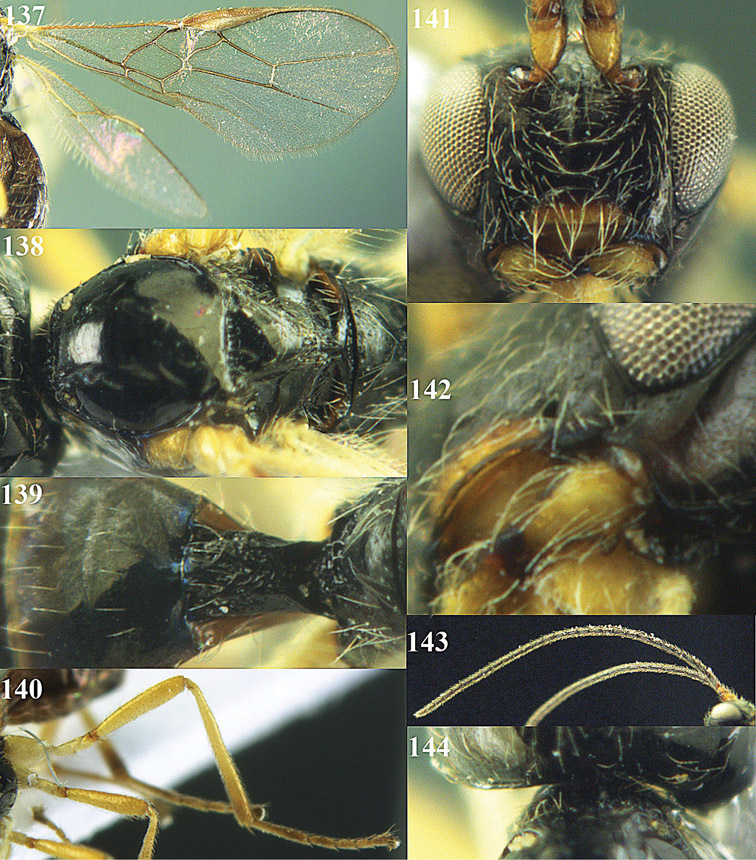
*Opius malarator* sp. n., male, holotype. **127** Wings **128** mesosoma dorsal **139** propodeum and 1^st^-2^nd^ metasomal tergites dorsal **140** hind leg **141** head anterior **142** mandible **143** antenna **144** pronope dorsal.

*Wings*. Fore wing (Fig. 137): pterostigma elliptical; 1-R1 reaching wing apex and 1.2 times as long as pterostigma; r:3-SR:SR1 = 4:26:56; 2-SR:3-SR:r-m = 21:26:9; r slender; 1-M nearly straight and SR1 straight; m-cu and cu-a distinctly postfurcal; 1-CU1 hardly widened; first subdiscal cell closed, CU1b short; apical fifth of M+CU1 sclerotized. Hind wing (Fig. 137): M+CU:1-M:1r-m = 15:13:8; cu-a straight; m-cu as a superficial impression; basal cell comparatively wide.

*Legs*. Length of femur, tibia and basitarsus of hind leg 5.2, 11.2 and 7.3 times as long as wide, respectively; hind femur and tibia with medium-sized setae (Fig. 140).

*Metasoma*. Length of first tergite 1.4 times its apical width, its surface convex but medially flattened and coarsely rugose and dorsal carinae developed in basal 0.4 of tergite (Fig. 139); second suture obsolescent; second and following tergites smooth; third tergite distinctly wider than second tergite.

*Colour*. Black; antenna (but scapus yellowish), metasoma (except first tergite), pterostigma and veins dark brown; palpi, mandible, clypeus ventrally, tegulae and legs (but hind tibia apically, hind tarsus and telotarsi darkened) pale yellow; subhyaline.

*Molecular data*. None.

#### Distribution.

*China (Hunan).

#### Biology.

Unknown.

#### Etymology.

Name derived from “mala” (Latin for “cheek”), because the deeply impressed malar suture.

#### Notes.

The new species runs in the key by Chen and Weng (2005) to *Phaedrotoma tabularis* (Weng & Chen, 2005) comb. n. *Opius malarator* differs by having vein 3-SR of fore wing 1.2 times vein 2-SR (1.8 times in *Phaedrotoma tabularis*), the malar suture deep and complete (absent), the antenna with about 29 segments (39) and the dorsal carinae absent on posterior half of the tergite (up to apex).

### Opius monilipalpis

Li & van Achterberg sp. n.

urn:lsid:zoobank.org:act:DF42D810-6E1A-4A6F-B9D0-5F4BB26842DE

http://species-id.net/wiki/Opius_monilipalpis

Figs 145 –156

#### Type material.

Holotype, ♀ (ZUH), “S. China: Hunan, nr Zhangjiajie, Badagong Mts, Bamaoxi, 2–3.VI.2009, 540 m, Xi-Ying Li, RMNH’09”. Paratypes (91 ♀ + 18 ♂; RMNH, ZUH): 28 ♀ + 2 ♂ with same data; 47 ♀ + 7 ♂, id., but Longtanping, 4-5.VI.2009, 550 m; 1 ♀ + 2 ♂, id., but Tian Ping Mt, 9–13.VII.2009, 550 m; 13 ♀ + 1 ♂, “S. China: Hunan, nr Suining, Huangsang N. R., Shaoyang, 12-13.VI.2009, 1000 m, Xi-Ying Li, RMNH’09”; 1 ♀ + 6 ♂, “S. China: Hunan, nr Chengbu, Nan Mt., Shaoyang, 1500 m, 10-11.VI.2009, Xi-Ying Li, RMNH’09”; 1 ♀, “S. China: Hunan, Changsha, garden Hunan Agr. Univ., 80 m, 31.V.2009, Xi-Ying Li, RMNH’09”.

#### Diagnosis.

**Figure 145. F40:**
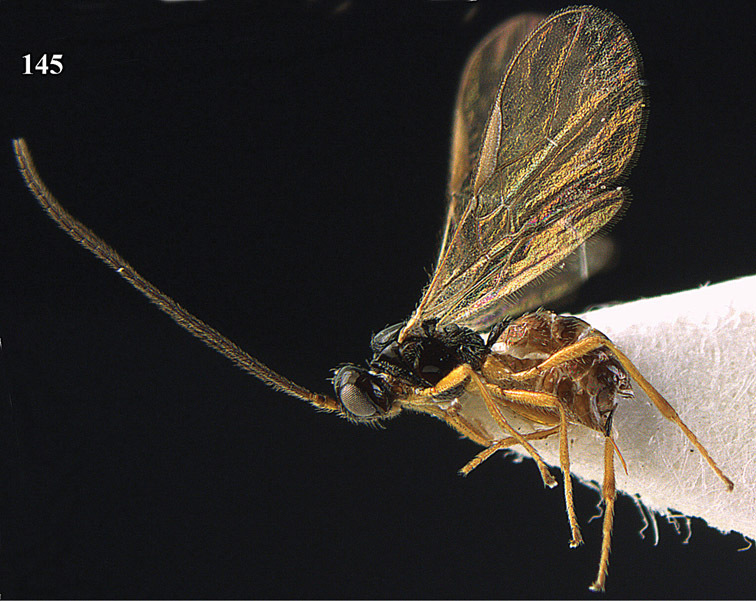
*Opius monilipalpis* sp. n., female, holotype. Habitus lateral.

**Figures 146–156. F41:**
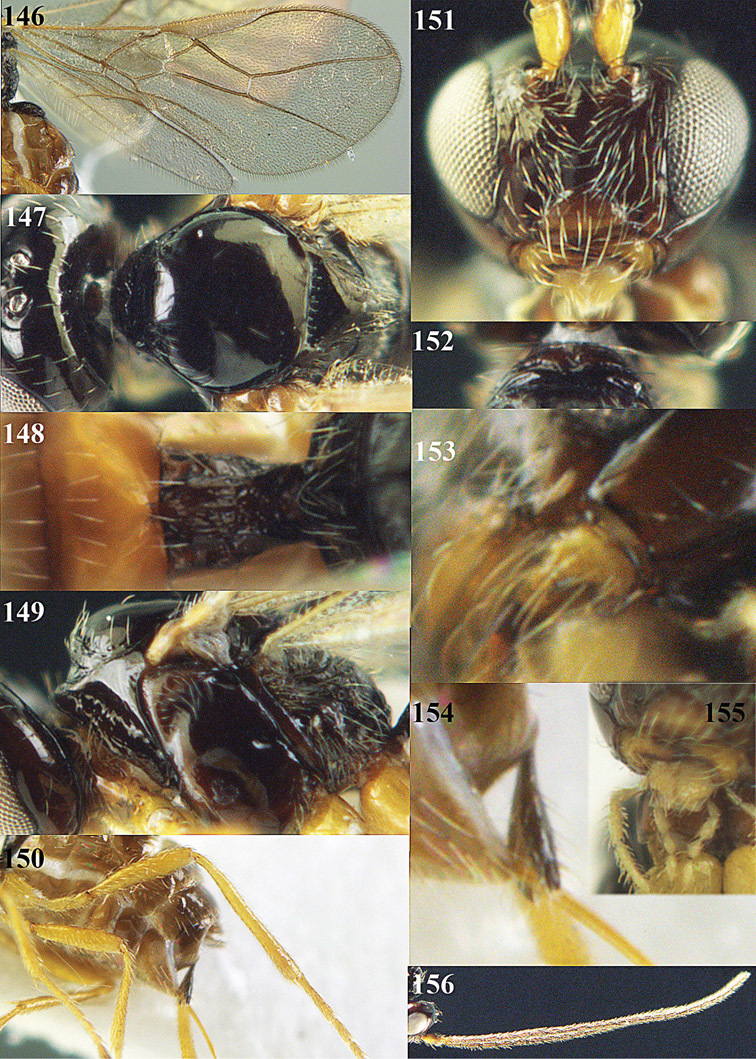
*Opius monilipalpis* sp. n., female, holotype. **146** Wings **147** mesosoma dorsal **148** propodeum and 1^st^-2^nd^ metasomal tergites dorsal **149** mesosoma lateral **150** hind leg **151** head anterior **152** pronope dorsal **153** mandible **154** ovipositor sheath **155** labial palpi **156** antenna.

Antenna with 23–27 segments and 1.1–1.3 times as long as fore wing; setose part of ovipositor sheath 0.06 times as long as fore wing and about half as long as first tergite (Fig. 145); clypeus truncate ventrally with a lamelliform rim and hypocly-peal depression distinct but rather narrow clypeus ventrally (Fig. 151); segments of labial palp moniliform, strongly narrowed basally (Fig. 155); medio-posterior depression of mesoscutum absent; propodeum and mesopleuron black or dark brown; propodeum less steep posteriorly than of *Opius pallipes*; vein r of fore wing distinct; second submarginal cell of fore wing more widened basally than in *Opius pallipes* (Fig. 146); vein CU1b of fore wing narrowly developed (Fig. 146); hind femur yellowish-brown dorso-apically hind tibia apically and tarsus usually infuscate.

#### Description.

Holotype, ♀, length of body 1.9 mm, of fore wing 2.2 mm.

*Head*. Antenna with 25 segments and 1.2 times as long as fore wing; length of third segment 1.1 times fourth segment, length of third, fourth and penultimate segments 4.2, 3.8, and 2.3 times their width, respectively (Fig. 156); length of maxillary palp 0.9 times height of head; labial palp segments petiolate, moniliform (Fig. 155); occipital carina moderately far removed hypostomal carina (Fig. 153) and dorsally absent; hypostomal carina narrow; length of eye in dorsal view 1.5 times temple; frons slightly convex and glabrous, smooth; face smooth, medially weakly elevated; width of clypeus 1.8 times its maximum height, clypeus flattened, largely smooth and its ventral margin thin and straight (Fig. 151); hypoclypeal depression slit-like, rather narrow (Fig. 151); malar suture present; mandible moderately widened basally, with short and weakly protruding ventral carina (Fig. 153).

*Mesosoma*. Length of mesosoma 1.4 times its height; dorsal pronope distinct, but rather small, round (Fig. 148); pronotal side mainly smooth, but medial groove faintly crenulate and posterior groove absent (Fig. 145); epicnemial area smooth dorsally; precoxal sulcus only medially shallowly impressed, smooth as rest of mesopleuron (Fig. 145); pleural sulcus smooth; notauli absent on disc, only anteriorly indicated by some rugulae (Fig. 147); mesoscutum glabrous except for a few setae; medio-posterior depression of mesoscutum absent; scutellar sulcus moderately crenulate; scutellum smooth and flat; surface of propodeum largely smooth, but medio-posteriorly rugulose (Fig. 148).

*Wings*. Fore wing (Fig. 146): pterostigma comparatively wide elliptical; 1-R1 reaching wing apex and 1.3 times as long as pterostigma; r:3-SR:SR1 = 1:21:48; 2-SR:3-SR:r-m = 12:21:6; 1-M straight; SR1 nearly straight; m-cu moderately postfurcal; cu-a postfurcal and 1-CU1 widened; first subdiscal cell narrowly open, CU1b minute. Hind wing (Fig. 146): M+CU:1-M:1r-m = 15:16:10; cu-a straight; m-cu absent.

*Legs*. Length of femur, tibia and basitarsus of hind leg 5.0, 9.6 and 4.8 times as long as wide, respectively; hind femur with long setae and of tibia medium-sized (Fig. 150).

*Metasoma*. Length of first tergite 1.1 times its apical width, its surface distinctly convex medially and with some rugae, but largely smooth and dorsal carinae developed in its anterior 0.7, parallel or nearly so medially (Fig. 148); second and following tergites smooth; length of ventrally visible setose part of ovipositor sheath 0.06 times fore wing and 0.2 times hind tibia (Figs 145, 154).

*Colour*. Black; antenna (but scapus laterally and ventrally and pedicellus ventrally yellowish), mesopleuron, first tergite, second tergite subposteriorly, third tergite medially, apical half of fourth tergite, most of following tergites and ovipositor sheath dark brown; second-fourth tergites membranous and pale yellowish posteriorly; clypeus, mandible, tegulae, propleuron and remainder of metasoma largely yellowish-brown; palpi and legs pale yellowish, but apex of middle and hind tibia, hind tarsus and telotarsi more or less infuscate; pterostigma and veins mainly brown; wing membrane hyaline.

*Molecular data*. COI, 16S, 28S (CVA 4242 (♀) and 4247 (♂)).

*Variation*. Length of body 1.5-1.9 mm, of fore wing 1.9-2.3 mm; antenna of female with 23 (2), 24 (17), 25 (42), 26 (15) or 27 (3) segments and 1.1-1.3 times as long as fore wing and 1.5-1.6 times body, of male with 23 (2), 24 (6), 25 (3) or 26 (1) segments and 1.2-1.3 times as long as fore wing; first tergite largely smooth or largely rugose-reticulate; side of pronotum black to largely yellowish-brown; mesopleuron and first tergite medio-posteriorly sometimes largely orange-brown; rarely first tergite and metapleuron entirely yellowish-brown; second tergite sometimes brown or partly dark brown; palpi of males less moniliform than of females; clypeus yellowish-brown to largely dark brown; sometimes legs completely pale yellow.

#### Distribution.

*China (Hunan).

#### Biology.

Unknown, but Wen et al. (2002) reported *Liriomyza* species (Agromyzidae) as hosts of their specimens that may belong to this species (see below).

#### Etymology.

Name derived from “monilis” (Latin for “string of beads”) and “palpus” (Latin for “feeler”), because of the petiolate labial palp segments.

#### Notes.

Very similar to *Opius pallipes* Wesmael, 1835 (Figs 413-416) but the new species has the third labial palp segment bead-like petiolate (gradually narrowed and normal shaped in *Opius pallipes*), the hypoclypeal depression medium-sized (minute), the ventral margin of the clypeus straight (slightly convex), the propleuron usually yellowish-brown (black); the hind femur of female narrow and pale yellowish (moderately wide and yellowish-brown), the first subdiscal cell of fore wing rather robust (comparatively slender), the antenna of the male 1.2-1.3 times as long as body (1.4-1.6 times), the second metasomal tergite of female yellowish-brown (usually dark brown) and there are differences in DNA sequences (COI, 16S). Most likely the *Opius pallipes* reported from South China (Wen et al. 2002, Fischer 2004) belongs to *Opius monilipalpis* sp. n. We have seen a female from North China (RMNH: Shandong, Anqiu, Suotou Mt., 31.VII.2009, c. 120 m) with normal third segment of labial palp (as in *Opius pallipes*) and third antennal segment about 4 times as long as wide, but with a medium-sized hypoclypeal depression and the propleuron entirely and the first tergite medio-posteriorly brownish. It may be a species closely related to *Opius pallipes* Wesmael and *Opius moniliformis* sp. n. It is not the very similar East Palaearctic *Opius extusus* Papp, 1981, because the female of *Opius extusus* has the antenna twice as long as its body.

The new species runs in the key by Chen and Weng (2005) to *Phaedrotoma postuma* (Chen & Weng, 2005) comb. n. *Opius monilipalpis* differs by having the mandible abruptly narrowed subbasally and assymmetical (gradually narrowed and nearly symmetrical in *Phaedrotoma postuma*), length of the mesosoma 1.4 times its height (1.2 times), the width of clypeus 1.8 times its maximum height (3 times), the pterostigma comparatively wide elliptical (narrow elliptical) and the length of the maxillary palp 0.9 times height of head (1.1–1.2 times).

### Opius pachymerus

Li & van Achterberg sp. n.

urn:lsid:zoobank.org:act:78C4E3C0-23FB-4C2A-AB15-6174856D7FB7

http://species-id.net/wiki/Opius_pachymerus

Figs 157 –166

#### Type material.

Holotype, ♀ (ZUH), “S. China: Hunan, Shaoyang, nr Suining, Huangsang N. R., 12–13.VI.2009, 1000 m, Xi-Ying Li, RMNH’09”, “CVA4251, sp. 17”. Paratype, ♀ (RMNH), “China: Hunan, Changde, Taoyuan, Cha-an-pu, Song-yang-ping, tea plantation (8), CN 1012, 31.vii.2010, P.-P. Chen, RMNH’11”.

#### Diagnosis.

**Figure 157. F42:**
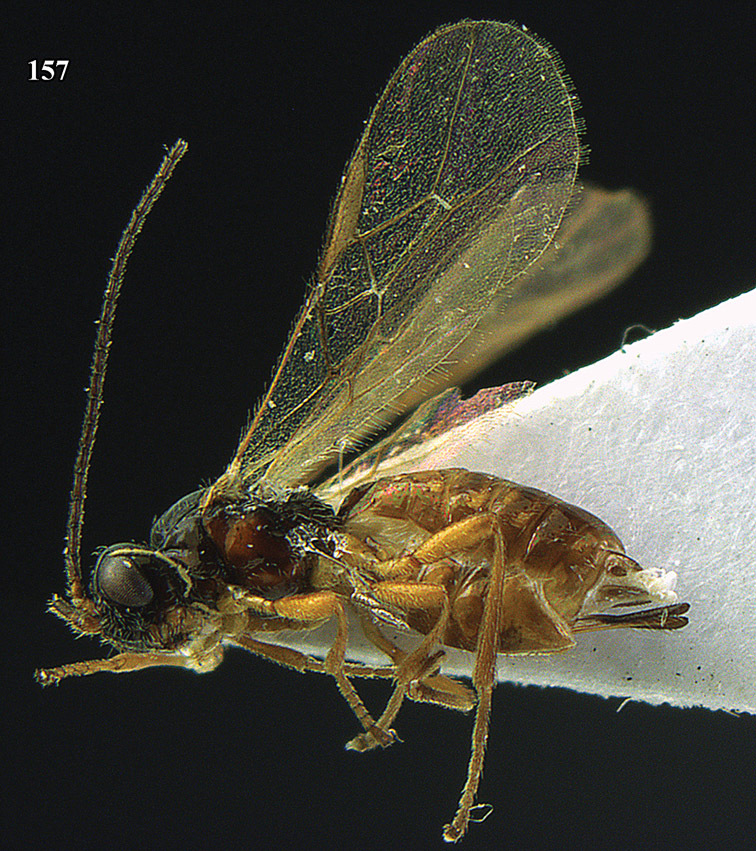
*Opius pachymerus* sp. n., female, holotype. Habitus lateral.

Hind femur robust (Fig. 157); third antennal segment of ♀ about 3.4 times as long as wide (Fig. 165); area between malar suture and clypeus with some distinct punctures (Fig. 163); setose part of ovipositor sheath about 0.16 times as long as fore wing and about 1.3 times as long as first tergite (Figs 157, 164); clypeus flattened; epistomal suture distinctly impressed; pronotum flattened and horizontal medio-anteriorly and with a minute round pronope; mesopleuron largely chestnut-brown.

#### Description.

Holotype, ♀, length of body 2.6 mm, of fore wing 2.5 mm.

*Head*. Antenna incomplete, with 21 segments remaining (paratype: with 30 segments and 1.3 times as long as fore wing); third segment as long as fourth segment, length of third and fourth segments 3.4 and 3.3 times their width, respectively (Fig. 165); length of maxillary palp equal to height of head; labial palp segments petiolate, rather moniliform (Fig. 157); occipital carina far removed from hypostomal carina (Fig. 163) and dorsally absent; hypostomal carina narrow; length of eye in dorsal view 1.4 times temple; frons slightly convex and glabrous, smooth; face smooth, medially weakly elevated; width of clypeus 1.8 times its maximum height and 0.55 times width of face; clypeus flattened, largely smooth (except some dorsal punctures) and its ventral margin thin and straight (Fig. 162); hypoclypeal depression slit-like, rather narrow (Fig. 162); malar suture present; with some distinct punctures between malar suture and clypeus (Fig. 163); mandible gradually widened baso-ventrally, with medium-sized and weakly protruding ventral carina (Fig. 163).

*Mesosoma*. Length of mesosoma 1.4 times its height; dorsal pronope minute, round and pronotum horizontal anteriorly (Fig. 166); pronotal side smooth and posterior groove absent (Fig. 157); epicnemial area smooth dorsally; precoxal sulcus only medially shallowly impressed, smooth as rest of mesopleuron (Fig. 157); pleural sulcus smooth; mesosternal sulcus narrow and finely crenulate; notauli absent on disc, only anteriorly indicated pair of narrow smooth depressions (Fig. 159); mesoscutum glabrous and strongly shiny; medio-posterior depression of mesoscutum absent (Fig. 159); scutellar sulcus narrow and finely crenulate; scutellum smooth and slightly convex; surface of propodeum smooth, except for some short carinae posteriorly (Fig. 160).

**Figures 158–166. F43:**
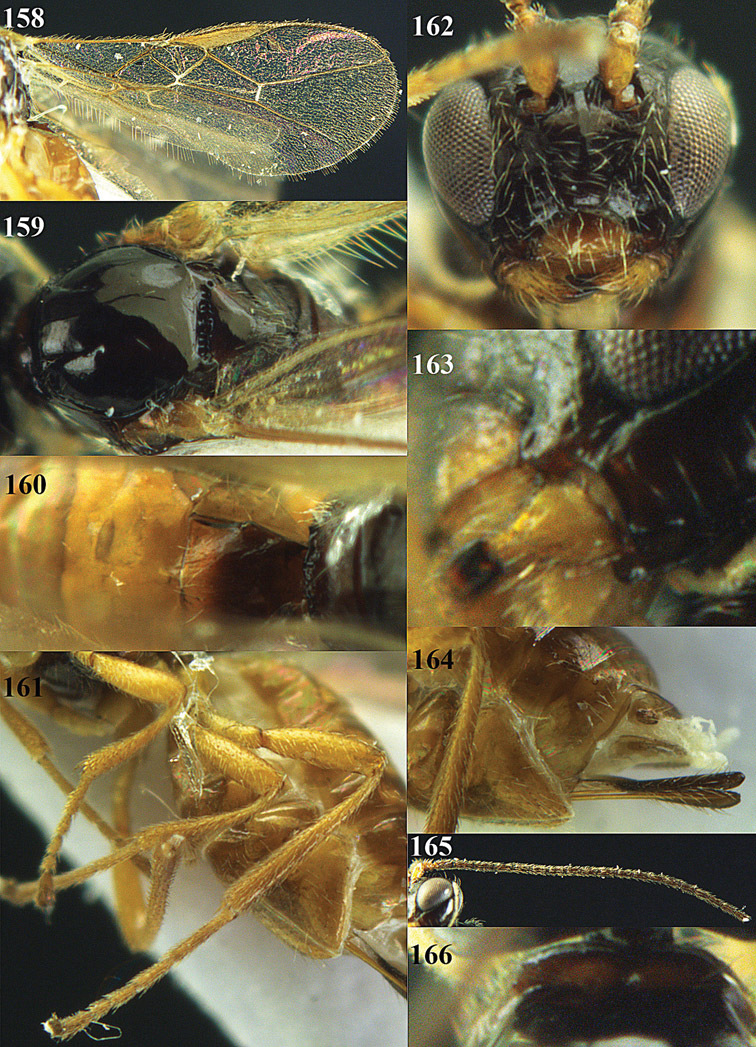
*Opius pachymerus* sp. n., female, holotype. **158** Wings **159** mesosoma dorsal **160** propodeum and 1^st^-2^nd^ metasomal tergites dorsal **161** hind leg **162** head anterior **163** mandible **164** ovipositor sheath **165** antenna **166** pronope dorsal.

*Wings*. Fore wing (Fig. 158): pterostigma long elliptical; 1-R1 reaching wing apex and 1.1 times as long as pterostigma; r:3-SR:SR1 = 2:26:57; 2-SR:3-SR:r-m = 16:26:7; r widened; 1-M and SR1 straight; m-cu slightly postfurcal; cu-a slightly postfurcal and 1-CU1 widened; first subdiscal cell narrowly open, CU1b absent; M+CU1 nearly completely unsclerotized. Hind wing (Fig. 158): M+CU:1-M:1r-m = 5:5:2; cu-a straight; m-cu absent, except for a superficial impression.

*Legs*. Length of femur, tibia and basitarsus of hind leg 3.3, 8.8 and 7.0 times as long as wide, respectively; hind femur and tibia with medium-sized setae (Fig. 161).

*Metasoma*. Length of first tergite equal to its apical width, its surface distinctly convex medially and largely smooth and dorsal carinae developed next to spiracles, straight (Fig. 160); second suture slightly impressed; second and following tergites smooth; length of ventrally visible setose part of ovipositor sheath 0.16 times fore wing, 1.3 times first tergite and 0.5 times hind tibia (Figs 157, 164).

*Colour*. Black; antenna (but scapus and pedicellus yellowish) and mesosternum dark brown; palpi, tegulae and legs pale yellowish, but apex hind tibia and hind tarsus and telotarsi slightly darkened; clypeus ventrally, mandible and metasoma largely (but basal 0.8 of first tergite dark brown) brownish-yellow; mesopleuron largely chestnut- brown; pterostigma and veins mainly brown; wing membrane subhyaline.

*Variation*. Female paratype has 30 antennal segments, first tergite somewhat rugulose posteriorly, pterostigma brown and mesosoma largely chestnut brown.

*Molecular data*. COI, 16S, 28S (CVA4251).

#### Distribution.

*China (Hunan).

#### Biology.

Unknown.

#### Etymology.

Name derived from “pachys” (Greek for “thick”) and “meros” (Greek for “part”), because of the thick parts of the leg, especially the femora.

#### Notes.

The new species runs in the key by Chen and Weng (2005) to *Opius clusilis* Weng & Chen, 2005. *Opius pachymerus* differs by having the head roundly narrowed behind the eyes in dorsal view (directly narrowed in *Opius clusilis*), the dorsal pronope minute (large), length of the first tergite equal times its apical width (1.4 times), the width of the clypeus 1.8 times its height (4 times) and length of the hind femur 3.3 times its width (4.6 times).

### Opius songi

Li & van Achterberg sp. n.

urn:lsid:zoobank.org:act:E900BE3F-A2AE-43A8-B05B-1C2E1DDB48A0

http://species-id.net/wiki/Opius_songi

Figs 167 –176

#### Type material.

Holotype, ♀ (ZUH), “S. China: Hunan, nr Zhangjiajie, Badagong Mts, Bamaoxi, 2–3.VI.2009, 540 m, Xi-Ying Li, RMNH’09”.

#### Diagnosis.

**Figure 167. F44:**
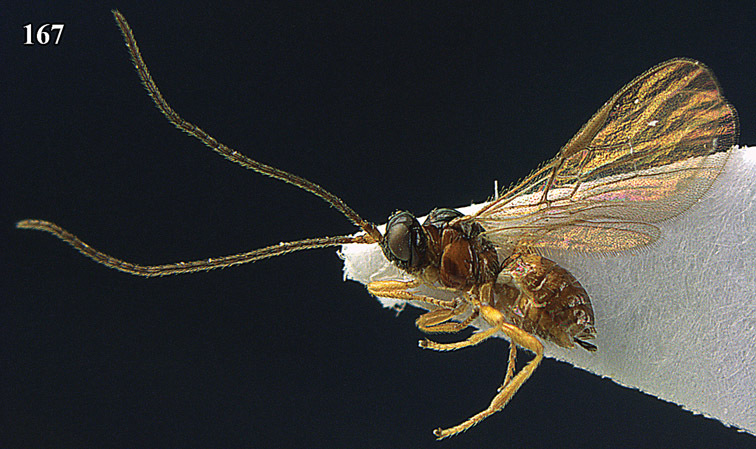
*Opius songi* sp. n., female, holotype. Habitus lateral.

**Figures 168–176. F45:**
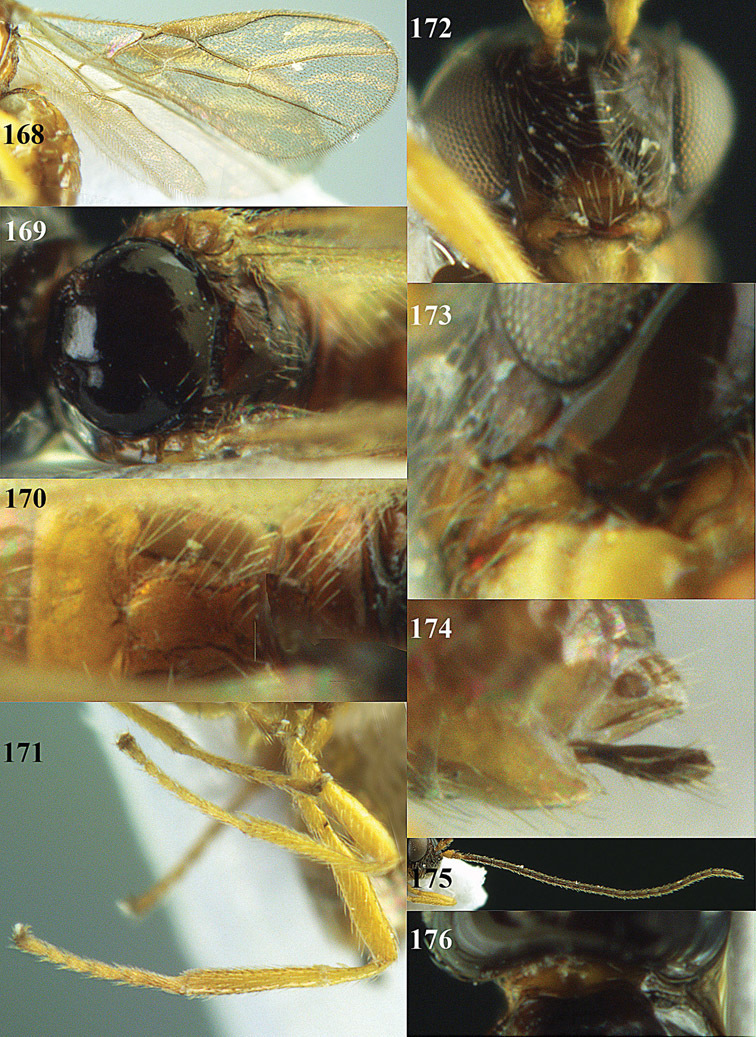
*Opius songi* sp. n., female, holotype. **168** Wings **169** mesosoma dorsal **170** propodeum and 1^st^-2^nd^ metasomal tergites dorsal **171** hind leg **172** head anterior **173** mandible **174** ovipositor sheath **175** antenna **176** pronope dorsal.

Setose part of ovipositor sheath 0.06 times as long as fore wing and about half as long as first tergite (Figs 167, 174); clypeus truncate ventrally; hypocly-peal depression narrowly developed (Fig. 172); pronotum yellowish-brown; pronope medium-sized (Fig. 176); medio-posterior depression of mesoscutum absent; propodeum and mesopleuron orange-brown; propodeum steep posteriorly (Fig. 170); vein r of fore wing distinct; vein CU1b of fore wing narrowly developed; second submarginal cell of fore wing slightly widened basally (Fig. 168); hind femur yellowish-brown dorso-apically.

#### Description.

Holotype, ♀, length of body 1.6 mm, of fore wing 1.9 mm.

*Head*. Antenna with 27 segments and 1.4 times as long as fore wing; third segment 1.1 times as long as fourth segment, length of third, fourth and penultimate segments 3.7, 3.2 and 2.7 times their width, respectively (Fig. 175); length of maxillary palp 0.8 times height of head; labial palp segments petiolate, rather moniliform; occipital carina moderately close to hypostomal carina and dorsally absent; hypostomal carina medium-sized; length of eye in dorsal view 2.4 times temple; frons flattened and glabrous, smooth; face smooth, medially weakly elevated; width of clypeus 1.7 times its maximum height and 0.5 times width of face; clypeus flattened, smooth and its ventral margin thin and straight; hypoclypeal depression slit-like, rather narrow (Fig. 172); malar suture present; without punctures between malar suture and clypeus; mandible gradually widened baso-ventrally, with medium-sized and weakly protruding ventral carina (Fig. 173).

*Mesosoma*. Length of mesosoma 1.2 times its height; dorsal pronope medium-sized, round and pronotum horizontal anteriorly (Fig. 176); pronotal side smooth and posterior groove absent; epicnemial area smooth dorsally; precoxal sulcus anteriorly and medially shallowly impressed, smooth as rest of mesopleuron (Fig. 167); pleural sulcus smooth; mesosternal sulcus very narrow and very finely crenulate; notauli absent on disc, only anteriorly with short smooth impressions (Fig. 169); mesoscutum glabrous and strongly shiny; medio-posterior depression of mesoscutum absent; scutellar sulcus narrow and finely crenulate; scutellum smooth and slightly convex; surface of propodeum smooth, except for some short carinae posteriorly, steep (Fig. 170).

*Wings*. Fore wing (Fig. 168): pterostigma long elliptical; 1-R1 reaching wing apex and 1.2 times as long as pterostigma; r:3-SR:SR1 = 2:21:56; 2-SR:3-SR:r-m = 15:21:7; r slender; 1-M slightly curved and SR1 nearly straight; m-cu slightly postfurcal; cu-a slightly postfurcal and 1-CU1 widened; first subdiscal cell narrowly open, CU1b absent; M+CU1 pigmented but completely unsclerotized. Hind wing (Fig. 168): M+CU:1-M:1r-m = 14:15:6; cu-a straight; m-cu absent, except for a slight impression.

*Legs*. Length of femur, tibia and basitarsus of hind leg 4.2, 9.6 and 5.0 times as long as wide, respectively; hind femur with long and tibia with medium-sized setae. (Fig. 171)

*Metasoma*. Length of first tergite 1.1 times its apical width, its surface distinctly convex medially, posteriorly distinctly angled and superficially rugulose and dorsal carinae developed in basal half of tergite, curved (Fig. 170); second suture absent; second and following tergites smooth; length of setose part of ovipositor sheath 0.06 times fore wing and 0.2 times hind tibia (Figs 167, 174).

*Colour*. Black; scapus and ventrally pedicellus, tegulae, second tergite, metasoma ventrally and legs (but hind tibia apically and hind tarsus and telotarsi slightly darkened) brownish-yellow; palpi pale yellowish; clypeus ventrally, mandible, mesosoma largely (but mesoscutum, scutellum and metanotum blackish-brown or dark brown) and metasoma (except second tergite) yellowish-brown; pterostigma and veins mainly brown; wing membrane subhyaline.

*Molecular data*. None.

#### Distribution.

*China (Hunan).

#### Biology.

Unknown.

#### Etymology.

Named in honour of Prof. Dr Dong-Bao Song, for his encouragement and for his contribution to the knowledge of Chinese Microgastrinae (Braconidae).

#### Notes.

The new species runs (with some difficulty) in the key by Chen and Weng (2005) to *Opius clusilis* Weng & Chen, 2005. *Opius songi* differs by having the head roundly narrowed behind the eyes in dorsal view (directly narrowed in *Opius clusilis*), length of the first tergite 1.1 times its apical width (1.4 times), the length of the pen-ultimate antennal segment of female 2.7 times its width (1.4 times), the length of the antenna 1.4 times length of fore wing (about as long) and length of the third antennal segment 3.7 times its width (2.6 times).

### Opius youi

Li & van Achterberg sp. n.

urn:lsid:zoobank.org:act:222F8D46-E702-4CFF-9FCF-8B7DEAD750D9

http://species-id.net/wiki/Opius_youi

Figs 177 –185

#### Type material.

Holotype, ♂ (ZUH), “S. China: Hunan, nr Zhangjiajie, Badagong Mts, Tian Ping Mt., 9–13.VII.2009, 550 m, Xi-Ying Li, RMNH’10”, “CVA4243, sp. 9”.

#### Diagnosis.

Clypeus flattened and comparatively large (Fig. 182), slightly convex ventrally; hypoclypeal depression narrow, slit-shaped (Fig. 182); pronotum short, oblique and without distinct pronope (Fig. 184) ; notauli absent on disc; medio-posterior depression of mesoscutum absent; hind tarsus (except telotarsus) brownish-yellow.

#### Description.

Holotype, ♂, length of body 1.8 mm, of fore wing 2.0 mm.

*Head*. Antenna with 25 segments and 1.3 times as long as fore wing; third segment 1.1 times as long as fourth segment, length of third, fourth and penultimate segments 3.8, 3.5 and 2.3 times their width, respectively (Fig. 185); length of maxillary palp 0.9 times height of head; labial palp segments short, slender; occipital carina far from hypostomal carina (Fig. 183) and dorsally absent; hypostomal carina medium-sized; length of eye in dorsal view 1.4 times temple; frons flattened and glabrous, smooth; face smooth, medially weakly elevated; width of clypeus 1.8 times its maximum height and 0.55 times width of face; clypeus slightly convex, smooth except for a few fine punctures and its ventral margin thin and slightly curved (Fig. 182); hypoclypeal depression slit-like, narrow (Fig. 182); malar suture present; without punctures between malar suture and clypeus; mandible rather abruptly widened baso-ventrally, with medium-sized and weakly protruding ventral carina (Fig. 183).

**Figure 177. F46:**
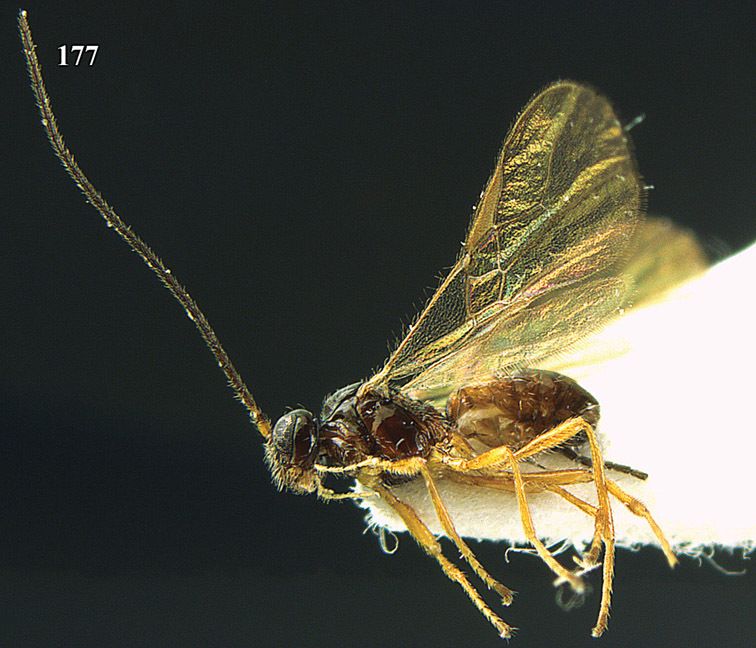
*Opius youi* sp. n., male, holotype. Habitus lateral.

*Mesosoma*. Length of mesosoma 1.2 times its height; dorsal pronope obsolescent and pronotum oblique anteriorly (Fig. 184); pronotal side smooth and posterior groove largely absent (Fig. 177); epicnemial area smooth dorsally; precoxal sulcus medially shallowly impressed, smooth as rest of mesopleuron (Fig. 177); pleural sulcus smooth; mesosternal sulcus narrow and moderately crenulate; notauli absent on disc, only anteriorly with short smooth impressions (Fig. 179); mesoscutum glabrous and strongly shiny; medio-posterior depression of mesoscutum absent; scutellar sulcus narrow and finely crenulate; scutellum smooth and slightly convex; surface of propodeum smooth, except for some short carinae posteriorly (Fig. 180).

**Figures 178–185. F47:**
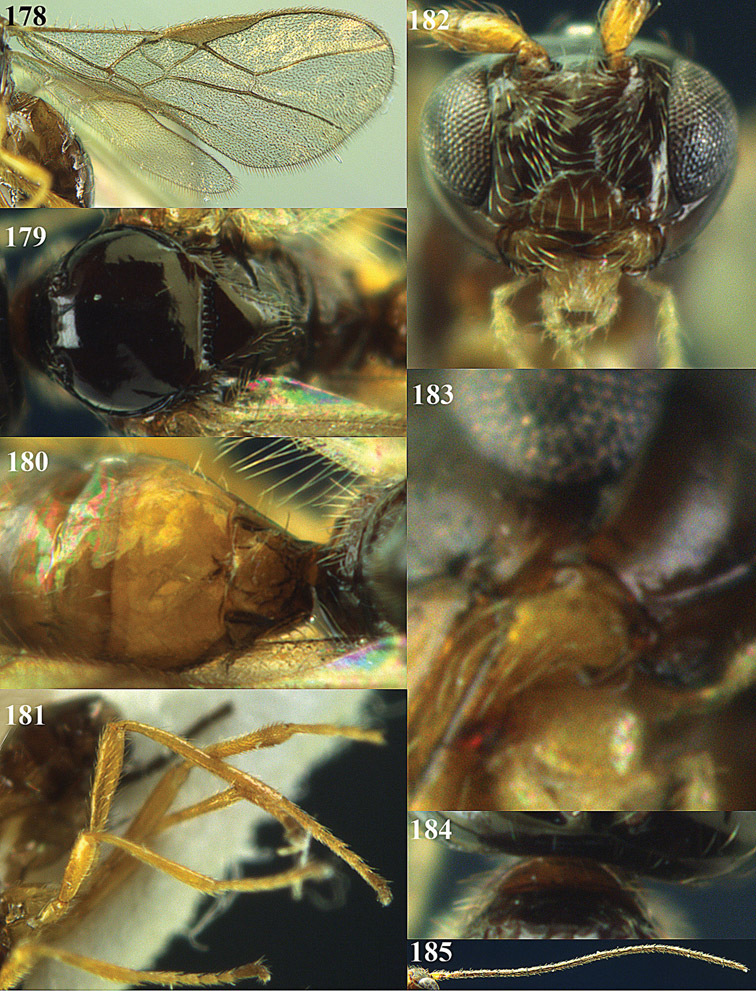
*Opius youi* sp. n., male, holotype. **178** Wings **179** mesosoma dorsal **180** propodeum and 1^st^-3^rd^ metasomal tergites dorsal **181** hind leg **182** head anterior **183** mandible **184** pronope dorsal **185** antenna.

*Wings*. Fore wing (Fig. 178): pterostigma elliptical; 1-R1 reaching wing apex and 1.3 times as long as pterostigma; r:3-SR:SR1 = 1:19:41; 2-SR:3-SR:r-m = 12:19:6; r strongly widened; 1-M straight and SR1 nearly so; m-cu slightly postfurcal; cu-a slightly postfurcal and 1-CU1 slightly widened; first subdiscal cell narrowly open, CU1b absent; M+CU1 nearly completely unsclerotized. Hind wing (Fig. 178): M+CU:1-M:1r-m = 15:14:6; cu-a straight; m-cu absent except for a weak impression.

*Legs*. Length of femur, tibia and basitarsus of hind leg 4.7, 9.2 and 5.0 times as long as wide, respectively; hind femur and tibia with medium-sized setae (Fig. 181).

*Metasoma*. Length of first tergite 1.2 times its apical width, its surface convex medially and sparsely rugulose, largely smooth and dorsal carinae developed in basal half of tergite, straight (Fig. 180); second suture absent; second and following tergites smooth.

*Colour*. Dark brown; scapus yellowish; palpi pale yellowish; clypeus, mandible, tegulae, second tergite, metasoma baso-ventrally and legs (but telotarsi slightly darkened) brownish-yellow; pronotum ventrally, propleuron, first tergite, metasoma after second tergite pterostigma and veins mainly brown; wing membrane subhyaline.

*Molecular data*. COI, 16S, 28S (CVA4243).

#### Distribution.

*China (Hunan).

#### Biology.

Unknown.

#### Etymology.

Named in honour of Prof. Dr Lan-Shao You, for his encouragement and for his contribution to the dissemination of knowledge about Braconidae in China.

#### Notes.

*Opius youi* is, according to 28S data, near the base of the *Phaedrotoma* clade. The new species runs in the key by Chen and Weng (2005) to *Opius clusilis* Weng & Chen, 2005. *Opius youi* differs by having the head roundly narrowed behind the eyes in dorsal view (directly narrowed in *Opius clusilis*), the dorsal pronope obsolescent (distinct), length of the first tergite 1.2 times its apical width (1.4 times) and length of the third antennal segment 3.8 (♂) times its width (2.6 times; ♀). It comes also near *Opius flavus* Weng & Chen, 2005, but it has the hind femur distinctly widened apically, vein r of fore wing about 1.5 times as long as wide, vein m-cu of fore wing more postfurcal and fourth antennal segment of female less slender.

### Opius zengi

Li & van Achterberg sp. n.

urn:lsid:zoobank.org:act:84FF6F95-5A42-403E-92C8-9FC12342A850

http://species-id.net/wiki/Opius_zengi

Figs 186 –195

#### Type material.

Holotype, ♀ (ZUH), “S. China: Hunan, Shaoyang, nr Suining, Huangsang N. R., 12-13.VI.2009, 1000 m, Xi-Ying Li, RMNH’09”.

#### Diagnosis.

**Figure 186. F48:**
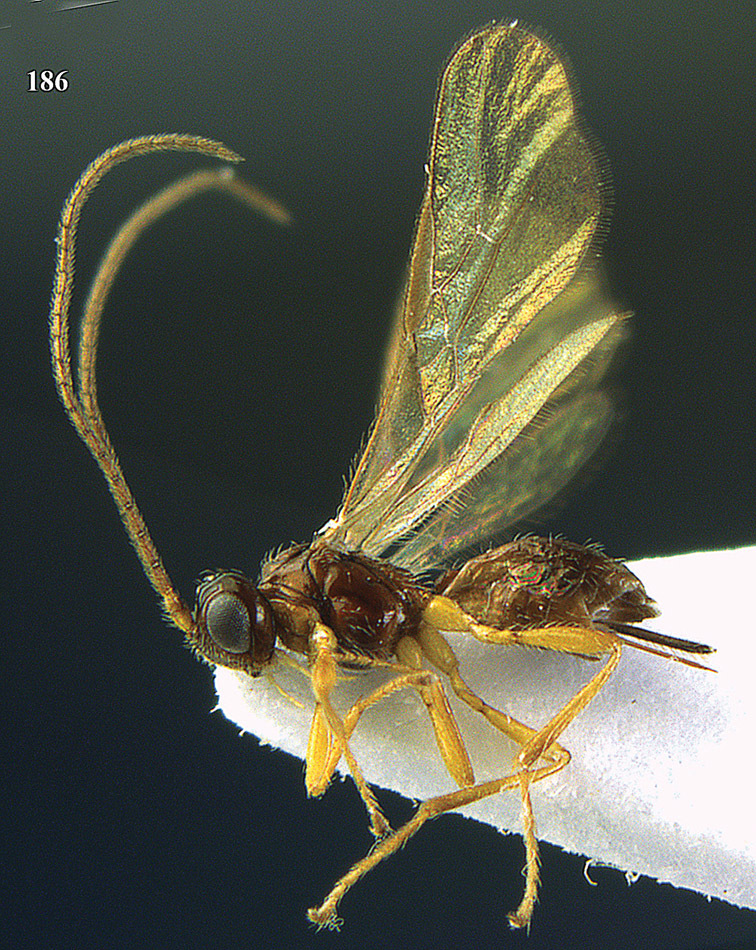
*Opius zengi* sp. n., female, holotype. Habitus lateral.

Hind femur slender (Fig. 190); third antennal segment of ♀ about 4.5 times as long as wide (Fig. 186); area between malar suture and clypeus without distinct punctures (Fig. 193); setose part of ovipositor sheath about 0.15 times as long as fore wing, half as long as hind tibia and 1.5 times as long as first tergite (Fig. 186); clypeus flattened; epistomal suture distinctly impressed; pronotum flattened and horizontal medio-anteriorly and with a minute round pronope (Fig. 194); mesopleuron largely chestnut-brown; first tergite comparatively slender (Fig. 189).

#### Description.

Holotype, ♀, length of body 1.7 mm, of fore wing 2.0 mm.

*Head*. Antenna with 27 segments and 1.2 times as long as fore wing; third segment 1.1 times as long as fourth segment, length of third, fourth and penultimate segments 4.5, 4.0 and 2.6 times their width, respectively (Fig. 191); length of maxillary palp 0.8 times height of head; labial palp segments petiolate, rather moniliform; occipital carina close to hypostomal carina (Fig. 193) and dorsally absent; hypostomal carina medium-sized (Fig. 193); length of eye in dorsal view 2.5 times temple; frons flattened and glabrous, smooth; face smooth, medially weakly elevated; width of clypeus 1.7 times its maximum height and 0.55 times width of face; clypeus flattened, smooth and its ventral margin thin and straight (Fig. 192); hypoclypeal depression slit-like, rather narrow (Fig. 192); malar suture present; without punctures between malar suture and clypeus; mandible gradually widened baso-ventrally, with medium-sized and weakly protruding ventral carina (Fig. 193).

*Mesosoma*. Length of mesosoma 1.4 times its height; dorsal pronope minute, round and pronotum horizontal anteriorly (Fig. 194); pronotal side smooth and posterior groove absent (Fig. 186); epicnemial area smooth dorsally; precoxal sulcus anteriorly and medially shallowly impressed, smooth as rest of mesopleuron (Fig. 186); pleural sulcus smooth; mesosternal sulcus very narrow and very finely crenulate; notauli absent on disc, only anteriorly with short smooth impressions (Fig. 188); mesoscutum glabrous and strongly shiny; medio-posterior depression of mesoscutum absent (Fig. 188); scutellar sulcus narrow and finely crenulate; scutellum smooth and slightly convex; surface of propodeum smooth, except for some short carinae posteriorly (Fig. 189).

**Figures 187–195.**
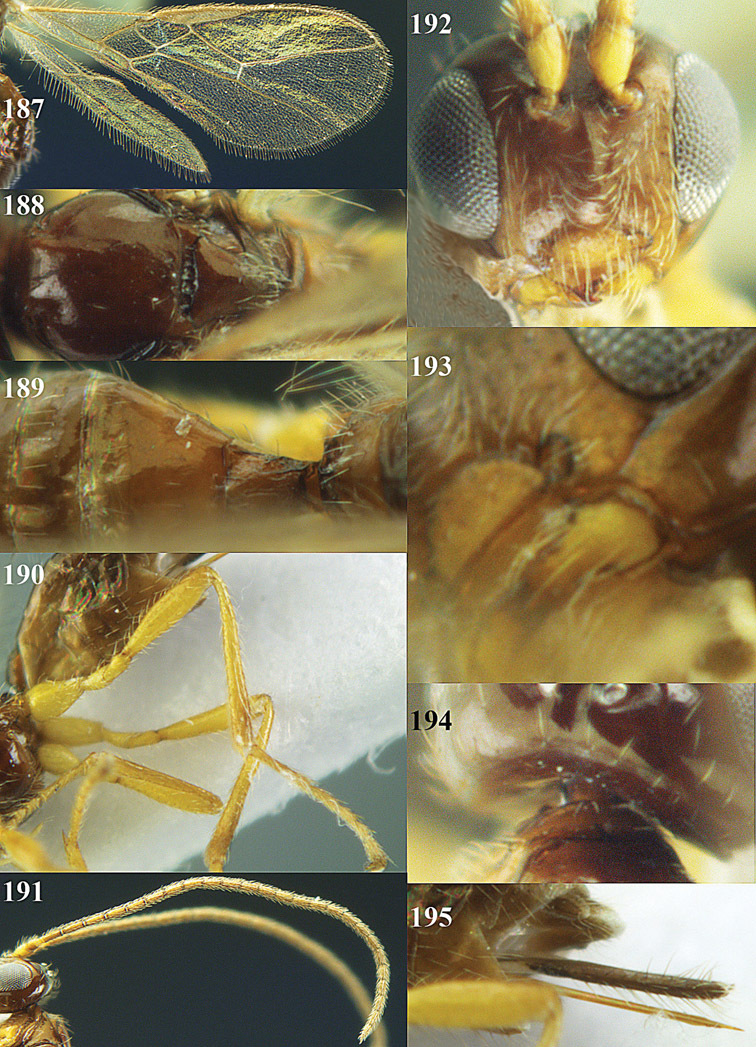
*Opius zengi* sp. n., female, holotype. **187** Wings **188** mesosoma dorsal **189** propodeum and 1^st^-2^nd^ metasomal tergites dorsal **190** hind leg **191** antennae **192** head anterior **193** mandible **194** pronope dorsal **195** ovipositor sheath.

*Wings*. Fore wing (Fig. 187): pterostigma long elliptical; 1-R1 reaching wing apex and 1.4 times as long as pterostigma; r:3-SR:SR1 = 3:23:53; 2-SR:3-SR:r-m = 14:23:6; r somewhat widened; 1-M straight and SR1 nearly so; m-cu slightly postfurcal; cu-a slightly postfurcal and 1-CU1 widened; first subdiscal cell narrowly open, CU1b obsolescent; M+CU1 nearly completely unsclerotized. Hind wing (Fig. 187): M+CU:1-M:1r-m = 5:5:2; cu-a straight; m-cu absent.

*Legs*. Length of femur, tibia and basitarsus of hind leg 4.5, 9.2 and 6.5 times as long as wide, respectively; hind femur and tibia with medium-sized setae (Fig. 190).

*Metasoma*. Length of first tergite 1.2 times its apical width, its surface distinctly convex medially and sparsely rugulose, largely smooth and dorsal carinae developed in basal half of tergite, straight (Fig. 189); second suture absent; second and following tergites smooth; length of setose part of ovipositor sheath 0.15 times fore wing, 1.5 times first tergite and 0.5 times hind tibia (Figs 186, 195).

*Colour*. Dark brown; scapus and pedicellus ventrally yellowish; palpi pale yellowish; clypeus mandible, humeral plate and legs (but hind tibia apically and hind tarsus and telotarsi slightly darkened) brownish-yellow; propleuron, propodeum, metasoma (except first tergite) and metasoma baso-ventrally yellowish-brown; pterostigma and veins mainly brown; wing membrane subhyaline.

*Molecular data*. None.

#### Distribution.

*China (Hunan).

#### Biology.

Unknown.

#### Etymology.

Named in honour of Prof. Dr Ai-Ping Zeng, for his encouragement of and help to the first two authors.

#### Notes.

The new species runs in the key by Chen and Weng (2005) to *Opius clusilis* Weng & Chen, 2005. *Opius zengi* differs by having the head roundly narrowed behind the eyes in dorsal view (directly narrowed in *Opius clusilis*), the setose part of the ovipositor sheath 1.5 times as long as the first tergite (0.6 times), length of the first tergite 1.2 times its apical width (1.4 times) and length of the third antennal segment 4.5 times its width (2.6 times).

### Orientopius

Fischer, 1966

http://species-id.net/wiki/Orientopius\according_to_Li_et_al_2013

Figs 417 –428

Orientopius Fischer, 1966: 147; van Achterberg et al. 2012a: 125, 2012b: 65. Type species (by original designation): *Orientopius curiosigaster* Fischer, 1966 [examined].

#### Diagnosis.

Clypeus truncate medio-ventrally (Fig. 424); labrum exposed; occipital carina present latero-dorsally and weakly or not protruding in lateral view (Fig. 420); head comparatively long in anterior view (Fig. 424) and malar space longer than basal width of mandible (Fig. 425); malar suture present (Fig. 425); inner sides of antennal sockets normal, not protruding (Fig. 424); around base of middle coxa no circular carina; medio-posterior depression of mesoscutum present (Fig. 420); notauli absent posteriorly or as row of punctures; postpectal carina variable, usually partly present medio-ventrally; vein 3-SR of fore wing 0.9-2.0 times as long as vein 2-SR (Fig. 418); metasoma with carapace (Figs 417, 421), but less developed in males; second tergite sculptured and distinctly longer than third tergite (Figs 417, 421); dorsope absent, dorsal carinae of first tergite variable, separated basally or medially united in a median carina (Fig. 421); second metasomal suture distinct (Fig. 421); third tergite of female with a sharp lateral crease (Fig. 417).

#### Biology.

Parasitoids of species of *Phytobia* Lioy, 1864 (Agromyzidae) mining near the cambium of deciduous trees and shrubs (van Achterberg et al. 2012a).

#### Distribution.

Palaearctic, Oriental and Australian (New Guinea) regions.

### Orientopius punctatus

van Achterberg & Li, 2012

http://species-id.net/wiki/Orientopius_punctatus\according_to_Li_et_al_2013

Figs 417 –428

Orientopius punctatus van Achterberg et al., 2012b: 65.

#### Type material.

Holotype (ZUH), ♀, “[S. China: Hunan], Nan Mt., meadow, 18.VII.1988, Fu-Xing Li”.

#### Diagnosis.

Vein SR1 ends near apex of fore wing (Fig. 418); vertex moderately densely punctate, with interspaces mostly wider than diameter of punctures or wider (Fig. 420); antenna dark brown, except basally; malar space about 1.5 times as long as basal width of mandible and head moderately elongate in anterior view (Fig. 424); pterostigma dark brown; mesosoma dark brown or blackish (Figs 419, 420); transverse carina of propodeum distinctly in front of middle of propodeum; hind basitarsus about 3.7 times as long as wide (Fig. 427); dorsal carina of first tergite united subbasally (Fig. 421); second tergite about twice as long as third tergite and with rows of punctures between striae (Fig. 421); third tergite 0.3 times longer than its basal width; third metasomal tergite semi-circular and partly distinctly punctate (Fig. 421); fourth tergite of female smooth and retracted (Fig. 417); setose part of ovipositor sheath 0.6 times as long as combined first-third metasomal tergites, 0.2 times as long as fore wing and 0.8 times as long as hind tibia (Fig. 426).

#### Description.

Holotype, ♀, length of body 2.3 mm, of fore wing 2.5 mm.

*Head*. Antenna with 25 segments and 1.1 times as long as fore wing; third segment 1.1 times as long as fourth segment, length of third, fourth and penultimate segments 2.7, 2.5 and 1.8 times their width, respectively (Figs 423, 428); length of maxillary palp unknown, palp submerged in glue; occipital carina widely removed from hypostomal carina and dorsally absent; hypostomal carina narrow; length of eye in dorsal view 3.3 times temple; temples directly narrowed (Fig. 420) and largely smooth; vertex finely punctate, with interspaces mostly wider than punctures; frons slightly depressed behind antennal sockets and with some curved rugulae, remainder slightly convex and setose, largely finely punctate, with interspaces wider than punctures; face medio-dorsally elevated, coarsely punctate, with interspaces slightly wider than punctures and some striae latero-dorsally; width of clypeus 2.8 times its maximum height and 0.6 times width of face (Fig. 424); clypeus flat, smooth and its ventral margin rather thin and medially straight; hypoclypeal depression wide and deep (Fig. 424); labrum flat (including ventral rim); malar suture complete; with punctures between malar suture and clypeus; length of malar space 1.5 times basal width of mandible (Fig. 425); mandible strongly constricted and twisted apically, without distinct ventral carina, second tooth medium-sized.

*Mesosoma*. Length of mesosoma 1.3 times its height; dorsal pronope absent, pronotum short and nearly vertical anteriorly; pronotal side smooth but oblique groove anteriorly and posterior groove coarsely crenulate (Fig. 419); epicnemial area with few crenulae dorsally; precoxal sulcus distinctly impressed, but posterior 0.4 absent, and coarsely crenulate (Fig. 419); pleural sulcus distinctly crenulate; mesosternal sulcus and postpectal carina not visible because of glue; metapleuron coarsely reticulate ventrally and dorsally largely smooth (except some punctures); notauli impressed and with few crenulae anteriorly, and largely absent on disc; mesoscutum flattened, with large elliptical medio-posterior depression, setose and punctulate; scutellar sulcus wide and with 3 coarse crenulae (Fig. 420); scutellum rather flat and sparsely punctulate; metanotum with weak median carina; propodeum posteriorly largely smooth, with coarse curved transverse carina in front of middle and anteriorly rugose and with rather short median carina (Fig. 420).

*Wings*. Fore wing (Fig. 418): pterostigma triangular; 1-R1 ending close to wing apex and 1.3 times as long as pterostigma; r:3-SR:SR1 = 5:16:50; 2-SR:3-SR:r-m = 16:16:5; r slender; 1-M and SR1 slightly curved; m-cu just postfurcal; cu-a slightly postfurcal and 1-CU1 hardly widened; first subdiscal cell closed, CU1b medium-sized and shorter than 3-CU1; M+CU1 sclerotized. Hind wing: M+CU:1-M:1r-m = 25:18:12; cu-a straight; m-cu absent.

*Legs*. Length of femur, tibia and basitarsus of hind leg 3.8, 7.0 and 3.7 times as long as wide, respectively (Fig. 427); hind femur with long setae and tibia densely rather short setose; third and fourth segments of fore tarsus distinctly longer than wide and about as long as wide, respectively.

*Metasoma*. Length of first tergite 0.8 times its apical width, its surface smooth in front of united dorsal carinae and coarsely punctate-reticulate behind carinae, convex and no median carina posteriorly (Fig. 421); second suture coarsely crenulate, nearly straight, slightly widened medially and distinctly impressed; second tergite with row of punctures between longitudinal striae; median length of second tergite 2.1 times median length of third tergite; third tergite mainly with rows of punctures, but medially and posteriorly smooth; following tergites smooth and largely retracted below carapace; length of setose part of ovipositor sheath 0.22 times fore wing, 0.6 times first-third tergites combined and 0.8 times longer than hind tibia; hypopygium far retracted, truncate apically and about 0.2 times as long as metasomal carapace.

*Colour*. Dark brown, including pterostigma, veins and antenna (but scapus yellow); head and mandible yellow, but head medio-dorsally and posteriorly infuscate; ovipositor sheath blackish; wing membrane subhyaline.

#### Distribution.

Oriental China (Hunan).

#### Biology.

Unknown.

### Genus Phaedrotoma

Foerster, 1862

http://species-id.net/wiki/Phaedrotoma

Figs 196 [Fig F51] [Fig F52] [Fig F53] [Fig F54] [Fig F55] [Fig F56] [Fig F57] [Fig F58] [Fig F59] [Fig F60] [Fig F61] [Fig F62] [Fig F63] [Fig F64] [Fig F65] [Fig F66] [Fig F67] [Fig F68] [Fig F69] [Fig F70] [Fig F71] –313

Phaedrotoma Foerster, 1862: 260. Type species (by original designation): *Phaedrotoma depeculator* Foerster, 1862 [examined].
Nosopoea Foerster, 1862: 260. Type species (by original designation): *Opius cingulatus* Wesmael, 1835 [examined]. **Syn. n.**
Eutrichopsis Foerster, 1862: 260. Type species (by original designation): *Eutrichopsis munda* Foerster, 1862 [examined]. **Syn. n.**
Tolbia Cameron, 1907: 85. Type species (by monotypy): *Tolbia scaevolae* Cameron, 1907. **Syn. n.**
Brachycentrus Szépligeti, 1907: 37 (nec Taschenberg, 1865). Type species (by monotypy): *Brachycentrus minutus* Szépligeti, 1907 [examined]. **Syn. n.**
Baeocentrum Schulz, 1911: 65 (new name for *Brachycentrus*). Type species (by implication): *Brachycentrus minutus* Szépligeti, 1907 [examined]. **Syn. n.**
Hexaulax Cameron, 1910: 12. Type species (by monotypy): *Hexaulax ruficeps* Cameron, 1910. **Syn. n.**
Atoreuteus ; Szépligeti, 1908: 55 (not Foerster 1862).
Coeloreuteus Roman, 1910: 112. Type species (by original designation): *Atoreuteus africanus* Szépligeti, 1908 [examined]. **Syn. n.**
Neodiospilus Szépligeti, 1911: 415; Shenefelt 1970: 216; Wharton 1987: 63 (synonymized with *Opius*). Type species (designated by Brues 1926): *Neodiospilus flavipes* Szépligeti, 1911 [examined; not *Opius flavipes* Szépligeti, 1898; = *Opius sepi-valfus* Wharton, 1987 (superfluous new name, because *Opius flavipes* Szépligeti, 1898, belongs to the genus *Opius* Wesmael s.s.); = *Aulonotus kanywareensis* Fischer, 2000 [examined]. **Syn. n.**]
Neopius Fischer, 1965: 187. Type species (by original designation): *Neopius macrops* Fischer, 1965 [examined; not *Neopius* Gahan, 1917]. **Syn. n.**
Euopius Fischer, 1967b: 959. Type species (by original designation): *Neopius macrops* Fischer, 1965 [examined]. **Syn. n.**
Gastrosema Fischer, 1972a: 353. Type species (by original designation): *Opius pumilio* Wesmael, 1835 [examined]. **Syn. n.**
Gerius Fischer, 1972a: 389. Type species (by original designation): *Opius fossulatus* Szépligeti, 1914. **Syn. n.**
Grimnirus Fischer, 1972b: 70. Type species (by original designation): *Opius fuscicarpus* Szépligeti, 1913. **Syn. n.**
Hoenirus Fischer, 1972b: 69. Type species (by original designation): *Opius punctulatus* Szépligeti, 1914 [examined; vein 3-CU1 of fore wing subhorizontal and vein CU1b largely reduced]. **Syn. n.**
Mimirus Fischer, 1972b: 70 Type species (by original designation): *Opius peregrinus* Szépligeti, 1914. [examined; clypeus with small medio-ventral tooth]. **Syn. n.**
Merotrachys Fischer, 1972a: 406. Type species (by original designation): *Opius laetatorius* Fischer, 1958 [examined;. **Syn. n.**
Phlebosema Fischer, 1972a: 350. Type species (by original designation): *Opius discreparius* Fischer, 1963. **Syn. n.**
Neoephedrus Samanta, Tamili, Saha & Raychaudhuri,1983: 93. Type species (by original designation): *Neoephedrus helichrysi* Samanta, Tamili, Saha & Raychaudhuri,1983 [examined]. **Syn. n.**
Adontopius Fischer, 1984b: 93. Type species (by original designation): *Opius adentatus* Fischer, 1980. **Syn. n.**
Serius Tobias & Jakimavičius, 1986: 23. Miswriting for *Gerius* Fischer, 1972.
Kainopaeopius Fischer, 1986: 609. Type species (by original designation): *Opius crassicrus* Thomson, 1895 [examined]. **Syn. n.**
Millenniopius Fischer, 1996: 675. Type species (by original designation): *Opius inflatipectus* Fischer, 1996 [examined]. **Syn. n.**
Neotropopius Fischer, 1999: 282. Type species (by original designation): *Opius hirtithorax* Fischer, 1963. **Syn. n.**

#### Diagnosis.

Face without tubercles; in front of anterior ocellus without distinct depression; frons without pair of distinct depressions above antennal sockets, but whole frons may be depressed; occipital carina present laterally, not or slightly curved ventrally and remain removed from hypostomal carina (Figs 243, 253), near level of middle of eye straight or nearly so, without transverse carina or crest; clypeus more or less convex and comparatively high; hypoclypeal depression variable; labrum normal, without emargination ventrally; mandible normal, gradually widened basally, at most with protruding carina; scapus, fore coxa and trochanter at most weakly compressed; epistomal suture without large depressions; medio-posterior depression of mesoscutum variable; pronope round or wide elliptical, or pronotum only with a shallow transverse groove; scutellar sulcus usually rather wide; propodeum usually smooth or superficially sculptured; postpectal carina completely absent; vein 2-SR of fore wing present, rarely absent; first subdiscal cell of fore wing at least partly closed by vein 3-CU1 postero-apically (Fig. 197); vein 1-M of fore wing usually straight; vein cu-a of hind wing nearly always present; vein 3-SR of fore wing distinctly longer than vein 2-SR; if subequal then vein m-cu of hind wing or precoxal sulcus (almost) absent; length of fore wing usually less than 3.5 mm; second and basal half of third tergite without sharp lateral crease, if sometimes weakly developed then second tergite smooth; length of second and third tergites combined less than 0.7 times length of metasoma behind first tergite; fourth and following tergites (at least partly) exposed; ovipositor sheath more or less setose basally.

#### Biology.

Parasitoids of mining Agromyzidae, Tephritidae, Ephydridae, Anthomyiidae, Scathophagidae, and Drosophilidae.

#### Distribution.

Cosmopolitan.

#### Notes.

“Taxonomic dustbin” genus of the Opiinae; if there are no obvious aut-apomorphies (except for the lack of a dorsope) species are included in this genus. The species share a straight occipital carina ventrally, the symmetrical and basally gradually widened mandibles, the lack of a dorsope, the gradually widened or parallel-sided first tergite and the vein m-cu of the fore wing angled with neighbouring veins. It is most likely that this is not a monophyletic assemblage and is in need of future study.

### Phaedrotoma acuticlypeata

Li & van Achterberg sp. n.

urn:lsid:zoobank.org:act:E8CD890F-0F07-4A45-ADC0-7F51D726C7AE

http://species-id.net/wiki/Phaedrotoma_acuticlypeata

Figs 196 –204

#### Type material.

Holotype, ♀ (ZUH), “S. China: Hunan, nr Zhangjiajie, Badagong Mts, Longtanping, 4–5.VI.2009, 550 m, Xi-Ying Li, RMNH’09”. Paratypes (RMNH): 1 ♀, “S. China: Hunan, Changsha, garden Hunan Agr. Univ., 80 m, 31.V.2009, Xi-Ying Li, RMNH’09”; 1 ♂, “S. China: Hunan, nr Suining, Huangsang N. R., Shaoyang, 12–13.VI.2009, 1000 m, Xi-Ying Li, RMNH’09”; 1 ♀, “S. China: Hunan, nr Chengbu, Nan Mt., Shaoyang, 1500 m, 10-11.VI.2009, Xi-Ying Li, RMNH’09”.

#### Diagnosis.

**Figure 196.**
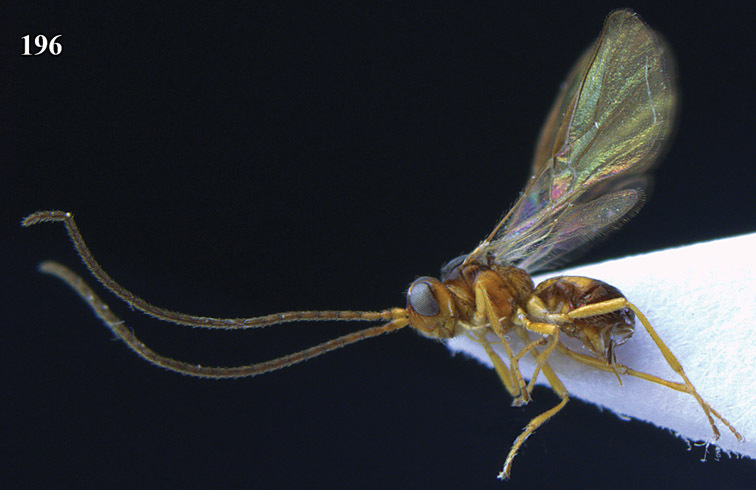
*Phaedrotoma acuticlypeata* sp. n., female, holotype. Habitus lateral.

**Figures 197–204. F51:**
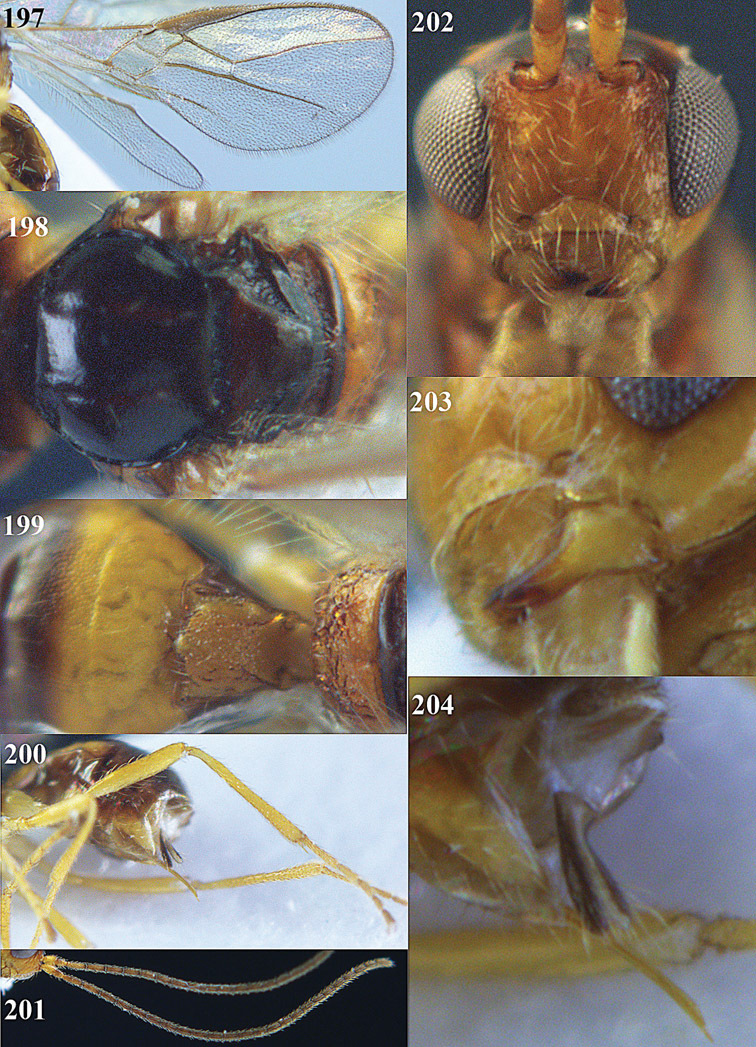
*Phaedrotoma acuticlypeata* sp. n., female, holotype. **197** Wings **198** mesosoma dorsal **199** propodeum and 1^st^-3^rd^ metasomal tergites dorsal **200** hind leg **201** antennae **202** head anterior **203** mandible **204** ovipositor sheath.

Length of mesosoma 1.2 times its height; antenna of female 1.5–1.7 times as long as fore wing (male: 1.5–1.6 times); length of eye in dorsal view about 2.8 times temple; clypeus convex medially (Fig. 202); propodeum largely coarsely rugose except anteriorly (Fig. 199); pronotal side and mesopleuron superficially granulate; precoxal sulcus wide and comparatively shallow, densely finely sculptured (Fig. 196); hind tarsus slender and pale yellowish as femur (Fig. 200); vein SR1 of fore wing 2.9–3.4 times as long as vein 3-SR (Fig. 197).

#### Description.

Holotype, ♀, length of body 1.6 mm, of fore wing 2.0 mm.

*Head*. Antenna with 26 segments and 1.6 times as long as fore wing; length of third segment 1.1 times fourth segment, length of third, fourth and penultimate segments 3.7, 3.5, and 2.5 times their width, respectively (Fig. 201); length of maxillary palp 0.9 times height of head; labial palp segments rather moniliform; occipital carina distinctly removed from hypostomal carina and dorsally absent; hypostomal carina medium-sized; length of eye in dorsal view 2.8 times temple; frons glabrous, very superficially granulate and strongly shiny and with pit medially, slightly convex laterally and in front of anterior ocellus; face largely smooth but laterally superficially granulate, medially slightly elevated (Fig. 202); width of clypeus 2.5 times its maximum height and 0.5 times width of face; clypeus weakly convex, ventrally protruding forwards, smooth and its ventral margin sharp and straight (Fig. 202); hypoclypeal depression medium-sized (Fig. 202); malar suture partly narrowly impressed (Fig. 203); mandible gradually widened basally, with narrow and non-protruding ventral carina (Fig. 203).

*Mesosoma*. Length of mesosoma 1.2 times its height; dorsal pronope rather large and round; pronotal side superficially granulate, oblique groove crenulate anteriorly and posterior groove largely absent (Fig. 196); epicnemial area superficially granulate dorsally; precoxal sulcus only medially impressed, wide and comparatively shallow, densely finely sculptured (Fig. 196); remainder of mesopleuron and pleural sulcus smooth; anterior groove of metapleuron crenulate; notauli absent on disc, only anteriorly indicated by shallow depressions; mesoscutum glabrous except for a few setae along imaginary notaulic courses (Fig. 198); medio-posterior depression of mesoscutum absent; lateral carina of mesoscutum present; scutellar sulcus moderately crenulate; scutellum smooth or nearly so and flattened; anterior surface of propodeum short and largely smooth, remainder coarsely rugose, without carinae (Fig. 199).

*Wings*. Fore wing (Fig. 197): pterostigma elliptical, narrowed apically; 1-R1 reaching wing apex and 1.4 times as long as pterostigma; r:3-SR:SR1 = 2:22:64; r slender; 2-SR:3-SR:r-m = 12:22:6; 1-M straight; SR1 slightly curved; m-cu distinctly postfurcal; cu-a just postfurcal and 1-CU1 widened; first subdiscal cell closed, CU1b short. Hind wing (Fig. 197): M+CU:1-M:1r-m = 10:13:5; cu-a straight; m-cu absent.

*Legs*. Length of femur, tibia and basitarsus of hind leg 5.2, 9.2 and 5.5 times as long as wide, respectively; hind femur with long setae and of tibia medium-sized (Fig. 200).

*Metasoma*. Length of first tergite 1.1 times its apical width, its surface weakly and gradually convex and largely finely and densely rugulose, dorsal carinae developed in its anterior 0.6, straight (Fig. 199); second and third tergites superficially granulate, division of tergites slightly elevated; length of ventrally visible setose part of ovipositor sheath 0.06 times fore wing and 0.2 times hind tibia; apex of hypopygium rather acute (Figs 196, 204).

*Colour*. Brownish-yellow; palpi, humeral plate and hind trochantellus ivory; legs pale yellowish (including hind tibial apex and hind tarsus, but telotarsi infuscate); antenna (but scapus yellowish), tegulum, head dorsally (but frons yellowish latero-posteriorly), mesoscutum, scutellum and metanotum, apex of third tergite and following tergites, ovipositor sheath, pterostigma and veins dark brown; wing membrane subhyaline.

*Molecular data*. None.

*Variation*. Length of body 1.6–1.9 mm, of fore wing 1.9–2.1 mm; antenna of female with 26 (1), 27 (1) or 28 (1) segments, of male with 31 (1) segments; oblique groove of pronotum distinctly or superficially crenulate.

#### Distribution.

*China (Hunan).

#### Biology.

Unknown.

#### Etymology.

Name derived from “acutus” (Latin for “sharp”) and clypeus, because of the acute ventral margin of the clypeus.

#### Notes.

The new species runs in the key by Chen and Weng (2005) to *Phaedrotoma improcera* (Weng & Chen, 2005) comb. n. *Phaedrotoma acuticlypeata* differs by having the anterior surface of the propodeum largely smooth (rugose in *Phaedrotoma improcera*), vein 3-SR of fore wing 1.8 times vein 2-SR (1.3 times) and length of eye in dorsal view 2.8 times temple (1.7 times).

### Phaedrotoma angiclypeata

Li & van Achterberg sp. n.

urn:lsid:zoobank.org:act:1888DD51-4108-436B-909A-BB3649406EA9

http://species-id.net/wiki/Phaedrotoma_angiclypeata

Figs 205 –214

#### Type material.

Holotype, ♀ (ZUH), “S. China: Hunan, nr Zhangjiajie, Badagong Mts, Longtanping, 4–5.VI.2009, 550 m, Xi-Ying Li, RMNH’09”. Paratype (RMNH): 1 ♂, “S. China: Hunan, nr Suining, Huangsang N. R., Shaoyang, 12–13.VI.2009, 1000 m, Xi-Ying Li, RMNH’09”.

#### Diagnosis.

**Figure 205. F52:**
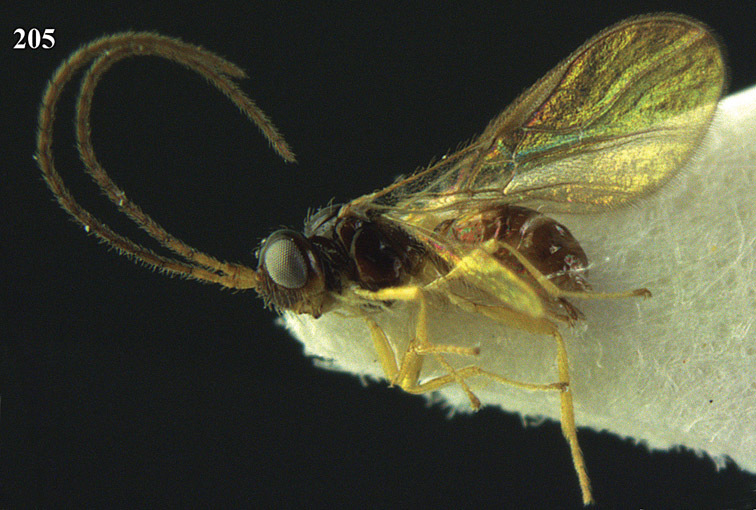
*Phaedrotoma angiclypeata* sp. n., female, holotype. Habitus lateral.

**Figures 206–214. F53:**
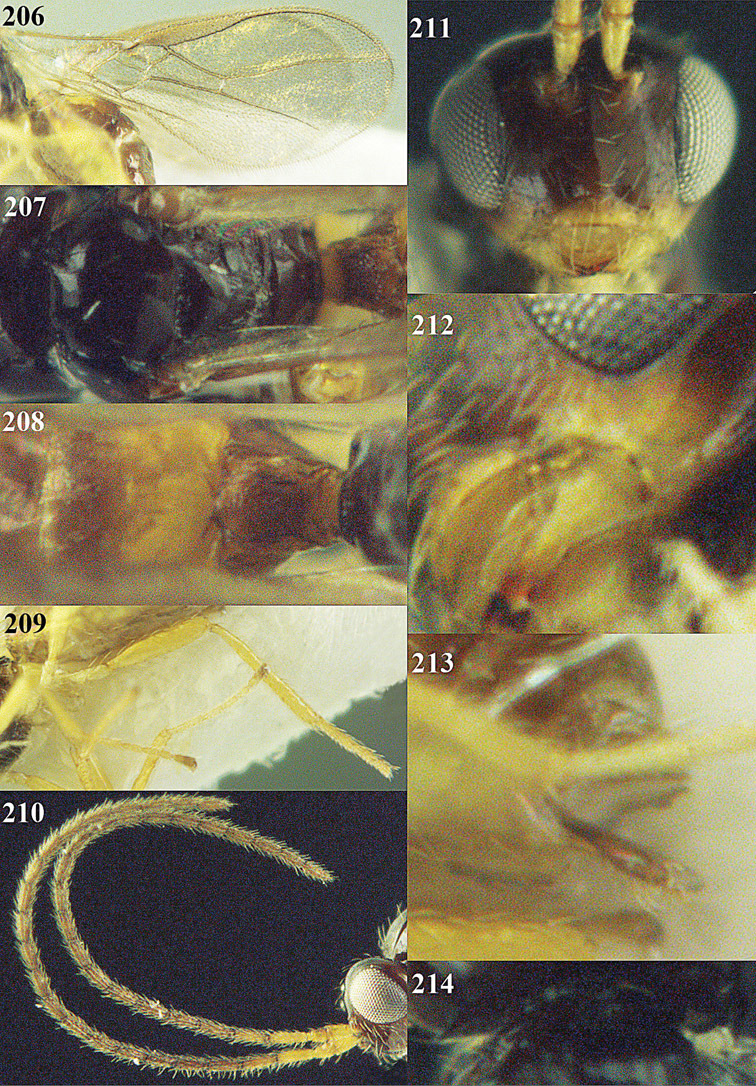
*Phaedrotoma angiclypeata* sp. n., female, holotype. **206** Wings **207** mesosoma dorsal **208** propodeum and 1^st^-3^rd^ metasomal tergites dorsal **209** hind leg **210** antennae **211** head anterior **212** mandible **213** ovipositor sheath **214** pronope dorsal.

Hypopygium of ♀ truncate apically and 0.2 times as long as metasoma (Fig. 213); clypeus normal ventrally and semicircular (Fig. 212); clypeus 0.6 times as wide as face or less, protruding medio-ventrally and truncate laterally (Fig. 211); occipital carina close to hypostomal carina; malar suture entirely absent; medio-posterior depression of mesoscutum absent; scutellar sulcus at most moderately crenulate; scutellum flat or nearly so; posterior groove of propleuron smooth or absent; pronope round and rather large (Fig. 214); lateral carina of mesoscutum largely absent; mesosoma largely black; precoxal sulcus finely crenulate or smooth (Fig. 205); propodeum smooth and shiny; vein m-cu of fore wing slightly postfurcal (Fig. 206); vein CU1b shorter that vein 3-CU1 (Fig. 206); hind femur about 5 times as long as wide; second and third metasomal tergites smooth; head and mesosternum largely dark brown; mandible gradually widened basally and narrow apically (Fig. 212); hind tarsus pale yellowish.

#### Description.

Holotype, ♀, length of body 1.4 mm, of fore wing 1.7 mm.

*Head*. Antenna with 23 segments and 1.3 times as long as fore wing; length of third segment 1.1 times fourth segment, length of third, fourth and penultimate segments 4.3, 3.8, and 3.0 times their width, respectively (Fig. 210); length of maxillary palp 0.9 times height of head; labial palp segments rather short; occipital carina close to hypostomal carina and dorsally absent; hypostomal carina narrow; length of eye in dorsal view 2.6 times temple; frons glabrous, smooth, evenly flattened; face smooth, medially weakly elevated; width of clypeus 3.1 times its maximum height and 0.55 times width of face; clypeus convex, protruding forwards, superficially punctate dorsally and its ventral margin thin and slightly concave (Fig. 211); hypoclypeal depression rather large (Fig. 211); malar suture entirely absent; mandible gradually widened basally, with short and non-protruding ventral carina (Fig. 212).

*Mesosoma*. Length of mesosoma 1.1 times its height; dorsal pronope distinct, rather large, round (Fig. 214); pronotal side smooth, but slightly crenulate in oblique groove (Fig. 205); epicnemial area smooth dorsally; precoxal sulcus only medially impressed, smooth as rest of mesopleuron (Fig. 205); pleural sulcus smooth as anterior groove of metapleuron; notauli absent on disc, only anteriorly indicated by shallow depressions (Fig. 207); meso-scutum glabrous except for a few setae; medio-posterior depression of mesoscutum absent; lateral carina of mesoscutum largely absent; scutellar sulcus moderately crenulate; scutellum smooth and flattened; surface of propodeum entirely smooth and shiny (Fig. 207).

*Wings*. Fore wing (Fig. 206): pterostigma elliptical, narrowed apically; 1-R1 reaching wing apex and 1.3 times as long as pterostigma; r:3-SR:SR1 = 2:18:53; 2-SR:3-SR:r-m = 11:18:6; r slender; 1-M straight; SR1 slightly sinuate; m-cu slightly postfurcal; cu-a postfurcal and 1-CU1 widened; first subdiscal cell closed, CU1b rather short. Hind wing (Fig. 206): M+CU:1-M:1r-m = 10:13:5; cu-a straight; m-cu absent.

*Legs*. Length of femur, tibia and basitarsus of hind leg 5.0, 8.5 and 4.5 times as long as wide, respectively; hind femur with long setae and of tibia medium-sized (Fig. 209).

*Metasoma*. Length of first tergite 1.2 times its apical width, its surface distinctly convex but flattened medially and largely finely rugose or rugulose, dorsal carinae developed in its anterior 0.6, curved (Fig. 208); second and following tergites largely smooth, but with some superficial granulation, division of tergites slightly elevated; length of ventrally visible setose part of ovipositor sheath 0.06 times fore wing and 0.2 times hind tibia (Figs 205, 213).

*Colour*. Black; palpi, mandible, clypeus, malar space, second tergite, metasoma ventrally and legs pale yellowish; first tergite yellowish-brown; antenna (but scapus, laterally pedicellus and third segment yellowish), face, temple, mesopleuron largely, ovipositor sheath, remainder of metasoma, pterostigma and veins dark brown; wing membrane subhyaline.

*Molecular data*. None.

*Variation*. Paratype has length of the fore wing 2.0 mm, the antenna with 27 segments, precoxal sulcus finely crenulate and the head largely brownish-yellow.

#### Distribution.

*China (Hunan).

#### Biology.

Unknown.

#### Etymology.

Name derived from “angustus” (Latin for “narrow”) and clypeus, because of the comparatively narrow clypeus.

#### Notes.

The new species runs in the key by Chen and Weng (2005) to *Opius clusilis* Weng & Chen, 2005, if the second tergite is considered to be smooth. *Phaedrotoma angiclypeata* differs by having the head roundly narrowed behind the eyes in dorsal view (directly narrowed in *Opius clusilis*), the mandible hardly widened basally (mandible distinctly widened), length of the first tergite 1.2 times its apical width (1.4 times) and length of the third antennal segment 4.5 times its width (2.6 times). The new species is close to *Phaedrotoma postuma* (Chen & Weng, 2005) comb. n., but *Phaedrotoma postuma* has the head (except usually dorsally) and mesosoma laterally yellowish-brown or brown, the third antennal segment comparatively slender, the fourth antennal segment largely yellowish-brown, the malar space slightly longer and the ovipositor sheath narrow.

### Phaedrotoma antenervalis

Li & van Achterberg sp. n.

urn:lsid:zoobank.org:act:A03369EE-6DE9-44C2-B288-F668079819C4

http://species-id.net/wiki/Phaedrotoma_antenervalis

Figs 215 –224

#### Type material.

Holotype, ♀ (ZUH), “S. China: Hunan, nr Zhangjiajie, Forest park Mts, 1.VIII.1989, Ben-Zhu Dai, No. 214”.

#### Diagnosis.

**Figure 215. F54:**
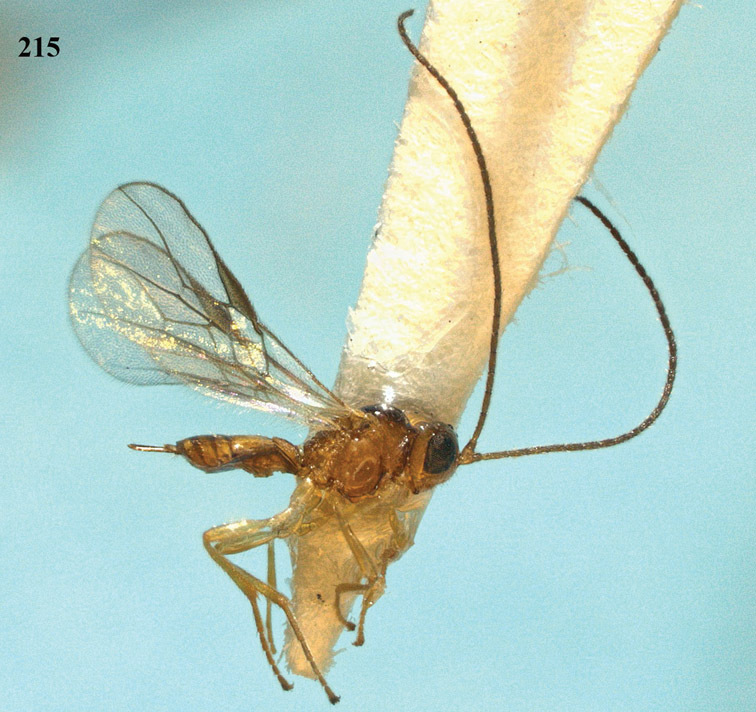
*Phaedrotoma anternervalis* sp. n., female, holotype. Habitus lateral.

**Figures 216–224. F55:**
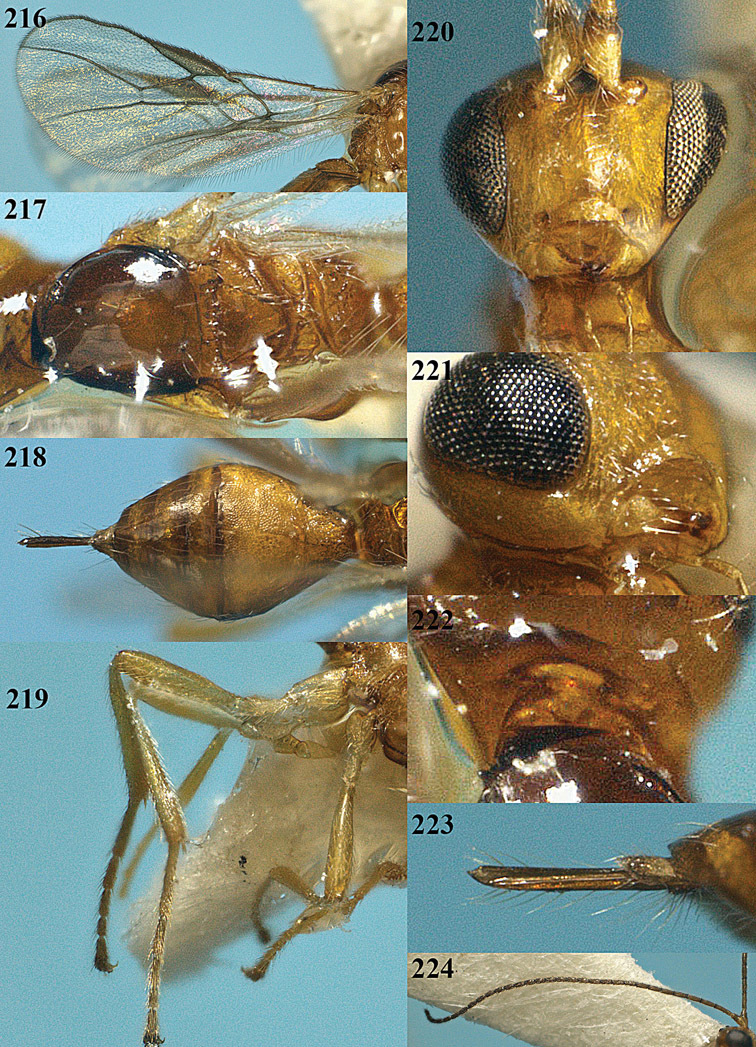
*Phaedrotoma antenervalis* sp. n., female, holotype. **216** Wings **217** mesosoma dorsal **218** propodeum and metasoma dorsal **219** hind leg **220** head anterior **221** mandible **222** pronope dorsal **223** ovipositor sheath **224** antenna.

Clypeus normal ventrally and semi-circular (Fig. 221); width of clypeus 2.8 times its maximum height; mesosoma yellowish-brown laterally and ventrally; pronotal side smooth; precoxal sulcus somewhat wider, in elliptical depression; anterior groove of metanotum crenulate (Fig. 215); propodeum usually largely rugose and medio-longitudinal carina absent (Fig. 217); vein SR1 of fore wing 2.6 times as long as vein 3-SR; vein m-cu of fore wing antefurcal (Fig. 216); vein 1r-m of hind wing distinctly oblique and 0.4 times vein 1-M (Fig. 216); second tergite densely and finely granulate-punctate (Fig. 218); setose part of ovipositor sheath 0.45 times as long as hind tibia (Fig. 223).

#### Description.

Holotype, ♀, length of body 1.8 mm, of fore wing 2.3 mm.

*Head*. Antenna with 32 segments and 1.4 times as long as fore wing; length of third segment 1.3 times fourth segment, length of third, fourth and penultimate segments 4.8, 3.8, and 2.0 times their width, respectively (Fig. 224); length of maxillary palp 0.5 times height of head; labial palp segments short; occipital carina moderately close to hypostomal carina (Fig. 221) and dorsally absent; hypostomal carina narrow; length of eye in dorsal view 2.0 times temple; frons glabrous, smooth, evenly flattened; face smooth, medially weakly elevated; width of clypeus 2.8 times its maximum height and 0.5 times width of face; clypeus convex, protruding forwards, superficially punctate dorsally and its ventral margin thin and slightly concave (Figs 220, 221); hypoclypeal depression large (Fig. 220); malar suture absent; mandible gradually widened basally and with a narrow ventral carina (Fig. 221).

*Mesosoma*. Length of mesosoma 1.2 times its height; dorsal pronope large and round (Fig. 222); pronotal side smooth and posterior groove absent (Fig. 215); epicnemial area smooth dorsally; precoxal sulcus only medially impressed, widely and finely crenulate, smooth as rest of mesopleuron (Fig. 215); pleural sulcus smooth; anterior groove of metapleuron crenulate; notauli absent on disc, only anteriorly indicated by shallow depressions (Fig. 217); mesoscutum smooth and glabrous; medio-posterior depression of mesoscutum absent; lateral carina of mesoscutum largely absent; scutellar sulcus moderately crenulate; scutellum smooth and flattened; surface of propodeum largely rugose (Fig. 217).

*Wings*. Fore wing (Fig. 216): pterostigma elliptical, narrowed apically; 1-R1 reaching wing apex and 1.3 times as long as pterostigma; r:3-SR:SR1= 3:31:80; 2-SR:3-SR:r-m = 17:31:7; r normal; 1-M slightly curved; SR1 slightly sinuate; m-cu antefurcal; cu-a postfurcal and 1-CU1 widened; first subdiscal cell closed, CU1b short. Hind wing (Fig. 216): M+CU:1-M:1r-m = 18:24:10; cu-a straight; m-cu absent.

*Legs*. Length of femur, tibia and basitarsus of hind leg 3.8, 7.1 and 6.5 times as long as wide, respectively; hind femur with long setae and of tibia medium-sized (Fig. 219).

*Metasoma*. Length of first tergite about equal to its apical width, its surface evenly gradually convex medially, longitudinally rugose and with dorsal carinae remain separated from each other and reaching apex of tergite (Fig. 218); second tergite densely and finely granulate-punctate (Fig. 218); third and following tergites smooth; length of ventrally visible setose part of ovipositor sheath 0.11times fore wing and 0.45 times hind tibia (Figs 215, 223).

*Colour*. Yellow; antenna, mesosoma laterally and ventrally, third and following tergites and ovipositor sheath yellowish-brown; pterostigma and veins brown; wing membrane subhyaline.

*Molecular data*. None.

#### Distribution.

*China (Hunan).

#### Biology.

Unknown.

#### Etymology.

Name derived from “ante” (Latin for “before”) and “nervus” (Latin for nerve or vein), because of the antefurcal vein m-cu of fore wing.

#### Notes.

The new species runs in the key by Chen and Weng (2005) to *Phaedrotoma osculas* (Weng & Chen, 2005) comb. n. *Phaedrotoma antenervalis* differs by having the setose part of ovipositor sheath distinctly longer than the first tergite (0.8 times in *Phaedrotoma osculas*), length of the maxillary palp about equal to height of head (1.2 times) and the propodeum densely rugulose anteriorly (smooth).

### Phaedrotoma depressa

Li & van Achterberg nom. n.

Figs 225 –236

Opius vittata [*recte: vittatus*] Chen & Weng, 2005: 106–107, fig. 44, 180, 198, photos 166–170 (not *Opius vittatus* Ruschka, 1915; junior homonym).

#### Type material.

Holotype of *Opius vittata*, ♀ (FAFU), “[China:] Fujian, Jiangle, Longqi Mt., 10.VII.1994, Wu Zhi-shan” and 3 ♀ + 1 ♂ topotypic paratypes collected 10–14.VII.1994.

#### Additional material.

4 ♀ (RMNH, ZUH), “S. China: Hunan, Changsha, garden Hunan Agr. Univ., 80 m, 31.V.2009, Xi-Ying Li, RMNH’09”; 2 ♀, “S. China: Hunan, nr Zhangjiajie, Badagong Mts, Tian Ping Mt., 9-13.VII.2009, 550 m, Xi-Ying Li, RMNH’10”; 1 ♀, “S. China: Hunan, nr Zhangjiajie, Badagong Mts, Bamaoxi, 2-3.VI.2009, 540 m, Xi-Ying Li, RMNH’09”.

#### Diagnosis.

Vein SR1 of fore wing 3.4-4.0 times as long as vein 3-SR; clypeus depressed ventrally and narrow sickle-shaped (Fig. 233); mesosoma (except black meso-scutum) orange-brown; second and third metasomal tergite micro-sculptured and propodeum smooth; hypopygium of ♀ obtuse apically or nearly so and 0.1–0.2 times as long as metasoma; labrum normal, without large space below clypeus (Fig. 233); hind femur about 4.5 times as long as wide.

#### Description.

Specimen from Changsha, ♀, length of body 1.2 mm, of fore wing 1.4 mm.

*Head*. Antenna with 23 segments and 1.4 times as long as fore wing; third segment 1.1 times as long as fourth segment, length of third, fourth and penultimate segments 3.8, 3.5 and 2.5 times their width, respectively (Fig. 236); length of maxillary palp 0.8 times height of head; labial palp segments moniliform; occipital carina moderately far removed from hypostomal carina (Fig. 234) and dorsally absent; hypostomal carina narrow; length of eye in dorsal view 1.8 times temple; frons slightly evenly convex and glabrous, smooth; face smooth, medially hardly elevated (Fig. 233); width of cly-peus 3.5 times its maximum height and 0.6 times width of face, depressed ventrally and sickle-shaped (Fig. 233); clypeus slightly convex, distinctly protruding forwards, smooth except for a few punctures and its ventral margin thick and slightly concave; hypoclypeal depression wide (Fig. 233); malar suture shallow, linear; mandible slender triangular and somewhat narrowed submedially, with a narrow ventral carina (Fig. 234).

**Figure 225–227. F56:**
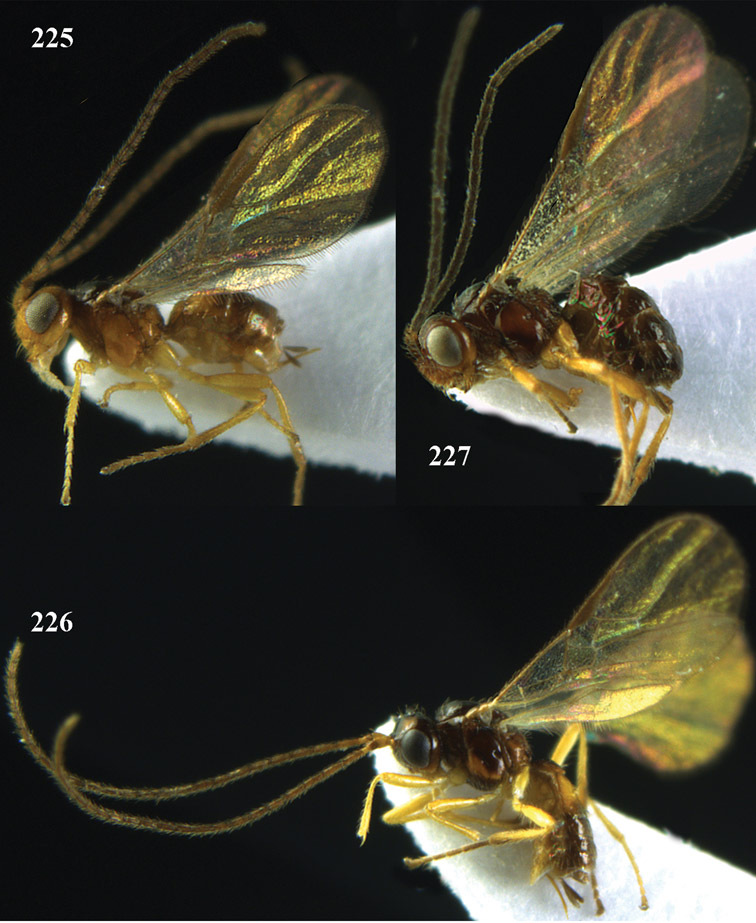
*Phaedrotoma depressa* nom. n., female, **225** Changsha and Badagong Mts, respectively. **225–227** Habitus lateral.

*Mesosoma*. Length of mesosoma 1.1 times its height; dorsal pronope obsolescent, pit-shaped; pronotal side smooth and posterior groove absent; epicnemial area smooth dorsally; precoxal sulcus medially superficially impressed, smooth as rest of mesopleuron; pleural sulcus smooth (Figs 225, 226, 227); mesosternal sulcus deep and narrow and smooth; notauli absent on disc, only anteriorly with pair of short smooth impressions (Fig. 230); mesoscutum glabrous and strongly shiny; medio-posterior depression of mesoscutum absent; scutellar sulcus narrow and finely crenulate; scutellum slightly convex medially; surface of propodeum smooth, except for crenulae posteriorly (Fig. 230).

**Figures 228–236. F57:**
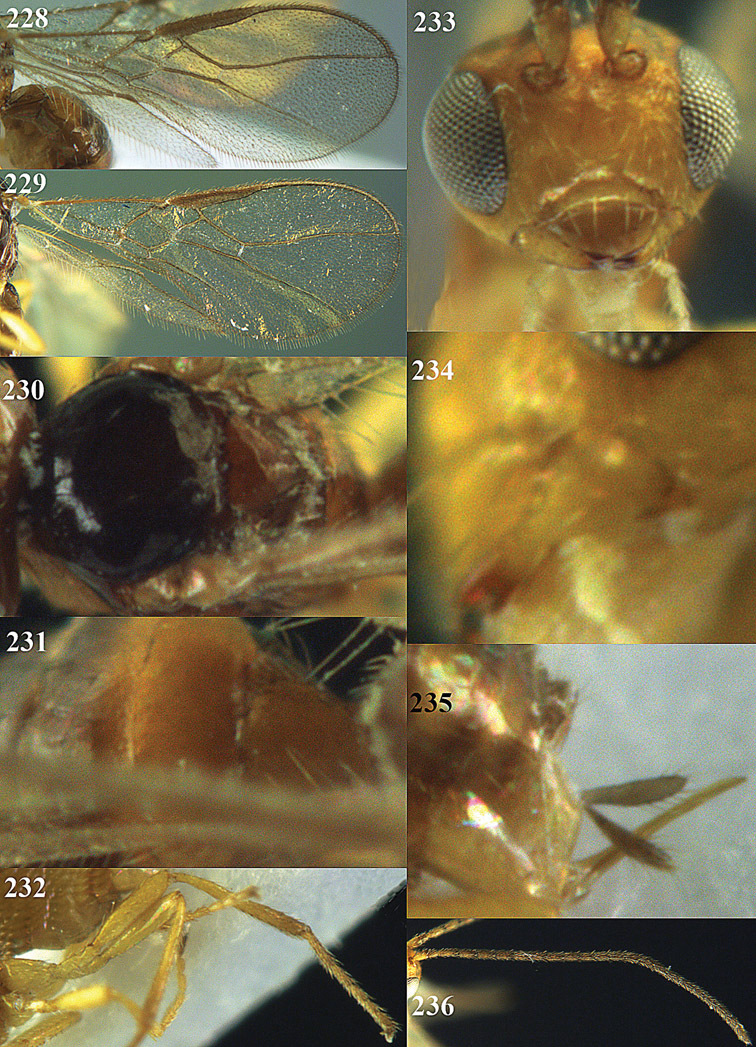
*Phaedrotoma depressa* nom. n., female, Changsha, but 229 Badagong Mts. **228, 229** Wings **230** mesosoma dorsal **231** 1^st^-3^rd^ metasomal tergites dorsal **232** hind leg **233** head anterior **234** mandible **235** ovipositor sheath **236** antenna.

*Wings*. Fore wing (Figs 228, 229): pterostigma elongate elliptical; 1-R1 reaching wing apex and 1.1 times as long as pterostigma; r:3-SR:SR1 = 2:12:48; 2-SR:3-SR:r-m = 8:12:4; r slender; 1-M straight and SR1 nearly straight; m-cu and cu-a slightly postfurcal; 1-CU1 hardly widened; first subdiscal cell open, CU1b absent; apical fifth of M+CU1 sclerotized. Hind wing (Fig. 229): M+CU:1-M:1r-m = 2:4:1; cu-a straight; m-cu absent; basal cell very narrow.

*Legs*. Length of femur, tibia and basitarsus of hind leg 4.5, 7.3 and 4.5 times as long as wide, respectively; hind femur and tibia with medium-sized setae (Fig. 232).

*Metasoma*. Length of first tergite 0.9 times its apical width, its surface flattened and coriaceous, matt and dorsal carinae developed in basal 0.4 of tergite (Fig. 231); second suture obsolescent; second and following tergites smooth; length of setose part of ovipositor sheath 0.07 times fore wing and 0.2 times length of hind tibia (Fig. 235).

*Colour*. Yellowish-brown; antenna (but scapus yellowish), stemmaticum, mesoscutum, pronotum dorsally, ovipositor sheath, third and following tergites, pterostigma and veins dark brown; palpi, mandible, tegulae and legs (but hind tibia apically, hind tarsus and telotarsi darkened) pale yellow; scutellum and metanotum brown; wing membrane subhyaline.

*Variation*. Length of body 1.2-1.4 mm, of fore wing 1.4-1.6 mm, antenna of ♀ with 23 (1) segments; vein SR1 of fore wing 3.4-4.0 times as long as vein 3-SR; thirds tergite smooth or superficially granulate; metasoma sometimes nearly completely yellowish-brown dorsally; melanistic specimens with body largely dark or chestnut brown occur (Figs 226, 227).

*Molecular data*. COI, 16S, 28S (CVA4239).

#### Distribution.

*China (Hunan).

#### Biology.

Unknown.

#### Etymology.

Name derived from “depressus” (Latin for “pressed down”), because of the narrow depressed clypeus.

#### Notes.

*Phaedrotoma depressa* runs in the key by Chen and Weng (2005) to *Opius clusilis* Weng & Chen, 2005. *Phaedrotoma depressa* differs by having the mandible gradually narrowed and nearly symmetrical (abruptly narrowed and asymmetrical in *Opius clusilis*), the head roundly narrowed behind eyes in dorsal view (directly narrowed), the mandible hardly widened basally (mandible distinctly widened), length of the first tergite 0.9 times its apical width (1.4 times) and length of the third antennal segment 3.8 times its width (2.6 times). *Phaedrotoma rugulosa* (Chen & Weng, 2005) comb. n. comes very close but has a higher clypeus (narrower), slenderer third and fourth antennal segments of ♀, T3-6 membranous and depressed posteriorly. Examination of the holotype of *Opius vittatus* Chen & Weng, 2005 (a junior homonym) showed that this is the same species as *Phaedrotoma depressa* despite it is not keying out to it.

### Phaedrotoma depressiclypealis

Li & van Achterberg sp. n.

urn:lsid:zoobank.org:act:D23DB5DB-6C0C-40B8-A31B-E18D5C6D7CB2

http://species-id.net/wiki/Phaedrotoma_depressiclypealis

Figs 237 –246

#### Type material.

Holotype, ♀ (ZUH), “S. China: Hunan, nr Zhangjiajie, Badagong Mts, Bamaoxi, 2–3.VI.2009, 540 m, Xi-Ying Li, RMNH’09”.

#### Diagnosis.

Malar suture partly shallowly impressed (Fig. 243); clypeus medium-sized (Fig. 242); second submarginal cell of fore wing medium-sized (Fig. 238); setose part of ovipositor sheath 0.1–0.2 times as long as hind tibia; pronope, sculpture of propodeum and second and third tergites variable; first tergite mainly rugose and granulate (Fig. 240); anterior groove of metapleuron crenulate; apical half of first metasomal tergite subparallel (Fig. 240); occipital carina comparatively close to hypostomal carina; clypeus largely subparallel-sided, densely punctate and medially depressed (Fig. 242); length of mesosoma 1.5 times its height; antenna of female about 1.3 times as long as fore wing; length of eye in dorsal view about 3.5 times temple; propodeum coarsely rugose (Fig. 240).

#### Description.

Holotype, ♀, length of body 2.1 mm, of fore wing 2.5 mm.

*Head*. Antenna with 29 segments and 1.3 times as long as fore wing; length of third segment 1.3 times fourth segment, length of third, fourth and penultimate segments 4.0, 3.0, and 3.0 times their width, respectively malar suture partly shallowly impressed (Fig. 244); length of maxillary palp 0.9 times height of head; labial palp segments normal, elongate; occipital carina moderately far removed from hypostomal carina and dorsally absent (Fig. 243); hypostomal carina rather narrow; length of eye in dorsal view 3.5 times temple; frons glabrous, smooth and strongly shiny and with shallow longitudinal depression medially, slightly convex; face largely smooth, medially slightly elevated; width of clypeus twice its maximum height and 0.5 times width of face; clypeus rather flattened and superficially sculptured, ventrally depressed, not protruding forwards and its ventral margin obtuse and slightly concave malar suture partly shallowly impressed (Fig. 242); hypoclypeal depression medium-sized (Fig. 242); malar suture partly superficially impressed near eye (Fig. 243); mandible gradually widened basally, with narrow and non-protruding ventral carina (Fig. 243).

**Figure 237. F58:**
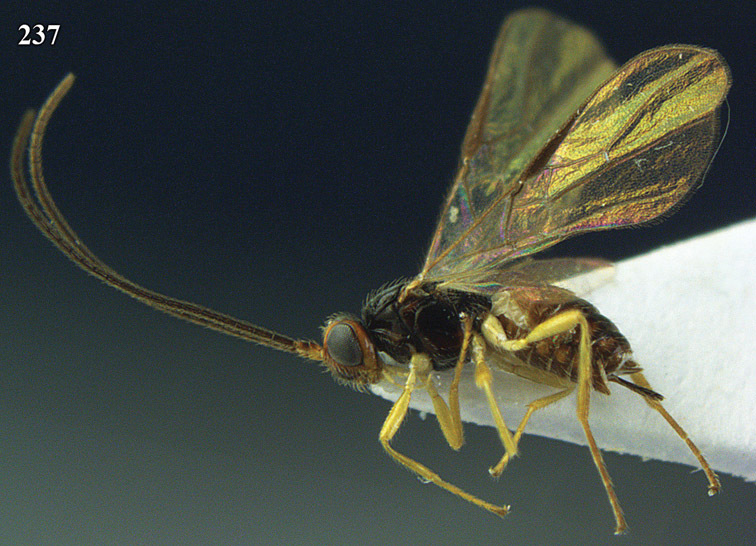
*Phaedrotoma depressiclypealis* sp. n., female, holotype. Habitus lateral.

*Mesosoma*. Length of mesosoma 1.5 times its height; dorsal pronope large and round (Fig. 246); pronotal side smooth and posterior groove largely absent; epicnemial area smooth; precoxal sulcus only medially impressed, narrow and comparatively deep, only with some micro-sculpture (Fig. 237); pleural sulcus smooth; anterior groove of metapleuron crenulate ventrally; notauli absent on disc, only anteriorly indicated by shallow depressions; mesoscutum glabrous except for a few setae along imaginary notaulic courses (Fig. 239); medio-posterior depression of mesoscutum absent; lateral carina of mesoscutum largely absent anteriorly; scutellar sulcus finely crenulate; scutellum smooth or nearly so and flattened; surface of propodeum rather coarsely rugose, without distinct transverse or median carinae (Fig. 239).

**Figures 238–246. F59:**
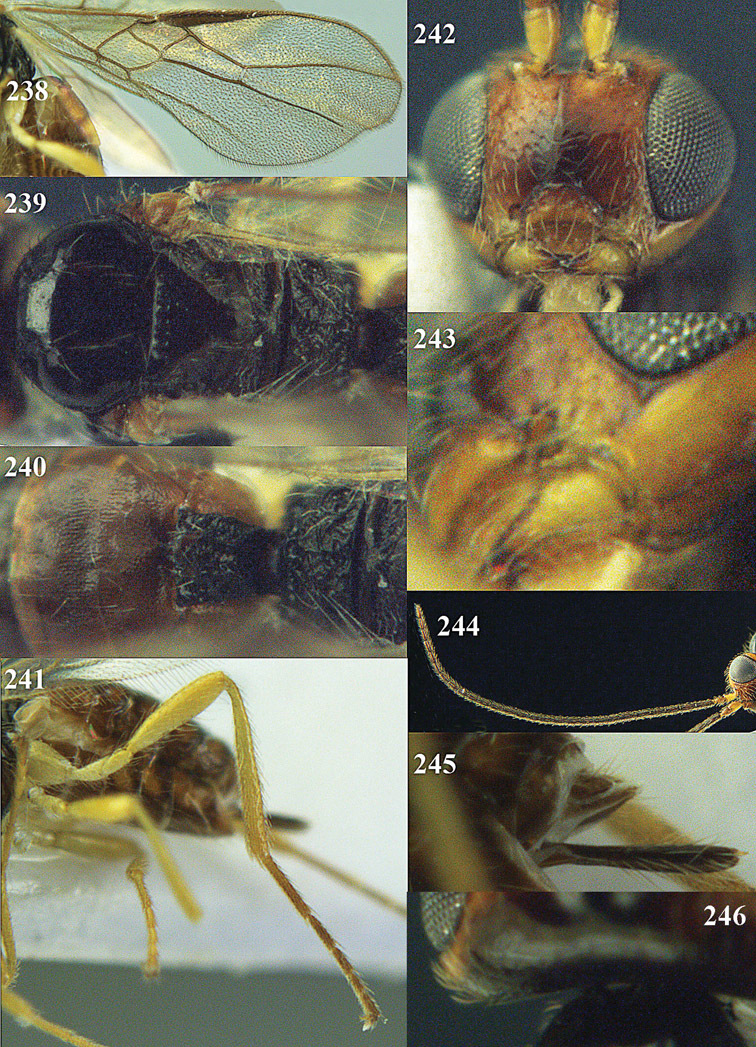
*Phaedrotoma depressiclypealis* sp. n., female, holotype. **238** Wings **239** mesosoma dorsal **240** propodeum and 1^st^-3^rd^ metasomal tergites dorsal **241** hind leg **242** head anterior **243** mandible **244** antenna **245** ovipositor sheath **246** pronope dorsal.

*Wings*. Fore wing (Fig. 238): pterostigma narrow elliptical, narrowed apically; 1-R1 reaching wing apex and 1.2 times as long as pterostigma; r:3-SR:SR1 = 4:30:75; r slender; 2-SR:3-SR:r-m = 17:30:8; 1-M nearly straight; SR1 slightly curved; m-cu narrowly postfurcal; cu-a postfurcal and 1-CU1 widened; first subdiscal cell closed, CU1b short. Hind wing (Fig. 238): M+CU:1-M:1r-m = 15:13:8; cu-a straight; m-cu absent.

*Legs*. Length of femur, tibia and basitarsus of hind leg 4.1, 10.6 and 7.1 times as long as wide, respectively; hind femur with long setae and of tibia medium-sized (Fig. 241).

*Metasoma*. Length of first tergite 1.2 times its apical width, its surface weakly and gradually convex and with widely spaced rugae and with granulate interspaces, dorsal carinae irregular and up to its posterior fifth (Fig. 240); second and third tergites superficially granulate, division of tergites slightly elevated (Fig. 240); length of ventrally visible setose part of ovipositor sheath 0.10 times fore wing and 0.35 times hind tibia (Figs 237, 245); apex of hypopygium rather protruding (Fig. 245).

*Colour*. Blackish-brown or dark brown; palpi, coxae and trochanters ivory; legs pale yellowish (but hind tibia apically and basally, and hind tarsus more or less infuscate); antenna (but scapus largely yellowish), ovipositor sheath, pterostigma and veins dark brown; wing membrane subhyaline; tegulae, face largely (except medially), frons (except medially), temple largely and metasoma largely laterally yellowish-brown; mesopleuron ventrally and antero-dorsally and remainder of metasoma (except first tergite) more or less brown.

*Molecular data*. None.

#### Distribution.

*China (Hunan).

#### Biology.

Unknown.

#### Etymology.

Name derived from “depressus” (Latin for “pressed down”) and cly-peus, because of the ventrally depressed clypeus.

#### Notes.

The new species runs in the key by Chen and Weng (2005) to *Phaedrotoma larga* (Weng & Chen, 2005) comb. n. *Phaedrotoma depressiclypealis* differs by having vein 3-SR of fore wing 1.8 times vein 2-SR (1.6 times in *Phaedrotoma larga*), the mandible hardly widened basally (distinctly widened) and the length of the first tergite 1.2 times its apical width (0.9 times).

### Phaedrotoma flavisoma

Li & van Achterberg sp. n.

urn:lsid:zoobank.org:act:BF4EDC56-57E6-428A-8DE3-D165D014D21F

http://species-id.net/wiki/Phaedrotoma_flavisoma

Figs 247 –256

#### Type material.

Holotype, ♂ (ZUH), “S. China: Hunan, Taoyuan, Wuyunjie Mts., Houxi, 6–8.VI.2010, 500 m, Xi-Ying Li, No. 38”.

#### Diagnosis.

**Figure 247. F60:**
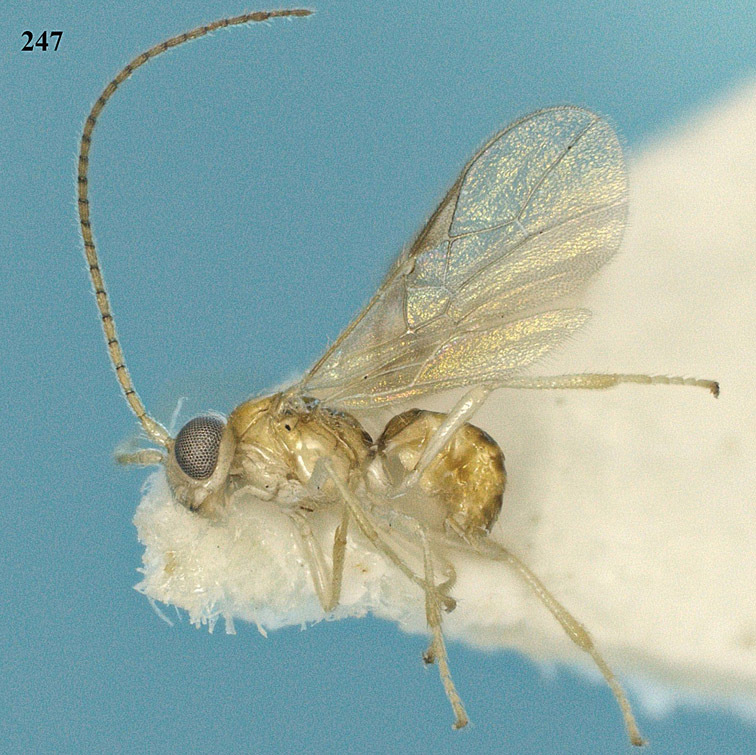
*Phaedrotoma flavisoma* sp. n., male, holotype. Habitus lateral.

Pronotal side smooth (Fig. 247); head moderately transverse and yellow; clypeus comparatively narrow, truncate ventrally, semi-circular and about 3.5 times as wide as high (Fig. 252); vein SR1 of fore wing 1.9 times as long as vein 3-SR; mesosoma completely pale yellow; propodeum, second and third metasomal tergite smooth (Figs 249, 250); first tergite superficially granulate (Fig. 250); anterior groove of metapleuron smooth; apical half of first metasomal tergite subparallel-sided (Fig. 250); occipital carina far removed from hypostomal carina; malar suture absent; hind tarsus pale yellowish as basal half of hind tibia; length of malar space 0.3 times basal width of mandible; third antennal segment about 3.5 (♂) times as long as wide; antenna 1.6 times longer than fore wing; face ivory and mesosoma pale yellowish; first tergite twice as long as wide apically (Fig. 240).

#### Description.

Holotype, ♂, length of body 1.7 mm, of fore wing 1.9 mm.

*Head*. Antenna with 27 segments and 1.4 times as long as fore wing; length of third segment 1.1 times fourth segment, length of third, fourth and penultimate segments 3.3, 3.0 and 2.8 times their width, respectively (Fig. 255); length of maxillary palp about equal to height of head; labial palp segments normal; occipital carina far removed from hypostomal carina and dorsally absent; hypostomal carina narrow; length of eye in dorsal view 4.0 times temple; frons smooth, glabrous, flattened (Fig. 254); face smooth, glabrous, medially weakly elevated (Fig. 252); width of clypeus 3.3 times its maximum height and 0.6 times width of face; clypeus comparatively narrow and truncate medially, normal ventrally and semi-circular, its ventral margin thin and straight (Figs 252, 253); hypoclypeal depression large (Fig. 252); malar suture absent; length of malar space 0.3 times basal width of mandible gradually widened basally, with a ventral carina (Fig. 253).

*Mesosoma*. Length of mesosoma 1.4 times its height; dorsal pronope absent; pronotal side smooth; epicnemial area smooth dorsally (Fig. 247); precoxal sulcus only medially impressed, smooth; rest of mesopleuron smooth, glabrous; pleural sulcus smooth; anterior groove of metapleuron smooth; notauli absent on disc, only anteriorly indicated by shallow depressions (Fig. 249); mesoscutum glabrous except for a few setae along imaginary notaulic courses; medio-posterior depression of mesoscutum absent; scutellar sulcus finely crenulate; scutellum smooth and flattened; surface of propodeum smooth (Fig. 249).

**Figures 248–256. F61:**
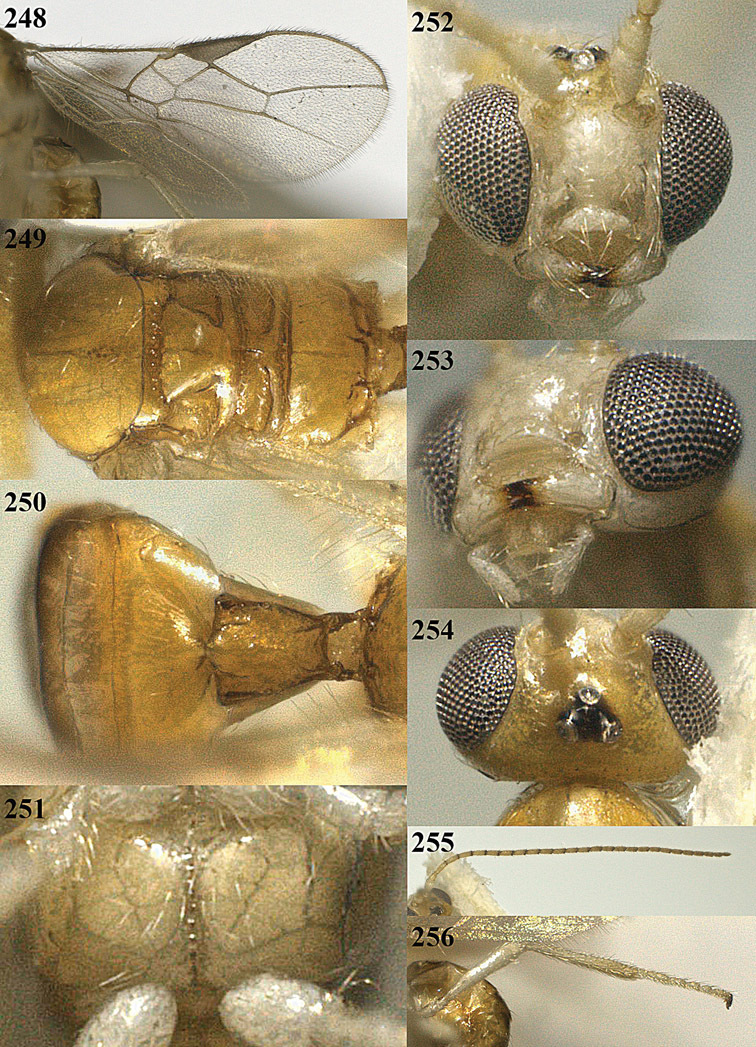
*Phaedrotoma flavisoma* sp. n., male, holotype. **248** Wings **249** mesosoma dorsal **250** propodeum and 1^st^-3^rd^ metasomal tergites dorsal **251** mesosternum ventral **252** head anterior **253** mandible **254** head dorsal **255** antenna **256** hind leg.

*Wings*. Fore wing (Fig. 248): pterostigma triangular; 1-R1 reaching wing apex and 1.6 times as long as pterostigma; r:3-SR:SR1 = 2:33:60; 2-SR:3-SR:r-m = 20:33:9; r rather short; 1-M slightly curved; SR1 nearly straight; m-cu postfurcal; cu-a slightly postfurcal and 1-CU1 widened; first subdiscal cell closed, CU1b medium-sized. Hind wing (Fig. 248): M+CU:1-M:1r-m = 22:18:9; cu-a straight; m-cu absent.

*Legs*. Length of femur, tibia and basitarsus of hind leg 7.0, 10.0 and 6.0 times as long as wide, respectively; hind femur with long setae and of tibia medium-sized (Fig. 256).

*Metasoma*. Length of first tergite 1.5 times its apical width, its surface moderately convex and largely finely rugose, dorsal carinae united in its anterior 0.5 and absent behind it (Fig. 250); second and following tergites smooth.

*Colour*. Yellow; antenna, pronotal side, mesopleuron, tegulae, pale yellowish; face, mandible, clypeus, palpi, malar space and legs ivory; pterostigma brown, veins yellowish-brown; wing membrane subhyaline.

*Molecular data*.None.

#### Distribution.

*China (Hunan).

#### Biology.

Unknown.

#### Etymology.

Name derived from “flavus” (Latin for “yellow”) and “soma” (Greek for body), because of the yellow body.

#### Notes.

The new species runs in the key by Chen and Weng (2005) to *Phaedrotoma rugulosa* (Chen & Weng, 2005) comb. n. and differs by having the clypeus 3.5 times wider than high (2.4 times in *Phaedrotoma rugulosa*), the malar space 0.3 times basal width of mandible (about 0.8 times), vein m-cu of fore wing far postfurcal (subinterstitial) and the triangular pterostigma (narrow elliptical).

### Phaedrotoma nigrisoma

Li & van Achterberg sp. n.

urn:lsid:zoobank.org:act:18ABBFEF-F413-4117-959B-F63FD6F45066

http://species-id.net/wiki/Phaedrotoma_nigrisoma

Figs 257–263

#### Type material.

Holotype, ♂ (ZUH), “S. China: Hunan, nr Changsha, Ningxiang, 5.X.1986, Xiao-ming Ou”.

#### Diagnosis.

Antenna of male with about 21 segments and about as long as fore wing; clypeus comparatively narrow, width of clypeus 4.0 times its maximum height (Fig. 260); body black; legs dark brown; vein SR1 of fore wing 3 times as long as vein 3-SR; vein 3-SR 1.7 times as long as vein 2-SR (Fig. 258); length of hind femur about 5.6 times its width.

#### Description.

Holotype, ♂, length of body 1.4 mm, of fore wing 1.6 mm.

**Figures 257–263. F62:**
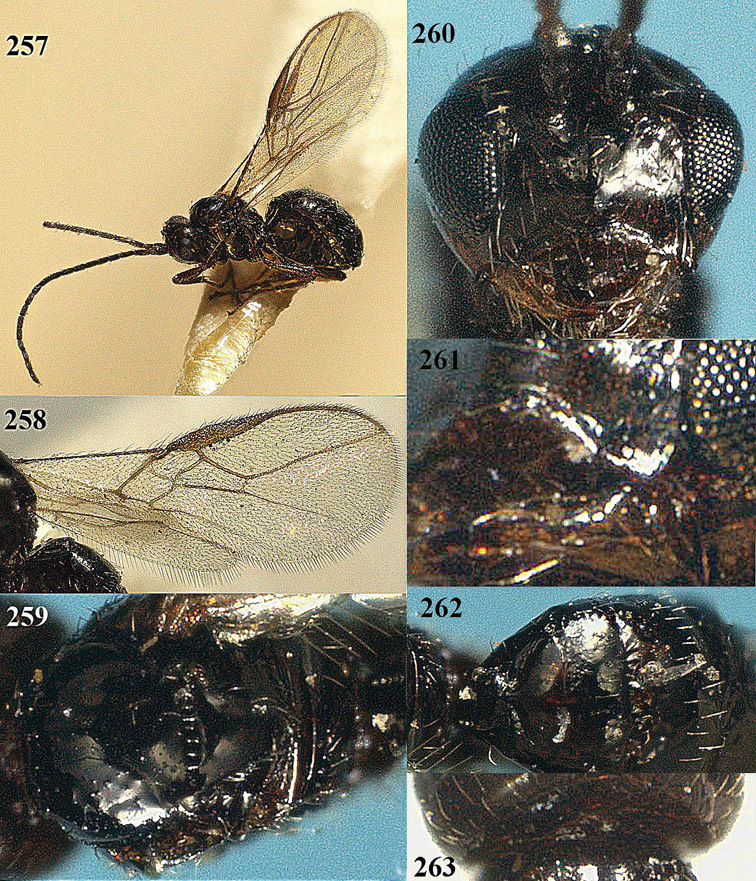
*Phaedrotoma nigrisoma* sp. n., male, holotype. **257** Habitus lateral **258** wings **259** mesosoma dorsal **260** head anterior **261** mandible **262** propodeum and 1^st^-3^rd^ metasomal tergites dorsal **263** temple andpronope dorsal.

*Head*. Antenna with 21 segments and about as long as fore wing; length of third segment 1.2 times fourth segment, length of third, fourth and penultimate segments 3.2, 2.8, and 1.9 times their width, respectively (Fig. 257); length of maxillary palp 0.9 times height of head; labial palp segments normal; occipital carina removed from hypostomal carina and dorsally absent; length of eye in dorsal view 1.6 times temple; frons glabrous, slightly convex laterally; face largely smooth but laterally superficially punctate, medially distinctly elevated (Fig. 260); width of clypeus 4.0 times its maximum height and 0.8 times width of face; clypeus distinctly convex, punctate and its ventral margin curved (Fig. 260); hypoclypeal depression large (Fig. 260); malar suture present; malar space 0.3 times basal width of mandible; mandible gradually widened basally, with narrow and ventral carina (Fig. 261).

*Mesosoma*. Length of mesosoma 1.2 times its height; dorsal pronope round (Fig. 263); pronotal side largely smooth but partly superficially granulate (Fig. 257); epicnemial area smooth dorsally; precoxal sulcus only medially impressed, finely sculptured; remainder of mesopleuron and pleural sulcus smooth; anterior groove of metapleuron smooth; notauli absent on disc, only anteriorly indicated by shallow depressions (Fig. 259); mesoscutum glabrous; medio-posterior depression of mesoscutum absent; scutellar sulcus moderately crenulate; scutellum smooth or nearly so; surface of propodeum largely smooth, without carinae (Fig. 259).

*Wings*. Fore wing (Fig. 258): pterostigma slender elliptical, narrowed apically; 1-R1 not reaching wing apex and 0.9 times as long as pterostigma; r:3-SR:SR1 = 4:16:52; r widened; 2-SR:3-SR:r-m = 13:16:5; 1-M straight; SR1 nearly straight; m-cu distinctly postfurcal; cu-a postfurcal and 1-CU1 widened; first subdiscal cell opened, CU1b absent; Hind wing (Fig. 258): M+CU:1-M:1r-m = 11:20:9; cu-a straight; m-cu absent.

*Legs*. Length of femur, tibia and basitarsus of hind leg 5.6, 7.4 and 3.8 times as long as wide, respectively; hind femur and tibia with medium-sized setae (Fig. 257).

*Metasoma*. Length of first tergite 0.7 times its apical width, its surface distinctly convex and largely smooth (Fig. 263); second and third tergites smooth.

*Colour*. Black; antenna, palpi and legs dark brown; pterostigma and veins dark brown; wing membrane subhyaline.

*Molecular data*. None.

#### Distribution.

*China (Hunan).

#### Biology.

Unknown.

#### Etymology.

Name derived from “niger” (Latin for “black”) and “soma”(Latin for “body”), because of the black body.

#### Notes.

The new species runs with difficulty in the key by Chen and Weng (2005) to *Rhogadopsis sulcifer* (Fischer, 1975) comb. n., but *Phaedrotoma nigrisoma* has vein m-cu angled with veins 2-M and 2-CU1 (linear or gradually merging in *Rhogadopsis sulcifer*), malar space 0.3 times as long as basal width of mandible (equal) and propodeum smooth (areolate).

### Phaedrotoma protuberator

Li & van Achterberg sp. n.

urn:lsid:zoobank.org:act:E40DC0FB-47DC-46F1-90CB-AEDC7AC986B5

http://species-id.net/wiki/Phaedrotoma_protuberator

Figs 264 –274 394 –412

#### Type material.

Holotype, ♀ (ZUH), “S. China: Hunan, nr Zhangjiajie, Badagong Mts, Bamaoxi, 2–3.vi.2009, 540 m, Xi-Ying Li, RMNH’09”. Paratypes (RMNH, ZUH): 4 ♀ + 4 ♂, with same label data as holotype; 4 ♀ + 4 ♂, id., but Longtanping, 4-5.vi.2009, 550 m; 1 ♀, id., but Tian Ping Mt, 9–13.vii.2009, 550 m; 3 ♀ + 1 ♂, “S. China: Hunan, nr Suining, Huangsang N. R., Shaoyang, 12–13.vi.2009, 1000 m, Xi-Ying Li, RMNH’09”; 1 ♀ + 2 ♂, “S. China: Hunan, nr Chengbu, Nan Mt., Shaoyang, 1500 m, 10–11.vi.2009, Xi-Ying Li, RMNH’09”; 1 ♀, “N. China: Shandong, Anqiu, Suotou Mt., 31.vii.2009, c. 120 m, Li Xi-Ying, RMNH’09”; 1 ♀ (RMNH), “China: Hunan, Changde, Taoyuan, Mao-zong-ling Xiang, Shuangxi-kou Xiang, roadside, at light, CN 1033, 12.viii.2010, P.-P. Chen, RMNH’11”.

#### Diagnosis.

Hypopygium of ♀ rather acute apically and 0.3 times as long as metasoma; labrum normal, without large space below clypeus (Fig. 270); clypeus normal ventrally and semicircular; clypeus 0.6 times as wide as face or less, protruding forwards; face less transverse (Fig. 270); occipital carina removed from hypostomal carina; malar suture entirely absent; medio-posterior depression of mesoscutum absent; scutellar sulcus at most moderately crenulate; scutellum flat or nearly so; posterior groove of propleuron smooth or absent; pronope round and large (Fig. 274); lateral carina of mesoscutum present; mesosoma largely black; precoxal sulcus finely crenulate or smooth; propodeum without oblique crests; vein m-cu of fore wing slightly postfurcal; vein CU1b shorter than vein 3-CU1 or subequal (Fig. 265); hind femur about 5 times as long as wide; second and third metasomal tergites more or less superficially granulate (Fig. 267); head and mesosternum largely yellowish-brown; mandible gradually widened basally and narrow apically; hind tarsus infuscate.

#### Description.

Holotype, ♀, length of body 1.6 mm, of fore wing 1.9 mm.

*Head*. Antenna with 30 segments and 1.7 times as long as fore wing; length of third segment 1.2 times fourth segment, length of third, fourth and penultimate segments 4.3, 3.7 and 2.5 times their width, respectively (Fig. 269); length of maxillary palp 0.9 times height of head; labial palp segments normal; occipital carina close to hypostomal carina and dorsally absent; hypostomal carina narrow; length of eye in dorsal view 1.5 times temple; frons glabrous, smooth, flattened and with pit medially, slightly convex laterally; face smooth, medially weakly elevated; width of clypeus 2.2 times its maximum height and 0.45 times width of face; clypeus convex, protruding forwards, largely punctate and its ventral margin thin and straight (Fig. 270); hypoclypeal depression medium-sized (Fig. 270); malar suture entirely absent; mandible gradually widened basally, with short and non-protruding ventral carina (Fig. 271).

**Figure 264. F63:**
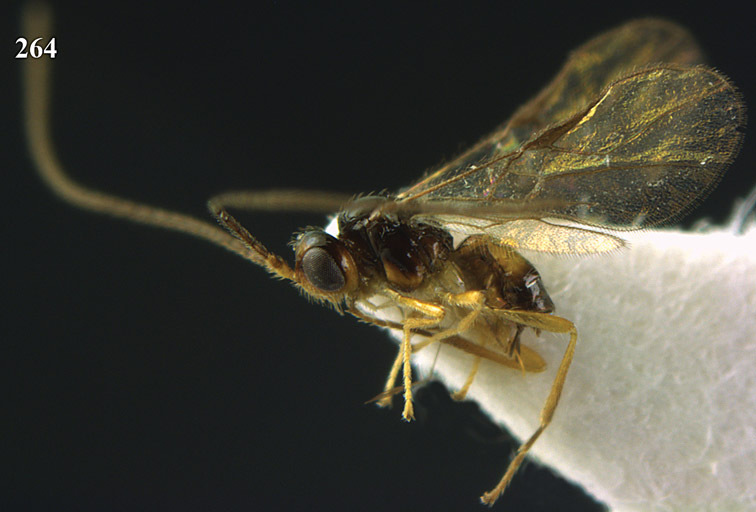
*Phaedrotoma protuberator* sp. n., female, holotype. Habitus lateral.

*Mesosoma*. Length of mesosoma 1.2 times its height; dorsal pronope distinct, large, round (Fig. 274); pronotal side mainly smooth, but slightly granulate posteriorly and posterior groove absent (Fig. 264); epicnemial area smooth dorsally; precoxal sulcus only medially impressed, nearly smooth as rest of mesopleuron (Fig. 264); pleural sulcus smooth; anterior groove of metapleuron crenulate; notauli absent on disc, only anteriorly indicated by shallow depressions; mesoscutum glabrous except for a few setae along imaginary notaulic courses (Fig. 266); medio-posterior depression of mesoscutum absent; lateral carina of mesoscutum present; scutellar sulcus moderately crenulate; scutellum smooth and flattened; surface of propodeum largely smooth anteriorly, posteriorly obliquely rugulose, without carinae (Fig. 266).

**Figures 265–274. F64:**
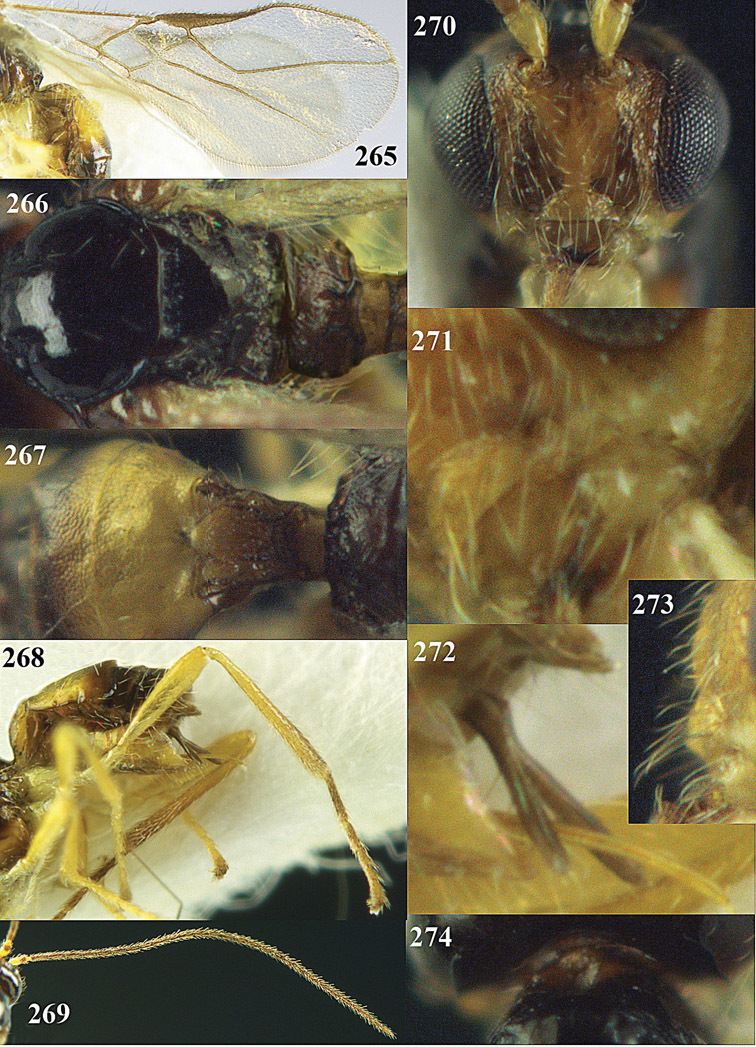
*Phaedrotoma protuberator* sp. n., female, holotype. **265** Wings **266** mesosoma dorsal **267** propodeum and 1^st^-3^rd^ metasomal tergites dorsal **268** hind leg **269** antenna **270** head anterior **271 **mandible **272** ovipositor sheath **273** clypeus lateral **274** pronope dorsal.

*Wings*. Fore wing (Fig. 265): pterostigma wide elliptical, narrowed apically; 1-R1 reaching wing apex and 1.2 times as long as pterostigma (Fig. 265); r:3-SR:SR1 = 2:21:61; 2-SR:3-SR:r-m = 13:21:6; 1-M straight; SR1 slightly curved; m-cu slightly postfurcal; cu-a postfurcal and 1-CU1 widened; first subdiscal cell closed, CU1b medium-sized. Hind wing (Fig. 265): M+CU:1-M:1r-m = 12:15:7; cu-a straight; m-cu absent.

*Legs*. Length of femur, tibia and basitarsus of hind leg 5.3, 10.3 and 6.0 times as long as wide, respectively; hind femur with long setae and of tibia medium-sized (Fig. 268).

*Metasoma*. Length of first tergite 1.2 times its apical width, its surface distinctly convex but flattened medially and largely finely rugose or rugulose, dorsal carinae developed on its anterior 0.6, curved (Fig. 267); second and following tergites largely smooth, but with some superficial granulation, division of tergites slightly elevated (Fig. 267); length of ventrally visible setose part of ovipositor sheath 0.07 times fore wing and 0.2 times hind tibia (Figs 264, 272); apex of hypopygium rather acute (Fig. 272).

*Colour*. Black; palpi, mandible, malar space, second and base of third tergite and legs pale yellowish, but hind tibia apically and hind tarsus more or less infuscate; face, temple medially and ventrally, mesopleuron ventrally, mesosternum, first tergite and metasoma ventrally, yellowish-brown; frons and vertex laterally brown; antenna (but scapus yellowish), tegulae, ovipositor sheath and apical dorsal half of metasoma dark brown; pterostigma and veins dark brown; wing membrane subhyaline.

*Molecular data*. COI, 16S, 28S (CVA4254).

*Variation*. Length of body 1.6-2.0 mm, of fore wing 1.9-2.4 mm; antenna of female with 25 (1), 27 (3), 28 (3), 29 (1), 30 (3) or 31 (1) segments, of male with 24 (1), 28 (3), 29 (1), 30 (1), 31 (3) or 32 (1) segments; second and third metasomal tergites nearly smooth and shiny to distinctly granulate and matt, sometimes only third tergite granulate; mesosoma laterally, metanotum and propodeum may be largely brown or chestnut brown; clypeus often largely smooth, protuberant, but ventrally with acute margin or obtuse, protruding or depressed; head (especially of males) may be largely brownish-yellow; shallow medial depression of frons present or absent; second and third tergites are dark brown in melanistic specimens. Some female paratypes (Figs 394, 398) have the hind tarsus hardly or not infuscate, the propodeum completely or largely coarsely sculptured and the precoxal sulcus distinctly crenulate.

#### Distribution.

*China (Hunan).

#### Biology.

Unknown.

#### Etymology.

Name derived from “protuberatus” (Latin for “bulging out”), because the clypeus more or less protrudes forwards.

#### Notes.

Belongs morphologically to the genus *Phaedrotoma* Foerster, but according to the DNA analysis belongs to the genus *Opius* Wesmael. The DNA analysis should be re-done to be sure this is not a result of contamination.

The new species does not run well in the key by Chen and Weng (2005); if the robust mesosoma is considered the most important character then it ends up at *Phaedrotoma amputata* (Weng & Chen, 2005) comb. n. *Phaedrotoma protuberator* differs by having the anterior groove of the metapleuron crenulate (smooth), vein 3-SR of fore wing 1.6 times vein 2-SR (twice in *Phaedrotoma amputata*), the precoxal sulcus comparatively long and nearly parallel to the pleural sulcus (short and angled with pleural sulcus) and vein m-cu of the fore wing is distinctly angled with vein 2-M (gradually merging).

### Phaedrotoma rugulifera

Li & van Achterberg sp. n.

urn:lsid:zoobank.org:act:779442B1-AE2C-4287-B234-14AC69E5A313

http://species-id.net/wiki/Phaedrotoma_rugulifera

Figs 275 –285

#### Type material.

Holotype, ♀ (ZUH), “S. China: Hunan, nr Zhangjiajie, Badagong Mts, Bamaoxi, 2–3.VI.2009, 540 m, Xi-Ying Li, RMNH’09”. Paratypes (RMNH): 2 ♀ + 1 ♂, same label data; 1 ♀ id., but Tian Ping Mt., 9–13.VII.2009, c 1400 m; 1 ♂, id., but Longtanping, 4–5.VI.2009, 550 m; 1 ♀ + 1 ♂, “S. China: Hunan, nr Chengbu, Nan Mt., Shaoyang, 1500 m, 10–11.VI.2009, Xi-Ying Li, RMNH’09”.

#### Diagnosis.

Malar suture largely absent (Fig. 282); clypeus medium-sized (Fig. 281); second submarginal cell of fore wing medium-sized (Fig. 276); setose part of ovipositor sheath 0.6-0.8 times as long as hind tibia; pronotum with large round pronope (Fig. 285); epicnemial area crenulate; propodeum usually largely densely rugose (Fig. 277); second and third metasomal tergites superficially granulate (Fig. 278).

#### Description.

Holotype, ♀, length of body 2.6 mm, of fore wing 2.8 mm.

*Head*. Antenna with 34 segments and 1.5 times as long as fore wing; length of third segment 1.3 times fourth segment, length of third, fourth and penultimate segments 4.7, 3.7 and 2.7 times their width, respectively (Fig. 280); length of maxillary palp 1.3 times height of head; labial palp segments normal, elongate; occipital carina rather close to hypostomal carina and dorsally absent (Fig. 282); hypostomal carina medium-sized; length of eye in dorsal view 3.5 times temple; frons glabrous, smooth and strongly shiny and flattened medially, slightly convex laterally; face largely smooth between sparse punctures, but slightly micro-sculptured medio-ventrally and dorso-laterally, medially slightly elevated (Fig. 281); width of clypeus 2.9 times its maximum height and 0.5 times width of face; clypeus moderately convex and punctate, ventrally not protruding forwards and its ventral margin rather sharp and straight (Fig. 281); hypoclypeal depression comparatively large (Fig. 281); malar suture largely absent, slightly impressed near eye (Fig. 282); malar space comparatively short (Fig. 282), half as long as basal width of mandible; mandible gradually widened basally, with narrow and non-protruding ventral carina (Fig. 282).

**Figure 275. F65:**
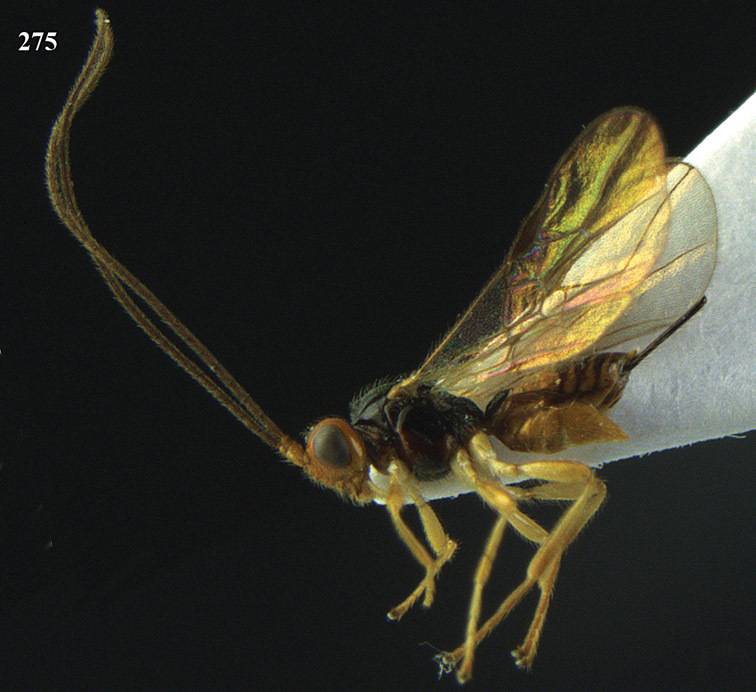
*Phaedrotoma rugulifera* sp. n., female, holotype. Habitus lateral.

*Mesosoma*. Length of mesosoma 1.3 times its height; dorsal pronope large and round (Fig. 285); pronotal side largely smooth and posterior groove largely absent (Fig. 275); epicnemial area crenulate; precoxal sulcus subanteriorly and medially impressed, narrow and comparatively deep, distinctly crenulate (Fig. 275); pleural sulcus smooth; anterior groove of metapleuron crenulate ventrally; notauli absent on disc, only anteriorly indicated by deep and smooth depressions (Fig. 277); meso-scutum glabrous except for a few setae along imaginary notaulic courses and about as long as wide (Fig. 277); medio-posterior depression of mesoscutum absent; lateral carina of mesoscutum largely absent anteriorly, only as a row of punctures; scutellar sulcus distinctly crenulate; scutellum smooth or nearly so and flattened; surface of propodeum coarsely and densely rugose, without distinct transverse or median carinae (Fig. 277).

*Wings*. Fore wing (Fig. 276): pterostigma rather wide elliptical, narrowed apically; 1-R1 reaching wing apex and 1.3 times as long as pterostigma; r:3-SR:SR1 = 3:27:73; r slender; 2-SR:3-SR:r-m = 20:27:7; 1-M straight; SR1 slightly curved; m-cu distinctly postfurcal; cu-a just postfurcal and 1-CU1 widened; first subdiscal cell closed, CU1b short. Hind wing (Fig. 276): M+CU:1-M:1r-m = 21:15:9; cu-a straight; m-cu absent.

*Legs*. Length of femur, tibia and basitarsus of hind leg 3.7, 9.8 and 5.7 times as long as wide, respectively; hind femur with long setae and of tibia medium-sized (Fig. 279).

*Metasoma*. Length of first tergite 0.9 times its apical width, its surface weakly and gradually convex and with widely spaced rugae and with granulate interspaces, dorsal carinae irregular and up to its posterior fifth (Fig. 278); second and third tergites superficially granulate, division of tergites slightly elevated; length of ventrally visible setose part of ovipositor sheath 0.27 times fore wing, 2.5 times first tergite and 0.8 times hind tibia (Figs 275, 284); apex of hypopygium acute (Fig. 283).

*Colour*. Black; palpi, malar space, mandible, coxae and trochanters ivory; legs pale yellowish (but hind tibia apically and hind tarsus slightly infuscate); antenna (but scapus and pedicellus largely yellowish), pronotal side ventrally propleuron largely, mesopleuron ventrally, apical half of metasoma dorsally, pterostigma and veins more or less dark brown; wing membrane subhyaline; tegula brown; head (except black stemmaticum and dark brown middle of vertex), metasoma ventrally and second and third tergites brownish-yellow.

*Molecular data*. COI, 16S, 28S (CVA4237, CVA4248).

*Variation*. Length of body 2.2-3.1 mm, of fore wing 2.4-3.2 mm; antenna of female with 32 (1), 33 (1), 34 (1) or 36 (1) segments, of male with 30 (1) or 34 (2) segments; mesosoma completely black or dark brown and laterally partly brownish-yellow; head largely dark brown to nearly completely brownish-yellow.

**Figure F66:**
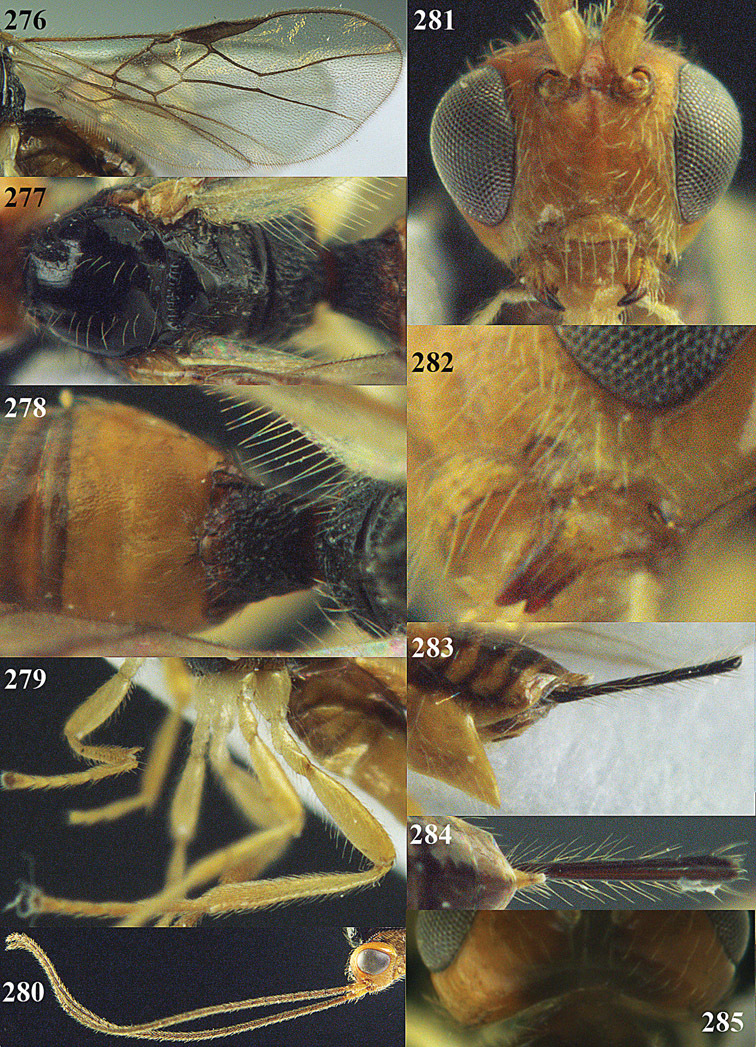
**Figures 276–285.** *Phaedrotoma rugulifera* sp. n., female, holotype. **276** Wings **277** mesosoma dorsal **278** propodeum and 1^st^-3^rd^ metasomal tergites dorsal **279** hind leg **280** antennae **281** head anterior **282 **mandible **283** ovipositor sheath lateral **284** id. dorsal **285** pronope dorsal.

#### Distribution.

*China (Hunan).

#### Biology.

Unknown.

#### Etymology.

Name derived from “rugulum” (Latin for “small ruga or wrinkle”) and “ferum” (suffix in Latin meaning “carrying” or “having”), because of the densely rugulose propodeum.

#### Notes. 

The new species does not run well in the key by Chen and Weng (2005), but if the length of the mesosoma is considered to be variable, it runs to *Phaedrotoma abortiva* (Weng & Chen, 2005) comb. n. *Phaedrotoma rugulifera* differs by having the mesosoma 1.3 times as long as high (1.5-1.6 times in *Phaedrotoma abortiva*), the malar suture largely absent, only shallowly impressed near the eye (deep and almost complete) and the length of the maxillary palp 1.3 times height of head (equal).

### Phaedrotoma semiplanata

Li & van Achterberg sp. n.

urn:lsid:zoobank.org:act:6B389F90-3761-432E-AAB1-6168A2493C63

http://species-id.net/wiki/Phaedrotoma_semiplanata

Figs 286 –294

#### Type material.

Holotype, ♀ (ZJUH), “S. China: Hunan, nr Zhangjiajie, Badagong Mts, Tian Ping Mt., 9-13.vii.2009, 550 m, Xi-Ying Li, RMNH’10”, “CVA4256, sp. 22”.

#### Diagnosis.

**Figure 286. F67:**
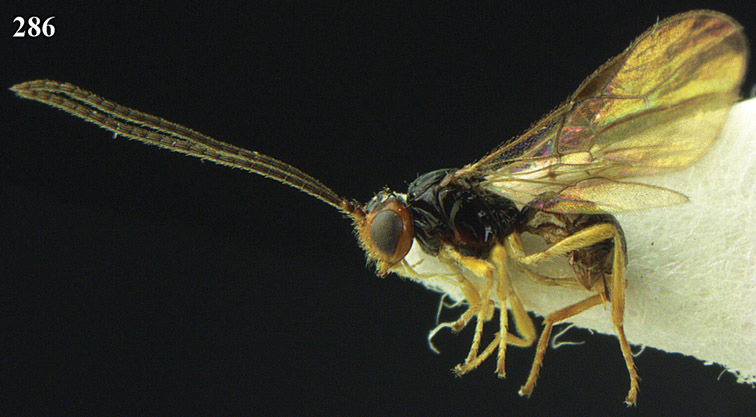
*Phaedrotoma semiplanata* sp. n., female, holotype. Habitus lateral.

**Figures 287–294. F68:**
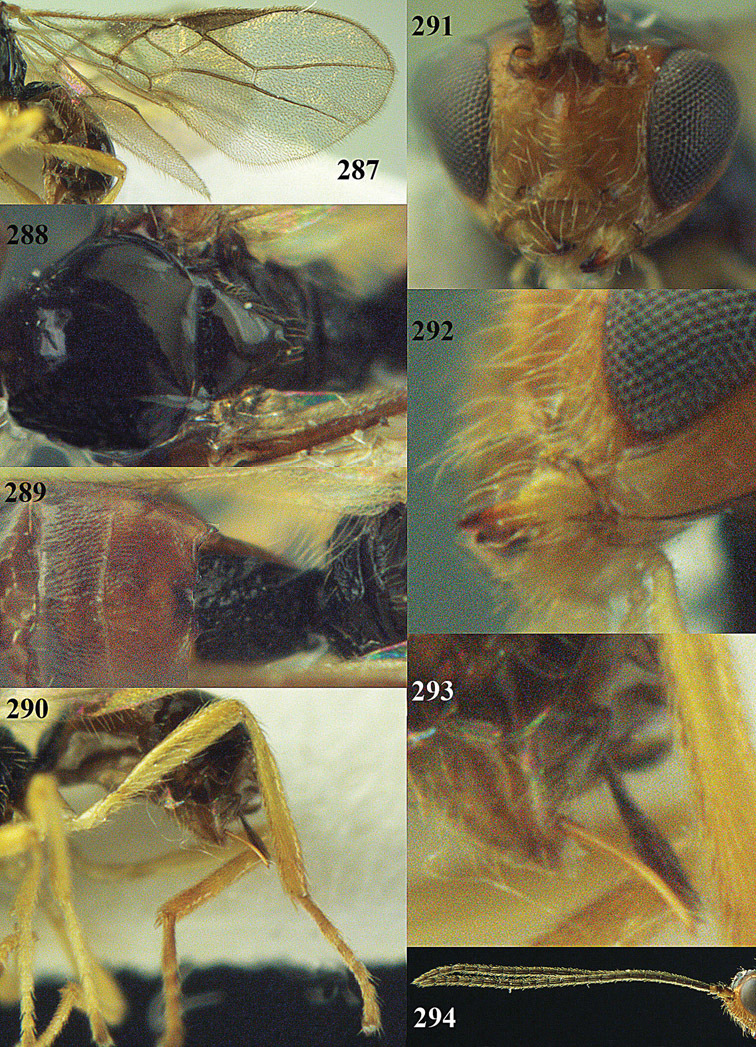
*Phaedrotoma semiplanata* sp. n., female, holotype. **287** Wings **288** mesosoma dorsal **289** propodeum and 1^st^-3^rd^ metasomal tergites dorsal **290** hind leg **291** head anterior **292** mandible **293** ovipositor sheath **294** antennae.

Vein 1r-m of hind wing about 0.4 times as long as vein 1-M (Fig. 287); posterior groove of pronotal side and anterior groove of metapleuron smooth (Fig. 286).

#### Description.

Holotype, ♀, length of body1.8 mm, of fore wing 2.1 mm.

*Head*. Antenna with 23 segments and 1.2 times as long as fore wing; third segment 1.4 times as long as fourth segment, length of third, fourth and penultimate segments 4.5, 3.2 and 2.2 times their width, respectively (Fig. 294); length of maxillary palp 1.1 times height of head; labial palp segments normal; occipital carina widely removed from hypostomal carina and dorsally absent (Fig. 292); hypostomal carina medium-sized; length of eye in dorsal view 2.3 times temple; frons slightly depressed behind antennal sockets and in front of anterior ocellus and glabrous, smooth; face smooth, medially elevated; width of clypeus 2.7 times its maximum height and 0.45 times width of face; clypeus rather convex but slightly protruding forwards and largely smooth (except for some punctures) and its ventral margin slightly concave and sharp (Fig. 291); hypoclypeal depression rather large (Fig. 291); malar suture deep; mandible triangular and with narrow ventral carina (Fig. 292).

*Mesosoma*. Length of mesosoma 1.2 times its height; dorsal pronope large, deep and round (Fig. 293); pronotal side smooth and posterior groove largely absent (Fig. 286); epicnemial area smooth dorsally; precoxal sulcus medially subvertical, deep and slightly finely crenulate (Fig. 286); rest of mesopleuron smooth; pleural sulcus smooth; mesosternal sulcus deep and moderately crenulate; anterior groove of metapleuron largely smooth; notauli absent on disc, only anteriorly with pair of short smooth impressions (Fig. 288); mesoscutum glabrous and strongly shiny; medio-posterior depression of mesoscutum absent; scutellar sulcus moderately crenulate; scutellum convex medially, smooth; propodeum with short medio-longitudinal carina and rugose anteriorly, surface posteriorly mainly superficially rugulose (Fig. 289).

*Wings*. Fore wing (Fig. 287): pterostigma elongate triangular; 1-R1 ending at wing apex and1.4 times as long as pterostigma; r:3-SR:SR1 = 2:25:62; 2-SR:3-SR:r-m = 17:25:7; r somewhat widened; 1-M slightly curved and SR1 straight; m-cu distinctly postfurcal; cu-a slightly postfurcal and 1-CU1 widened; first subdiscal cell closed, CU1b medium-sized; apical quarter of M+CU1 sclerotized. Hind wing (Fig. 287): M+CU:1-M:1r-m = 5:5:2; cu-a straight; m-cu present as faint unpigmented trace.

*Legs*. Length of femur, tibia and basitarsus of hind leg 4.2, 9.4 and 5.0 times as long as wide, respectively; hind femur with long and tibia with medium-sized setae (Fig. 290).

*Metasoma*. Length of first tergite 1.3 times its apical width, its surface evenly moderately convex and coarsely vermiculate, its dorsal carinae developed in basal 0.4 of tergite, straight (Fig. 289); second suture slightly indicated; second and third tergites superficially longitudinally rugulose (Fig. 289); length of setose part of ovipositor sheath 0.07 times fore wing and 0.2 times length of hind tibia (Figs 286, 293).

*Colour*. Black; antenna (but scapus yellowish laterally and ventrally), stemmaticum, head dorsally (except near eyes), first tergite, most of fourth and following tergites, ovipositor sheath and pterostigma dark brown; face, malar space, palpi, mandible and legs (but hind tarsus and telotarsi darkened) pale yellow; tegulae and veins brown; wing membrane subhyaline.

*Molecular data*.COI, 16S, 28S (CVA4256).

#### Distribution.

*China (Hunan).

#### Biology.

Unknown.

#### Etymology.

Name derived from “semi” (Latin for “half”) and “planus” (Latin for “flat”), because of the rather flat clypeus.

**Notes.** The new species runs in the key by Chen and Weng (2005) to *Phaedrotoma dimidia* (Chen& Weng, 2005) comb. n. *Phaedrotoma semiplanata* differs by having the setose part of the ovipositor sheath 0.7 times as long as first tergite ((about 3 times in *Phaedrotoma dimidia*), the notauli largely absent on the mesoscutal disc and smooth (anterior half present on disc and crenulate) and the propodeum without a transverse carina or posterior areola (present).

### Phaedrotoma striatinota

Li & van Achterberg sp. n.

urn:lsid:zoobank.org:act:C1156DDB-5E95-4B2D-8B9B-B74AE5B47E10

http://species-id.net/wiki/Phaedrotoma_striatinota

Figs 295 –303

#### Type material.

Holotype, ♀ (ZUH), “S. China: Hunan, Shaoyang, Chengbu, Nan Mts., 1.VI.1985, Bai-Hua Xia, No. 300”.

#### Diagnosis.

Head strongly transverse and yellow; clypeus very wide (Fig. 300); mandible large, gradually widened baso-ventrally and resulting in comparatively robust teeth (Fig. 301); occipital carina slightly curved ventrally and remaining removed from hypostomal carina; pronotal side striate; notauli nearly complete, basal half crenulate and gradually reduced posteriorly (Fig. 297); scutellar sulcus wide and coarsely crenulate (Fig. 297); surface of propodeum coarsely and densely rugose and without a transverse carina subbasally (Fig. 298); vein 1r-m of hind wing distinctly oblique and 0.3 times vein 1-M (Fig. 296); second tergite smooth.

#### Description.

Holotype, ♀, length of body 4.2 mm, of fore wing 3.6 mm.

**Figure 295. F69:**
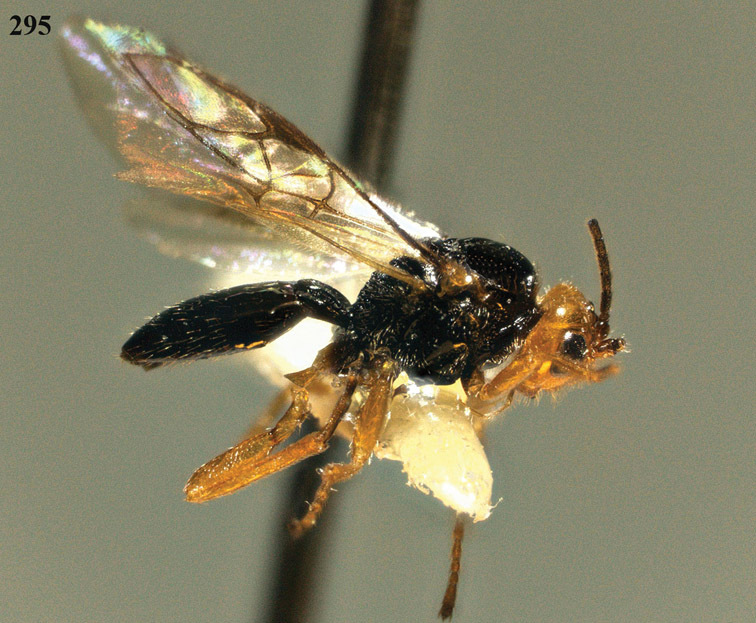
*Phaedrotoma striatinota* sp. n., male, holotype. Habitus lateral.

*Head*. Antenna on unique specimen broken (only 7 segments remaining); third segment 1.1 times as long as fourth segment, length of third and fourth segments 2.0 and 2.0 times their width, respectively (Fig. 295); length of maxillary palp 0.8 times height of head; labial palp segments short; occipital carina far removed from hypo-stomal carina and dorsally absent; hypostomal carina medium-sized; length of eye in dorsal view 0.9 times temple; frons depressed behind antennal sockets and glabrous, smooth (Fig. 302); face smooth, medially broadly elevated (Fig. 300); width of clypeus 3.0 times its maximum height and 0.7 times width of face, clypeus flattened, smooth except for some punctures and its ventral margin thick and distinctly concave (Fig. 301); hypoclypeal depression wide (Fig. 300); malar suture reduced; length of malar space slightly less than basal width of mandible; mandible large, gradually widened baso-ventrally and with a narrow ventral carina (Fig. 301).

*Mesosoma*. Length of mesosoma 1.3 times its height; dorsal pronope shallow (Fig. 302); pronotal side largely striate (Fig. 303); epicnemial area coarsely crenulate dorsally; precoxal sulcus wide, deep and coarsely crenulate, smooth as rest of mesopleuron (Fig. 303); pleural sulcus smooth; notauli nearly complete, crenulate and gradually reduced posteriorly (Fig. 297); mesoscutum smooth and glabrous; medio-posterior depression of mesoscutum elliptical (Fig. 297); scutellar sulcus wide and coarsely crenulate; scutellum smooth, slightly convex medially; surface of propodeum coarsely and densely rugose (Fig. 298).

**Figures 296–303. F70:**
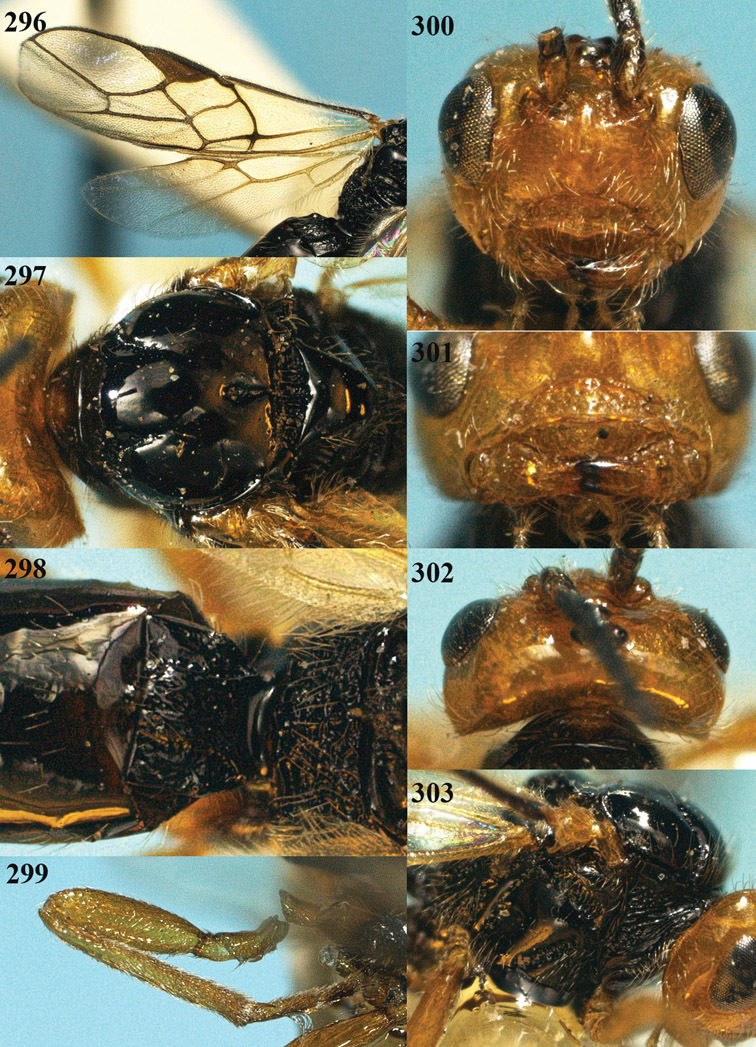
*Phaedrotoma striatinota* sp. n., male, holotype. **296** Wings **297** mesosoma dorsal **298** propodeum and 1^st^-2^nd^ metasomal tergites dorsal **299** hind leg **300** head anterior **301** clypeus and mandible anterior **302** head dorsal **303** mesosoma lateral.

*Wings*. Fore wing (Fig. 296): pterostigma triangular; 1-R1 reaching wing apex and 1.2 times as long as pterostigma; r:3-SR:SR1 = 10:45:93; 2-SR:3-SR:r-m = 37:45:15; 1-M slightly curved, 1-SR+M sinuate and SR1 nearly straight; m-cu and cu-a slightly postfurcal; 1-CU1 hardly widened; first subdiscal cell robust, 3-CU1:CU1b=11:9; apical fifth of M+CU1 unsclerotized. Hind wing (Fig. 296): M+CU:1-M:1r-m = 51:53:17; cu-a straight; m-cu absent; SR present.

*Legs*. Length of femur, tibia and basitarsus of hind leg 3.0, 6.2 and 4.0 times as long as wide, respectively; hind femur and tibia with medium-sized setae (Fig. 299).

*Metasoma*. Length of first tergite 0.9 times its apical width, its surface evenly convex medially, coarsely rugose and with dorsal carinae united near apical fifth of tergite (Fig. 298); second and following tergites smooth and normally sclerotized.

*Colour*. Body black; antenna dark brown; head, tegulae and legs (but hind coxa and hind tarsus brown) yellow; pterostigma and veins brown; wing membrane subhyaline.

#### Distribution.

*China (Hunan).

#### Biology.

Unknown.

#### Etymology.

Etymology. Name derived from “stria” (Latin for “furrow, line”) and “notos” (Greek for “back”), because of the longitudinally striate pronotal sides.

#### Notes.

The new species does not run well in the key by Chen and Weng (2005); it ends near *Xynobius indagatrix* (Weng & Chen, 2005)comb. n. and *Xynobius multiarculatus* (Chen & Weng, 2005) comb. n. Both species have dorsope, which is absent in *Phaedrotoma striatinota*. The pronotal side of both species lacks striae (present in *Phaedrotoma striatinota*) and the male of the new species has normally sclerotized tergites (male holotype of *Xynobius indagatrix* has third-sixth tergites largely membranous).

### Phaedrotoma terga

(Chen & Weng, 2005) comb. n.

http://species-id.net/wiki/Phaedrotoma_terga

Opius (Tolbia) tergus Chen & Weng, 2005: 148, 206, fig. 69.

#### Distribution.

China (Fujian, Hunan).

#### Biology.

Unknown.

### Phaedrotoma vermiculifera

Li & van Achterberg sp. n.

urn:lsid:zoobank.org:act:04A021CC-6CB2-484B-BCD7-D434783373BF

http://species-id.net/wiki/Phaedrotoma_vermiculifera

Figs 304 –313

#### Type material.

Holotype, ♀ (ZUH), “S. China: Hunan, nr Zhangjiajie, Badagong Mts, Bamaoxi, 2–3.VI.2009, 540 m, Xi-Ying Li, RMNH’09”. Paratype, 1 ♀ (RMNH), same label data.

#### Diagnosis.

Malar suture absent and malar space short (Fig. 310); clypeus medium-sized (Fig. 309); second submarginal cell of fore wing medium-sized (Fig. 305); setose part of ovipositor sheath 0.1-0.2 times as long as hind tibia; pronope small and round (Fig. 313); second and third tergites smooth; first metasomal tergite longitudinally costate-striate (Fig. 307); anterior groove of metapleuron smooth; propodeum largely finely vermiculate-rugose or -rugulose (Fig. 307); clypeus slightly protruding medially (Fig. 309); apical half of first tergite widened apically (Fig. 307); occipital carina remaining far removed from hypostomal carina (Fig. 310).

#### Description.

Holotype, ♀, length of body 2.4 mm, of fore wing 2.8 mm.

*Head*. Antenna with 34 segments and 1.5 times as long as fore wing; length of third segment 1.3 times fourth segment, length of third, fourth and penultimate segments 4.0, 3.2, and 2.7 times their width, respectively (Fig. 312); length of maxillary palp 1.1 times height of head; labial palp segments normal, elongate; occipital carina remain far hypostomal carina, crenulate and dorsally absent (Fig. 310); hypostomal carina medium-sized; length of eye in dorsal view 2.8 times temple; frons glabrous, smooth, without pit medially, slightly convex laterally and in front of anterior ocellus; face smooth, rather convex, medially weakly elevated (Fig. 309); width of clypeus twice its maximum height and 0.6 times width of face; clypeus flattened, with some punctures and its ventral margin protruding forwards, thin and convex (Fig. 309); hypoclypeal depression rather large (Fig. 309); malar suture entirely absent and malar space comparatively short; mandible not widened basally, with long and non-protruding ventral carina (Fig. 310).

**Figure 304. F71:**
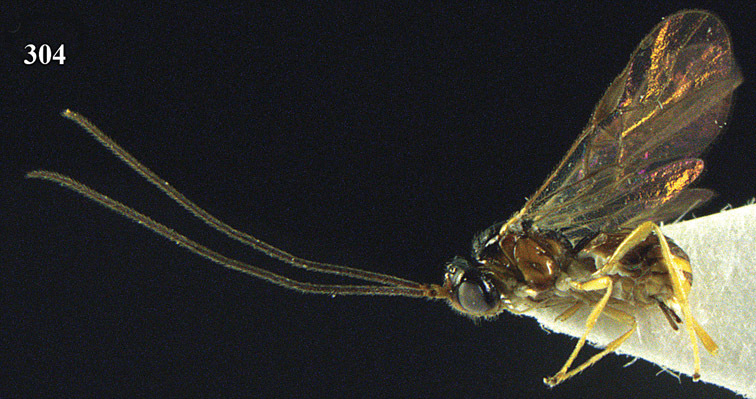
*Phaedrotoma vermiculifera* sp. n., female, holotype. Habitus lateral.

*Mesosoma*. Length of mesosoma 1.2 times its height; dorsal pronope small and round (Fig. 313); pronotal side mainly smooth, but oblique groove distinctly crenulate anteriorly and posterior groove absent (Fig. 304); epicnemial area smooth dorsally; precoxal sulcus only medially impressed, distinctly crenulate and rather wide (Fig. 304); remainder of mesopleuron and pleural sulcus smooth; anterior groove of metapleuron smooth; notauli absent on disc, only anteriorly indicated by shallow depressions (Fig. 306); mesoscutum glabrous except for a few setae along imaginary notaulic courses; medio-posterior depression of mesoscutum absent; lateral carina of mesoscutum absent anteriorly and area (as notauli area anteriorly in lateral view) finely rugulose; scutellar sulcus distinctly crenulate; scutellum smooth and flattened; surface of propodeum largely finely vermiculate-rugose or -rugulose, without distinct carinae (Fig. 307).

**Figures 305–313.**
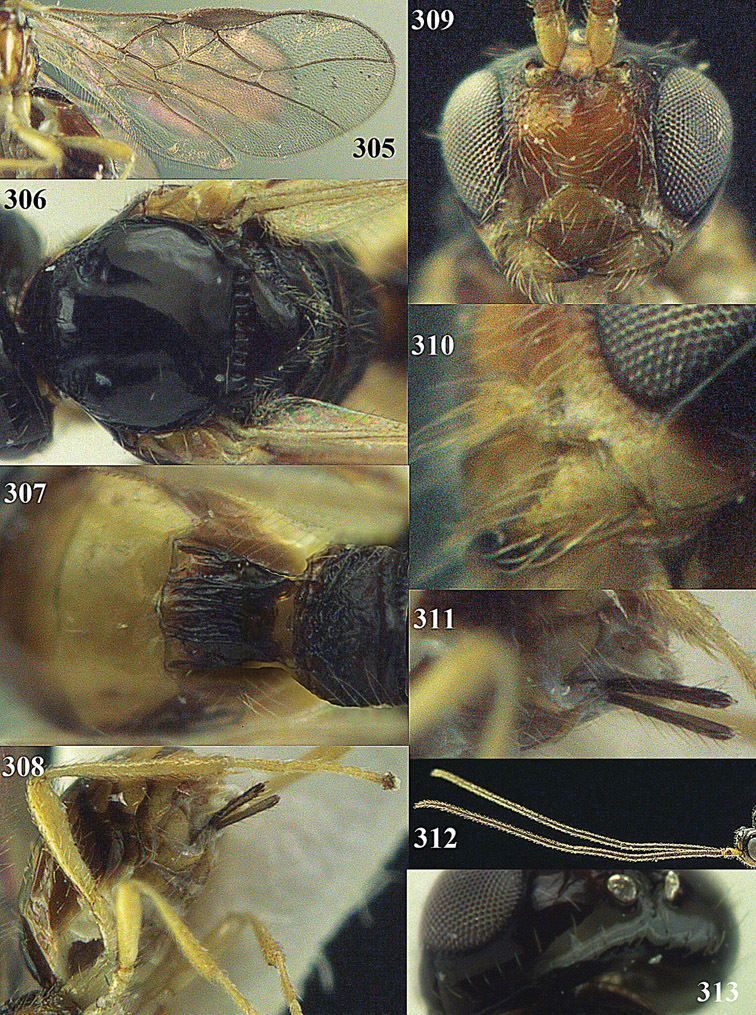
*Phaedrotoma vermiculifera* sp. n., female, holotype. **305** Wings **306** mesosoma dorsal **307** propodeum and 1^st^-2^nd^ metasomal tergites dorsal **308** hind leg **309** head anterior **310** mandible **311** ovipositor sheath **312** antennae **313** pronope dorsal.

*Wings*. Fore wing (Fig. 305): pterostigma wide elliptical, narrowed apically; 1-R1 reaching wing apex and 1.3 times as long as pterostigma; r:3-SR:SR1 = 2:32:74; 2-SR:3-SR:r-m = 21:32:8; 1-M nearly straight; SR1 slightly curved; m-cu subinterstitial or slightly antefurcal; cu-a subinterstitial; first subdiscal cell closed, CU1b short. Hind wing (Fig. 305): M+CU:1-M:1r-m = 5:5:2; cu-a straight; m-cu absent.

*Legs*. Length of femur, tibia and basitarsus of hind leg 4.1, 9.6 and 5.0 times as long as wide, respectively; hind femur and tibia with long setae (Fig. 308).

*Metasoma*. Length of first tergite 1.1 times its apical width, its surface rather convex but flattened medially and largely longitudinally costate-striate, dorsal carinae up to apex of tergite, slightly sinuate (Fig. 307); second and following tergites smooth, division of second with third tergite slightly elevated; length of setose part of ovipositor sheath 0.11 times fore wing and 0.4 times hind tibia; apex of hypopygium rather acute (Figs 304, 311).

*Colour*. Blackish-brown; palpi, mandible, malar space, temple ventrally, tegulae and legs (except infuscate telotarsi) whitish or pale yellowish; clypeus, scapus, propleuron, pronotal side ventrally, second and fourth to sixth tergites, metasoma ventro-apically partly yellow; face, frons medially, mesopleuron, mesosternum (but largely darkened) and metapleuron, brownish-yellow; first tergite largely and remainder of antenna dark brown; third and fourth tergites, pterostigma and veins brown; metasoma ventro-basally pale brown; wing membrane subhyaline.

*Molecular data*. COI, 16S, 28S (CVA4250).

*Variation*. The paratype female is very similar to the holotype, length of body 1.9 mm, of fore wing 2.4 mm; antenna with more than 31 segments (apical segment(s) missing).

#### Distribution.

*China (Hunan).

#### Biology.

Unknown.

#### Etymology.

Name derived from “vermiculus” (Latin for “small worm”) and “ferum” (suffix in Latin meaning carrying or having), because of the densely rugose or rugulose propodeum.

#### Notes.

The new species runs in the key by Chen and Weng (2005) to *Phaedrotoma osculas* (Weng & Chen, 2005) comb. n.*Phaedrotoma vermiculifera* differs by having the malar space comparatively short (Fig. 298), half as long as the basal width of the mandible (0.7 times in *Phaedrotoma osculas*), the surface of the propodeum largely finely vermiculate-rugose or -rugulose (Fig. 296), without distinct carinae (smooth anteriorly and with pair of carinae posteriorly), the length of the mesosoma 1.2 times its height (1.4 times) and the length of maxillary palp 1.1 times height of head (1.3 times).

### Rhogadopsis

Brèthes, 1913 stat. n.

Figs 314 –373

Rhogadopsis Brèthes, 1913: 44; Shenefelt 1975: 1212; Wharton 1987: 66(synonymy with subgenus *Lissosema*). Type species (by monotypy): *Rhogadopsis miniacea* Brèthes, 1913 [examined].
Lissosema Fischer, 1972a: 359. Type species (by original designation): *Opius parvungula* Thomson, 1895 [examined].

#### Diagnosis.

Propodeum with a medio-longitudinal carina anteriorly (Figs 317, 327, 336, 344, 352, 366); vein m-cu of fore wing gradually merging into 2-CU1 and linear with vein 2-M or nearly so; vein 1r-m of hind wing less oblique and 0.7-1.0 times as long as vein 1-M; anterior groove of metapleuron crenulate, but smooth in *Rhogadopsis semiplanata*; vein CU1b of fore wing medium-sized.

#### Biology.

Parasitoids of Agromyzidae.

**Notes.** *Lissosema* Fischer, 1972, is a junior synonym of *Rhogadopsis* Brèthes (Wharton, 1987), its type species has a short malar space with no distinct malar suture. Both names have been used as subgenera in the genus *Opius* (Fischer 1972b; Wharton 1987), but according to the molecular data and the derived wing venation this group forms a separate clade and, therefore, *Rhogadopsis* is elevated to generic rank.

### Rhogadopsis latipennis

Li & van Achterberg sp. n.

urn:lsid:zoobank.org:act:04155CB2-4A2F-4BC5-95A4-07EE11276996

http://species-id.net/wiki/Rhogadopsis_latipennis

Figs 314 –324

#### Type material.

Holotype, ♀ (ZUH), “S. China: Hunan, Yongzhou, Jiangyong, Yuankou, 28.VI.1988, Jian-Ping Liu, No. 184”.

#### Diagnosis.

**Figure 314.**
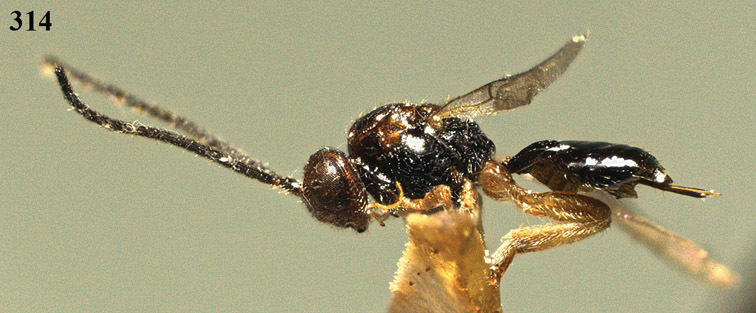
*Rhogadopsis latipennis* sp. n., female, holotype. Habitus lateral.

Length of eye about 2.0 times temple in dorsal view; hypoclypeal depression medium-sized (Fig. 321); ventral half of posterior groove of pronotal side crenulate; precoxal sulcus moderately wide and crenulate (Fig. 314); anterior groove of metapleuron crenulate; medio-posterior depression of mesoscutum large, round and deep (Fig. 317); propodeum with medio-longitudinal carina anteriorly (Fig. 317); vein 1r-m of hind wing about 1.3 times as long as vein 1-M (Fig. 316); area below pterostigma hyaline; first tergite about as long as wide (Fig. 318).

#### Description.

Holotype, ♀, length of body 3.0 mm, of fore wing 2.8 mm.

*Head*. Antenna with 23+ segments; third segment 1.3 times as long as fourth segment, length of third and fourth segments 2.2 and 1.7 times their width, respectively (Fig. 319); length of maxillary palp 0.7 times height of head; labial palp segments slender; occipital carina rather close to hypostomal carina and dorsally absent; hypostomal carina wide; length of eye in dorsal view 2.0 times temple; frons medially convex, depressed behind antennal sockets, smooth and glabrous (Fig. 322); face coarsely punctate, setose and medially widely elevated (Fig. 320); width of clypeus 2.6 times its maximum height and 0.6 times width of face; clypeus convex, protruding forwards and punctate and its ventral margin slightly concave and thick (Figs 320, 321); hypoclypeal depression medium-sized (Fig. 321); malar suture deep; length of malar space 0.6 times basal width of mandible; mandible triangular and with narrow ventral carina (Fig. 321).

*Mesosoma*. Length of mesosoma 1.3 times its height; pronotal side largely smooth, but medial groove and oblique groove coarsely crenulate (Fig. 314); epicnemial area mainly smooth dorsally, but finely crenulate laterally; precoxal sulcus moderately wide and crenulate; remainder of mesopleuron smooth (Fig. 314); pleural sulcus smooth; anterior groove of metapleuron crenulate; notauli absent on disc, only anteriorly indicated by shallow depressions (Fig. 317); mesoscutum glabrous except for row of long setae along notaulic courses; medio-posterior depression of mesoscutum large, round and deep (Fig. 317); scutellar sulcus wide and coarsely crenulate (Fig. 317); scutellum slightly convex medially, smooth; propodeum with nearly complete medio-longitudinal carina and remainder largely reticulate-rugose (Fig. 317).

**Figures 315–324.**
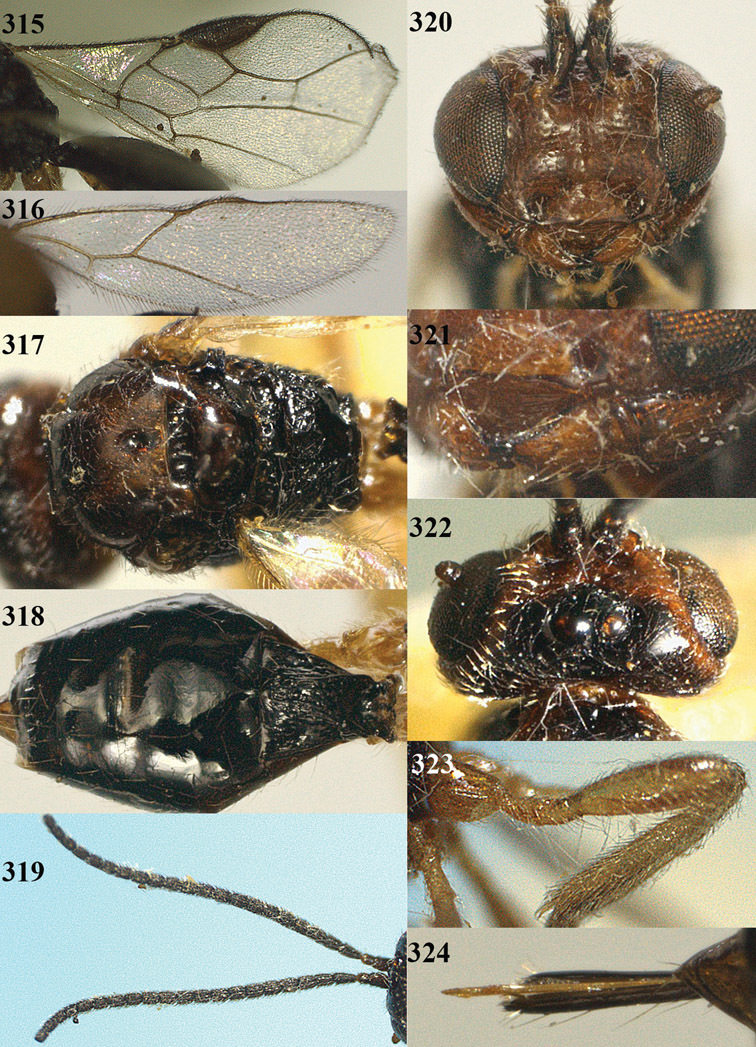
*Rhogadopsis latipennis* sp. n., female, holotype. **315** Fore wing **316** hind wing **317** mesosoma dorsal **318** metasoma dorsal **319** antennae **320** head anterior **321** mandible **322** head dorsal **323** hind leg **324** ovipositor sheath.

*Wings*. Fore wing (Fig. 315): pterostigma triangular; 1-R1 not reaching wing apex and 1.2 times as long as pterostigma; r:3-SR:SR1 = 17:42:67; 2-SR:3-SR:r-m = 30:42:17; r long; 1-M distinctly curved, SR1 nearly straight; m-cu postfurcal; cu-a subinterstitial; first subdiscal cell closed, CU1b medium-sized; apical third of M+CU1 desclerotized. Hind wing (Fig. 316): M+CU:1-M:1r-m = 37:21:27; cu-a straight; m-cu completely absent.

*Legs*. Length of femur, tibia and basitarsus of hind leg 3.5, 7.0 and 4.0 times as long as wide, respectively; hind femur and tibia with medium-sized setae (Fig. 323).

*Metasoma*. Length of first tergite 1.1 times its apical width, its surface longitudinally striate and evenly convex medially (Fig. 318); second and following tergites smooth; length of ovipositor sheath 0.5 times (setose part 0.4 times) as long as hind tibia (Figs 314, 324).

*Colour*. Dark brown; head (except medio-dorsally and posteriorly), notaulic courses, tegulae, ovipositor sheath yellowish-brown; palpi and legs yellow; pterostigma and veins brown; wing membrane subhyaline.

#### Distribution.

*China (Hunan).

#### Etymology.

Name derived from “latus” (Latin for “wide”) and “penna” (Latin for “wing”), because of the wide hind wing.

#### Notes.

The new species runs in the key by Chen and Weng (2005) with some difficulty to *Opius aquacaducus* Chen & Weng, 2005, but *Rhogadopsis latipennis* has normal mandibles (asymmetrical in *Opius aquacaducus*), the medio-posterior depression is round (droplet-shaped) and the scutellum is slightly convex (strongly tuberculate convex). *Rhogadopsis tabidula*(Weng & Chen, 2005) comb. n. and*Rhogadopsis pratellae***(**Weng & Chen, 2005) comb. n. are similar but have vein 1r-m of hind wing less enlarged (about as long as vein 1-M and about 1.3 times in *Rhogadopsis latipennis*) and the setose part of the ovipositor sheath is either shorter (0.3 times hind tibia in *Rhogadopsis tabidula*) or longer (0.6 times hind tibia in *Rhogadopsis pratellae*) than in *Rhogadopsis latipennis* (0.4 times hind tibia). *Rhogadopsis tabidula* has a droplet shaped medio-posterior depression of the mesoscutum (round in *Rhogadopsis latipennis*) and the anterior half of the notauli present on the disc (as in *Rhogadopsis latipennis*; absent in *Rhogadopsis tabidula*).

### Rhogadopsis longicaudifera

Li & van Achterberg sp. n.

urn:lsid:zoobank.org:act:5A361F3E-3FD2-419E-9DE5-5BE285A03279

http://species-id.net/wiki/Rhogadopsis_longicaudifera

Figs 325 –333

#### Type material.

Holotype, ♀ (ZUH), “S. China: Hunan, Yongzhou, Jiangyong, Yuankou, 28.V.1988, Jian-Ping Liu, No. 181”.

#### Diagnosis.

Posterior groove of pronotal side crenulate; medio-posterior depression of mesoscutum absent (Fig. 327); anterior groove of metapleuron crenulate; propodeum with medio-longitudinal carina anteriorly (Fig. 328); vein m-cu of fore wing postfurcal (Fig. 326); vein 1r-m of hind wing about 0.9 times as long as vein 1-M; vein CU1b of fore wing medium-sized (Fig. 326); first tergite normal and without median carina (Fig. 328); length of setose part of ovipositor sheath 0.5 times fore wing and twice as long as hind tibia (Fig. 333).

#### Description.

Holotype, ♀, length of body 2.1 mm, of fore wing 2.6 mm.

*Head*. Antenna with 27 segments and 1.1 times as long as fore wing; third segment 1.2 times as long as fourth segment, length of third, fourth and penultimate segments 4.0, 3.4 and 1.3 times their width, respectively (Fig. 332); length of maxillary palp 0.8 times height of head; labial palp segments short; occipital carina close to hypostomal carina and dorsally absent; hypostomal carina wide; length of eye in dorsal view 1.3 times temple; frons flat, smooth and glabrous; face smooth, medially elevated (Fig. 330); width of clypeus 1.8 times its maximum height and 0.4 times width of face; clypeus moderately convex, largely smooth, slightly concave and thin (Fig. 330); hypoclypeal depression medium-sized (Fig. 330); malar suture absent; length of malar space about equal to basal width of mandible; mandible triangular and with narrow ventral carina (Fig. 331).

*Mesosoma*. Length of mesosoma 1.2 times its height; dorsal pronope obsolescent; pronotal side smooth; epicnemial area smooth dorsally; precoxal sulcus wide and crenulate; remainder of mesopleuron smooth; pleural sulcus smooth; anterior groove of metapleuron smooth; notauli absent on disc, only anteriorly with pair of narrow and short smooth impressions (Fig. 327); mesoscutum glabrous; medio-posterior depression of mesoscutum absent; scutellar sulcus moderately wide and crenulate; scutellum slightly convex medially, smooth, glabrous; propodeum with nearly complete medio-longitudinal carina and remainder largely rugulose (Fig. 327).

*Wings*. Fore wing (Fig. 326): pterostigma triangular; 1-R1 ending at wing apex and 1.3 times as long as pterostigma; r:3-SR:SR1 = 10:30:80; 2-SR:3-SR:r-m = 25:30:11; r widened; 1-M and SR1 nearly straight; m-cu postfurcal; cu-a postfurcal; first subdiscal cell closed, CU1b short; apical quarter of M+CU1 sclerotized. Hind wing (Fig. 326): M+CU:1-M:1r-m = 21:20:17; cu-a straight; m-cu completely absent.

*Legs*. Length of femur, tibia and basitarsus of hind leg 4.0, 7.1 and 5.0 times as long as wide, respectively; hind femur and tibia with medium-sized setae (Fig. 329).

**Figure 325.**
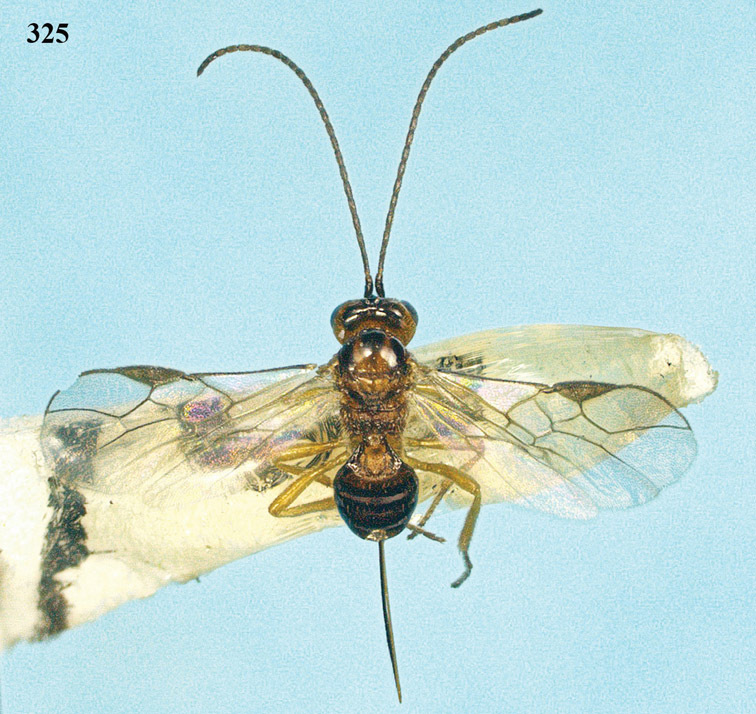
*Rhogadopsis longicaudifera* sp. n., female, holotype. Habitus lateral.

*Metasoma*. Length of first tergite 1.3 times its apical width, its surface evenly gradually convex medially, longitudinally rugose and with dorsal carinae separated and up to apex (Fig. 328); basal half of second tergite densely and finely granulate; third tergite and following tergites smooth; length of setose part of ovipositor sheath 0.5 times fore wing and twice hind tibia (Figs 325, 333).

*Colour*. Yellowish-brown; antenna, pterostigma, veins, vertex, mesoscutum medially and laterally, third and following tergites, tarsi and ovipositor sheath dark brown; remainder of legs and palpi yellowish. wing membrane subhyaline.

*Molecular data*. None.

**Figures 326–333.**
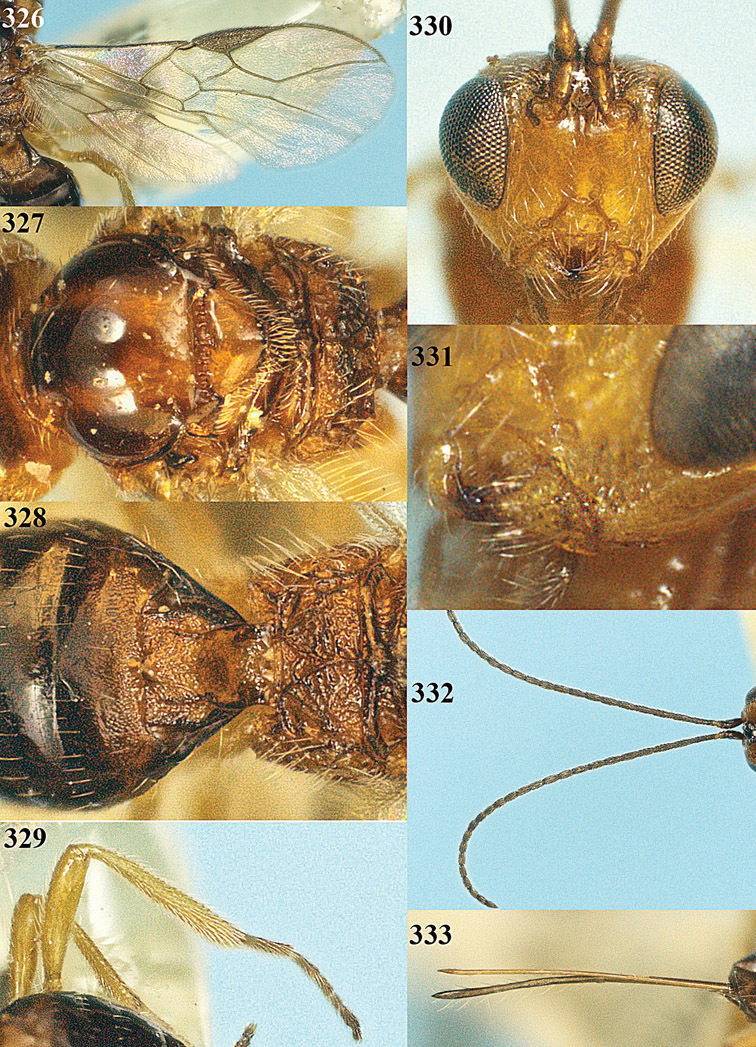
*Rhogadopsis longicaudifera* sp. n., female, holotype. **326** Wings **327** mesosoma dorsal **328** propodeum and 1^st^-2^nd^ metasomal tergites dorsal **329** hind leg **330** head anterior **331** mandible **332** antennae **333** ovipositor sheath.

#### Distribution.

*China (Hunan).

#### Biology.

Unknown.

#### Etymology.

Name derived from “longus” (Latin for “long”), “caudus” (Latin for “tail”) and “ferum” (suffix in Latin meaning carrying or having), because of the long ovipositor sheath.

#### Notes.

The new species runs in the key by Chen and Weng (2005) to *Rhogadopsis dimidia* (Chen & Weng, 2005) comb. n. or *Xynobius complexus* (Weng & Chen, 2005) comb. n. The new species differs from the latter because of absence of the dorsope (present in *Xynobius complexus*), no notauli on the mesoscutal disc (entirely impressed) and the normally sclerotized second and following tergites (partly desclerotized). The new species differs from *Rhogadopsis dimidia* by having the length of eye 1.3 times temple in dorsal view (about 7 times in *Rhogadopsis dimidia*), basal half of notauli absent on mesoscutal disc (present and crenulate), and second and third tergites granulate (longitudinally striate). *Rhogadopsis diutia* (Chen & Weng, 2005) comb. n. is similar, but *Rhogadopsis longicaudifera* has the ovipositor sheath about 4 times as long as the first tergite (about half as long in *Rhogadopsis diutia*), the pleural sulcus smooth (crenulate), length of the malar space about equal to basal width of mandible (0.6 times) and the pterostigma triangular (elliptical).

### Rhogadopsis longuria

(Chen & Weng, 2005) comb. n.

http://species-id.net/wiki/Rhogadopsis_longuria

Figs 334 –341

Opius longurius Chen & Weng, 2005: 95, 97–98 (fig. 40), 100, 183, 197, photos 145–149.

#### Type material.

Holotype, ♀ (FAFU), “[China:] Fujian, Wuyi Mt., Sangang, 30.VI.1988, Zhang Xia-bin”. Paratypes (FAFU): 1 ♀, topotypic, but 14.V.1994, 1 ♂ id., but Dazhulan, 18.VI.1993, 1 ♀ id., but Guhuangkeng, 25.VI.1993 and 1 ♂ “[China:] Fujian, Longqi Mt., Jiangle, 11.VII.1994, Wu Zu-shan”.

#### Additional material.

2 ♂ (ZUH, RMNH), “S. China: Hunan, nr Zhangjiajie, Badagong Mts, Bamaoxi, 2–3.VI.2009, 540 m, Xi-Ying Li, RMNH’09”.

#### Diagnosis.

Vein 1r-m of hind wing 0.7–1.0 times as long as vein 1-M (Fig. 335); anterior groove of metapleuron crenulate; posterior groove of pronotal side largely absent and remainder nearly smooth; first tergite elongate and with median carina (Fig. 337); medio-posterior depression of mesoscutum absent (Fig. 336).

#### Description.

Male from Bamaoxi, length of body 2.8 mm, of fore wing 2.9 mm.

*Head*. Antenna with 32 segments and 1.3 times as long as fore wing; third segment 1.3 times as long as fourth segment, length of third, fourth and penultimate segments 4.0, 3.0 and 2.5 times their width, respectively (Fig. 338); length of maxillary palp 1.2 times height of head; labial palp segments slender; occipital carina rather close to hypostomal carina and dorsally absent; hypostomal carina wide; length of eye in dorsal view 3.8 times temple; frons rather depressed anteriorly and glabrous, largely smooth; face largely coarsely punctate and with distinct smooth interspaces, medially elevated (Fig. 339); width of clypeus 2.9 times its maximum height and 0.65 times width of face; clypeus rather convex, distinctly protruding forwards and coarsely punctate and its ventral margin slightly concave and thin, acute (Fig. 339); hypoclypeal depression large (Fig. 339); malar suture absent; length of malar space 0.3 times basal width of mandible; mandible triangular and with narrow ventral carina (Fig. 340).

**Figure 334.**
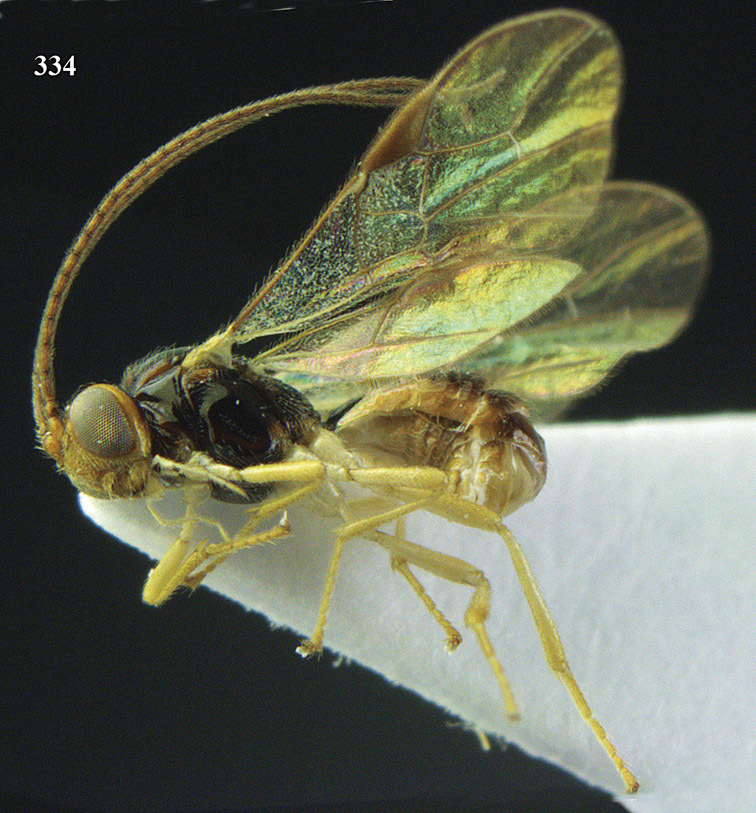
*Rhogadopsis longuria* (Chen & Weng), male, Hunan, Bamaoxi. Habitus lateral.

*Mesosoma*. Length of mesosoma 1.3 times its height; dorsal pronope absent; pronotal side smooth, but posterior groove partly impressed and narrowly superficially crenulate (Fig. 334); epicnemial area mainly smooth dorsally except for some fine punctures; precoxal sulcus medially oblique and moderately crenulate (Fig. 334); rest of mesopleuron smooth; pleural sulcus smooth; mesosternal sulcus shallow and moderately crenulate; anterior groove of metapleuron crenulate; notauli absent on disc, only anteriorly with pair of narrow and short smooth impressions; mesoscutum glabrous except for row of setae along imaginary notaulic courses and strongly shiny (Fig. 336); medio-posterior depression of mesoscutum absent; scutellar sulcus moderately wide and coarsely crenulate (Fig. 336); scutellum slightly convex medially, smooth; propodeum with nearly complete medio-longitudinal carina and remainder largely smooth (Fig. 336).

**Figures 335–341.**
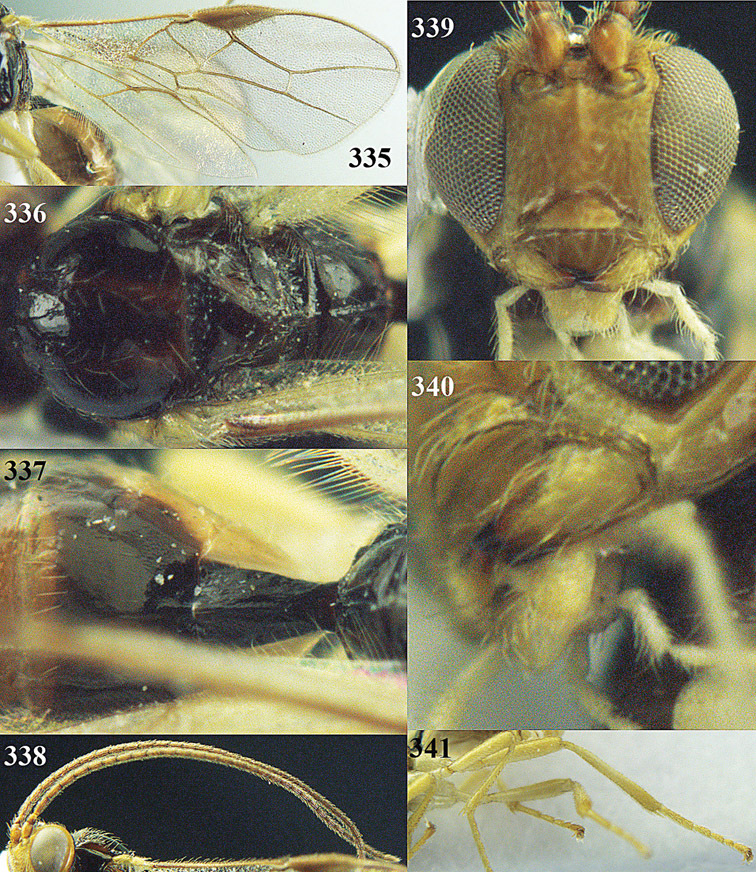
*Rhogadopsis longuria* (Chen & Weng), male, Hunan, Bamaoxi. **335** Wings **336** mesosoma dorsal **337** propodeum and 1^st^-2^nd^ metasomal tergites dorsal **338** antennae **339** head anterior **340 **mandible **341** hind leg.

*Wings*. Fore wing (Fig. 335): pterostigma triangular; 1-R1 ending at wing apex and 1.4 times as long as pterostigma; r:3-SR:SR1 = 4:41:69; 2-SR:3-SR:r-m = 28:41:12; r widened; 1-M and SR1 slightly curved; m-cu narrowly postfurcal; cu-a subinterstitial; first subdiscal cell closed, CU1b medium-sized; apical quarter of M+CU1 sclerotized. Hind wing (Fig. 335): M+CU:1-M:1r-m = 20:17:13; cu-a straight; m-cu completely absent.

*Legs*. Length of femur, tibia and basitarsus of hind leg 4.5, 8.5 and 5.2 times as long as wide, respectively; hind femur and tibia with medium-sized setae (Fig. 341).

*Metasoma*. Length of first tergite 1.5 times its apical width, its surface keel-like convex and largely smooth, its dorsal carinae united at basal 0.3 and with long median carina (Fig. 337); second suture slightly indicated; second and following tergites smooth.

*Colour*. Dark brown, head (except medio-dorsally and posteriorly), scapus and apically pedicellus brownish-yellow; notaulic courses, mesoscutum laterally, mesopleuron antero-dorsally, streak below precoxal sulcus, apex of second, apex and base of following tergites yellowish-brown; pterostigma dark brown; remainder of antenna and veins brown; palpi, tegulae, legs and metasoma ventrally pale yellow; wing membrane subhyaline.

*Variation*. Second male from Bamaoxi has 34 antennal segments, length of fore wing 3.0 mm and surroundings of medio-longitudinal carina of propodeum finely crenulate.

*Molecular data*. None.

#### Distribution.

China (Fujian, Hunan).

#### Biology.

Unknown.

#### Notes.

The males run in the key by Chen and Weng (2005) to *Opius diutius* Chen & Weng, 2005. *Rhogadopsis longuria* differs by having the propodeum with a nearly complete medio-longitudinal carina and remainder largely smooth (entirely reticulate-rugose in *Opius diutius*), the width of the clypeus about 3 times its height (about twice), the length of the malar space 0.3 times the basal width of mandible (0.6 times) and the first tergite with a keel-like convexity medially (flattened medially and without keel).

### Rhogadopsis maculosa

Li, van Achterberg & Tan sp. n.

urn:lsid:zoobank.org:act:DBEDD3C3-4FB3-429A-853F-752F87348880

http://species-id.net/wiki/Rhogadopsis_maculosa

Figs 342 –350

#### Type material.

Holotype, ♂ (ZUH), “S. China: Hunan, Chenzhou, Yizhang, Mang Mts, 7.V.1989, Ben-Zhu Dai, No. 301”.

#### Diagnosis.

Length of body about 3.8 mm; antennal segments about 43; area below pterostigma slightly infuscate; length of eye in dorsal view about equal to temple in dorsal view; mesoscutum about as long as wide; anterior groove of metapleuron crenulate; precoxal sulcus widely crenulate (Fig. 349); medio-posterior depression of mesoscutum elongate (Fig. 344); propodeum with medio-longitudinal carina anteriorly (Fig. 345); vein CU1b of fore wing medium-sized; vein 1r-m of hind wing 0.6 times as long as vein 1-M (Fig. 343); second submarginal cell of fore wing narrowed apically; first tergite about as long as wide (Fig. 345).

#### Description.

Holotype, ♂, length of body 3.8 mm, of fore wing 4.1 mm.

**Figure 342.**
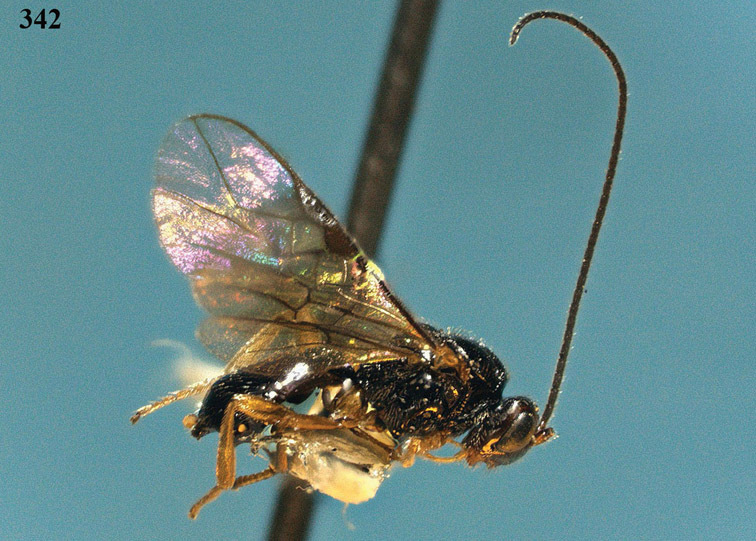
*Rhogadopsis maculosa* sp. n., male, holotype. Habitus lateral.

*Head*. Antenna with 43 segments and 1.3 times as long as fore wing; third segment 1.2 times as long as fourth segment, length of third, fourth and penultimate segments 3.0, 2.5 and 1.5 times their width, respectively (Figs 342, 350); length of maxillary palp 1.1 times height of head; labial palp segments slender; occipital carina moderately close to hypostomal carina and medio-dorsally absent; hypostomal carina wide; length of eye in dorsal view about equal to temple; frons medially convex, depressed behind antennal sockets, largely smooth and glabrous; face largely punctate, medially distinctly elevated (Fig. 347); width of clypeus 2.7 times its maximum height and 0.6 times width of face; clypeus rather convex, distinctly protruding forwards and coarsely punctate and its ventral margin concave and thick (Fig. 347); hypoclypeal depression large (Fig. 347); malar suture absent; length of malar space 0.7 times basal width of mandible; mandible triangular and with narrow ventral carina (Fig. 348).

*Mesosoma*. Length of mesosoma 1.3 times its height; dorsal pronope absent; pronotal side smooth, but medial groove and oblique groove coarsely crenulate (Fig. 349); epicnemial area mainly smooth dorsally except for some fine punctures; precoxal sulcus widely crenulate (Fig. 349); rest of mesopleuron smooth; pleural sulcus smooth; notauli absent on disc, only anteriorly present with pair of short smooth impressions (Fig. 344); mesoscutum glabrous except for row of setae along imaginary notaulic courses; medio-posterior depression of mesoscutum elongate (Fig. 344); scutellar sulcus wide and coarsely crenulate (Fig. 344); scutellum slightly convex medially, smooth; propodeum with nearly complete medio-longitudinal carina and remainder largely reticulate-rugose, posteriorly areolate (Fig. 345).

*Wings*. Fore wing (Fig. 343): pterostigma triangular; 1-R1 reaching wing apex and 1.3times as long as pterostigma (Fig. 343); r:3-SR:SR1 = 7:60:100; 2-SR:3-SR:r-m = 40:60:16; 1-M slightly curved; 1-SR+M sinuate and SR1 nearly straight; m-cu postfurcal; cu-a subinterstitial; first subdiscal cell closed, 3-CU1:CU1b=2:1; apical quarter of M+CU1 sclerotized. Hind wing (Fig. 343): M+CU:1-M:1r-m = 33:45:27; cu-a straight; m-cu completely absent.

*Legs*. Length of femur, tibia and basitarsus of hind leg 4.0, 8.0 and 5.0 times as long as wide, respectively; hind femur and tibia with medium-sized setae. (Fig. 346)

*Metasoma*. Length of first tergite about equal to its apical width, its surface evenly convex medially, dorsally coarsely rugose and laterally with longitudinally striate; dorsal carinae protruding, remain separated from each other and reaching apex of tergite (Fig. 345); second tergite mainly smooth (except for some indistinct striae) and following tergites entirely smooth.

*Colour*. Black; antenna (but scapus and pedicellus yellowish-brown), head (but near eyes yellowish-brown), second and following tergites dark brown; clypeus and mandible yellowish-brown; palpi, tegulae and legs yellow; pterostigma and veins brown; wing membrane subhyaline, but area below pterostigma slightly infuscate.

#### Distribution.

*China (Hunan).

#### Biology.

Unknown.

#### Etymology.

Name derived from “macula” (Latin for “spot”), because of the faintly spotted fore wings.

#### Notes.

The new species does not run to any species in the key by Chen and Weng (2005). Examination of the holotypes in FAFU (Fuzhou) showed that *Rhogadopsis tabidula*(Weng & Chen, 2005) comb. n.and *Rhogadopsis pratellae***(**Weng & Chen, 2005) comb. n.are similar. However, both have fewer antennal segments (24 and 29, respectively), the area below the pterostigma is subhyaline and *Rhogadopsis tabidula* has the third tergite basally narrowly striate.

**Figure:**
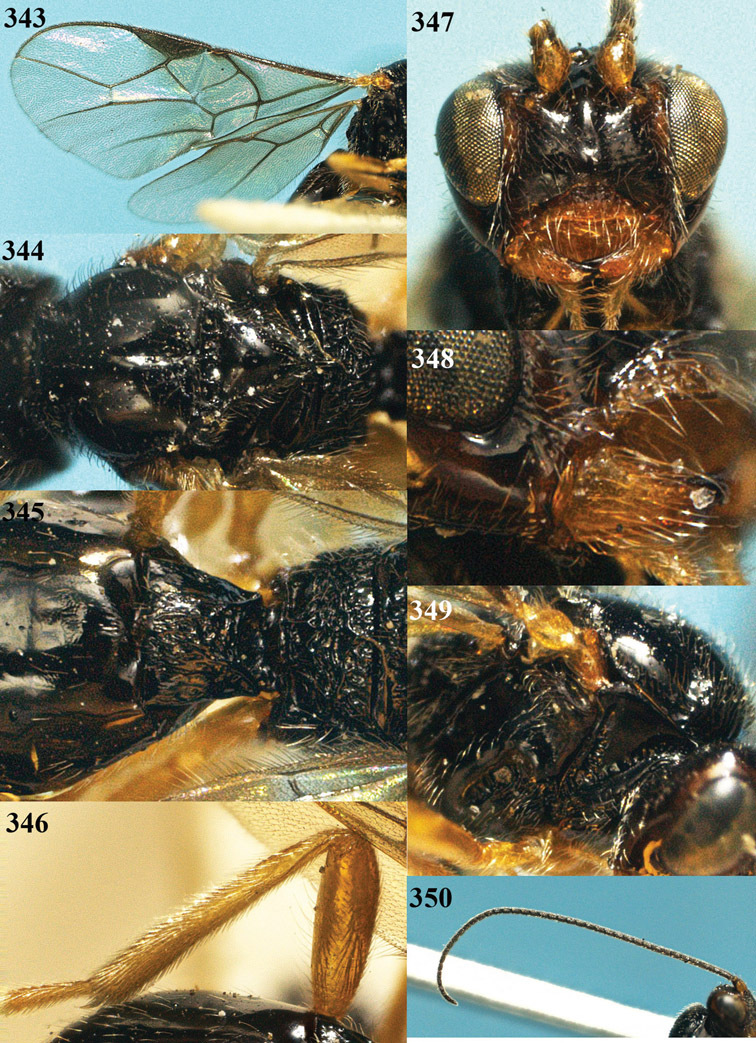
**Figures 343–350.** *Rhogadopsis maculosa* sp. n., male, holotype. **343** Wings **344** mesosoma dorsal **345 **propodeum and 1^st^-2^nd^ metasomal tergites dorsal **346** hind leg **347** head anterior **348** mandible **349** mesosoma lateral **350** antenna.

### Rhogadopsis obliqua

Li & van Achterberg sp. n.

urn:lsid:zoobank.org:act:A3642234-43D0-400A-9E5B-06935644688A

http://species-id.net/wiki/Rhogadopsis_obliqua

Figs 351 –362

#### Type material.

Holotype, ♀ (ZUH), “S. China: Hunan, nr Zhangjiajie, Badagong Mts, Tian Ping Mt., 9–13.vii.2009, 550 m, Xi-Ying Li, RMNH’10”, “CVA4241, sp. 7”. Paratype, 1 ♂ (RMNH), same locality label data.

#### Diagnosis.

**Figure 351.**
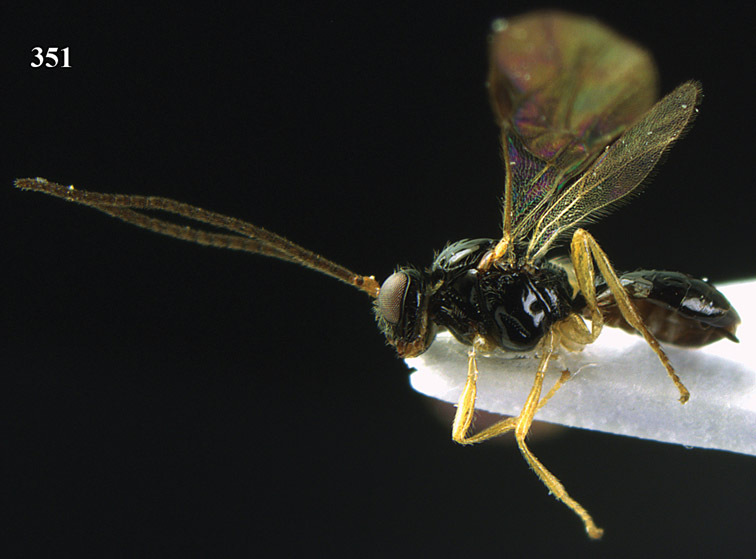
*Rhogadopsis obliqua* sp. n., female, holotype. Habitus lateral.

Vein 1r-m of hind wing 0.7–1.0 times as long as vein 1-M (Fig. 352); posterior groove of pronotal side and anterior groove of metapleuron crenulate (Fig. 351); ventral half of posterior groove of pronotal side crenulate; first tergite normal; medio-posterior depression of mesoscutum present (Fig. 353); vein m-cu of fore wing slightly longer than vein 2-SR+M (Fig. 352); basal cell of hind wing wide and vein 1r-m of hind wing 0.8-1.0 times as long as vein 1-M (Fig. 352); second and third tergites smooth (Fig. 354).

#### Description.

Holotype, ♀, length of body 2.5 mm, of fore wing 2.6 mm.

*Head*. Antenna with 24 segments and as long as fore wing; third segment 1.3 times as long as fourth segment, length of third, fourth and penultimate segments 2.5, 2.0 and 1.6 times their width, respectively (Figs 356, 357); length of maxillary palp 0.9 times height of head; labial palp segments normal; occipital carina rather close to hypostomal carina and dorsally absent; hypostomal carina narrow; length of eye in dorsal view 3.8 times temple; frons slightly depressed behind antennal sockets and glabrous, smooth; face smooth, medially elevated (Fig. 358); width of clypeus 2.1 times its maximum height and 0.55 times width of face; clypeus rather convex, slightly protruding forwards and largely smooth (except for some punctures) and its ventral margin slightly concave and obtuse (Fig. 358); hypoclypeal depression rather large (Fig. 360); malar suture deep; length of malar space 0.6 times basal width of mandible; mandible triangular and with narrow ventral carina (Fig. 359).

*Mesosoma*. Length of mesosoma 1.2 times its height; dorsal pronope large, deep and round (Fig. 362); pronotal side largely smooth, but posterior groove entirely crenulate and oblique groove with some crenulae (Fig. 351); epicnemial area mainly smooth dorsally, with some minute punctures; precoxal sulcus medially oblique and moderately crenulate (Fig. 351); rest of mesopleuron smooth; pleural sulcus smooth; mesosternal sulcus deep and finely crenulate; anterior groove of metapleuron crenulate; notauli absent on disc, only anteriorly with pair of short smooth impressions (Fig. 353); meso-scutum glabrous and strongly shiny; medio-posterior depression of mesoscutum round and medium-sized in a shallow longitudinal impression (Fig. 353); scutellar sulcus widely crenulate; scutellum slightly convex medially, smooth; propodeum with short medio-longitudinal carina and irregular coarsely reticulate-rugose (Fig. 353).

*Wings*. Fore wing (Fig. 352): pterostigma triangular; 1-R1 ending at wing apex and 1.5 times as long as pterostigma; r:3-SR:SR1 = 2:23:32; 2-SR:3-SR:r-m = 14:23:6; r slender; 1-M and SR1 slightly curved; m-cu far postfurcal; cu-a interstitial; first subdiscal cell closed, CU1b rather long; apical third of M+CU1 sclerotized. Hind wing (Fig. 352): M+CU:1-M:1r-m = 3:2:2; cu-a straight; m-cu present as faint unpigmented trace.

*Legs*. Length of femur, tibia and basitarsus of hind leg 4.5, 9.0 and 7.0 times as long as wide, respectively; hind femur with long and tibia with medium-sized setae (Fig. 355).

*Metasoma*. Length of first tergite 1.1 times its apical width, its surface convex and densely reticulate-rugose, its dorsal carinae united at apex of tergite (in paratype at basal 0.3), straight (Fig. 354); second suture slightly indicated; second and third tergites smooth; length of setose part of ovipositor sheath 0.06 times fore wing and 0.2 times length of hind tibia (Fig. 361).

*Colour*. Black; antenna (but scapus yellowish laterally and ventrally) and metasoma (except first tergite) ovipositor sheath and pterostigma dark brown; clypeus and mandible largely chestnut-brown; palpi, tegulae and legs (but hind tarsus and telotarsi darkened) pale yellow; veins brown; wing membrane subhyaline.

*Variation*. The male paratype has length of fore wing 2.7 mm, antenna with 29 similar short antennal segments and 1.1 times as long as fore wing (Fig. 357), inner apex of hind tibia dark brown and vein 1r-m of hind wing 0.8 times as long as vein 1-M.

*Molecular data*. COI, 16S, 28S (CVA4241).

**Figure:**
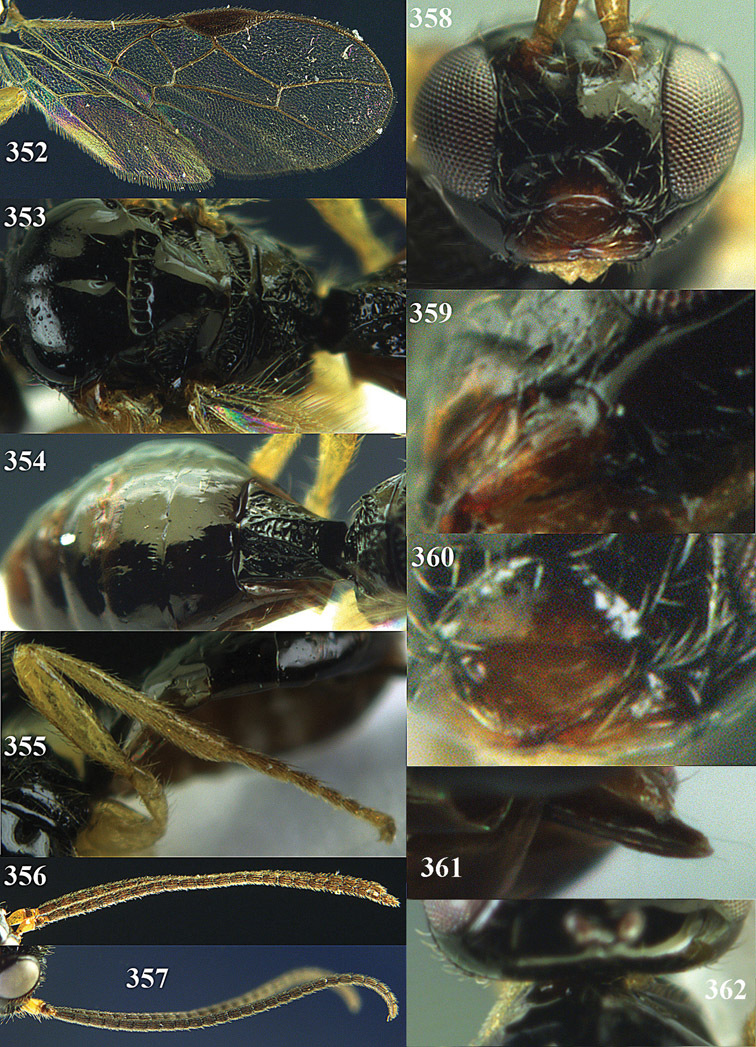
**Figures 352–362.** *Rhogadopsis obliqua* sp. n., female but 357 male paratype, holotype. **352** wings **353** mesosoma dorsal **354** propodeum and 1^st^-2^nd^ metasomal tergites dorsal **355** hind leg **356**, **357** antennae **358** head anterior **359** mandible **361** ovipositor sheath **362** pronope dorsal.

#### Distribution.

*China (Hunan).

#### Biology.

Unknown.

#### Etymology.

Name derived from “obliquus” (Latin for “slanting”), because of the more or less oblique carinae of the propodeum.

#### Notes.

The new species runs in the key by Chen and Weng (2005) to *Opius aquacaducus* Chen & Weng, 2005. *Rogadopsis obliqua* differs by having the width of the clypeus 2.1 times its height (about 3.1 times in *Opius aquacaducus*), the medio-posterior depression of the mesoscutum round (teardrop-shaped), the propodeum mainly irregularly coarsely reticulate-rugose (densely punctate), the length of malar space 0.6 times the basal width of mandible (0.9 times), the length of the eye in dorsal view 3.8 times the temple (equal) and the antenna with 24–29 segments (34–36 segments).

The long vein m-cu of the fore wing is shared with *Rhogadopsis sculpta* (Chen & Weng, 2005) comb. n., but *Rhogadopsis sculpta* has the anterior half of the notauli linearly impressed and nearly smooth, face laterally and dorsally yellowish-brown; malar space shorter, vein r of fore wing oblique, second submarginal cell of fore wing is more elongate.

### Rhogadopsis sculpturator

Li & van Achterberg sp. n.

urn:lsid:zoobank.org:act:1C517906-274E-4502-9C60-A983183FFB94

http://species-id.net/wiki/Rhogadopsis_sculpturator

Figs 363 –373

#### Type material.

Holotype, ♂ (ZUH), “S. China: Hunan, nr Zhangjiajie, Badagong Mts, Bamaoxi, 2–3.VI.2009, 540 m, Xi-Ying Li, RMNH’09”.

#### Diagnosis.

Anterior groove of metapleuron and ventral half of posterior groove of pronotal side crenulate; first tergite normal; medio-posterior depression of meso-scutum present (Fig. 366); vein m-cu of fore wing distinctly longer than vein 2-SR+M (Fig. 364); basal cell of hind wing comparatively narrow and vein 1r-m of hind wing 0.7 times as long as vein 1-M (Fig. 364); second and third tergites finely longitudinally rugulose (Fig. 367).

#### Description.

Holotype, ♂, length of body 2.3 mm, of fore wing 2.4 mm.

*Head*. Antenna with 28 segments and 1.3 times as long as fore wing; third segment 1.4 times as long as fourth segment, length of third, fourth and penultimate segments 4.3, 3.2 and 2.2 times their width, respectively (Fig. 373); length of maxillary palp 1.2 times height of head; labial palp segments normal; occipital carina rather close to hypostomal carina and dorsally absent; hypostomal carina narrow; length of eye in dorsal view 4.0 times temple; frons slightly depressed behind antennal sockets and glabrous, smooth; face smooth, medially elevated (Fig. 369); width of clypeus twice its maximum height and 0.5 times width of face; clypeus rather convex, slightly protruding forwards and largely smooth (except for some punctures) and its ventral margin slightly concave and obtuse (Figs 369, 370, 371); hypoclypeal depression rather large (Fig. 369); malar suture deep; mandible triangular and with narrow ventral carina (Fig. 369).

**Figure 363.**
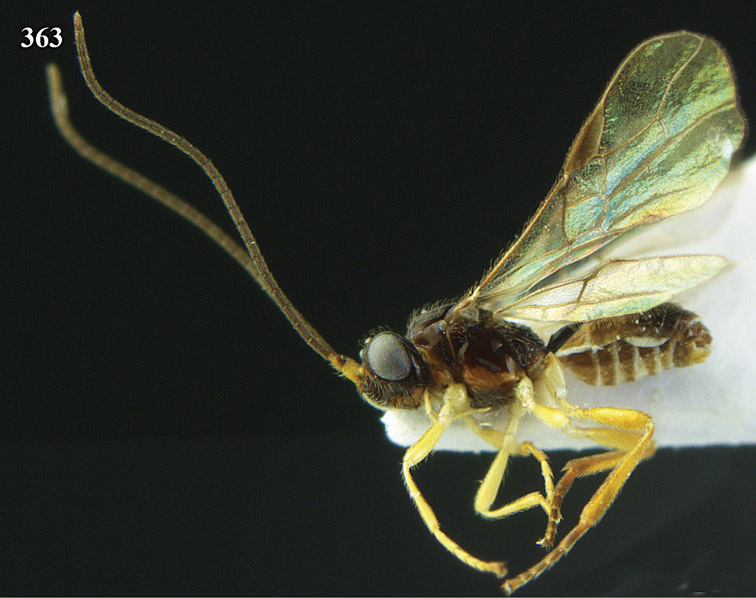
*Rhogadopsis sculpturator* sp. n., male, holotype. Habitus lateral.

*Mesosoma*. Length of mesosoma 1.2 times its height; dorsal pronope small, shallow and round (Fig. 372); pronotal side largely smooth, but ventral half of posterior groove finely crenulate (Fig. 363); epicnemial area mainly smooth dorsally; precoxal sulcus medially oblique and finely crenulate (Fig. 363); rest of mesopleuron smooth; pleural sulcus smooth; mesosternal sulcus deep and finely crenulate; anterior groove of metapleuron crenulate; notauli absent on disc, only anteriorly with pair of narrow and short smooth impressions (Fig. 366); mesoscutum glabrous except for row of setae along imaginary notaulic courses and strongly shiny; medio-posterior depression of mesoscutum droplet-shaped, medium-sized (Fig. 366); scutellar sulcus wide but only finely punctate (Fig. 366); scutellum slightly convex medially, smooth; propodeum with short medio-longitudinal carina anteriorly with some irregular rugae and rugulae, posteriorly depressed and largely smooth, except for some fine transverse aciculae (Fig. 366).

**Figures 364–373.**
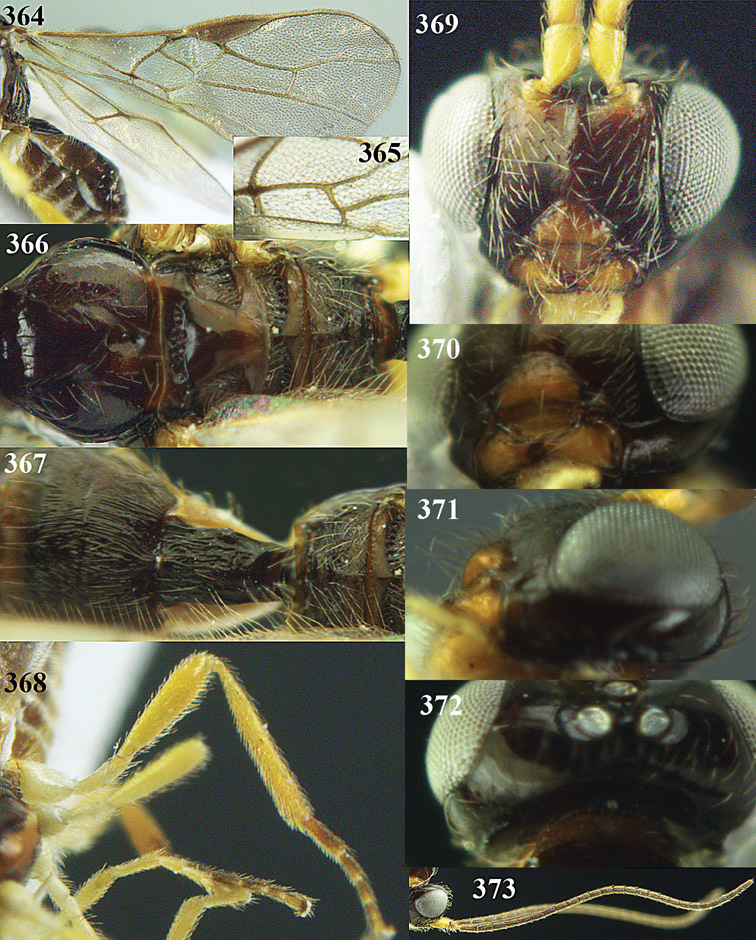
*Rhogadopsis sculpturator* sp. n., male, holotype. **364** Wings **365** detail first subdiscal cell of fore wing **366** mesosoma dorsal **367** propodeum and 1^st^-3^rd^ metasomal tergites dorsal **368** hind leg **369** head anterior **370** mandible **371** clypeus lateral **372** pronope dorsal **373** antennae.

*Wings*. Fore wing (Fig. 364): pterostigma triangular; 1-R1 ending at wing apex and 1.4 times as long as pterostigma; r:3-SR:SR1 = 3:36:52; 2-SR:3-SR:r-m = 20:36:15; r slightly widened; 1-M and SR1 slightly curved; m-cu far postfurcal; cu-a interstitial; first subdiscal cell closed, CU1b medium-sized (Fig. 365); apical third of M+CU1 sclerotized. Hind wing (Fig. 364): M+CU:1-M:1r-m = 20:16:11; cu-a straight; m-cu present as a long faint unpigmented trace.

*Legs*. Length of femur, tibia and basitarsus of hind leg 4.0, 7.4 and 5.2 times as long as wide, respectively; hind femur with long and tibia with medium-sized setae (Fig. 368).

*Metasoma*. Length of first tergite 1.4 times its apical width, its surface convex and finely longitudinally rugose, its dorsal carinae united at basal 0.3 (Fig. 367); second suture slightly indicated; second and basal half of third tergite finely longitudinally aciculate (Fig. 367).

*Colour*. Dark brown, including antenna (but scapus and ventrally pedicellus yellow); head dorsally, first, second and basal half of third tergites black; clypeus, tegulae, mandible, pronotum ventrally, propleuron, area below precoxal sulcus and metasoma ventrally yellowish-brown; pterostigma and veins brown; palpi, and legs (but hind tarsus darkened) pale yellow; wing membrane subhyaline.

*Molecular data*. None.

#### Distribution.

*China (Hunan).

#### Biology.

Unknown.

#### Etymology.

Name derived from “sculptus” (Latin for “carve”), because of the sculptured second and third metasomal tergites.

**Notes.** The new species runs in the key by Chen and Weng (2005) to *Rhogadopsis sculpta* (Chen & Weng, 2005) comb. n. *Rogadopsis sculpturator*differs by having the length of mesosoma 1.2 times its height (1.4 times in *Rhogadopsis sculpta*), the length of the third antennal segment about 4 times as long as wide (about twice) and the propodeum largely smooth posteriorly (reticulate). According to Fig. 206 in Chen and Weng (2005) the second tergite of *Rhogadopsis sculpta* is smooth. If this character is considered to be variable then also *Apodesmia puncta* (Weng & Chen, 2005) comb. n. might be considered. This species has (besides the smooth second tergite and the curved occipital carina) 36 antennal segments, the first tergite 1.2 times as long as wide posteriorly and the propodeum with a large areola. *Rhogadopsis apii* (Chen & Weng, 2005)comb. n.is similar but has the first tergite about 1.1 times as long as wide, the antenna 1.0-1.1 times as long as the body, the clypeus narrow semi-elliptical and second and third tergites striate.

### Utetes

Foerster, 1862

http://species-id.net/wiki/Utetes

Figs 374 –383

Utetes Foerster, 1862: 261. Type species (by original designation): *Opius testaceus* Wesmael, 1838 [examined].
Therobolus Foerster, 1862: 260. Type species (by original designation): *Opius ruficeps* Wesmael, 1835 [examined].
Frekius Fischer, 1972a: 389 (nom. nud.), 1972b: 78; Wharton, 2006: 341 (as subgenus of *Utetes* Foerster).Type species (by original designation): *Opius castaneus* Granger, 1949 [examined].

#### Diagnosis.

Hind tibia with an oblique carina basally (Fig. 378) or with series of fine oblique striae; face without tubercles; in front of anterior ocellus with a distinct semi-circular or triangular depression, rarely absent; frons with pair of distinct depressions above antennal sockets (Fig. 381); occipital carina present laterally not or slightly curved ventrally and remaining removed from hypostomal carina, near level of middle of eye straight or nearly so, without transverse carina or crest, or completely absent; clypeus more or less convex and high or usually narrow, or longer, not impressed; labrum normal, without emargination ventrally; hypoclypeal depression distinct; scapus, fore coxa and trochanter at most weakly compressed; epistomal suture without large depressions (Fig. 379); mandible usually shorter and wider, slender basally and twisted apically, with protruding carina (Fig. 380) or distinctly widened to a baso-ventral tooth or lobe; medio-posterior depression of mesoscutum usually present (Fig. 376); pro-nope slit-like or elliptical, very large to large and deep (Fig. 381) or absent; scutellar sulcus usually rather wide; propodeum coarsely (reticulate-)rugose (Fig. 376); precoxal sulcus usually distinctly sculptured; postpectal carina completely absent; vein 2-SR of fore wing present, rarely absent; first subdiscal cell of fore wing at least partly closed by vein 3-CU1 postero-apically (Fig. 375); vein 1-M of fore wing more or less curved and in part of species vein 1-SR comparatively long; vein cu-a of hind wing nearly always present; vein 3-SR of fore wing distinctly longer than vein 2-SR; if subequal then vein m-cu of hind wing or precoxal sulcus (almost) absent; length of fore wing usually more than 3.5 mm; second and basal half of third tergite without sharp lateral crease, if sometimes weakly developed then second tergite smooth; length of second and third tergites combined less than 0.7 times length of metasoma behind first tergite; fourth and following tergites (at least partly) exposed; ovipositor sheath more or less setose basally, its length 0.1–0.7 times fore wing.

#### Biology.

Parasitoids of fruit infesting Tephritidae and to a lesser degree of Agromyzidae and Anthomyiidae; at least part of the records of latter families may be the result of misidentification of the hosts.

#### Distribution.

Cosmopolitan.

### Utetes longicarinatus

Li & van Achterberg sp. n.

urn:lsid:zoobank.org:act:550123D1-D4BA-4571-8638-B13FE5EAEDA2

http://species-id.net/wiki/Utetes_longicarinatus

Figs 374 –383

#### Type material.

Holotype, ♂ (ZUH), “S. China: Hunan, Shanyang, Chengbu, Nan Mts., 16.VII.1985, Fu-Xing Li, No. 310”.

#### Diagnosis.

Mandible triangular, normal (Fig. 380); ventral margin of clypeus truncate and thick; pronope slit-shaped and deep (Fig. 381); medio-posterior depression of mesoscutum elliptical, large and deep (Fig. 376); precoxal sulcus largely smooth; propodeum with complete medio-longitudinal carina (Fig. 376); vein m-cu of fore wing subinterstitial (Fig. 375); inner side of hind tibia with oblique carinula baso-laterally (Fig. 378).

#### Description.

Holotype, ♂, length of body 3.6 mm, of fore wing 3.8 mm.

*Head*. Antenna with 35 segments and 0.9 times as long as fore wing; length of third segment 1.3 times fourth segment, length of third, fourth and penultimate segments 2.5, 2.1and 1.5 times their width, respectively (Fig. 382); length of maxillary palp 0.7 times height of head; labial palp segments short; occipital carina moderately close to hypostomal carina and dorsally absent; hypostomal carina wide; length of eye in dorsal view 1.3 times temple; frons depressed behind antennal sockets and glabrous, smooth, medially convex (Fig. 381); face smooth, medially broadly elevated; width of clypeus 3.0 times its maximum height and 0.6 times width of face, clypeus evenly convex, sparsely punctate and its ventral margin truncate and thick (Fig. 380); hypoclypeal depression medium-sized (Fig. 379); malar suture obsolescent; mandible slightly convex (Fig. 380).

**Figure 374.**
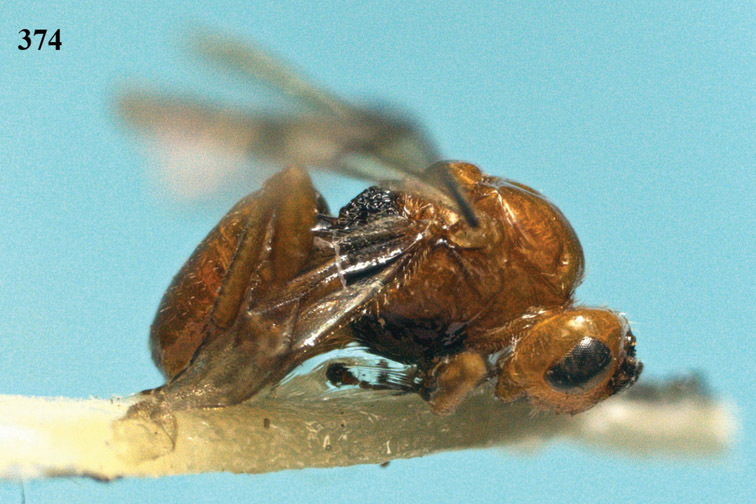
*Utetes longicarinatus* sp. n., male, holotype. Habitus lateral.

*Mesosoma*. Length of mesosoma 1.2 times its height; pronope slit-shaped and deep (Fig. 381); pronotal side smooth (Fig. 374); epicnemial area more or less crenulate dorsally; precoxal sulcus largely smooth; rest of mesopleuron smooth; pleural sulcus smooth; notauli absent on disc, except for a short smooth part anteriorly (Fig. 376); mesoscutum glabrous; medio-posterior depression of mesoscutum elliptical, large and deep (Fig. 376); scutellar sulcus moderately crenulate; scutellum smooth and evenly convex; propodeum with complete medio-longitudinal carina, surface of propodeum largely reticulate-rugose (Fig. 377).

**Figures 375–383.**
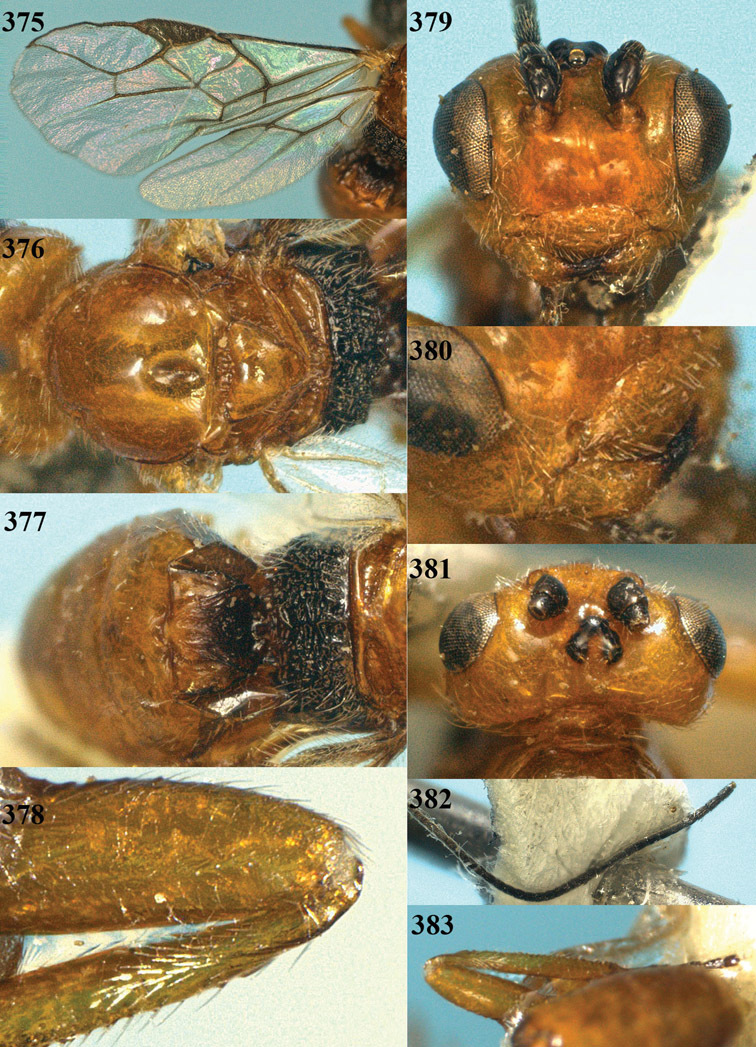
*Utetes longicarinatus* sp. n., male, holotype. **375** Wings **376** mesosoma dorsal **377 **propodeum and 1^st^-2^nd^ metasomal tergites dorsal **378** base of hind tibia inner side **379** head anterior **380** mandible **381** head dorsal **382** antenna **383** hind leg.

*Wings*. Fore wing (Fig. 375): pterostigma triangular; 1-R1 reaching wing apex and 1.3 times as long as pterostigma; r:3-SR:SR1 = 11:53:100; 2-SR:3-SR:r-m = 40:53:20; r widened; 1-M weakly curved; 1-SR+M sinuate; SR1 nearly straight; m-cu subinterstitial; cu-a postfurcal and 1-CU1 widened; first subdiscal cell closed, CU1b short; apical quarter of M+CU1 sclerotized. Hind wing (Fig. 375): M+CU:1-M:1r-m= 47:40:33; cu-a straight; m-cu absent.

*Legs*. Length of femur, tibia and basitarsus of hind leg 2.8, 8.0 and 5.6 times as long as wide, respectively (Fig. 383); setae of hind femur and tibia moderately long; inner side of hind tibia with medium-sized carinula baso-laterally (Fig. 378).

*Metasoma*. Length of first tergite 0.8 times its apical width, its surface evenly convex medially and with longitudinal carinae and dorsal carinae separated from each other and reaching apex of tergite (Fig. 377); second and following tergites smooth.

*Colour*. Yellowish brown; apex of mandible, antenna, pleural sulcus (and its surroundings) and propodeum dark brown; basal half of first tergite, tarsus, pterostigma and veins brown; wing membrane subhyaline.

*Molecular data*. None.

#### Distribution.

*China (Hunan).

#### Biology.

Unknown.

#### Etymology.

Name derived from “longus” (Latin for “long”) and “carina” (Latin for “keel, ridge”) because of the long carina of the hind tibia.

#### Notes.

Runs in the key by Chen and Weng (2005) with difficulty to *Areotetes laevigatus* (Weng & Chen, 2005) comb. n., but *Utetes longicarinatus* has the ventral margin of the clypeus thick (thin in *Areotetes laevigatus*), the propodeum densely reticulate-rugose (mainly areolate), the second metasomal tergite smooth (finely striate) and pronope large and slit-shaped (large elliptical).

### Genus Xynobius

Foerster, 1862

http://species-id.net/wiki/Xynobius

Figs 384 –393

Xynobius Foerster, 1862: 235. Type species (by original designation): *Xynobius pallipes* Foerster, 1862 (= *Opius caelatus* Haliday, 1837) [examined].
Aclisis Foerster, 1862: 267. Type species (by original designation): *Aclisis isomera* Foerster, 1862 (= *Opius caelatus* Haliday, 1837) [examined].
Holconotus Foerster, 1862: 259 (nec Schmidt-Göbel, 1846). Type species (by original designation): *Opius comatus* Wesmael, 1835) [examined].
Aulonotus Ashmead, 1900b: 368 (new name for *Holconotus* Foerster). Type species (by original designation): *Opius comatus* Wesmael, 1835) [examined].
Eristernaulax Viereck, 1914a: 362. Type species (by original designation): *Eristernaulax leucotaenia* Viereck, 1914) [examined].
Stigmatopoea Fischer, 1986: 610, 611 (as subgenus of *Opius* Wesmael), 1998: 25 (key to species); Wharton, 1988: 356; 2006: 338 (as subgenus of *Eurytenes* Foerster, 1862; possible paraphyly in *Xynobius*). Type species (by original designation): *Opius macrocerus* Thomson, 1895 [examined]. Syn. by van Achterberg (2004).
Xynobiotenes Fischer, 1998: 23 (as subgenus of *Eurytenes* Foerster, 1862). Type species (by original designation): *Opius scutellatus* Fischer, 1962 [examined]. **Syn. n.**

#### Diagnosis.

Hypoclypeal depression present, large, and medially ventral margin of cly-peus above upper level of condyli of mandibles (“subcyclostome condition”; Fig. 388); mandible simple basally, at most with a narrow ventral carina (Fig. 390); notauli complete (Fig. 386) or largely absent; medio-posterior depression of mesoscutum present (Fig. 386); below precoxal sulcus without a second sculptured sulcus; vein m-cu of fore wing usually (sub)parallel to vein 1-M (Fig. 385); vein r more or less angled with vein 3-SR of fore wing and distinctly shorter than vein 2-SR (Fig. 385); pterostigma long and narrow and more or less widened towards its apex (Fig. 385), or elliptical or triangular; dorsope present (Fig. 389); hypopygium of ♀ at most slightly incised (Fig. 392).

#### Biology.

Koinobionts endoparasitoids of leaf miners of Anthomyiidae (species of the genus *Pegomya* Robineau-Desvoidy, 1830), of Tephritidae (species of the genera *Euleia* Walker, 1835, and *Trypeta* Meigen, 1803) and Scathophagidae (species of the genus *Parallelomma* Becker, 1894 (= *Chylizosoma* Hendel, 1924)).

#### Notes.

Wharton (1988) treated *Xynobius* as a synonym of the genus *Opius* Wesmael, 1837, but it has a distinct dorsope and usually (at least in the type species) normal mandibles, and therefore it cannot be included in the genus *Opius*. The molecular data clearly show that *Xynobius* is distantly related to *Opius* (Figs 1-3 and unpublished data of S. Yaakop). Part of it (the subgenus *Stigmatopoea* Fischer, 1986) has been included by Fischer (1998) and Weng and Chen (2005) in the genus *Eurytenes* Foerster, 1862, but it fits better in the genus *Xynobius* (van Achterberg, 2004a). Wharton (1988, 2006) and Walker and Wharton (2011) placed *Xynobius* as a subgenus of *Eurytenes* but we consider it better to keep them separated till more data are known about their relationships.

### Xynobius maculipennis

(Enderlein, 1912) comb. n.

http://species-id.net/wiki/Xynobius_maculipennis

Opius maculipennis Enderlein, 1912: 262.

#### Diagnosis.

Dorsope present; notauli complete, smooth and narrow; middle lobe of mesoscutum densely setose; medio-posterior depression of mesoscutum present; scutellum strongly convex; wing membrane with conspicuous dark patch; hind tibia pale yellowish and with brown patch basally; apical third of antenna of ♀ dark brown; second metasomal tergite smooth.

#### Biology.

Unknown.

### Xynobius notauliferus

Li & van Achterberg sp. n.

http://species-id.net/wiki/Xynobius_notauliferus

Figs 384 –393

#### Type material.

Holotype, ♀ (ZUH), “S. China: Hunan, nr Zhangjiajie, Badagong Mts, Tian Ping Mt., 9–13.VII.2009, 550 m, Xi-Ying Li, RMNH’10”.

#### Diagnosis.

Dorsope present (Fig. 389); notauli complete (Fig. 386); medio-posterior depression of mesoscutum present (Fig. 386); hind tibia pale yellowish and with brown patch basally; 17^th^-19^th^ (left) or 17^th^-20^th^ (right) antennal segments of ♀ pale yellowish (Fig. 387); second metasomal tergite longitudinally costate (Fig. 389).

#### Description.

Holotype, ♀, length of body 2.2 mm, of fore wing 2.7 mm.

**Figure 384.**
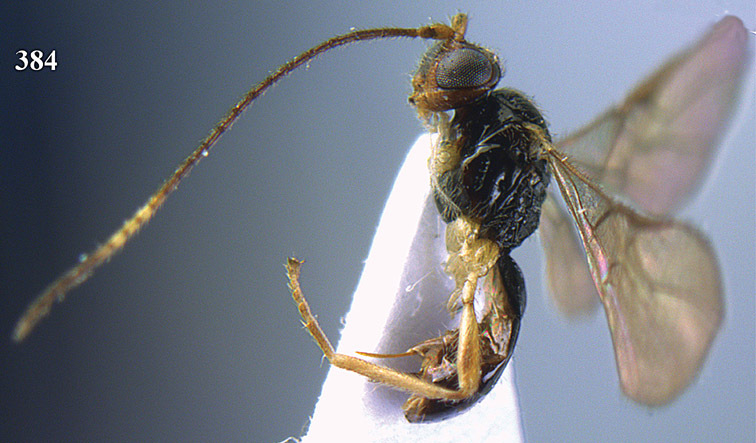
*Xynobius notauliferus* sp. n., female, holotype. Habitus lateral.

*Head*. Antenna with 26 segments and 1.3 times as long as fore wing; length of third segment 1.4 times fourth segment, length of third, fourth and penultimate segments 5.0, 3.6, and 3.2 times their width, respectively (Fig. 387); length of maxillary palp 1.4 times height of head; labial palp segments elongate (Fig. 384); occipital carina moderately close to hypostomal carina and dorsally absent; median groove behind stemmaticum present; hypostomal carina narrow; length of eye in dorsal view 4.4 times temple; frons with shallow median groove and pit, flattened and glabrous medially, smooth and laterally setose; face smooth, medially keel-shaped elevated (Fig. 388); width of clypeus 1.6 times its maximum height and 0.45 times width of face, clypeus convex, truncate ventrally, smooth and its ventral margin not differentiated and straight; hypoclypeal depression large (Fig. 388); malar suture absent; mandible normal, with medium-sized narrow ventral carina (Fig. 390).

*Mesosoma*. Length of mesosoma 1.4 times its height; dorsal pronope absent; pronotal side smooth, but medial groove with ventral oblique carina anteriorly and a short carina perpendicularly connected to it and posterior groove obsolescent (Fig. 384); epicnemial area smooth dorsally; precoxal sulcus only medially distinctly impressed, moderately widely rugose; rest of mesopleuron smooth (Fig. 384); pleural sulcus smooth; mesosternal sulcus strongly crenulate; notauli complete on disc, deep and moderately crenulate (Fig. 386); lateral mesoscutal lobes glabrous and middle lobe setose (Fig. 384); medio-posterior depression of mesoscutum small and part of notauli (Fig. 386); scutellar sulcus moderately crenulate; scutellum smooth and flat; dorsal surface of propodeum largely irregularly reticulate and with weak medio-longitudinal carina, posteriorly areolate (Fig. 386).

**Figures 385–393.**
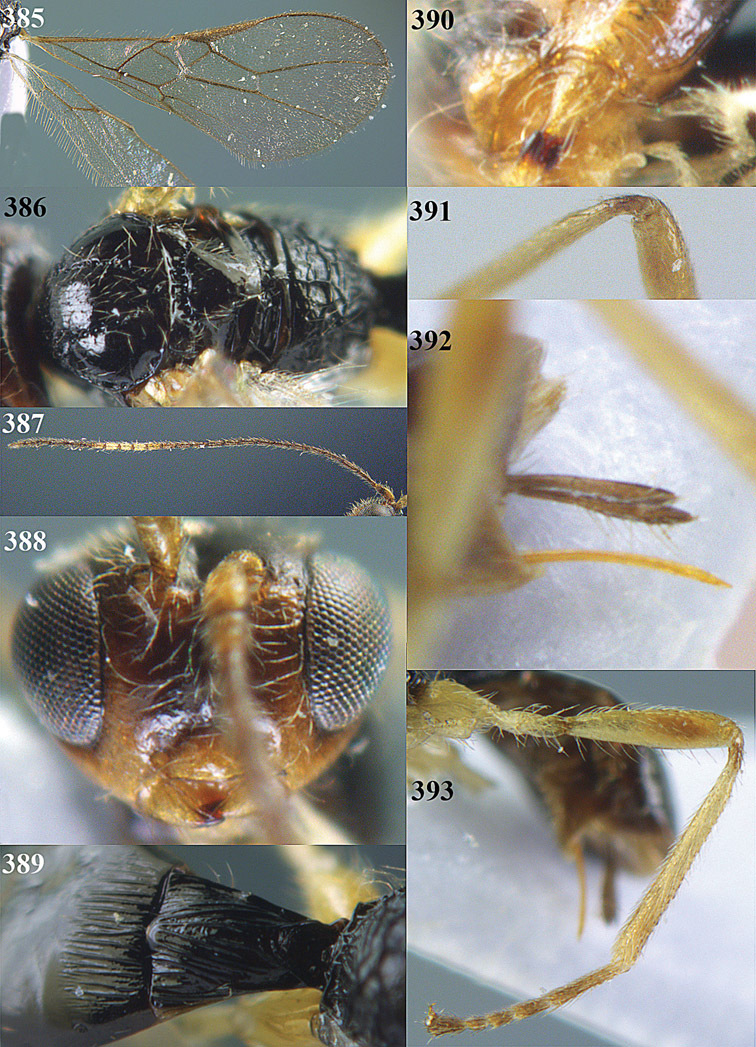
*Xynobius notauliferus* sp. n., female, holotype. **385** Wings **386** mesosoma dorsal **387** antenna **388** head anterior **389** propodeum and 1^st^-2^nd^ metasomal tergites dorsal **390** mandible **391 **base of hind tibia inner side **392** ovipositor sheath **393** hind leg.

*Wings*. Fore wing (Fig. 385): pterostigma elliptical; 1-R1 reaching wing apex and 1.4 times as long as pterostigma; r:3-SR:SR1 = 2:25:60; 2-SR:3-SR:r-m = 16:25:8; r widened; 1-M straight; SR1 nearly straight; m-cu slightly postfurcal; cu-a slightly postfurcal and 1-CU1 slightly widened; first subdiscal cell closed, CU1b short; M+CU1 unsclerotized. Hind wing (Fig. 385): M+CU:1-M:1r-m = 4:3:2; cu-a straight; m-cu absent except for a faint trace.

*Legs*. Length of femur, tibia and basitarsus of hind leg 4.5, 10.2 and 6.3 times as long as wide, respectively; hind femur with long setae and of tibia moderately long (Fig. 393).

*Metasoma*. Length of first tergite 1.3 times its apical width, its surface evenly convex medially and with longitudinal (medially oblique) costae and dorsal carinae united in its anterior 0.5 and up to apex (Fig. 389); second tergite largely longitudinally finely costate (Fig. 389); third and following tergites smooth; length of setose part of ovipositor sheath 0.08 times fore wing and 0.25 times hind tibia (Fig. 392).

*Colour*. Black or blackish-brown; 17^th^-19^th^ (left) or 17^th^-20^th^ (right) antennal segments, palpi, tegulae and legs (but apical half of outer side of hind femur, base of hind tibia and hind tarsus more or less dark brown) pale yellowish; scapus, orbita, face laterally, clypeus, mandible, malar space and temple brownish-yellow; remainder of head dark brown; pterostigma and veins mainly brown; wing membrane slightly infuscate.

*Molecular data*. COI, 16S, 28S (CVA 4240).

#### Distribution.

*China (Hunan).

#### Biology.

Unknown.

#### Etymology.

Name derived from “notauli” (Latin for “grooves of the notum”) and “fero” (suffix in Latin meaning “carrying or having”), because of the developed notauli.

#### Notes.

The new species runs in the key by Chen and Weng (2005) to *Opius mitis* Chen & Weng, 2005 (= *Xynobius wengi* nom. n.). *Xynobius notauliferus* differs by having the notauli complete (posteriorly absent in *Xynobius wengi*), the lateral lobes of the meso-scutum are glabrous (largely setose), the length of eye in dorsal view 4.4 times temple (2.3 times) and the antenna with a pale yellowish ring (absent).

**Figure 394.**
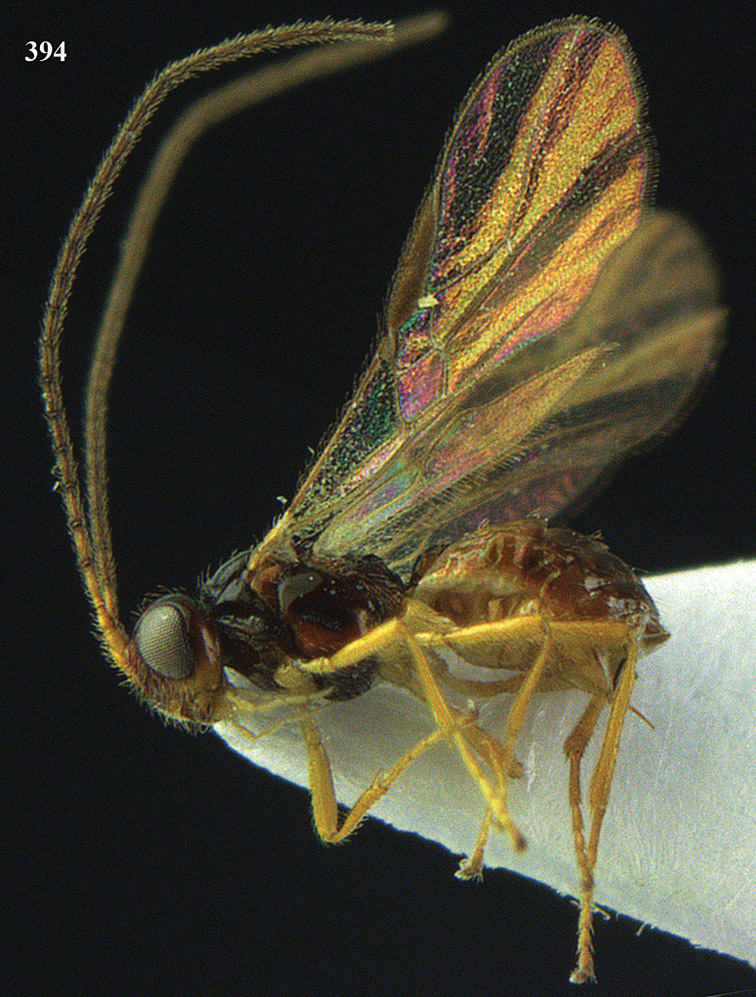
*Phaedrotoma protuberator* sp. n., female, paratype. Habitus lateral.

**Figures 395–402.**
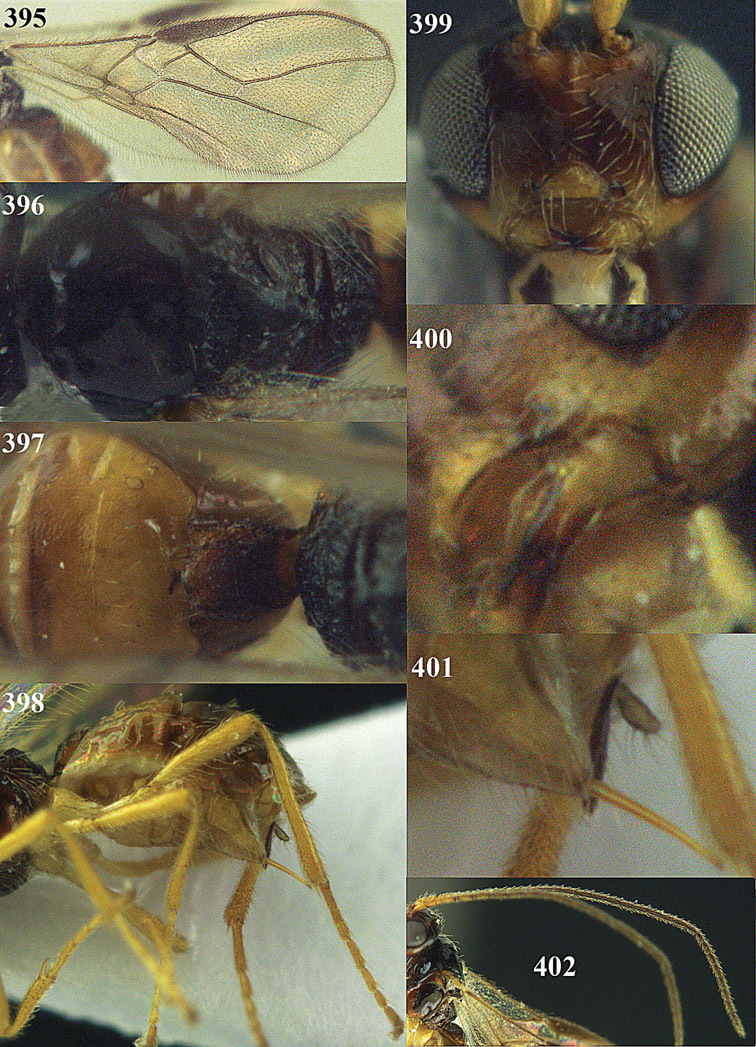
*Phaedrotoma protuberator* sp. n., female, paratype. **395** Wings **396** mesosoma dorsal **397** propodeum and 1^st^-3^rd^ metasomal tergites dorsal **398** hind leg **399** head anterior **400** mandible **401 **ovipositor sheath **402** antennae.

**Figure 403.**
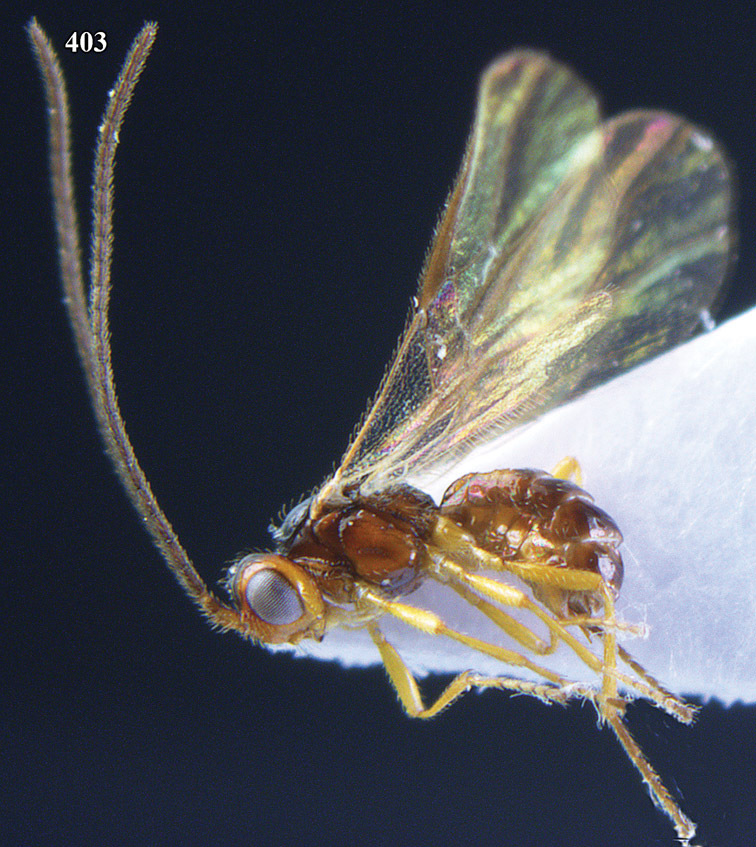
*Phaedrotoma protuberator* sp. n., female, paratype. Habitus lateral.

**Figures 404–412.**
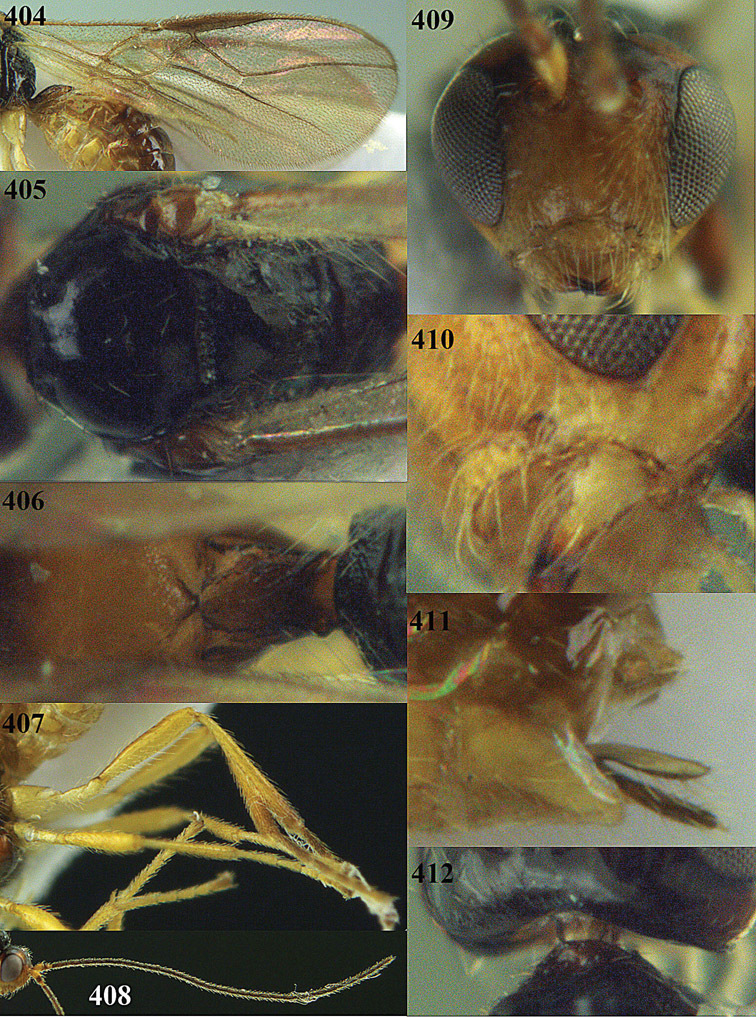
*Phaedrotoma protuberator* sp. n., female, paratype. **404** Wings **405** mesosoma dorsal **406** propodeum and 1^st^-2^nd^ metasomal tergites dorsal **407** hind leg **408** antenna **409** head anterior **410 **mandible **411** ovipositor sheath **412** pronope dorsal.

**Figures 413–417.**
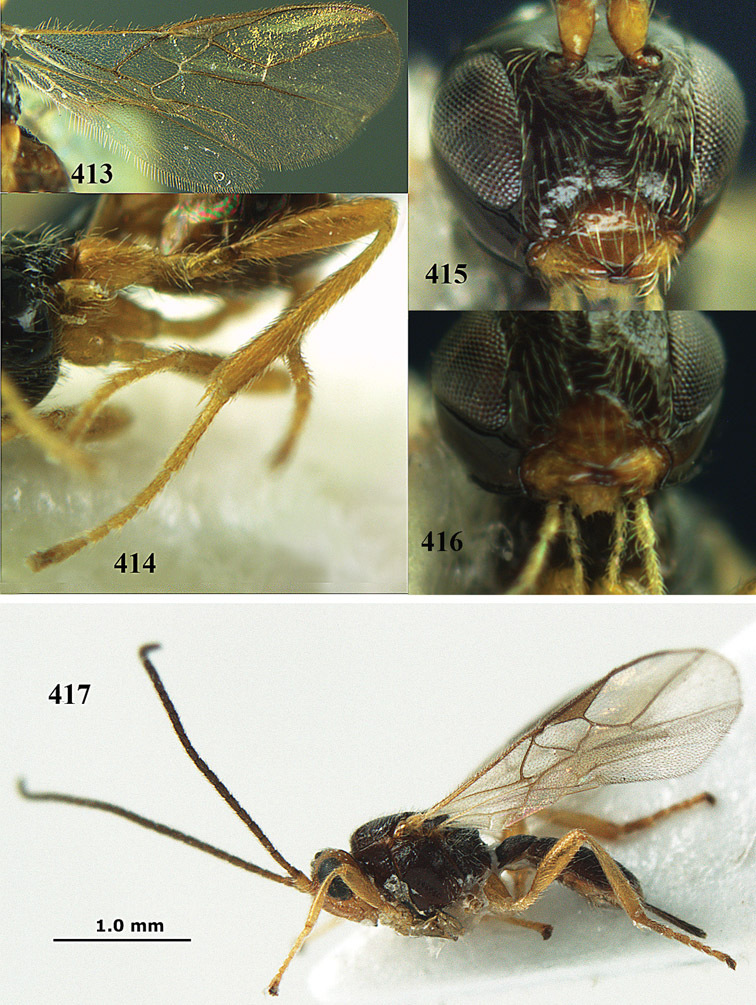
*Opius pallipes* Wesmael, female, Netherlands, Waarder. **413** Wings **414** hind leg **415** head anterior **416** labial palpi anterior **417** *Orientopius punctatus* van Achterberg & Li, female, holotype. Habitus lateral.

**Figures 418–428.**
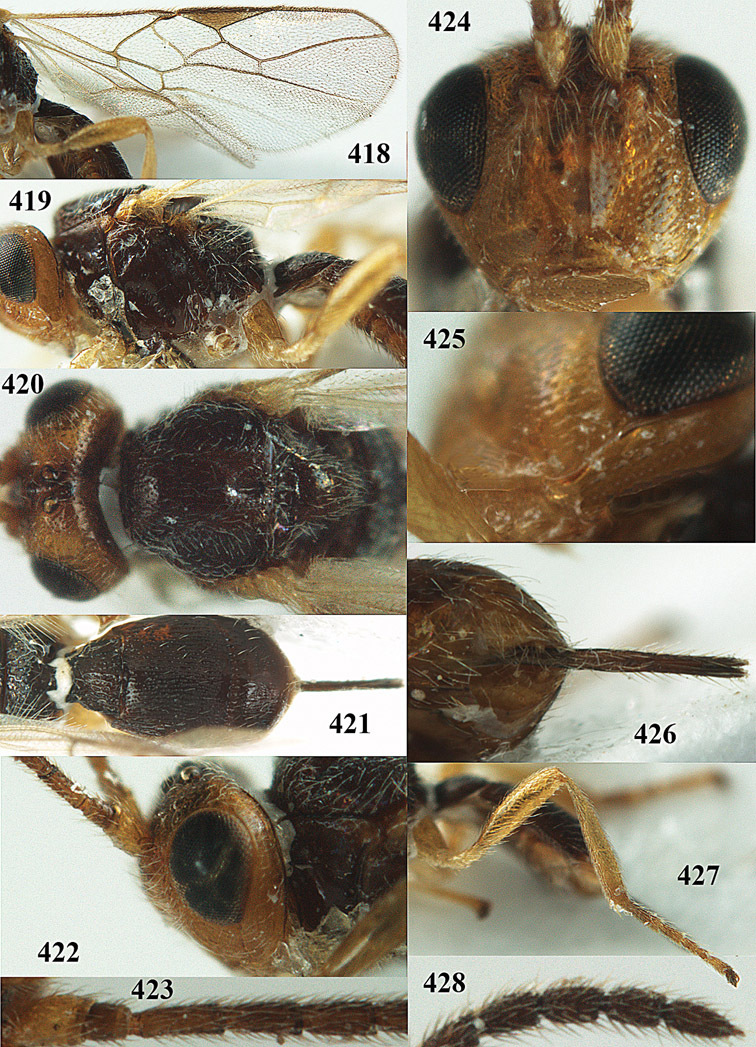
*Orientopius punctatus* van Achterberg & Li, female, holotype. **418** Wings **419** mesosoma lateral **420** mesosoma dorsal **421** metasoma dorsal **422** head lateral **423** base of antenna **424** head anterior **425** malar space **426** ovipositor sheath ventral **427** hind leg **428** apex of antenna.

##### Notes on two homonyms described from China

### Opius cheni

van Achterberg & Li nom. n.

Opius ambiguus Weng & Chen (in Chen & Weng), 2005: 95–96.

#### Notes.

*Opius ambiguus* Weng & Chen, 2005 (not Wesmael 1835) is a primary homonym**.** We here rename the species *Opius cheni* nom. n.after Prof. Dr Jia-Hua Chen for his contribution to our knowledge of the Chinese Braconidae and for his hospitality to the two first authors during several visits to the Fujian Agricultural & Forestry University at Fuzhou.

### Xynobius wengi

van Achterberg & Li nom. n.

Opius mitis Chen & Weng, 2005: 72-74.

#### Notes.

*Opius mitis* Chen & Weng, 2005 (not Fischer 1963) is a primary homonym**.** We here rename the species *Xynobius wengi* nom. n.after Dr Rui-Quan Weng for his contribution to our knowledge of the Chinese Opiinae and for his hospitality to the authors during visits to the Fujian Agricultural & Forestry University at Fuzhou. The holotype has been examined and proved to belong to the genus *Xynobius* Foerster, 1862.

## Supplementary Material

XML Treatment for Opiinae

XML Treatment for Apodesmia

XML Treatment for Apodesmia bruniclypealis

XML Treatment for Apodesmia melliclypealis

XML Treatment for Areotetes

XML Treatment for Areotetes albiferus

XML Treatment for Areotetes carinuliferus

XML Treatment for Areotetes striatiferus

XML Treatment for Biosteres

XML Treatment for Biosteres pavititus

XML Treatment for Coleopioides

XML Treatment for Coleopioides diversinotum

XML Treatment for Coleopioides postpectalis

XML Treatment for Diachasmimorpha

XML Treatment for Diachasmimorpha longicaudata

XML Treatment for Fopius

XML Treatment for Fopius dorsopiferus

XML Treatment for Indiopius

XML Treatment for Indiopius chenae

XML Treatment for Neopius

XML Treatment for Opiognathus

XML Treatment for Opiognathus aulaciferus

XML Treatment for Opiognathus brevibasalis

XML Treatment for Opiostomus

XML Treatment for Opiostomus aureliae

XML Treatment for Opius

XML Treatment for Opius crenuliferus

XML Treatment for Opius malarator

XML Treatment for Opius monilipalpis

XML Treatment for Opius pachymerus

XML Treatment for Opius songi

XML Treatment for Opius youi

XML Treatment for Opius zengi

XML Treatment for Orientopius

XML Treatment for Orientopius punctatus

XML Treatment for Phaedrotoma

XML Treatment for Phaedrotoma acuticlypeata

XML Treatment for Phaedrotoma angiclypeata

XML Treatment for Phaedrotoma antenervalis

XML Treatment for Phaedrotoma depressa

XML Treatment for Phaedrotoma depressiclypealis

XML Treatment for Phaedrotoma flavisoma

XML Treatment for Phaedrotoma nigrisoma

XML Treatment for Phaedrotoma protuberator

XML Treatment for Phaedrotoma rugulifera

XML Treatment for Phaedrotoma semiplanata

XML Treatment for Phaedrotoma striatinota

XML Treatment for Phaedrotoma terga

XML Treatment for Phaedrotoma vermiculifera

XML Treatment for Rhogadopsis

XML Treatment for Rhogadopsis latipennis

XML Treatment for Rhogadopsis longicaudifera

XML Treatment for Rhogadopsis longuria

XML Treatment for Rhogadopsis maculosa

XML Treatment for Rhogadopsis obliqua

XML Treatment for Rhogadopsis sculpturator

XML Treatment for Utetes

XML Treatment for Utetes longicarinatus

XML Treatment for Xynobius

XML Treatment for Xynobius maculipennis

XML Treatment for Xynobius notauliferus

XML Treatment for Opius cheni

XML Treatment for Xynobius wengi
